# Global and regional reference curves for pain across the lifespan in 6.1 million individuals in 118 countries

**DOI:** 10.1038/s41591-026-04696-w

**Published:** 2026-10-05

**Authors:** Matt Fillingim, Christophe Tanguay-Sabourin, Lindsay Neuert, Azin Zare, Jax Norman, Gianluca V. Guglietti, Lise Hobeika, Abimbola Ayorinde, Ahmad Salimzadeh, Ahmad-reza Jamshidi, Aqeel M. Alenazi, Arash Tehrani-Banihashemi, Bader A. Alqahtani, Beatriz Olaya, Benjamin Longo Mbenza, Bibilola Oladeji, Brenda Penninx, Buddhi Paudyal, Caique de Melo, Chantal Berna, Clarissa Humberg, Pascal Claudepierre, Elisa Smith, Elizabeth VanDenKerkhof, Erik J. Giltay, Esther Garcia-Esquinas, Faisal Parlindungan, Fatima Qayyum Abbasi, Fernando Rodríguez Artalejo, Gareth T. Jones, Gary Slade, Hanifa Bouziri, Herta Flor, Hiroyasu Iso, Ian Gilron, Imad Uthman, Ingris Peláez-Ballestas, Jean-Paul Divengi Nzambi, Jose Luis Ayuso-Mateos, Josep Maria Haro, Julia Wager, Juliana Barcellos de Souza, Kazutoshi Nakamura, Keiko Yamada, Lisa-Marie Rau, Makram Talih, Marcus J. Beasley, María Dueñas, Marie Zins, Marta Miret, Melissa Wake, Mirza Zaka Pratama, Mohammed Almalki, Monique Chaaya, Muhammad Anshory, Naoki Kondo, Navid Moghadam, Norma Mansor, Omar Alsaed, Orawan Keeratisiroj, Oye Gureje, Pande Ketut Kurniari, Pardis Noormohammadpour, Pierrot Lebughe, Raga A. Elzahaf, Ramin Kordi, Raquel Lucas, Rashmi Parekh Bhandari, Rosa Weiss Telles, Rudy Hidayat, Saba Samreen, Samar Al-Emadi, Sandhi Maria Barreto, Shima RoknSharifi, Shinsuke Inoue, Sudeep Adhikari, Suryo Anggoro Kusumo Wibowo, Sweekriti Sharma, Sylvia Villeneuve, Takahiro Tabuchi, Tayyeba Khursheed, Tie Yamato, Todd Jackson, Veronica Souza Santos, Wantana Siritaratiwat, Xianchen Liu, Zhen-Zhen Liu, IMAGEN Consortium, Herta Flor, Tobias Banaschewski, Gareth J. Barker, Arun L. W. Bokde, Rüdiger Brühl, Sylvane Desrivières, Penny Gowland, Antoine Grigis, Andreas Heinz, Herve Lemaitre, Jean-Luc Martinot, Marie-Laure Paillere Martinot, Eric Artiges, Frauke Nees, Dimitri Papadopoulos Orfanos, Tomáš Paus, Luise Poustka, Michael N. Smolka, Sarah Hohmann, Nilakshi Vaidya, Henrik Walter, Robert Whelan, Paul Wirsching, Gunter Schumann, Susan M. Sawyer, Saman Khalatbari-Soltani, Ólöf Anna Steingrímsdóttir, Gary J. Macfarlane, Fiona Blyth, Etienne Vachon-Presseau

**Affiliations:** 1 McGill University, Montreal, Quebec, Canada; 2 University of Montreal, Montreal, Quebec Canada; 3 University of Warwick, Coventry, UK; 4 Tehran University of Medical Sciences, Tehran, Iran; 5 Prince Sattam Bin Abdulaziz University, Al-Kharj, Saudi Arabia; 6 Preventive Medicine and Public Health Research Center, Iran University of Medical Sciences, Tehran, Iran; 7 Universitat Autònoma de Barcelona, Bellaterra, Spain; 8 Université de Kinshasa, Kinshasa, Democratic Republic of the Congo; 9 University of Ibadan, Ibadan, Nigeria; 10 Amsterdam UMC, Amsterdam, The Netherlands; 11 Patan Academy of Health Sciences, Lalitpur, Nepal; 12 Universidade Cidade de São Paulo, São Paulo, Brazil; 13 University of Lausanne, Lausanne, Switzerland; 14 Witten/Herdecke University, Witten, Germany; 15 Université Paris Est Créteil, Créteil, France; 16 University of Aberdeen, Aberdeen, UK; 17 Queen’s University, Kingston, Ontario Canada; 18 Leiden University Medical Center, Leiden, The Netherlands; 19 Instituto de Salud Carlos III, Madrid, Spain; 20 Universitas Indonesia, Depok City, Indonesia; 21 Pakistan Institute of Medical Sciences, Islamabad, Pakistan; 22 Universidad Autónoma de Madrid-CIBERESP, Madrid, Spain; 23 University of North Carolina at Chapel Hill, Chapel Hill, NC, USA; 24 Université Paris-Cité, Paris, France; 25 Central Institute of Mental Health, Mannheim, Germany; 26 Heidelberg University, Heidelberg, Germany; 27 Japan Institute for Health Security, Tokyo, Japan; 28 American University of Beirut, Beirut, Lebanon; 29 Hospital General de Mexico Dr. Eduardo Liceaga, Mexico City, Mexico; 30 Universidad Autónoma de Madrid-CIBERSAM, Madrid, Spain; 31 Parc Sanitari Sant Joan de Déu, Sant Boi de Llobregat, Spain; 32 Universidade do Estado de Santa Catarina (UDESC), Florianópolis, Brazil; 33 Niigata University Graduate School of Medical and Dental Sciences, Niigata, Japan; 34 Juntendo University Graduate School of Medicine, Tokyo, Japan; 35 Instituto de Saúde Pública da Universidade do Porto, Porto, Portugal; 36 Universidad de Cádiz, Cádiz, Spain; 37 Universidad Autónoma de Madrid, Madrid, Spain; 38 The University of Melbourne, Melbourne, Victoria Australia; 39 Faculty of Medicine Universitas Brawijaya, Malang, Indonesia; 40 King Salman Armed Forces Hospital, Tabuk, Saudi Arabia; 41 Universitas Brawijaya, Malang, Indonesia; 42 Kyoto University, Kyoto, Japan; 43 Universiti Malaya, Kuala Lumpur, Malaysia; 44 Hamad Medical Corporation, Doha, Qatar; 45 Faculty of Public Health, Naresuan University, Phitsanulok, Thailand; 46 Universitas Udayana, Denpasar, Indonesia; 47 College of Medical Technology, Tripoli, Libya; 48 Universidade do Porto, Porto, Portugal; 49 Stanford University Medical Center, Palo Alto, CA USA; 50 Universidade Federal de Minas Gerais, Belo Horizonte, Brazil; 51 Foundation University Islamabad, Islamabad, Pakistan; 52 Kaguyama Orthopaedic Clinic, Nisshin City, Japan; 53 Oxford University Clinical Research Unit Nepal, Lalitpur, Nepal; 54 The University of Sydney, Sydney, New South Wales Australia; 55 Tohoku University Graduate School of Medicine, Sendai, Japan; 56 University of Macau, Taipa, China; 57 McMaster University, Hamilton, Ontario Canada; 58 Khon Kaen University, Khon Kaen, Thailand; 59 Shandong University School of Public Health, Jinan, China; 60 Changchun University of Chinese Medicine, Changchun, China; 61 Norwegian Institute of Public Health, Oslo, Norway; 62 King’s College London, London, UK; 63 Trinity College Dublin, Dublin, Ireland; 64 Physikalisch-Technische Bundesanstalt, Braunschweig, Germany; 65 University of Nottingham, Nottingham, UK; 66 Universite Paris-Saclay, Orsay, France; 67 University of Tubingen, Tübingen, Germany; 68 Ludwig-Maximilians-Universitat, Munich, Germany; 69 University Hospital Heidelberg, Heidelberg, Germany; 70 Technische Universitat Dresden, Dresden, Germany; 71 Charite Universitatsmedizin Berlin, Berlin, Germany

**Keywords:** Pain, Risk factors

## Abstract

Pain is the leading cause of disability worldwide, yet no harmonized self-reported reference framework exists to characterize how its burden is distributed across the lifespan and world regions. In this study, we harmonized individual-level self-reported pain data from 6,125,459 participants across 902 population-based data sources in 118 countries to establish global reference trajectories of pain. We implemented these trajectories in an open-access benchmarking platform for positioning external datasets against global pain norms. Pain prevalence ranged from roughly 2% for facial pain to 40% for back pain, was consistently higher in women across all 11 anatomical sites (risk ratio range: 1.08−1.83) and increased most steeply before age 55 years. Contrary to existing estimates that generally project higher prevalence of pain conditions in higher Human Development Index (HDI) regions, we found that individuals in the lowest HDI countries experienced a substantially higher late-life prevalence of any bodily pain than those in the highest (risk difference: 30.6 percentage points (95% confidence interval: 26.9−34.3)), with low back pain approaching twice the prevalence at the oldest ages. Globally, 18.3% of pain burden across anatomical sites was attributable to three modifiable risk factors (smoking, obesity and low income), but this varied from 12.6% in sub-Saharan Africa to 27.1% in eastern Europe, indicating that the factors underlying pain in lower-HDI settings remain poorly characterized. Together, these reference trajectories provide a common framework for defining and comparing pain burden and informing more equitable global surveillance and prevention.

## Abstract (web summary)

Global age- and sex-specific reference curves for 11 pain sites, from 6.1 million people in 118 countries, found higher late-life pain in the lowest Human Development Index (HDI) countries and provide a tool for benchmarking pain assessments in other cohorts.

## Main

Pain is a leading cause of years lived with disability worldwide^1^. Unlike most major health conditions, it lacks an objective biomarker and cannot be assessed independently of self-report^2^. As a result, its population burden depends on how pain is captured across studies and health systems. Existing frameworks, including the Global Burden of Disease (GBD) study, rely on clinically defined conditions and modeled estimates of heterogeneous data sources. These approaches are essential for disease surveillance but may not fully capture pain that does not enter diagnostic pathways, particularly in settings with limited access to care. Site-specific self-reported pain provides a complementary framework because anatomical pain reports are more consistently available across population-based studies than diagnostic information and can be compared across settings where clinical work-up, diagnostic criteria and health system access vary.

Although self-reported pain is routinely collected in population-based studies, these data are fragmented across heterogeneous instruments, recall periods, anatomical definitions and study designs that have not been systematically harmonized at global scale. Reported prevalence in the general population, therefore, varies widely, ranging from 8% to 64% across studies^3–5^, with much of the evidence being derived from high-income settings, often focusing on single phenotypes such as low back pain. Dimensions more closely related to disability and care seeking, such as pain intensity, generalized pain and multi-site pain, are also rarely captured in comparable ways across populations^6,7^. Consequently, the absence of a consistent framework leaves fundamental questions unresolved: When does pain emerge across the lifespan, and how do these trajectories differ by phenotype, sex and world region? Establishing reference trajectories for pain would provide a common ground for comparing cohorts, clinical populations and countries and for assessing whether patterns derived from self-report align with those from condition-based surveillance, particularly in settings underrepresented in global estimates.

In the present study, we applied generalized linear mixed-effects models (GLMMs) to individual-level self-reported pain data from a global network of population-based studies to construct reference trajectories of pain across the lifespan, implemented in an open-access benchmarking platform. Here we present distinct lifespan trajectories that differ by anatomical site and population context, with most burden accumulating rapidly before age 55 years. Moreover, we report that self-reported pain is substantially higher in lower human development settings than condition-based estimates suggest, with the drivers of this excess poorly understood and distinct from the modifiable risk factors that dominate pain burden in high-income settings.

## Results

### Study population and data coverage

**Fig. 1:**
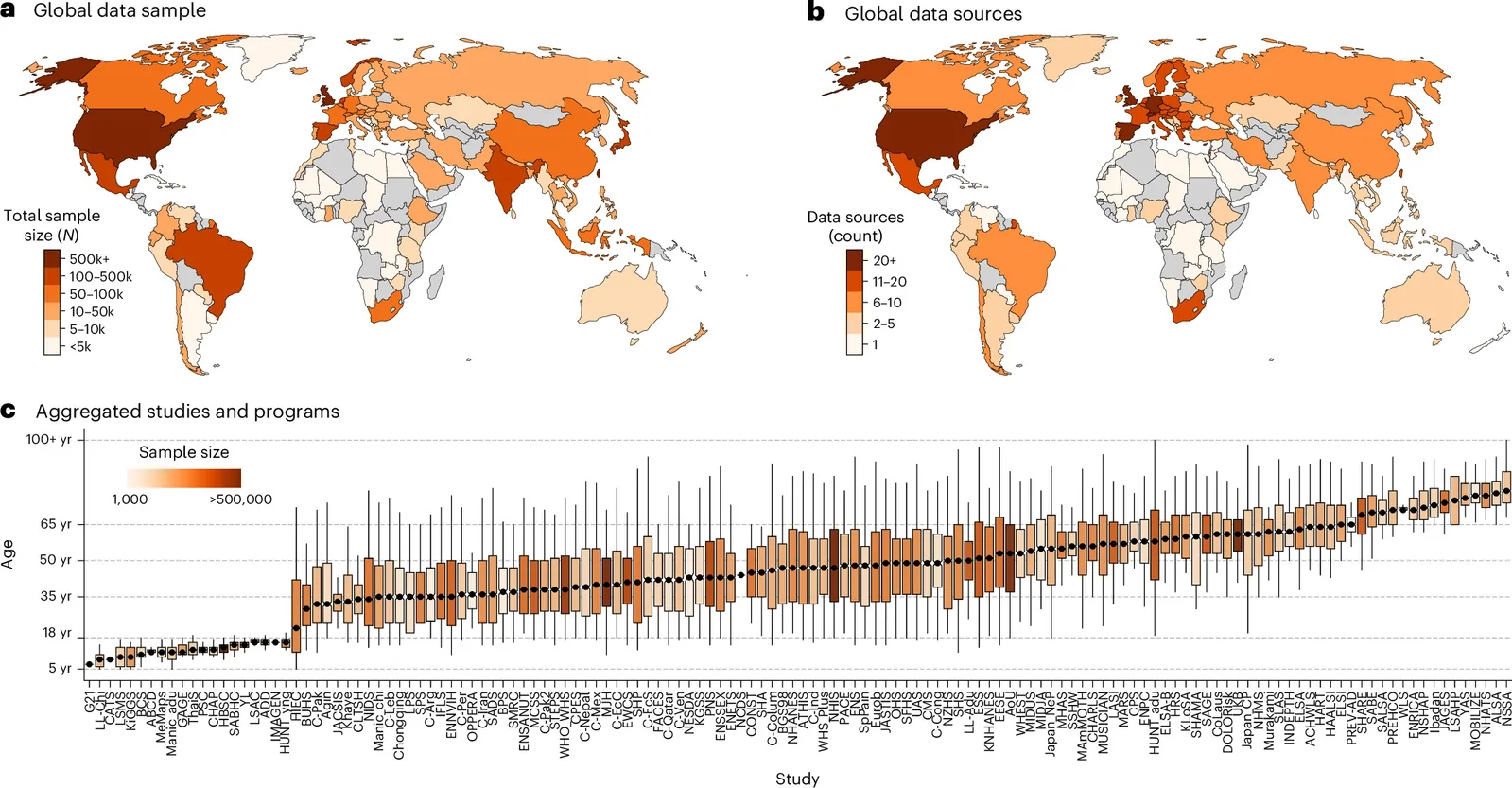
Global coverage and age structure of the pooled dataset. **a**, Country-level total sample size (participants with valid data for at least one pain outcome), shown in categories from <5,000 to ≥500,000. **b**, Number of contributing data sources per country (distinct surveys and cohorts), shown in categories from 1 to ≥20. Countries without eligible data are shown in gray. **c**, Age distribution for each contributing study program or data source, plotted as box plots (interquartile range with whiskers indicating the age range) and ordered by median age (black points). Box color indicates study sample size (1,000 to >500,000 participants). Each box summarizes the age distribution of one contributing study program or data source; across all sources, the total sample is *n* = 6,125,459 participants (902 data sources, 138 study programs). yr, years.

We compiled individual participant data from population-based national, regional and international health studies and surveys conducted between 1990 and 2025 that assessed self-reported bodily pain (Supplementary Table 2). We analyzed a total of 6,125,459 participants across 902 data sources from 138 population-based study programs, spanning 118 countries and territories (Fig. 1a). Participants were aged 5 to more than 100 years, and 55% were female. Demographic characteristics of the analytic sample are summarized by world region in Supplementary Table 1. Studies represented all inhabited continents, and data density ranged from 2.1 data sources per country in sub-Saharan Africa to 19.4 in western Europe (Fig. 1b). The age distribution of contributing study populations is shown in Fig. 1c.

### Lifespan pain trajectories

Pain prevalence estimates are known to vary with methodological factors. To generate global reference curves, our harmonization procedure mapped study-specific wording and body map coding schemes to standardized categories (Supplementary Information, section 3) after evaluating seven key methodological features (Methods and Supplementary Information, section 6.2) through a series of sensitivity analyses (Supplementary Information, section 6 and Supplementary Figs. 1–13). Binary indicators of self-reported pain were harmonized at 11 major anatomical sites: head, face, neck or shoulder, foot or ankle, hand or wrist, elbow, chest, back, stomach or abdomen, hip and knee. Age-specific trajectories of pain prevalence were estimated across the lifespan (ages 5 to 100+; Supplementary Information, section 6.5) using GLMMs with a logit link, incorporating natural cubic splines to flexibly capture nonlinear age trajectories. Random effects accounted for the hierarchical correlation structure of the data, with individuals nested within cohorts and world regions.

**Fig. 2:**
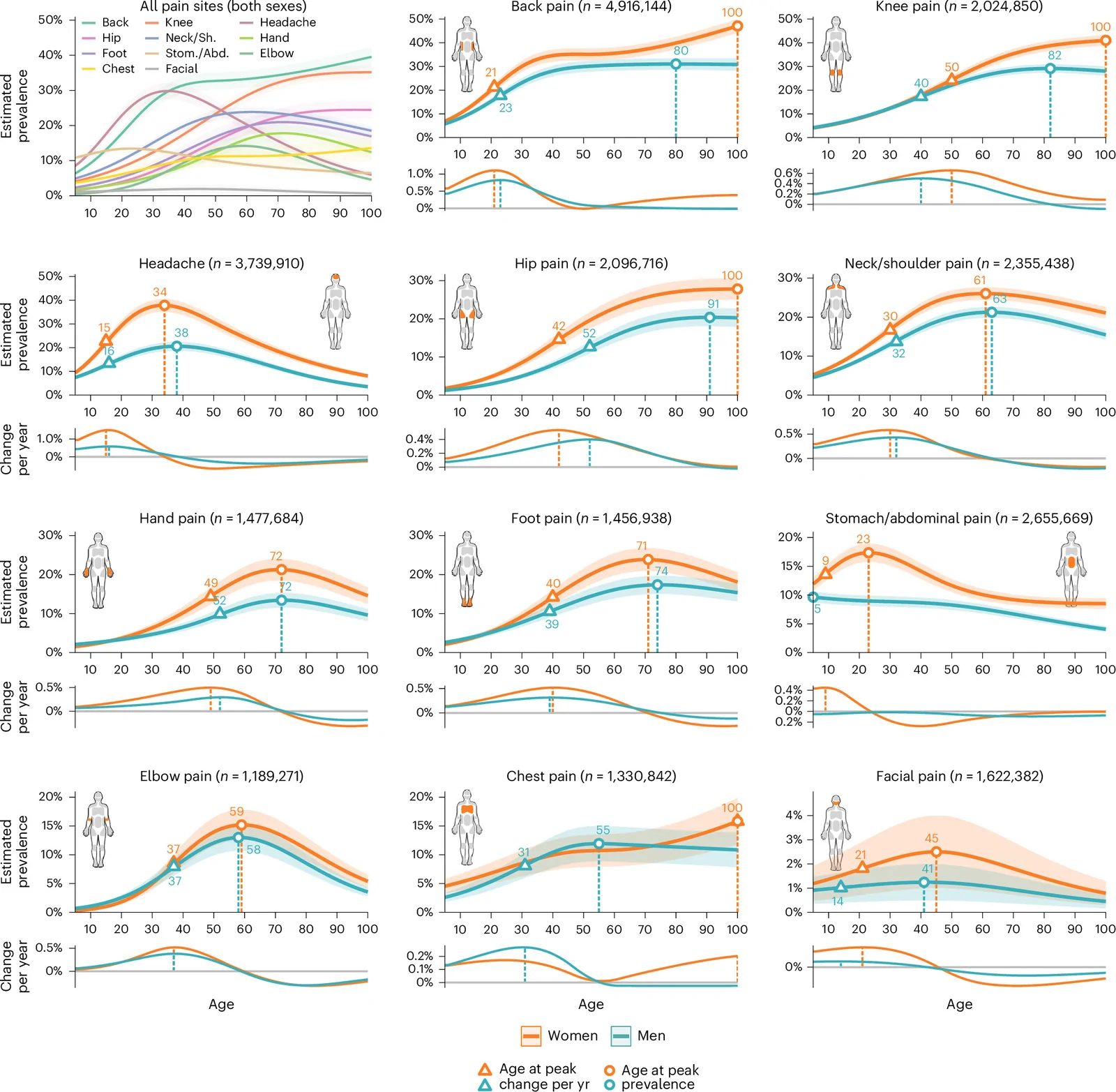
Lifespan trajectories of pain prevalence for 11 anatomical sites. Each site-specific trajectory was estimated using a mixed-effects logistic regression model with natural cubic splines (3 degrees of freedom, selected via study-clustered five-fold cross-validation) to capture nonlinear age effects, fitted to individual-level data from 902 population-based sources nested within cohorts and world regions. Trajectories are marginal predictions standardized to a past-month recall period and general case definition. For each site, the upper panel shows model-estimated prevalence (%) across the lifespan (ages 5 to 100+), and the lower panel shows the corresponding age-specific rate of change (percentage-point change per year). Shaded bands indicate 95% CIs. Open triangles mark the age of peak rate of increase in prevalence; open circles mark the age of peak prevalence. Colors denote sex (women: orange; men: blue). Annotated ages are shown in the prevalence panels, with corresponding vertical dashed lines in the rate-of-change panels. Sample sizes reflect the number of distinct individual-level records per site and are shown in panel titles. In all panels, plotted lines denote the model-estimated marginal mean prevalence, and shaded bands denote 95% CIs. yr, year.

**Extended Data Fig. 1:**
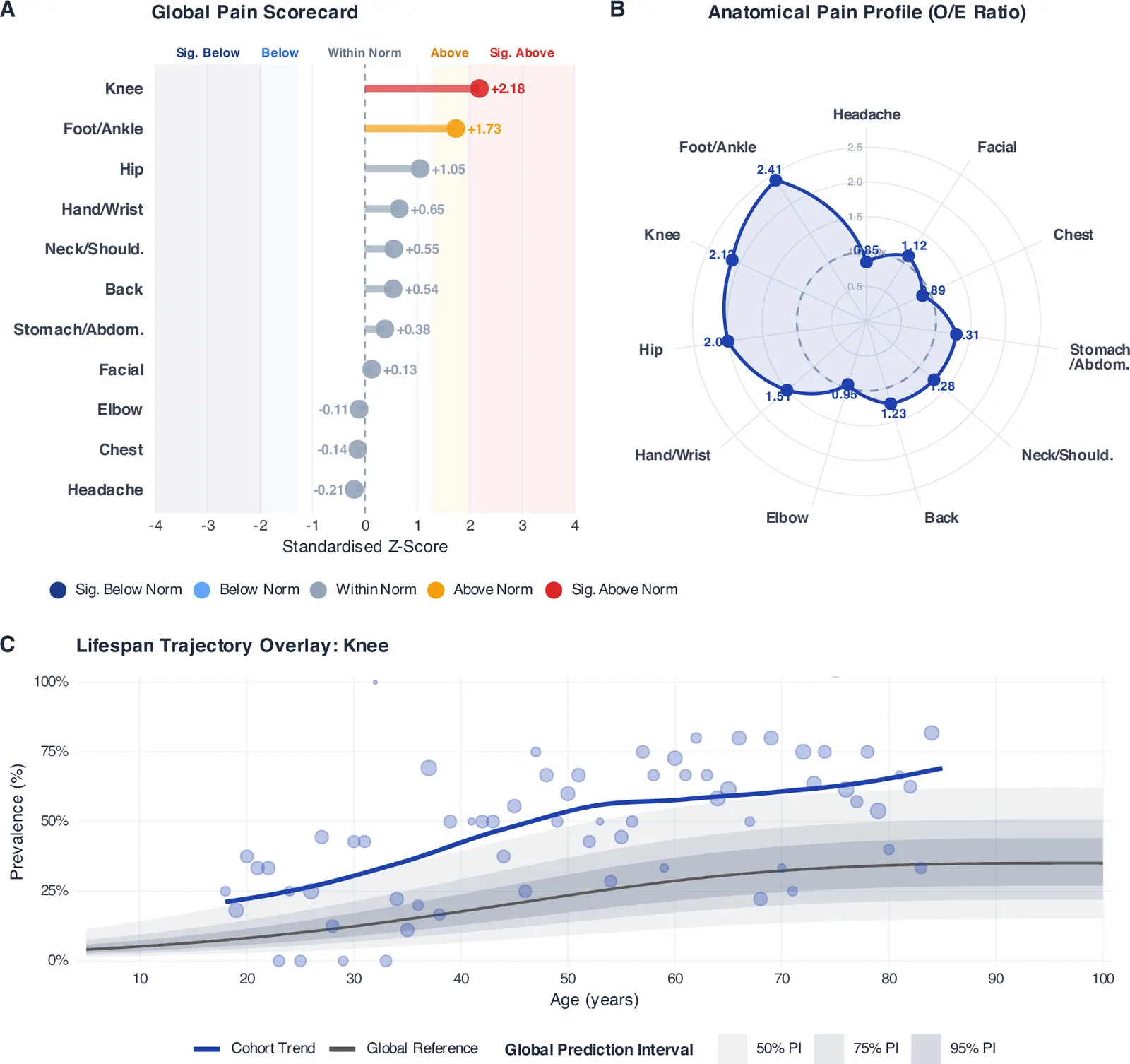
Representative output of the Global Lifespan Pain Benchmarking Tool applied to a hypothetical external cohort. The tool benchmarks external cohort data against the global normative reference derived from the primary analysis (see Supplementary Information, section 10 for full statistical methodology and application architecture). (**A**) Global Pain Scorecard: lollipop chart of standardised Z-scores for 11 anatomical pain sites, with colour-coded deviation zones (Significantly Below Norm, |Z| ≥ 1.96; Below Norm, 1.28 ≤ |Z| < 1.96; Within Norm, |Z| < 1.28; Above Norm; Significantly Above Norm). (**B**) Anatomical Pain Profile: radar chart mapping O/E ratios across all 11 sites in anatomical order (head to foot). (**C**) Lifespan Trajectory Overlay: age-prevalence chart for knee pain superimposing the cohort’s observed LOESS trend (blue) against the model-estimated marginal mean global reference trajectory (grey); shaded bands denote its 50%, 75%, and 95% prediction intervals derived from between-study heterogeneity on the logit scale. Points are sized by sample size per age. The tool is freely accessible at https://evppainlab.shinyapps.io/global-pain-benchmark/.

Drawing on individual-level data from 1.2−4.9 million distinct participants per pain site, pain prevalence varied markedly across anatomical sites and followed distinct age trajectories by site and sex (Fig. 2). Overall prevalence ranged from 2% for facial pain to 40% for back pain. Women consistently reported higher prevalence than men across all 11 sites (risk ratio (RR) range: 1.08−1.83), with the largest sex differences observed for facial (RR = 1.83 (95% confidence interval (CI): 1.79−1.88)), headache (RR = 1.74 (95% CI: 1.73−1.75)) and stomach/abdominal (RR = 1.60 (95% CI: 1.59−1.62)) pain. Musculoskeletal sites including back, hip and knee showed progressive nonlinear increases through adulthood, with the most rapid gains between ages 20 and 55 years and peaking at or beyond age 75. Using the age-specific rates of change from the fitted trajectories, most sites (seven of 11) exhibited an inverted-U trajectory, with prevalence peaking in mid-to-late adulthood and declining thereafter. This pattern was most pronounced for headache (peak age 34 years) and abdominal pain (23 years) and across upper-body sites including neck or shoulder (62 years), elbow (59 years) and facial (45 years). These results refine the prevailing assumption that pain accumulates monotonically with age and instead suggest that several common pain phenotypes peak by mid-to-late adulthood and decline thereafter. Across all sites, prevalence rose most steeply before age 55, indicating that the majority of pain burden accumulates before midlife. These trajectories are publicly accessible through the Global Pain Benchmarking Tool (https://evppainlab.shinyapps.io/global-pain-benchmark/; Extended Data Fig. 1 and Supplementary Information, section 10).

### Any bodily pain, high-intensity pain and generalized pain across the lifespan

**Fig. 3:**
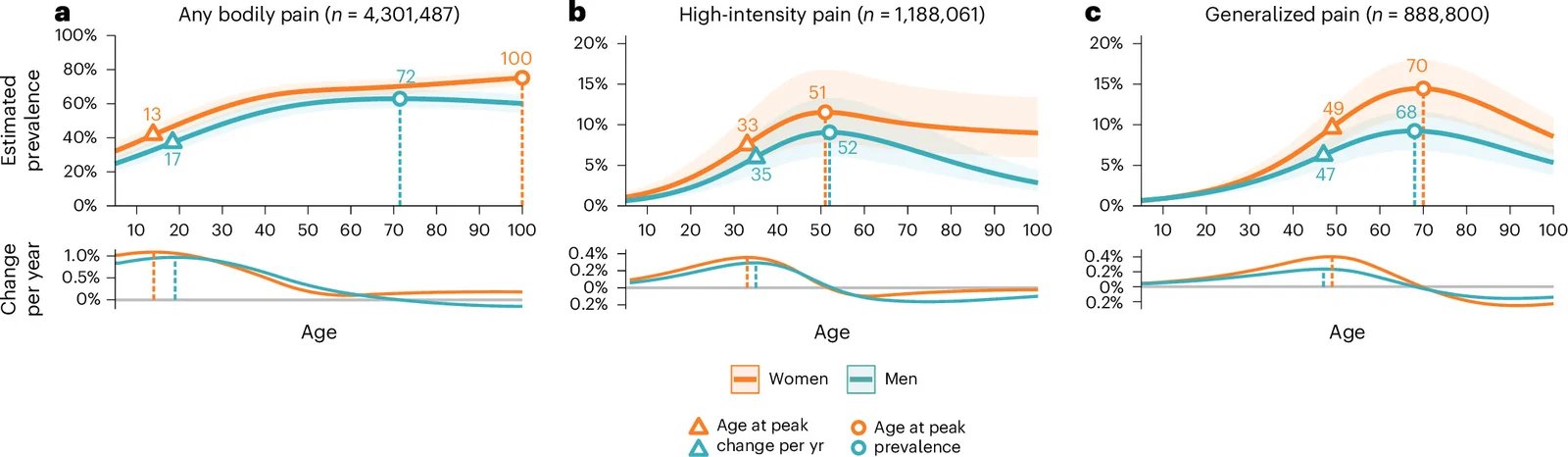
Lifespan trajectories of any bodily pain, high-intensity pain and generalized pain. Each trajectory was estimated using a mixed-effects logistic regression model with natural cubic splines (3 degrees of freedom, selected via study-clustered five-fold cross-validation), fitted to individual-level data from 902 population-based sources nested within cohorts and world regions. Trajectories are marginal predictions standardized to a past-month recall period and general case definition. **a**, Any bodily pain prevalence. **b**, High-intensity pain, defined as NRS ≥ 7. **c**, Generalized pain, defined as pain in at least four of five body regions per the 2016 ACR fibromyalgia spatial criteria. In each panel, the upper plot shows model-estimated prevalence (%) across the lifespan (ages 5 to 100+), and the lower plot shows the corresponding age-specific rate of change (percentage-point change per year). Shaded bands indicate 95% CIs. Open triangles mark the age of peak rate of increase in prevalence; open circles mark the age of peak prevalence. Colors denote sex (women: orange; men: blue). Vertical dashed lines correspond to the marked ages. Sample sizes reflect the number of distinct individual-level records and are shown in panel titles. yr, year.

**Extended Data Fig. 2:**
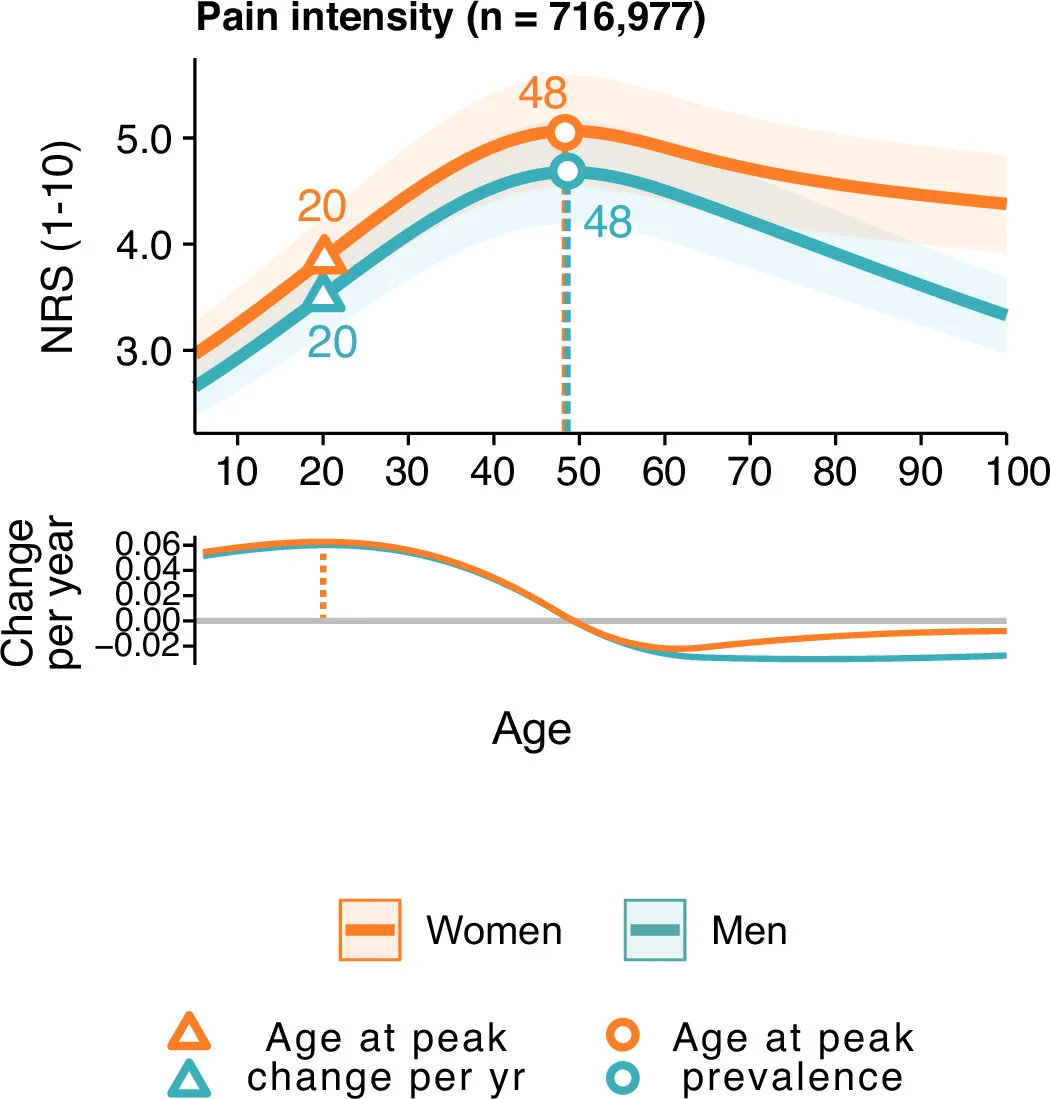
Average pain intensity across the lifespan by sex. Upper panel: model-estimated average pain intensity (numerical rating scale, 1–10) among individuals reporting pain (n = 716,977), by age and sex. Shaded bands indicate 95% confidence intervals. Open circles indicate the age at peak intensity; open triangles indicate the age of most rapid increase. Lower panel: rate of change in predicted intensity (NRS points per year), with the grey horizontal line indicating zero change.

A composite ‘any bodily pain’ outcome was coded as present if pain was reported at any site (Supplementary Information, section 3). Secondary outcomes included high-intensity pain (average intensity of 7 or higher on a 0−10 scale) and generalized pain (pain in at least four of five body regions following the 2016 fibromyalgia spatial criteria^8^), capturing dimensions of burden more closely related to disability and care seeking^6,7,9^. Here, the prevalence of any bodily pain increased progressively across the lifespan, from approximately 30% at age 5 years to 70% at age 100 years or older (Fig. 3). All three aggregate pain measures were consistently higher in women than in men: any bodily pain (RR = 1.16 (95% CI: 1.16−1.17)), high-intensity pain (RR = 1.52 (95% CI: 1.49−1.55)) and generalized pain (RR = 1.50 (95% CI: 1.46−1.54)). Whereas any bodily pain increased with age, the severity-based measures showed mid- to late-life peaks followed by declines, with high-intensity pain peaking around 50 years and generalized pain peaking later, around age 70 years. Average pain intensity among individuals reporting pain followed a similar nonlinear trajectory, peaking at approximately 5.0 in women and 4.6 in men around age 48 before declining through later life (Extended Data Fig. 2). Overall, our results suggest that, although the likelihood of experiencing any pain rises steadily with age, the most disabling forms of pain followed an inverted-U trajectory, peaking in mid-to-late adulthood before declining. This distinction, between pain accumulation and pain that intensifies and then resolves, has direct implications for where prevention and care should be concentrated across the lifespan.

### Regional variation and national development

**Fig. 4:**
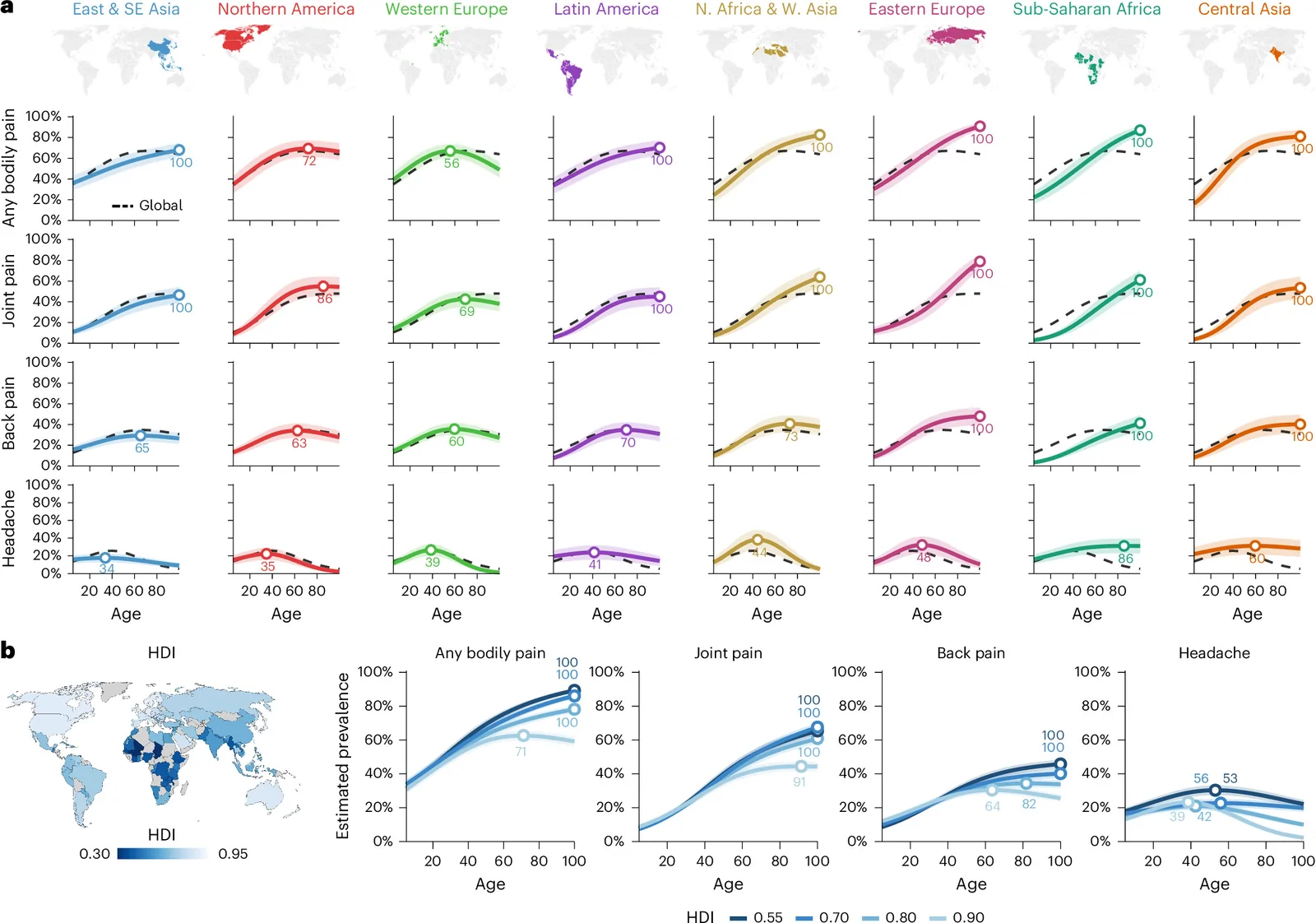
Regional variation and national development in lifespan pain trajectories. **a**, Model-estimated prevalence (%) by age for any bodily pain, joint pain, back pain and headache across eight world regions (Oceania excluded due to insufficient data), defined by the United Nations SDGs regional classification. Each column corresponds to one world region, with contributing countries highlighted on the inset world map above the column and each row to a pain phenotype. Solid colored lines show region-specific trajectories; open circles mark the age of peak prevalence (annotated with the corresponding age); and shaded bands denote 95% CIs. The dashed black line shows the global reference trajectory estimated from the full pooled dataset. **b**, Model-estimated prevalence trajectories for the same four pain outcomes as a continuous function of country-level HDI, plotted at four representative values (0.55, 0.70, 0.80 and 0.90) spanning low to very high development. The inset map shows country-level HDI values used in the analysis; countries without eligible data are shown in gray. Open circles mark the age of peak prevalence; shaded bands denote 95% CIs. In both panels, plotted lines denote the model-estimated marginal mean prevalence, and shaded bands denote 95% CIs.

Regional trajectories revealed a consistent pattern of early-life convergence and late-life divergence. For any bodily pain and joint pain, prevalence was broadly similar across regions in early adulthood but diverged substantially after age 65 years (Fig. 4a). Eastern Europe showed the greatest late-life excess, reaching approximately 21 percentage points above the global reference at age 80 or older, whereas western Europe remained below it throughout late adulthood. Sub-Saharan Africa, central and southern Asia and north Africa and west Asia showed comparatively low prevalence in early adulthood followed by steeper increases after midlife, ultimately converging with or exceeding the global reference in older age. Back pain followed a similar pattern, diverging across regions after approximately age 50 years, with continued increases into late life in eastern Europe, sub-Saharan Africa and central and southern Asia, and plateauing or declining trajectories in western Europe and northern America.

Headache showed the greatest regional heterogeneity, differing not only in magnitude but also in trajectory shape. Northern America and western Europe followed an early-adulthood peak and decline consistent with the global reference, whereas east and southeast Asia showed consistently lower and flatter prevalence across the lifespan. Eastern Europe and north Africa and west Asia showed later peaks with more gradual declines, and sub-Saharan Africa showed a modest monotonic increase from adolescence into later life. These contrasting patterns highlight how regional trajectories can differ substantially from the global reference when data from historically underrepresented regions are examined directly.

To examine whether regional differences partly reflected variation in national development, we estimated trajectories as a continuous function of country-level HDI (Fig. 4b). From age 40 years onward, the prevalence of any bodily pain, joint pain and back pain increased more steeply in countries with low HDI values, such that, by age 80 years or older, people in low-HDI settings experienced a substantially higher prevalence than in high-HDI settings (any bodily pain: risk difference: 30.6 percentage points (95% CI: 26.9−34.3); joint pain: 21.0 percentage points (95% CI: 20.2−21.8); back pain: 20.2 percentage points (95% CI: 19.6–20.9)). For low back pain specifically, prevalence in low-HDI settings approached twice that of high-HDI settings at the oldest ages. Headache prevalence was higher in lower-HDI settings throughout the lifespan. In high-HDI settings, headache prevalence declined after age 35 years, whereas this decline was attenuated in lower-HDI settings.

### Individual risk factors and socioeconomic gradients

**Fig. 5:**
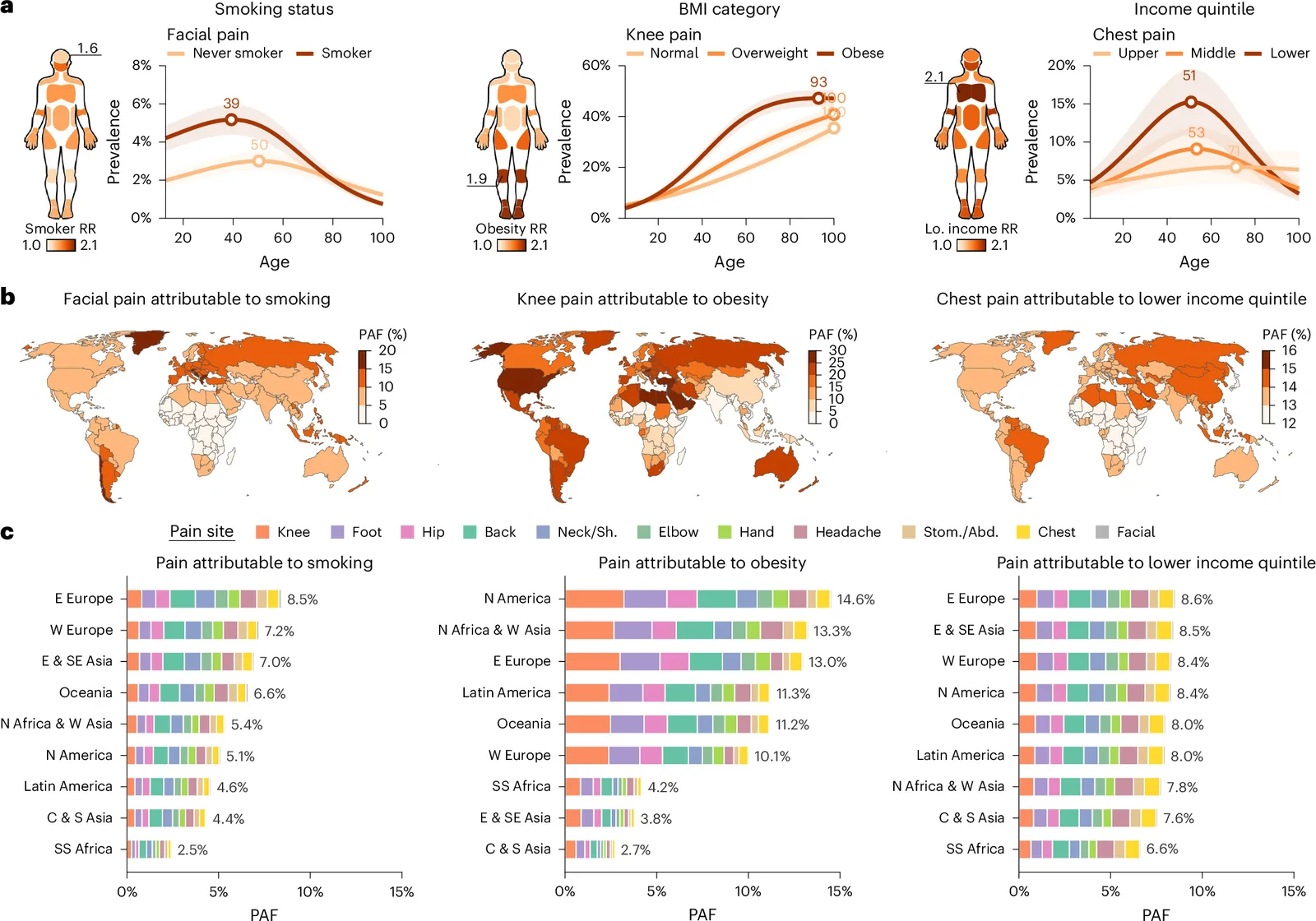
Risk factors, site-specific associations and PAFs for pain across the lifespan. **a**, Body maps display lifespan RRs for each anatomical site: current versus never smokers (left), people with obesity versus normal weight (center) and lowest versus highest household income quintile (right); color intensity indicates RR magnitude. Accompanying line plots show estimated marginal mean prevalence (%) across age with 95% CIs for a representative site per exposure: facial pain for smoking, knee pain for people with obesity (with overweight as an intermediate category) and chest pain for income (with the middle quintile for reference). Open circles and annotated ages mark the age of peak prevalence. **b**, Country-level PAFs for the same sentinel site−exposure pairs. PAF color scales differ across exposures to reflect variation in range. **c**, Total PAF across all pain sites by world region for each exposure. Stacked bars show the contribution of each anatomical site to the regional total. SS, sub-Saharan.

**Extended Data Fig. 3:**
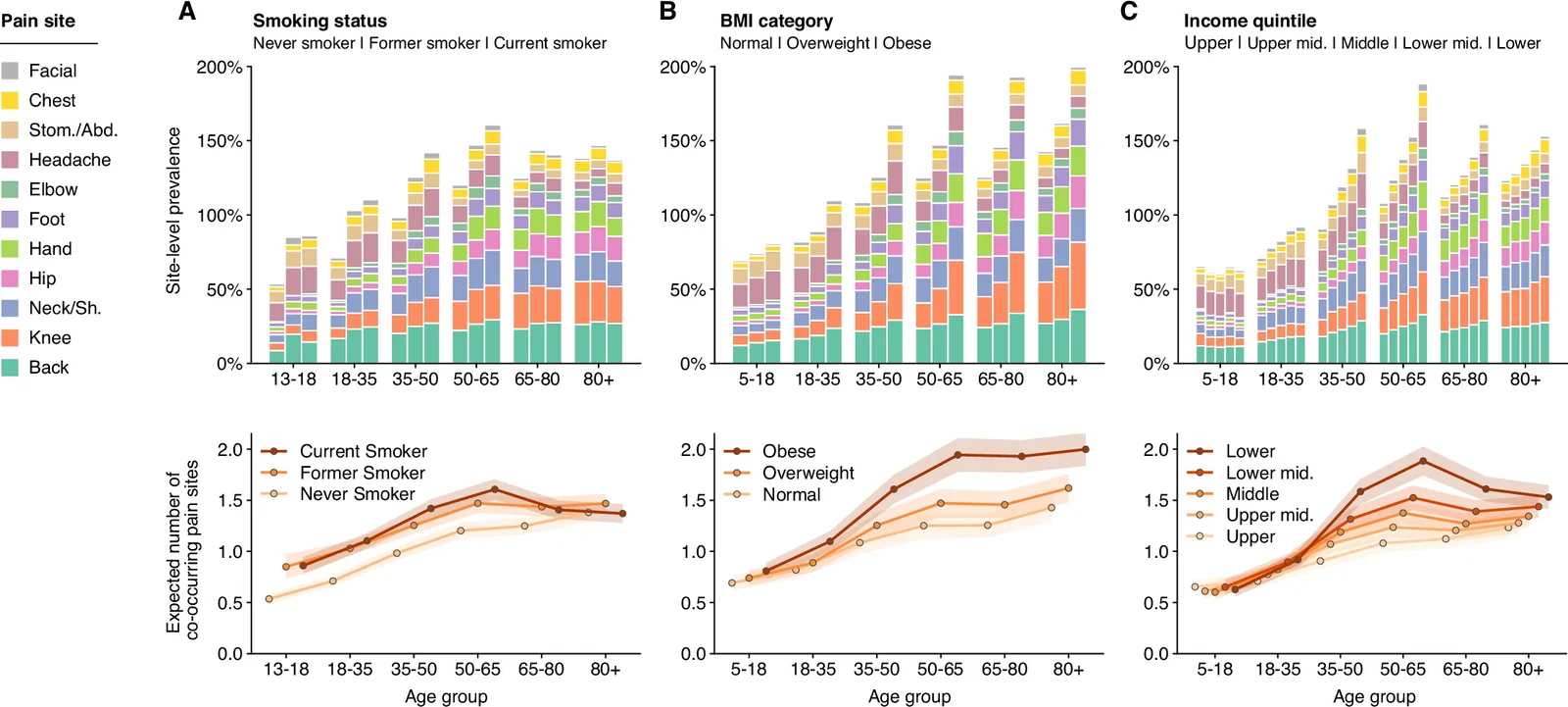
Pain burden across the lifespan by smoking, BMI, and household income. Upper-row stacked bars show age-group-specific, site-level prevalence (%) for each anatomical pain site, stratified by (**A**) smoking status (never smoker, former smoker, current smoker), (**B**) BMI category (normal weight, overweight, obese), and (**C**) household income quintile (upper, upper middle, middle, lower middle, lower). Colours denote pain sites. Because individuals can report pain at multiple sites, site-level prevalences are not mutually exclusive and stacked totals can exceed 100%. Lower-row line plots show the model-estimated marginal mean number of co-occurring pain sites for the same age groups and exposure categories, calculated by summing site-level prevalence across anatomical sites; shaded bands denote 95% confidence intervals. Smoking results are shown from ages 13 years and older.

**Extended Data Fig. 4:**
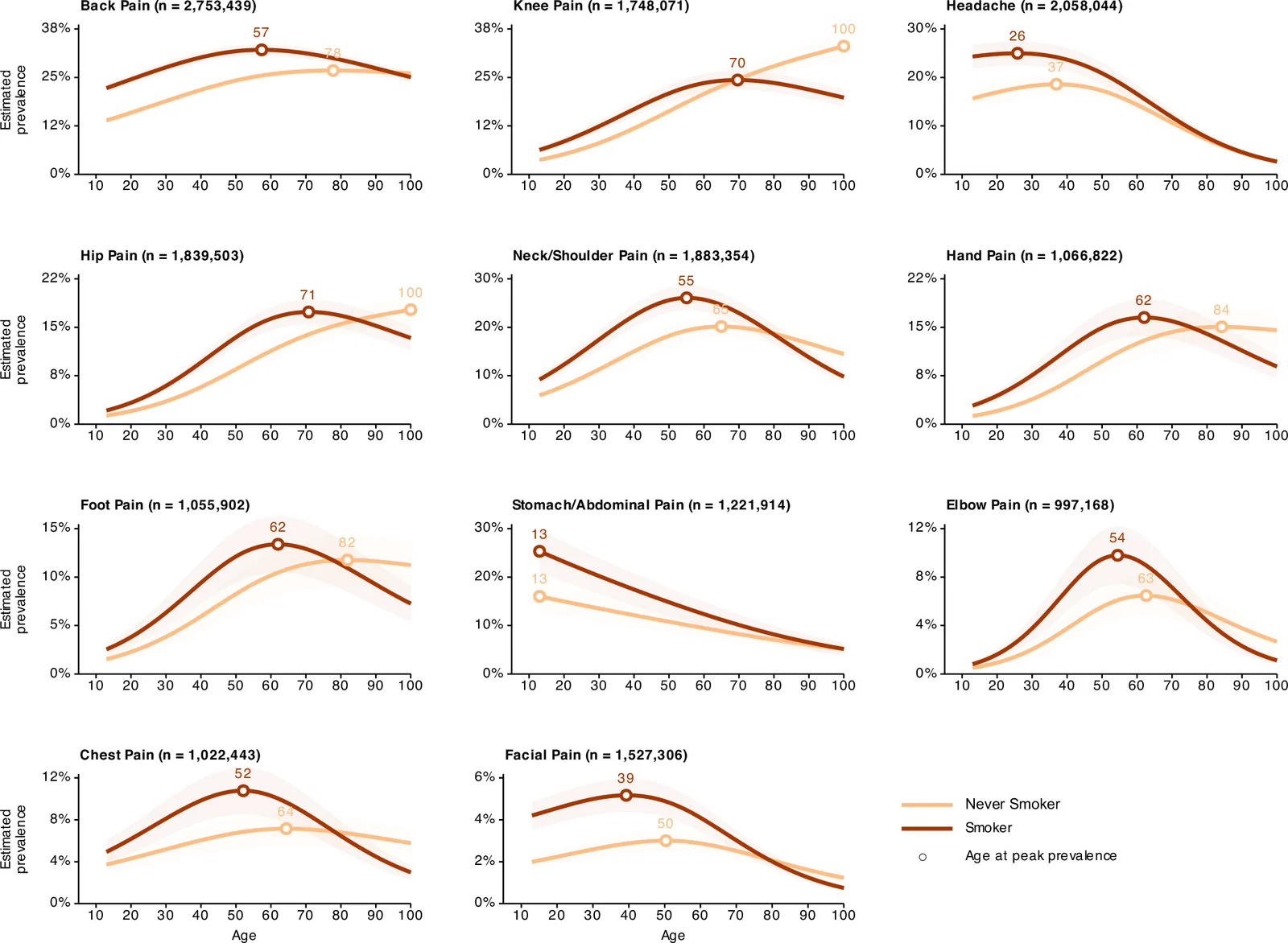
Prevalence trajectories by smoking status. Plotted lines denote the model-estimated marginal mean prevalence of 11 pain sites across the lifespan by smoking status (current smoker vs never smoker), and shaded bands denote 95% confidence intervals. Open circles indicate age at peak prevalence.

**Extended Data Fig. 5:**
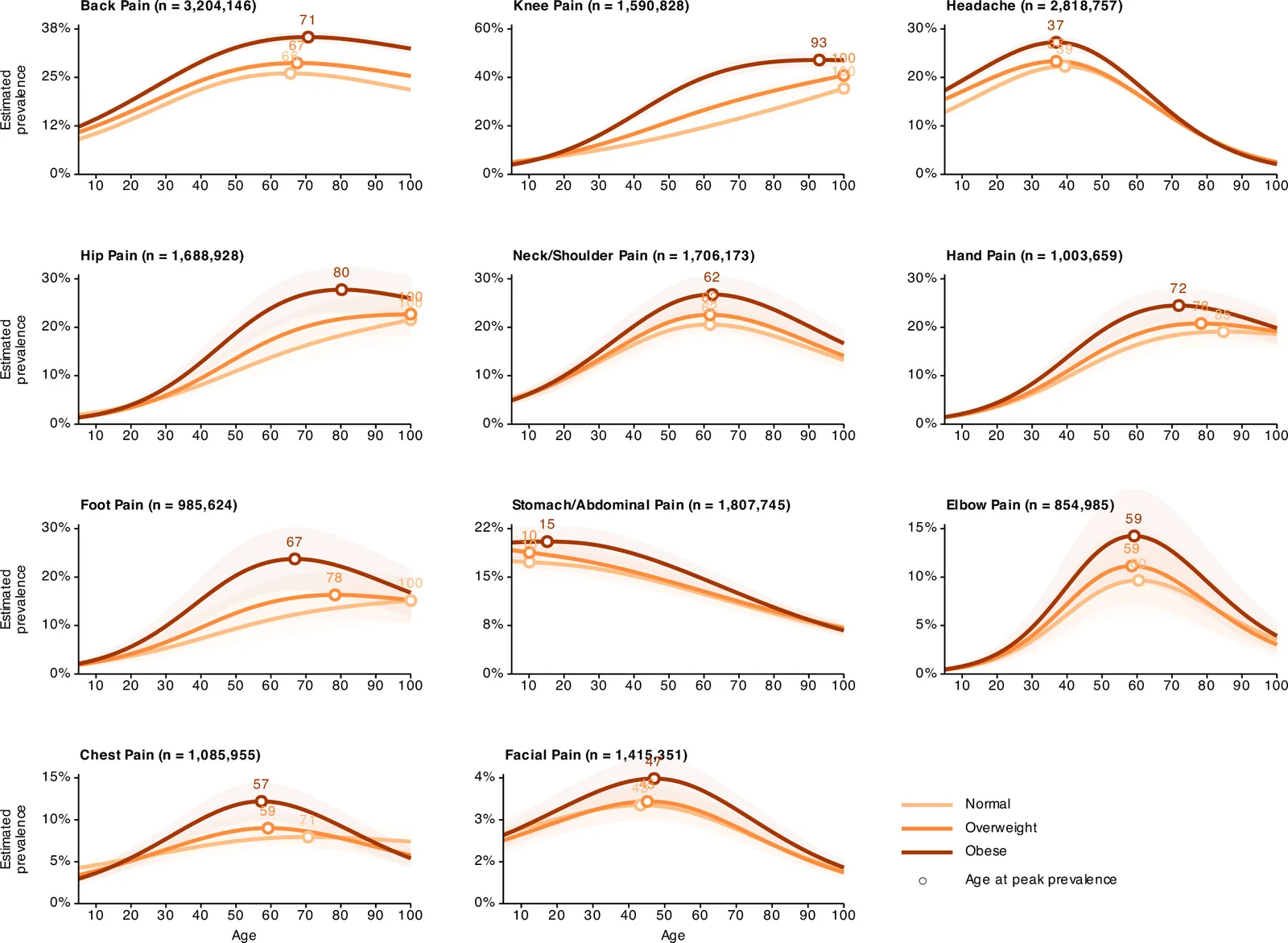
Prevalence trajectories by BMI category. Plotted lines denote the model-estimated marginal mean prevalence of 11 pain sites across the lifespan by BMI category (normal, overweight, obese), and shaded bands denote 95% confidence intervals. Open circles indicate age at peak prevalence.

**Extended Data Fig. 6:**
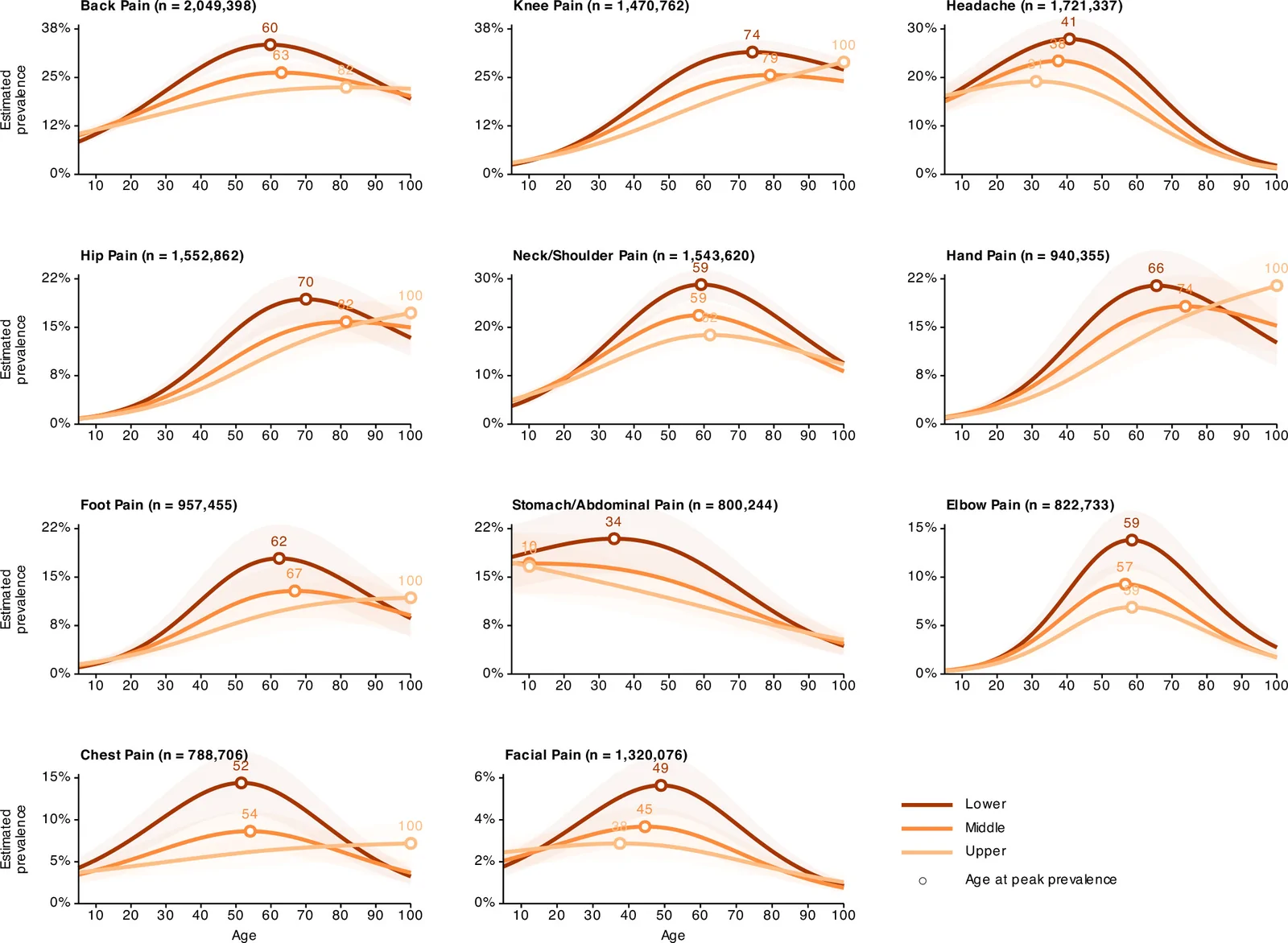
Prevalence trajectories by household income level. Estimated prevalence of 11 pain sites across the lifespan by three representative household income quintiles (lower, middle, upper). Income was harmonised as study-specific relative rank (equal-frequency quintiles), reflecting within-study socioeconomic position. Plotted lines denote the model-estimated marginal mean prevalence, and shaded bands denote 95% confidence intervals. Open circles indicate age at peak prevalence.

Smoking, obesity and low household income each showed distinct age-specific and anatomically patterned associations with pain prevalence (Fig. 5a and Extended Data Fig. 3). Current smoking was associated with a modest but consistent excess across pain sites from adolescence to age 65 years, with the largest effect observed for facial pain (RR = 1.67 (95% CI: 1.63−1.70) versus never smokers; Extended Data Fig. 4). Obesity showed a progressively diverging association from early adulthood onward, concentrated at weight-bearing joints: foot or ankle (RR = 1.91 (95% CI: 1.82−1.97) versus normal weight), knee (RR = 1.86 (95% CI: 1.82−1.90)) and hip (RR = 1.61 (95% CI: 1.57−1.66)), peaking in midlife (Extended Data Fig. 5). Household income level showed a pronounced dose−response pattern across all pain sites from early adulthood onward (Extended Data Fig. 6). This gradient was steepest for chest pain, with individuals in the lowest income level experiencing more than twice the risk compared to those in the highest (RR = 2.09 (95% CI: 2.01−2.17)), a disparity that peaked in midlife before converging in older age.

**Extended Data Fig. 7:**
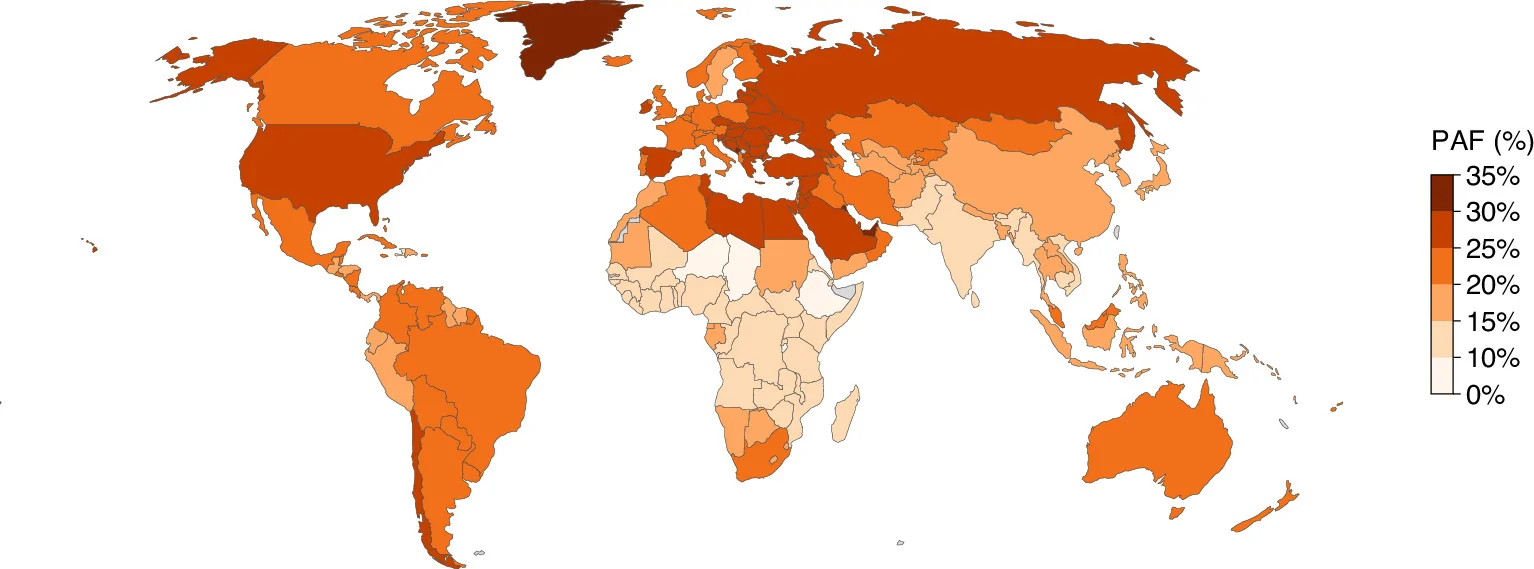
Combined population attributable fraction for pain by country. Country-level combined population attributable fraction (PAF, %) representing the proportion of site-specific pain burden jointly attributable to obesity, smoking, and low household income. Age- and sex-specific PAFs were computed at the country level by combining study-derived risk ratios with country-specific exposure prevalence from the Global Burden of Disease study (obesity: GBD 2021; smoking: GBD 2015) and population denominators. Income was operationalised as within-study relative rank (quintiles), with a fixed prevalence of 20% per quintile across all countries. Values are case-weighted averages across 11 anatomical pain sites. Grey indicates countries without available exposure data.

To quantify how much of the population-level pain burden these exposures collectively explain, we estimated population attributable fractions (PAFs) across regions (Fig. 5b,c). Obesity showed the widest regional variation, ranging from 2.7% in central and southern Asia to 14.6% in northern America, with high burden also concentrated in north Africa and west Asia and eastern Europe. Smoking attributable fractions ranged from 2.5% in sub-Saharan Africa to 8.5% in eastern Europe, and income attributable fractions were more consistent across regions (ranging from 6.6% to 8.6%). Globally, the combined attributable burden reached 27.1% in eastern Europe compared to 12.6% in sub-Saharan Africa (Extended Data Fig. 7 and Supplementary Table 7). In sub-Saharan Africa and central and southern Asia, obesity and smoking PAFs were each below 5%, indicating that the exposures responsible for the largest share of pain burden in high-HDI regions account for little of the burden in the lower-HDI regions, where the drivers of that burden remain poorly understood.

## Discussion

This study integrates individual-level self-reported pain data from over six million participants, spanning ages 5 to over 100 years, representing one of the largest harmonization efforts in pain epidemiology to date. Using raw participant-level observations rather than aggregated statistics or clinically defined conditions, we constructed global reference trajectories of pain across the lifespan, including age groups that remain sparsely represented in current surveillance. These curves provide a common framework against which individual cohorts, clinical populations and countries can be compared. Drawing on 902 population-based sources across 118 countries, these curves provide sufficient geographic coverage to directly test whether self-reported pain burden aligns with or diverges from condition-based global estimates.

The global lifespan trajectories show that pain is not a single epidemiological phenomenon but, rather, a set of distinct nonlinear patterns that differ in their timing and anatomical distribution. Across most phenotypes, pain did not increase monotonically with age; instead, the steepest rises occurred before age 55 years, indicating that a substantial share of lifetime pain burden develops during working-age adulthood. Trajectories varied markedly by phenotype: headache, abdominal and facial pain peaked earlier in life before declining, whereas musculoskeletal pain generally rose progressively with age. Within musculoskeletal sites, upper body pain (including neck, shoulder and elbow) peaked around midlife and declined thereafter, whereas lower body pain (including back, hip and knee) continued to increase across the lifespan, consistent with the greater role of cumulative mechanical and degenerative load at weight-bearing sites. A similar pattern was observed for severity-based measures, with both high-intensity and generalized pain peaking in midlife before declining. Because pain often occurs across multiple locations and may reflect broader overlapping pain phenotypes, we also examined generalized pain using the 2016 American College of Rheumatology (ACR) fibromyalgia spatial criteria and single-region versus multi-region pain among studies with sufficient anatomical coverage (Supplementary Fig. 15). These complementary analyses showed that co-occurring pain burden increases substantially across adulthood, reinforcing that site-specific trajectories should be interpreted alongside broader measures of multi-site and generalized pain. Overall, across most anatomical sites (seven of 11) and across severity-based measures, pain followed an inverted-U trajectory rather than a uniform age-related accumulation. These patterns may reflect true site-specific lifecourse processes, including earlier peaks in headache and abdominal pain and later accumulation of musculoskeletal pain, but cohort and reporting effects may also contribute. Older adults may appraise pain differently, experience pain across multiple regions rather than as a discrete site or be less likely to identify a single dominant location when pain is widespread. Still, the concentration of burden accumulation before midlife across most phenotypes suggests that the window for effective prevention may occur earlier than current clinical and policy frameworks typically assume.

Our study differs from the GBD framework in two key respects. First, GBD primarily estimates the prevalence of clinically defined conditions, such as osteoarthritis defined by clinical or radiographic criteria^10^ and migraine defined according to International Headache Society diagnostic criteria^11^. For neck pain and low back pain, GBD draws on heterogeneous sources, including population surveys, administrative health records using International Classification of Diseases codes and studies of activity-limiting pain^1,12^. The difference between self-reported and diagnosis-based estimates is particularly relevant as self-reported pain can be captured and compared in settings with limited access to care. A second difference is that GBD estimates are derived from aggregated summary statistics and modeled using Bayesian meta-regression to project estimates even where empirical data are sparse or absent^13,14^. Our analysis instead draws directly on individual-level observations from population-based surveys. As a result, GBD estimates for low back pain and knee osteoarthritis show a near-linear increase across adulthood until approximately 80 years of age followed by a decline. We instead showed nonlinear trajectories across the lifecourse, with the steepest increases occurring between puberty and midlife. Differences were also apparent across regions and levels of national development. Whereas GBD generally reports higher prevalence of low back pain, osteoarthritis and migraine in high-income regions, we instead observed higher prevalence of self-reported bodily, joint, back and headache pain in lower-HDI settings, particularly among older adults. Our findings for neck pain, however, were broadly consistent with GBD estimates, with prevalence peaking around age 60 years and lower levels in high-HDI settings. Overall, by estimating pain irrespective of clinical evaluations, using self-reported data, we provide a different view of burden that is informative in populations with limited health system access or data monitoring.

These global reference trajectories provide a benchmark against which historically underrepresented regions can be compared. The consistent pattern of early-life convergence and late-life divergence across regions indicates that regional disparities in pain burden emerge primarily after midlife. The greatest late-life excess was concentrated in eastern Europe and in the regions least represented in current surveillance (that is, sub-Saharan Africa, central and southern Asia and north Africa and west Asia) where trajectories rose steeply through midlife to exceed the global reference in older age. This regional patterning was not arbitrary but tracked the gradient of national development: the regions with the steepest late-life trajectories were those at the lower end of the HDI distribution, suggesting that the macro-level conditions HDI captures, including access to care, occupational exposures and comorbidity burden, may shape how pain accumulates across the lifespan. In addition, headache showed the greatest regional heterogeneity, characterized by divergence not only in magnitude but also in trajectory shape: the early-adulthood peak and decline characteristic of high-income regions was flatter in east and southeast Asia and absent in sub-Saharan Africa and central and southern Asia, where prevalence increased modestly across the lifespan, pointing to regionally distinct determinants that current data cannot fully resolve. Pain surveillance in children and adolescents remains sparse even in higher-development settings and is largely absent in lower-HDI regions, which may contribute to this heterogeneity and limits the ability to characterize early-life trajectories where they may diverge most. These are among the first population-based lifespan pain estimates available for these settings, and their divergence from the global reference underscores how heavily current global estimates are shaped by data from wealthier regions.

Modifiable risk factors accounted for a substantial but geographically uneven share of pain burden. In eastern Europe, northern America, western Europe and Latin America, the combined PAF of smoking, obesity and low income reached 22% to 27% of site-specific pain burden, identifying a meaningful preventable component concentrated in settings where these exposures are most prevalent. In sub-Saharan Africa and central and southern Asia, however, these same exposures accounted for less than 14% of burden, indicating that the drivers of pain in the world’s most affected populations are fundamentally different from those that dominate in high-income settings. The likely candidates include occupational physical load and employment conditions^15^, limited access to pain management and rehabilitation^16^ and a greater burden of infectious conditions and nutritional deficiencies^17,18^. Interventions effective in high-income settings are, therefore, unlikely to translate directly to lower-HDI contexts, where the drivers of pain burden appear fundamentally distinct and remain poorly characterized.

This work has important limitations. Most contributing datasets were cross-sectional, so the trajectories describe population age patterns rather than within-person change. Sampling designs also varied and were not uniformly nationally representative, although trajectories were materially unchanged in restricted-sample analyses except for stomach or abdominal pain, where the observed deviation coincided with a substantial reduction in sample size (Supplementary Information, section 6.7). Pain measures differed across studies, countries and languages, including current, past-week, past-month and 3−12-month recall periods; general versus chronic-explicit case definitions; direct site-specific questions versus body map coding; and different anatomical resolution, such as separate sites versus grouped regions including neck/shoulder, hand/wrist, foot/ankle or stomach/abdominal pain. We mapped these measures to prespecified anatomical categories and evaluated methodological covariates and recall-period restrictions in sensitivity analyses (Supplementary Table 3 and Supplementary Information, sections 3, 6.2 and 6.3), although residual differences in translation, symptom interpretation and response behavior may influence absolute prevalence estimates. Because responses capture current or recent symptoms, estimates should be interpreted as point or period prevalence rather than persistence or chronicity.

Coverage was uneven across regions and across the youngest and oldest ages, which widens uncertainty where data are sparse. Data coverage for non-musculoskeletal pain in mid- to low-HDI strata was insufficient to derive reliable trajectories for some sites, particularly chest, facial and abdominal pain. Socioeconomic and system indicators such as income and HDI are proxies and may not fully capture underlying contextual determinants, and survivorship at older ages and unmeasured clinical factors could also influence estimated levels and slopes. The broad 1990−2025 observation window increased geographic and age coverage but also combines historical and contemporary data collected under changing survey practices, care access, case definitions and symptom-reporting norms. Although primary models adjusted for survey year and this had minimal impact on the estimated trajectories, these curves should not be interpreted as secular trends in pain prevalence. Finally, the database may be affected by selection bias in which studies measured pain, made individual-level data available, received data-access approval or could be incorporated through investigator collaboration. Taken together, these constraints affect interpretation but do not alter the central lifecourse patterns seen across body sites.

Our findings establish that self-reported pain follows distinct lifespan trajectories that diverge markedly from condition-based global estimates, with the sharpest divergence in the populations most underrepresented in current surveillance. The reference curves and benchmarking infrastructure introduced here provide a foundation for more equitable global pain monitoring on which future surveillance and prevention efforts can build.

## Methods

### Ethics approval

The pooled analysis was approved by the McGill University Faculty of Medicine and Health Sciences Research Ethics Board (institutional review board study number A12-M69-23B; 23-10-068). The study involved secondary analysis of deidentified individual-level data obtained from public repositories and through data-transfer or data-use agreements with collaborating institutions. Contributing studies obtained informed consent from participants in accordance with their own ethics approvals; as a secondary analysis of existing deidentified data, the present study involved no additional participant consent or compensation.

### Data sources

We compiled individual participant data from population-based national, regional and international health studies and surveys conducted between 1990 and 2025 that assessed self-reported bodily pain (Supplementary Table 2). Data sources were identified and acquired between April 2024 and June 2026 through structured searches of bibliographic databases and public microdata repositories, review of national and international survey programs and direct contact with investigators, data custodians, cohort and biobank administrators and rheumatology networks. Publicly available datasets were downloaded directly, whereas restricted-access studies were incorporated through data-use agreements, formal data-access applications or standardized local analysis protocols when individual-level data transfer was not permitted.

Studies were eligible if they sampled community-dwelling participants without restricting participation based on health status, assessed pain by direct participant self-report and included either (1) a pain-presence item at one or more target anatomical sites or (2) an overall or average pain-intensity rating on a 0−10 numerical rating scale (NRS) or visual analogue scale (VAS), with 0 indicating no pain. Pain was assessed by direct participant self-report, except that parent-reported pain items were accepted for young children below the age of reliable self-report in a small number of pediatric cohorts (Supplementary Information, section 2.2 and Supplementary Table 2). Studies were excluded if they relied on clinician or observational reports or other proxy reports or did not measure any target pain outcome. The final analytic dataset included 6,125,459 individuals with valid data for at least one pain outcome, drawn from 138 study programs, across 118 countries and territories (Fig. 1). Study programs comprised 902 distinct data sources, reflecting multiple survey waves or country-specific samples within multi-country studies.

### Pain outcomes

The primary outcomes were binary indicators of self-reported pain at 11 anatomical sites: head, face, neck or shoulder, foot or ankle, hand or wrist, elbow, chest, back, stomach or abdomen, hip and knee. Reported pain at these anatomical sites was harmonized by mapping study-specific wording and body map coding schemes to standardized categories (Supplementary Information, section 3 and Supplementary Table 3). Where laterality was assessed, pain was coded as present if reported on either side. A composite ‘any bodily pain’ outcome was derived for studies with sufficient site coverage (Supplementary Information, section 3) and coded as present if pain was reported at any bodily site.

Secondary outcomes were as follows: (1) high-intensity pain, defined as average pain intensity of 7 or higher on a 0−10 NRS or VAS, and (2) generalized pain, defined as pain present in at least four of five body regions (left upper, right upper, left lower, right lower and axial), following the spatial criteria of the 2016 fibromyalgia revisions^8^. These secondary outcomes captured complementary dimensions of pain burden beyond site-specific prevalence^6,7,9^.

### Geographic and sociodemographic features

Countries were grouped into nine world regions using a modified United Nations Sustainable Development Goals (SDGs) regional classification (Supplementary Table 4)^19^. National development was indexed using the HDI, obtained from United Nations Development Programme reports and matched to each participantʼs country and survey year^20^. Sex was self-reported within each contributing study.

### Individual risk factors

We evaluated a prespecified set of three modifiable or policy-relevant exposures with established associations with pain outcomes across the literature: body mass index (BMI), smoking status and household income. These exposures were selected based on prior empirical support, alignment with GBD risk factor frameworks^1^ and sufficient cross-study harmonizability to support pooled estimation^21–23^. Together, they capture metabolic, behavioral and socioeconomic dimensions of pain risk. Other social and occupational determinants, including employment status, type of work, housing security and food insecurity, were not consistently available or harmonizable across the contributing datasets and require dedicated study.

Smoking status was categorized as current (regular or occasional), former or never. BMI was obtained directly from study-provided values when available or otherwise derived from measured or self-reported height and weight. Adults were classified as having normal weight (18.5−24.9 kg m^−^^2^), overweight (25.0−29.9 kg m^−^^2^) or obesity (30.0 kg m^−^^2^ or higher). For participants aged 19 years or younger, BMI categories were assigned using age-specific and sex-specific cutoffs based on pediatric growth standards from the World Health Organization^24^. Household income was harmonized within each study by ranking participants into study-specific income quintiles (equal-frequency groups), reflecting relative socioeconomic position within studies, consistent with established inequality monitoring practice^25–27^.

### Harmonization procedure

Pain prevalence estimates vary with methodological factors such as interview setting, question phrasing, recall period and study design characteristics^28–30^. To address this, our harmonization procedure systematically evaluated seven key methodological features for their association with reported pain: recall period, case definition (general versus chronic-explicit), survey format, survey setting, question valence, question format and sampling strategy (Methods and Supplementary Information, section 6.2). Recall period showed the most consistent and substantial association with reported pain and was included in all models (short: past week; medium: past month; long: past 3−12 months). We additionally coded case definition as general versus chronic-explicit, where chronic-explicit indicated that the question specified pain lasting longer than 3 months; this covariate was included to adjust for the small subset of studies using chronic-explicit wording. The remaining five features (that is, survey format, survey setting, question valence, question format and sampling strategy) had small or site-specific effects (±2 percentage points) that did not alter age-by-site patterns and were not retained to preserve parsimony. Sensitivity analyses on the stability of global trajectories across studies (Supplementary Information, section 6.1), methodological covariates (Supplementary Information, section 6.2) and recall periods (Supplementary Information, section 6.3), random-effects structures (Supplementary Information, section 6.4), functional form (Supplementary Information, section 6.5), data missingness (Supplementary Information, section 6.6) and sampling design (Supplementary Information, section 6.7) support the robustness of our harmonization procedure.

### Statistical analysis

We estimated age-specific prevalence trajectories for each pain outcome across the lifespan (ages 5 to 100 or older; ages below 5 and above 100 were bottom-coded and top-coded) using GLMMs with a logit link. Age was modeled using natural cubic splines to flexibly capture nonlinear trajectories. The spline degrees of freedom were selected using study-clustered five-fold cross-validation; 3 degrees of freedom were retained based on out-of-sample fit (Supplementary Information, section 6.5). We report 95% CIs throughout.

Primary models included an interaction between age and the exposure of interest, namely sex (Figs. 2 and 3), world region or HDI (Fig. 4) and BMI category, smoking status or income quintile (Fig. 5 and Extended Data Figs. 3–6), and were adjusted for methodological differences using fixed effects for recall period and case definition and, except for the HDI models, survey year (centered on the sample mean). Survey year was omitted from the HDI models because HDI is assigned by country year and showed limited overlap with survey year across the pooled dataset, making joint adjustment prone to extrapolation. All models additionally adjusted for sex, except those in which sex was the primary exposure. Random intercepts for study program and world region were included to account for clustering and enable partial pooling across heterogeneous data sources. In regional analyses, region was instead treated as a fixed effect with a study nested within region random intercept, preventing any single multi-country study from dominating region-specific estimates. Sensitivity analyses for missing risk factor data are reported in Supplementary Information, section 6.6.

Adjusted prevalence trajectories were obtained as marginal predictions and standardized to a past-month recall period and the general case definition and the mean survey year (Supplementary Information, section 5). HDI trajectories were plotted at four representative values (0.55, 0.70, 0.80 and 0.90) spanning low to very high development. We also quantified the rate of age-related change as the year-to-year difference in predicted prevalence (percentage-point change per year). We used these age-specific rates of change to classify trajectory shape. A site was described as showing an inverted-U pattern only when the fitted trajectory showed both an early-life increase and a later-life decline, defined by 95% CIs excluding zero for the rate of change in both phases. Relative risks and their 95% CIs were computed for all sex, exposure and HDI contrasts across pain sites and age bands, and a Benjamini−Hochberg false discovery rate correction was applied across this full family of comparisons to account for multiple testing (Supplementary Table 6). Complete numerical outputs and supporting documentation are provided in Supplementary Data 1–5, comprising relative risks, model coefficients, site-level exposure prevalences, predicted prevalence trajectories and pain question wording, respectively. All analyses were performed in R (version 4.4.1) and Python (version 3.8.13).

### Ethics and inclusion statement

This study synthesized individual-level data from population-based surveys, cohort studies and national health studies conducted across multiple world regions. Data were obtained through publicly available repositories or through data-transfer and data-use agreements with collaborating researchers and institutions. The collaboration included investigators from diverse geographic regions who contributed expertise in epidemiology, clinical pain research, population health and lived experiences of pain.

### Reporting summary

Further information on research design is available in the Nature Portfolio Reporting Summary linked to this article.

## Online content

Any methods, additional references, Nature Portfolio reporting summaries, source data, extended data, supplementary information, acknowledgements, peer review information; details of author contributions and competing interests; and statements of data and code availability are available at https://doi.org/10.1038/s41591-026-04696-w.

## Supplementary information

Supplementary Information Supplementary Sections 1−10, Supplementary Figs. 1−15, Supplementary Tables 1−7 and Supplementary Note: Acknowledgements and funding for contributing studies.

Reporting Summary

Peer Review File

Supplementary Data 1−5 Combined workbook containing age-specific and overall RRs; complete model coefficients; exposure prevalence estimates used in PAF analyses; predicted pain prevalence and intensity trajectories by sex, world region and HDI, with 95% CI; and pain questions by study and country.

## Data Availability

An interactive benchmarking tool and downloadable summary statistics from this study are publicly available at https://evppainlab.shinyapps.io/global-pain-benchmark/. Researchers seeking access to the original individual-level data should apply directly to the respective data custodians according to their access policies. The authors cannot provide these data directly but will respond to inquiries about data availability and access within 2 weeks.

## Code availability

Detailed and annotated analysis code is publicly available via GitHub (https://github.com/EVPlab/lifespan-pain-trajectories).
