# Supplementary material for: Global and regional reference curves for pain across the lifespan in 6.1 million individuals in 118 countries
Source: Nat Med. 2026 Oct 5;32(11):4298–306. doi: 10.1038/s41591-026-04696-w (PMC13768165; doi:10.1038/s41591-026-04696-w)
Supplement: Supplementary file 1 [file 41591_2026_4696_MOESM1_ESM.pdf]

nature medicine

Supplementary information

https://doi.org/10.1038/s41591-026-04696-w

Global and regional reference curves for pain across the lifespan in 6.1 million individuals in 118 countries

In the format provided by the authors and unedited

Table of Contents

Section 1. Global Pain Assessment Database ***3***

Section 2. Data sources ***3***

Supplementary Table 1. Demographic characteristics of the analytic sample, by world region. 4
Supplementary Table 2: Characteristics of included data sources 5

Section 3. Variable definitions, harmonization, and derived outcomes ***35***

Supplementary Table 3: Harmonization definitions and inclusion criteria for anatomical pain sites 38
Supplementary Table 4: Classification of countries and territories by world region 42

Section 4. Data processing and quality control ***46***

Section 5. Statistical methods ***46***

Section 6. Sensitivity analyses ***50***

Section 6.1 Leave-One-Study-Out Sensitivity Analysis **50**

Supplementary Figure 1: Stability of global trajectories in leave-one-study-out sensitivity analysis 52
Supplementary Figure 2: Matrix of individual study influence on prevalence estimates 53

Section 6.2. Sensitivity Analysis of Methodological Covariates **55**

Supplementary Figure 3: Sensitivity of prevalence estimates to methodological covariates 56
Supplementary Figure 4: Trajectory robustness under maximum methodological adjustment 57
Supplementary Figure 5: Directionality of methodological effects on reported prevalence 58

Section 6.3. Sensitivity Analysis of Data Exclusion based on Recall Period **59**

Supplementary Figure 6: Deviation of restricted data subsets from the primary model 60
Supplementary Figure 7: Concordance between primary model and restricted data subsets 61

Section 6.4. Sensitivity Analysis of Random-Effects Structure **62**

Supplementary Figure 8: Magnitude of deviation between random-intercept and random-slope models 63
Supplementary Figure 9: Sensitivity of trajectories to random-effects structure 64

Section 6.5. Sensitivity Analysis of Functional Form (Age Spline Complexity) **65**

Supplementary Figure 10: Model selection statistics for functional form 66
Supplementary Figure 11: Sensitivity of age trajectories to spline complexity 67
Supplementary Table 5: Model selection metrics for age-trajectory functional form 68

Section 6.6. Sensitivity Analysis of Missing Risk Factor Data **70**

Supplementary Figure 12: Sensitivity of relative risk estimates to missing data imputation 71

Section 6.7. Sensitivity Analysis of Sampling Design **72**

Supplementary Figure 13: Sensitivity of age-by-site trajectories to sampling design 73

Section 6.8. Sensitivity analysis: HDI–survey-year common support **74**

Supplementary Figure 14: HDI sensitivity analysis under common support for survey year. 75

Section 7. Individual-level risk factor gradients of pain across the lifespan ***76***

Supplementary Table 6. Risk ratios for sex and health exposures across pain sites. 77

Section 8. Population attributable fraction by region and country ***79***

Supplementary Table 7: Population attributable fractions for pain by exposure and world region 80

Section 9. Average pain intensity across the lifespan ***81***

Section 10. Global benchmarking framework and interactive application ***82***

Supplementary Figure 15: Single-region versus multi-region pain across the lifespan 84

Supplementary Note. Acknowledgements and funding for contributing studies *87*

Section 1. Global Pain Assessment Database

Collaboration overview

The present study was made possible by an international collaboration of investigators who contributed individual participant data from population-based surveys and cohort studies. The collaboration was established to map reference curves of pain across the human lifespan and to quantify how these trajectories differ by sociodemographic (sex and total household income), health features (smoking status and BMI), and broader environment (world region and country-level Human Development Index).

Section 2. Data sources

2.1 Data access

We compiled individual level data from a wide range of population based national, regional, and international health studies and surveys conducted between 1990 and 2025 that assessed self-reported bodily pain. The analytic sample comprised 6,125,459 participants across 902 data sources in 118 countries, with demographic characteristics by world region summarised in Supplementary Table 1. Major sources included surveys coordinated by the World Health Organization, the Community Oriented Program for Control of Rheumatic Diseases (COPCORD), the National Health Interview Surveys (NHIS), and the United Kingdom Biobank (UKB), alongside additional national and regional population-based studies. A full list of data sources is provided in Supplementary Table 2.

Supplementary Table 1. Demographic characteristics of the analytic sample, by world region.

Region / population	Data sources (n)	Countries (n)	N (analytic sample)	Female (%)	Age, years (median, IQR)	Years of data collection (median, range)
Overall (all regions)	902	118	6,125,459	54.7	44 (24–60)	2010 (1991–2025)
Western Europe	387	20	1,925,815	53.4	48 (16–62)	2008 (1991–2023)
Northern America	80	3	1,540,297	58.0	48 (32–64)	2015 (1994–2025)
East and southeast Asia	60	14	1,047,036	51.8	43 (32–57)	2003 (1996–2025)
Eastern Europe	222	23	515,073	52.8	15 (13–34)	2010 (2000–2022)
Latin America	48	15	502,658	57.6	44 (30–60)	2012 (1999–2022)
Central and southern Asia	20	5	196,518	55.1	45 (28–58)	2017 (1996–2024)
Sub-Saharan Africa	44	21	189,862	55.6	35 (23–55)	2007 (2000–2022)
North Africa and west Asia	35	13	164,545	54.4	27 (16–43)	2008 (2000–2022)
Oceania	6	4	43,655	52.0	30 (16–52)	2019 (1992–2021)

N (analytic sample) denotes participants with at least one recorded pain site together with valid age and sex. Age is summarised as the median and interquartile range (IQR); years of data collection as the median survey year and full range. Regions follow the United Nations Sustainable Development Goals classification and are ordered by sample size. Percentages are of participants within each region.

Supplementary Table 2: Characteristics of included data sources

No.	Study name	Country	Year of data collection	N (analytic sample)	Age range (years)	Survey type	Pain sites collected	Risk factors analyzed
1	Adolescent Brain Cognitive Development Study (ABCD)	United States	2017	10843	11–14	Cohort	PI, P, Ha, N/S, C, S/A, B, K, H, F, Hnd, Ft, E	BMI; Income
2	Agincourt Integrated Family Survey (AIFS)	South Africa	2004	1507	18–92	Surveillance/HDSS	P, Ha, C, S/A, B	BMI; Income
3	Alameda County Health and Ways of Living Study (ACHWLS)	United States	1994	2729	46–100	Panel	Ha, C, S/A, B	Smoking; Income
4	All of Us Research Program (AoU)	United States	2018	520906	18–87	Cohort	PI, P	BMI; Smoking; Income
5	Australian Longitudinal Study on Aging (ALSA)	Australia	1992	2080	65–100	Cohort	P, Ha, C, B, K, H, Hnd, Ft, E	Smoking; Income
6	Austrian Health Information Survey (ATHIS)	Austria	2006	15474	17–87	Household survey	PI, P, Ha, N/S, C, S/A, B, K, H, F, Hnd, Ft	BMI; Smoking; Income
7	Bangladesh Urban Health Survey (BUHS)	Bangladesh	2006	28010	13–59	Household survey	Ha	BMI; Income
8	Brazil National Survey of Health (PNS)	Brazil	2013	60202	18–100	Household survey	B	BMI; Income
9	Brazil National Survey of Health (PNS)	Brazil	2019	90846	15–100	Household survey	B	BMI; Income
10	Brazilian Children's Musculoskeletal Pain Survey (BCS)	Brazil	2020	1988	6–18	School-based survey	PI, P, N/S, B	BMI
11	Brazilian Longitudinal Study of Adult Health	Brazil	2021	12409	41–84	Cohort	PI, P, N/S, B, K, H, Hnd, Ft, E	BMI; Smoking; Income
12	Brazilian Longitudinal Study of Aging (ELSI)	Brazil	2019	9949	50–100	Household survey	P, Ha, N/S, F	BMI; Smoking
13	Brazilian Pain Survey	Brazil	2015	723	16–75	Household survey	PI, P, Ha, N/S, C, S/A, B, K, H, F, Hnd, Ft	
14	COPCORD	Argentina	2011	644	18–85	Community survey	P, N/S, B, K, H, Hnd, Ft, E	
15	COPCORD	Colombia	2014	6690	15–99	Community survey	PI, P, N/S, B, K, H, Hnd, Ft, E	Smoking; Income
16	COPCORD	Democratic Republic of the Congo	2012	1315	7–88	Community survey	P, N/S, B, K, H, Hnd, Ft, E	BMI
17	COPCORD	Ecuador	2014	4867	18–97	Community survey	PI, P, N/S, B, K, H, Hnd, Ft, E	Income
18	COPCORD	Ecuador	2016	2681	18–97	Community survey	P, N/S, B, K, H, Hnd, Ft, E	Income
19	COPCORD	Indonesia	2023	3898	16–100	Community survey	PI, P, N/S, B, K, Hnd, Ft, E	BMI; Smoking
20	COPCORD	Iran	2004	10288	15–100	Community survey	P, N/S, B, K, H, Hnd, Ft, E	BMI
21	COPCORD	Iran	2008	2099	15–84	Community survey	P, N/S, B, K, H, Hnd, Ft, E	BMI
22	COPCORD	Iran	2011	2654	15–100	Community survey	P, N/S, B, K, H, Hnd, Ft, E	
23	COPCORD	Iran	2014	2000	15–90	Community survey	P, N/S, B, K, H, Hnd, Ft, E	BMI
24	COPCORD	Lebanon	2009	3517	15–90	Community survey	PI, P, N/S, B, K, H, Hnd, Ft	BMI; Smoking; Income
25	COPCORD	Mexico	2012	23181	17–100	Community survey	PI, P, N/S, B, K, H, Hnd, Ft	Income
26	COPCORD	Nepal	2021	2143	18–95	Community survey	P, N/S, B, K, H, Hnd, Ft, E	Smoking; Income
27	COPCORD	Pakistan	2018	5495	12–99	Community survey	P, N/S, B, K, Hnd, Ft	Smoking; Income
28	COPCORD	Pakistan	2024	7336	18–100	Community survey	P, N/S, B, K, H, Hnd, Ft, E	

29	COPCORD	Peru	2010	3036	17–100	Community survey	PI, P, N/S, B, K, H, Hnd, Ft, E	BMI; Income
30	COPCORD	Qatar	2019	1225	15–96	Community survey	PI, P, N/S, B, K, H, Hnd, Ft	BMI
31	COPCORD	Venezuela	2011	3974	17–100	Community survey	P, N/S, B, K, Hnd, Ft, E	
32	Cape Area Panel Study (CAPS)	South Africa	2006	2248	50–97	Panel	P, Ha, C, S/A, B	BMI; Income
33	Childhood to Adolescence Transition Study (CATS)	Australia	2012	1191	8–11	Cohort	P, Ha, N/S, C, S/A, B, K, H, Hnd, Ft, E	BMI; Income
34	Chilean Gender and Sexuality Survey (ENSSEX)	Chile	2022	20374	18–100	Household survey	P, Ha	Income
35	Chilean Health Survey (ENS)	Chile	2003	3557	16–99	Household survey	PI, P, N/S, B, K, H, Hnd, Ft, E	BMI; Smoking; Income
36	Chilean Health Survey (ENS)	Chile	2009	5276	15–100	Household survey	PI, P, N/S, C, B, K, H, Hnd, Ft, E	BMI; Smoking; Income
37	Chilean Health Survey (ENS)	Chile	2016	6233	15–98	Household survey	PI, P, N/S, B, K, H, F, Hnd, Ft, E	BMI; Smoking; Income
38	Chilean Workers Survey (ENETS)	Chile	2009	9501	15–99	Household survey	P, Ha, N/S, B, K, H, Hnd, Ft, E	Income
39	China Health and Retirement Longitudinal Study (CHARLS)	China	2011	24353	21–100	Panel	PI, P, Ha, N/S, C, S/A, B, K, H, Hnd, Ft	BMI; Smoking; Income
40	Chongqing Pain Survey	China	2012	992	15–90	Household survey	P, Ha, N/S, C, S/A, B, F	
41	Chronic Headache in Adolescents (CHAP)	Germany	2017	2280	10–18	Cohort	PI, P, Ha, C, S/A, B	BMI; Income
42	CoLaus	Switzerland	2015	3590	45–87	Cohort	PI, P, N/S, B, K, H, F, Hnd, Ft, E	Smoking; Income
43	Collaborative Psychiatric Epidemiology Surveys (CPES)	United States	2001	4645	18–97	Cross-section	P, Ha, C, S/A, B	BMI; Smoking; Income
44	Community-Led Total Sanitation and Hygiene in Ethiopia (CLTSH)	Ethiopia	2017	4726	5–80	Program/Household survey	Ha	
45	Constances[1]	France	2016	29728	24–80	Cohort	PI, P, N/S, B, K, Hnd, E	BMI; Smoking
46	Cross Marketing Survey (CMS)	Japan	2022	1946	20–79	Community survey	P, N/S, B, K, H, Hnd, Ft, E	
47	DOLORisk	United Kingdom	2016	7236	24–99	Cohort	PI, P, Ha, N/S, C, S/A, B, K, H, F, Hnd, Ft	
48	English Longitudinal Study on Aging (ELSA)	United Kingdom	2006	2777	19–99	Panel	P, C, B, K, H, F, Ft	Smoking; Income
49	English Longitudinal Study on Aging (ELSA)	United Kingdom	2008	3277	24–99	Panel	P, C, B, K, H, F, Ft	BMI; Smoking; Income
50	English Longitudinal Study on Aging (ELSA)	United Kingdom	2010	301	35–88	Panel	P, C, B, K, H, F, Ft	Smoking; Income
51	English Longitudinal Study on Aging (ELSA)	United Kingdom	2012	8119	31–89	Panel	P, B, K, H, F, Ft	BMI; Smoking; Income
52	English Longitudinal Study on Aging (ELSA)	United Kingdom	2014	524	29–88	Panel	P, B, K, H, F, Ft	Smoking; Income
53	English Longitudinal Study on Aging (ELSA)	United Kingdom	2016	40	37–79	Panel	P, B, K, H, F, Ft	Smoking; Income
54	English Longitudinal Study on Aging (ELSA)	United Kingdom	2018	1156	33–76	Panel	P, B, K, H, F, Ft	Smoking; Income
55	English Longitudinal Study on Aging (ELSA)	United Kingdom	2020	1872	26–88	Panel	P, B, K, H, F, Ft	Smoking; Income
56	Epidemiology of Neuropathic Pain in Canada (ENPC)	Canada	2017	1483	5–93	Cross-section	PI, P, Ha, N/S, C, B, K, H, Hnd, Ft, E	Smoking; Income
57	Eurobarometer	Austria	2006	1008	15–92	Repeat cross-section	P, Ha, N/S, B, K, H, Hnd, Ft, E	Smoking
58	Eurobarometer	Belgium	2006	1010	15–93	Repeat cross-section	P, Ha, N/S, B, K, H, Hnd, Ft, E	Smoking
59	Eurobarometer	Bulgaria	2006	1016	15–91	Repeat cross-section	P, Ha, N/S, B, K, H, Hnd, Ft, E	Smoking

60	Eurobarometer	Croatia	2006	998	15–89	Repeat cross-section	P, Ha, N/S, B, K, H, Hnd, Ft, E	Smoking
61	Eurobarometer	Cyprus	2006	1006	15–96	Repeat cross-section	P, Ha, N/S, B, K, H, Hnd, Ft, E	Smoking
62	Eurobarometer	Czech Republic	2006	1047	15–87	Repeat cross-section	P, Ha, N/S, B, K, H, Hnd, Ft, E	Smoking
63	Eurobarometer	Denmark	2006	1060	15–93	Repeat cross-section	P, Ha, N/S, B, K, H, Hnd, Ft, E	Smoking
64	Eurobarometer	Estonia	2006	989	15–94	Repeat cross-section	P, Ha, N/S, B, K, H, Hnd, Ft, E	Smoking
65	Eurobarometer	Finland	2006	1023	15–95	Repeat cross-section	P, Ha, N/S, B, K, H, Hnd, Ft, E	Smoking
66	Eurobarometer	France	2006	1014	15–95	Repeat cross-section	P, Ha, N/S, B, K, H, Hnd, Ft, E	Smoking
67	Eurobarometer	Germany	2006	1537	15–94	Repeat cross-section	P, Ha, N/S, B, K, H, Hnd, Ft, E	Smoking
68	Eurobarometer	Greece	2006	999	15–91	Repeat cross-section	P, Ha, N/S, B, K, H, Hnd, Ft, E	Smoking
69	Eurobarometer	Hungary	2006	993	15–92	Repeat cross-section	P, Ha, N/S, B, K, H, Hnd, Ft, E	Smoking
70	Eurobarometer	Ireland	2006	1294	15–95	Repeat cross-section	P, Ha, N/S, B, K, H, Hnd, Ft, E	Smoking
71	Eurobarometer	Italy	2006	999	15–84	Repeat cross-section	P, Ha, N/S, B, K, H, Hnd, Ft, E	Smoking
72	Eurobarometer	Latvia	2006	986	15–74	Repeat cross-section	P, Ha, N/S, B, K, H, Hnd, Ft, E	Smoking
73	Eurobarometer	Lithuania	2006	992	15–95	Repeat cross-section	P, Ha, N/S, B, K, H, Hnd, Ft, E	Smoking
74	Eurobarometer	Luxembourg	2006	497	15–90	Repeat cross-section	P, Ha, N/S, B, K, H, Hnd, Ft, E	Smoking
75	Eurobarometer	Malta	2006	499	15–89	Repeat cross-section	P, Ha, N/S, B, K, H, Hnd, Ft, E	Smoking
76	Eurobarometer	Netherlands	2006	1063	15–92	Repeat cross-section	P, Ha, N/S, B, K, H, Hnd, Ft, E	Smoking
77	Eurobarometer	Poland	2006	976	15–91	Repeat cross-section	P, Ha, N/S, B, K, H, Hnd, Ft, E	Smoking
78	Eurobarometer	Portugal	2006	991	15–98	Repeat cross-section	P, Ha, N/S, B, K, H, Hnd, Ft, E	Smoking
79	Eurobarometer	Romania	2006	1016	15–91	Repeat cross-section	P, Ha, N/S, B, K, H, Hnd, Ft, E	Smoking
80	Eurobarometer	Slovakia	2006	1167	15–91	Repeat cross-section	P, Ha, N/S, B, K, H, Hnd, Ft, E	Smoking
81	Eurobarometer	Slovenia	2006	1036	15–91	Repeat cross-section	P, Ha, N/S, B, K, H, Hnd, Ft, E	Smoking
82	Eurobarometer	Spain	2006	1022	15–94	Repeat cross-section	P, Ha, N/S, B, K, H, Hnd, Ft, E	Smoking
83	Eurobarometer	Sweden	2006	1005	15–92	Repeat cross-section	P, Ha, N/S, B, K, H, Hnd, Ft, E	Smoking
84	Eurobarometer	United Kingdom	2006	1061	15–95	Repeat cross-section	P, Ha, N/S, B, K, H, Hnd, Ft, E	Smoking
85	European Health Survey in Spain (EESE)	Spain	2014	22840	15–100	Household survey	P, Ha, N/S, B	BMI; Smoking; Income
86	European Health Survey in Spain (EESE)	Spain	2020	22069	15–100	Household survey	P, Ha, N/S, B	BMI; Smoking; Income
87	European Social Survey (ESS)	Austria	2014	4143	15–96	Repeat cross-section	P, Ha, B	BMI; Smoking; Income
88	European Social Survey (ESS)	Belgium	2014	3358	15–93	Repeat cross-section	P, Ha, B	BMI; Smoking; Income
89	European Social Survey (ESS)	Bulgaria	2014	2236	16–90	Repeat cross-section	P, Ha, B	BMI; Smoking; Income
90	European Social Survey (ESS)	Croatia	2014	1554	15–90	Repeat cross-section	P, Ha, B	BMI; Smoking; Income
91	European Social Survey (ESS)	Cyprus	2014	666	17–90	Repeat cross-section	P, Ha, B	BMI; Smoking; Income

92	European Social Survey (ESS)	Czech Republic	2014	2117	15–88	Repeat cross-section	P, Ha, B	BMI; Smoking; Income
93	European Social Survey (ESS)	Denmark	2014	1502	15–100	Repeat cross-section	P, Ha, B	BMI; Smoking; Income
94	European Social Survey (ESS)	Finland	2014	3650	15–100	Repeat cross-section	P, Ha, B	BMI; Smoking; Income
95	European Social Survey (ESS)	France	2014	3682	15–99	Repeat cross-section	P, Ha, B	BMI; Smoking; Income
96	European Social Survey (ESS)	Germany	2014	5447	15–100	Repeat cross-section	P, Ha, B	BMI; Smoking; Income
97	European Social Survey (ESS)	Greece	2014	2755	15–90	Repeat cross-section	P, Ha, B	BMI; Smoking; Income
98	European Social Survey (ESS)	Hungary	2014	3780	15–92	Repeat cross-section	P, Ha, B	BMI; Smoking; Income
99	European Social Survey (ESS)	Iceland	2014	839	16–90	Repeat cross-section	P, Ha, B	BMI; Smoking; Income
100	European Social Survey (ESS)	Ireland	2014	4376	15–97	Repeat cross-section	P, Ha, B	BMI; Smoking; Income
101	European Social Survey (ESS)	Israel	2014	3422	15–100	Repeat cross-section	P, Ha, B	BMI; Smoking; Income
102	European Social Survey (ESS)	Italy	2014	2835	15–90	Repeat cross-section	P, Ha, B	BMI; Smoking; Income
103	European Social Survey (ESS)	Latvia	2014	1212	15–90	Repeat cross-section	P, Ha, B	BMI; Smoking; Income
104	European Social Survey (ESS)	Lithuania	2014	3597	15–92	Repeat cross-section	P, Ha, B	BMI; Smoking; Income
105	European Social Survey (ESS)	Montenegro	2014	1587	16–87	Repeat cross-section	P, Ha, B	BMI; Smoking; Income
106	European Social Survey (ESS)	Netherlands	2014	3606	14–95	Repeat cross-section	P, Ha, B	BMI; Smoking; Income
107	European Social Survey (ESS)	Norway	2014	2771	15–100	Repeat cross-section	P, Ha, B	BMI; Smoking; Income
108	European Social Survey (ESS)	Poland	2014	3029	15–90	Repeat cross-section	P, Ha, B	BMI; Smoking; Income
109	European Social Survey (ESS)	Portugal	2014	2638	15–94	Repeat cross-section	P, Ha, B	BMI; Smoking; Income
110	European Social Survey (ESS)	Serbia	2014	1543	15–90	Repeat cross-section	P, Ha, B	BMI; Smoking; Income
111	European Social Survey (ESS)	Slovakia	2014	1431	15–90	Repeat cross-section	P, Ha, B	BMI; Smoking; Income
112	European Social Survey (ESS)	Slovenia	2014	2472	15–94	Repeat cross-section	P, Ha, B	BMI; Smoking; Income
113	European Social Survey (ESS)	Spain	2014	3760	15–100	Repeat cross-section	P, Ha, B	BMI; Smoking; Income
114	European Social Survey (ESS)	Sweden	2014	3019	15–97	Repeat cross-section	P, Ha, B	BMI; Smoking; Income
115	European Social Survey (ESS)	Switzerland	2014	2907	15–92	Repeat cross-section	P, Ha, B	BMI; Smoking; Income
116	European Social Survey (ESS)	United Kingdom	2014	3890	15–94	Repeat cross-section	P, Ha, B	BMI; Smoking; Income
117	European Working Conditions Survey (EWCS)	Albania	2010	992	16–86	Repeat cross-section	P, Ha, N/S, S/A, B	Income
118	European Working Conditions Survey (EWCS)	Albania	2015	1002	16–75	Repeat cross-section	P, Ha, N/S, B	Income
119	European Working Conditions Survey (EWCS)	Albania	2021	501	17–73	Repeat cross-section	P, Ha, N/S, B	Income

120	European Working Conditions Survey (EWCS)	Austria	1995	588	16–70	Repeat cross-section	P, Ha, N/S, S/A, B	
121	European Working Conditions Survey (EWCS)	Austria	2000	704	17–72	Repeat cross-section	P, Ha, N/S, S/A, B	
122	European Working Conditions Survey (EWCS)	Austria	2005	282	16–74	Repeat cross-section	P, Ha, N/S, S/A, B	
123	European Working Conditions Survey (EWCS)	Austria	2010	1003	15–89	Repeat cross-section	P, Ha, N/S, S/A, B	Income
124	European Working Conditions Survey (EWCS)	Austria	2015	1024	15–87	Repeat cross-section	P, Ha, N/S, B	Income
125	European Working Conditions Survey (EWCS)	Austria	2021	889	16–75	Repeat cross-section	P, Ha, N/S, B	Income
126	European Working Conditions Survey (EWCS)	Belgium	1991	344	17–67	Repeat cross-section	P, N/S, B	
127	European Working Conditions Survey (EWCS)	Belgium	1995	398	19–67	Repeat cross-section	P, Ha, N/S, S/A, B	
128	European Working Conditions Survey (EWCS)	Belgium	2000	788	16–88	Repeat cross-section	P, Ha, N/S, S/A, B	
129	European Working Conditions Survey (EWCS)	Belgium	2005	287	18–66	Repeat cross-section	P, Ha, N/S, S/A, B	
130	European Working Conditions Survey (EWCS)	Belgium	2010	3954	16–84	Repeat cross-section	P, Ha, N/S, S/A, B	Income
131	European Working Conditions Survey (EWCS)	Belgium	2015	2583	15–88	Repeat cross-section	P, Ha, N/S, B	Income
132	European Working Conditions Survey (EWCS)	Belgium	2021	2099	16–83	Repeat cross-section	P, Ha, N/S, B	Income
133	European Working Conditions Survey (EWCS)	Bosnia and Herzegovina	2021	568	16–73	Repeat cross-section	P, Ha, N/S, B	Income
134	European Working Conditions Survey (EWCS)	Bulgaria	2001	689	19–64	Repeat cross-section	P, Ha, N/S, S/A, B	
135	European Working Conditions Survey (EWCS)	Bulgaria	2005	521	17–75	Repeat cross-section	P, Ha, N/S, S/A, B	
136	European Working Conditions Survey (EWCS)	Bulgaria	2010	1012	18–77	Repeat cross-section	P, Ha, N/S, S/A, B	Income
137	European Working Conditions Survey (EWCS)	Bulgaria	2015	1064	18–78	Repeat cross-section	P, Ha, N/S, B	Income
138	European Working Conditions Survey (EWCS)	Bulgaria	2021	885	16–80	Repeat cross-section	P, Ha, N/S, B	Income
139	European Working Conditions Survey (EWCS)	Croatia	2005	479	18–76	Repeat cross-section	P, Ha, N/S, S/A, B	
140	European Working Conditions Survey (EWCS)	Croatia	2010	1089	17–80	Repeat cross-section	P, Ha, N/S, S/A, B	Income
141	European Working Conditions Survey (EWCS)	Croatia	2015	1006	17–84	Repeat cross-section	P, Ha, N/S, B	Income
142	European Working Conditions Survey (EWCS)	Croatia	2021	894	18–74	Repeat cross-section	P, Ha, N/S, B	Income
143	European Working Conditions Survey (EWCS)	Cyprus	2001	297	16–71	Repeat cross-section	P, Ha, N/S, S/A, B	
144	European Working Conditions Survey (EWCS)	Cyprus	2005	259	19–76	Repeat cross-section	P, Ha, N/S, S/A, B	
145	European Working Conditions Survey (EWCS)	Cyprus	2010	997	17–84	Repeat cross-section	P, Ha, N/S, S/A, B	Income
146	European Working Conditions Survey (EWCS)	Cyprus	2015	1001	16–82	Repeat cross-section	P, Ha, N/S, B	Income
147	European Working Conditions Survey (EWCS)	Cyprus	2021	681	18–75	Repeat cross-section	P, Ha, N/S, B	Income
148	European Working Conditions Survey (EWCS)	Czech Republic	2001	720	18–69	Repeat cross-section	P, Ha, N/S, S/A, B	
149	European Working Conditions Survey (EWCS)	Czech Republic	2005	324	18–99	Repeat cross-section	P, Ha, N/S, S/A, B	
150	European Working Conditions Survey (EWCS)	Czech Republic	2010	992	18–85	Repeat cross-section	P, Ha, N/S, S/A, B	Income
151	European Working Conditions Survey (EWCS)	Czech Republic	2015	997	18–81	Repeat cross-section	P, Ha, N/S, B	Income
152	European Working Conditions Survey (EWCS)	Czech Republic	2021	997	17–87	Repeat cross-section	P, Ha, N/S, B	Income

153	European Working Conditions Survey (EWCS)	Denmark	1991	589	16–75	Repeat cross-section	P, N/S, B	
154	European Working Conditions Survey (EWCS)	Denmark	1995	598	15–69	Repeat cross-section	P, Ha, N/S, S/A, B	
155	European Working Conditions Survey (EWCS)	Denmark	2000	936	16–70	Repeat cross-section	P, Ha, N/S, S/A, B	
156	European Working Conditions Survey (EWCS)	Denmark	2005	451	15–74	Repeat cross-section	P, Ha, N/S, S/A, B	
157	European Working Conditions Survey (EWCS)	Denmark	2010	1068	15–68	Repeat cross-section	P, Ha, N/S, S/A, B	Income
158	European Working Conditions Survey (EWCS)	Denmark	2015	998	15–76	Repeat cross-section	P, Ha, N/S, B	Income
159	European Working Conditions Survey (EWCS)	Denmark	2021	894	16–75	Repeat cross-section	P, Ha, N/S, B	Income
160	European Working Conditions Survey (EWCS)	Estonia	2001	771	18–74	Repeat cross-section	P, Ha, N/S, S/A, B	
161	European Working Conditions Survey (EWCS)	Estonia	2005	349	18–74	Repeat cross-section	P, Ha, N/S, S/A, B	
162	European Working Conditions Survey (EWCS)	Estonia	2010	1000	18–81	Repeat cross-section	P, Ha, N/S, S/A, B	Income
163	European Working Conditions Survey (EWCS)	Estonia	2015	997	15–86	Repeat cross-section	P, Ha, N/S, B	Income
164	European Working Conditions Survey (EWCS)	Estonia	2021	901	18–77	Repeat cross-section	P, Ha, N/S, B	Income
165	European Working Conditions Survey (EWCS)	Finland	1995	683	17–70	Repeat cross-section	P, Ha, N/S, S/A, B	
166	European Working Conditions Survey (EWCS)	Finland	2000	1190	15–98	Repeat cross-section	P, Ha, N/S, S/A, B	
167	European Working Conditions Survey (EWCS)	Finland	2005	445	16–64	Repeat cross-section	P, Ha, N/S, S/A, B	
168	European Working Conditions Survey (EWCS)	Finland	2010	1027	15–74	Repeat cross-section	P, Ha, N/S, S/A, B	Income
169	European Working Conditions Survey (EWCS)	Finland	2015	999	18–83	Repeat cross-section	P, Ha, N/S, B	Income
170	European Working Conditions Survey (EWCS)	Finland	2021	953	18–80	Repeat cross-section	P, Ha, N/S, B	Income
171	European Working Conditions Survey (EWCS)	France	1991	437	16–63	Repeat cross-section	P, N/S, B	
172	European Working Conditions Survey (EWCS)	France	1995	521	18–63	Repeat cross-section	P, Ha, N/S, S/A, B	
173	European Working Conditions Survey (EWCS)	France	2000	953	16–68	Repeat cross-section	P, Ha, N/S, S/A, B	
174	European Working Conditions Survey (EWCS)	France	2005	298	17–60	Repeat cross-section	P, Ha, N/S, S/A, B	
175	European Working Conditions Survey (EWCS)	France	2010	3042	15–75	Repeat cross-section	P, Ha, N/S, S/A, B	Income
176	European Working Conditions Survey (EWCS)	France	2015	1525	16–75	Repeat cross-section	P, Ha, N/S, B	Income
177	European Working Conditions Survey (EWCS)	France	2021	1603	17–74	Repeat cross-section	P, Ha, N/S, B	Income
178	European Working Conditions Survey (EWCS)	Germany	1991	1050	15–75	Repeat cross-section	P, N/S, B	
179	European Working Conditions Survey (EWCS)	Germany	1995	1200	16–72	Repeat cross-section	P, Ha, N/S, S/A, B	
180	European Working Conditions Survey (EWCS)	Germany	2000	920	15–75	Repeat cross-section	P, Ha, N/S, S/A, B	
181	European Working Conditions Survey (EWCS)	Germany	2005	231	17–65	Repeat cross-section	P, Ha, N/S, S/A, B	
182	European Working Conditions Survey (EWCS)	Germany	2010	2130	15–77	Repeat cross-section	P, Ha, N/S, S/A, B	Income
183	European Working Conditions Survey (EWCS)	Germany	2015	2087	15–79	Repeat cross-section	P, Ha, N/S, B	Income
184	European Working Conditions Survey (EWCS)	Germany	2021	2048	16–80	Repeat cross-section	P, Ha, N/S, B	Income
185	European Working Conditions Survey (EWCS)	Greece	1991	484	16–76	Repeat cross-section	P, N/S, B	

186	European Working Conditions Survey (EWCS)	Greece	1995	782	17–75	Repeat cross-section	P, Ha, N/S, S/A, B	
187	European Working Conditions Survey (EWCS)	Greece	2000	1267	17–78	Repeat cross-section	P, Ha, N/S, S/A, B	
188	European Working Conditions Survey (EWCS)	Greece	2005	707	16–85	Repeat cross-section	P, Ha, N/S, S/A, B	
189	European Working Conditions Survey (EWCS)	Greece	2010	1036	16–80	Repeat cross-section	P, Ha, N/S, S/A, B	Income
190	European Working Conditions Survey (EWCS)	Greece	2015	1007	18–85	Repeat cross-section	P, Ha, N/S, B	Income
191	European Working Conditions Survey (EWCS)	Greece	2021	899	17–74	Repeat cross-section	P, Ha, N/S, B	Income
192	European Working Conditions Survey (EWCS)	Hungary	2001	612	17–79	Repeat cross-section	P, Ha, N/S, S/A, B	
193	European Working Conditions Survey (EWCS)	Hungary	2005	458	20–74	Repeat cross-section	P, Ha, N/S, S/A, B	
194	European Working Conditions Survey (EWCS)	Hungary	2010	1006	16–77	Repeat cross-section	P, Ha, N/S, S/A, B	Income
195	European Working Conditions Survey (EWCS)	Hungary	2015	1011	18–89	Repeat cross-section	P, Ha, N/S, B	Income
196	European Working Conditions Survey (EWCS)	Hungary	2021	893	18–77	Repeat cross-section	P, Ha, N/S, B	Income
197	European Working Conditions Survey (EWCS)	Ireland	1991	281	16–72	Repeat cross-section	P, N/S, B	
198	European Working Conditions Survey (EWCS)	Ireland	1995	291	15–71	Repeat cross-section	P, Ha, N/S, S/A, B	
199	European Working Conditions Survey (EWCS)	Ireland	2000	445	16–78	Repeat cross-section	P, Ha, N/S, S/A, B	
200	European Working Conditions Survey (EWCS)	Ireland	2005	270	16–75	Repeat cross-section	P, Ha, N/S, S/A, B	
201	European Working Conditions Survey (EWCS)	Ireland	2010	994	16–79	Repeat cross-section	P, Ha, N/S, S/A, B	Income
202	European Working Conditions Survey (EWCS)	Ireland	2015	1043	15–87	Repeat cross-section	P, Ha, N/S, B	Income
203	European Working Conditions Survey (EWCS)	Ireland	2021	879	16–77	Repeat cross-section	P, Ha, N/S, B	Income
204	European Working Conditions Survey (EWCS)	Italy	1991	315	15–64	Repeat cross-section	P, N/S, B	
205	European Working Conditions Survey (EWCS)	Italy	1995	686	15–66	Repeat cross-section	P, Ha, N/S, S/A, B	
206	European Working Conditions Survey (EWCS)	Italy	2000	936	16–68	Repeat cross-section	P, Ha, N/S, S/A, B	
207	European Working Conditions Survey (EWCS)	Italy	2005	369	19–80	Repeat cross-section	P, Ha, N/S, S/A, B	
208	European Working Conditions Survey (EWCS)	Italy	2010	1466	16–76	Repeat cross-section	P, Ha, N/S, S/A, B	Income
209	European Working Conditions Survey (EWCS)	Italy	2015	1398	18–86	Repeat cross-section	P, Ha, N/S, B	Income
210	European Working Conditions Survey (EWCS)	Italy	2021	1563	16–84	Repeat cross-section	P, Ha, N/S, B	Income
211	European Working Conditions Survey (EWCS)	Kosovo	2010	1013	15–79	Repeat cross-section	P, Ha, N/S, S/A, B	Income
212	European Working Conditions Survey (EWCS)	Kosovo	2021	573	17–64	Repeat cross-section	P, Ha, N/S, B	Income
213	European Working Conditions Survey (EWCS)	Latvia	2001	795	16–80	Repeat cross-section	P, Ha, N/S, S/A, B	
214	European Working Conditions Survey (EWCS)	Latvia	2005	623	17–74	Repeat cross-section	P, Ha, N/S, S/A, B	
215	European Working Conditions Survey (EWCS)	Latvia	2010	1000	18–80	Repeat cross-section	P, Ha, N/S, S/A, B	Income
216	European Working Conditions Survey (EWCS)	Latvia	2015	989	17–86	Repeat cross-section	P, Ha, N/S, B	Income
217	European Working Conditions Survey (EWCS)	Latvia	2021	893	17–77	Repeat cross-section	P, Ha, N/S, B	Income
218	European Working Conditions Survey (EWCS)	Lithuania	2001	741	19–79	Repeat cross-section	P, Ha, N/S, S/A, B	

219	European Working Conditions Survey (EWCS)	Lithuania	2005	481	19–75	Repeat cross-section	P, Ha, N/S, S/A, B	
220	European Working Conditions Survey (EWCS)	Lithuania	2010	1003	16–80	Repeat cross-section	P, Ha, N/S, S/A, B	Income
221	European Working Conditions Survey (EWCS)	Lithuania	2015	1003	17–86	Repeat cross-section	P, Ha, N/S, B	Income
222	European Working Conditions Survey (EWCS)	Lithuania	2021	933	19–77	Repeat cross-section	P, Ha, N/S, B	Income
223	European Working Conditions Survey (EWCS)	Luxembourg	1991	237	18–71	Repeat cross-section	P, N/S, B	
224	European Working Conditions Survey (EWCS)	Luxembourg	1995	260	17–70	Repeat cross-section	P, Ha, N/S, S/A, B	
225	European Working Conditions Survey (EWCS)	Luxembourg	2000	305	16–80	Repeat cross-section	P, Ha, N/S, S/A, B	
226	European Working Conditions Survey (EWCS)	Luxembourg	2005	220	18–67	Repeat cross-section	P, Ha, N/S, S/A, B	
227	European Working Conditions Survey (EWCS)	Luxembourg	2010	985	16–81	Repeat cross-section	P, Ha, N/S, S/A, B	Income
228	European Working Conditions Survey (EWCS)	Luxembourg	2015	1002	17–79	Repeat cross-section	P, Ha, N/S, B	Income
229	European Working Conditions Survey (EWCS)	Luxembourg	2021	672	16–74	Repeat cross-section	P, Ha, N/S, B	Income
230	European Working Conditions Survey (EWCS)	Malta	2001	339	16–79	Repeat cross-section	P, Ha, N/S, S/A, B	
231	European Working Conditions Survey (EWCS)	Malta	2005	298	16–74	Repeat cross-section	P, Ha, N/S, S/A, B	
232	European Working Conditions Survey (EWCS)	Malta	2010	1000	16–78	Repeat cross-section	P, Ha, N/S, S/A, B	Income
233	European Working Conditions Survey (EWCS)	Malta	2015	1001	16–75	Repeat cross-section	P, Ha, N/S, B	Income
234	European Working Conditions Survey (EWCS)	Malta	2021	732	16–76	Repeat cross-section	P, Ha, N/S, B	Income
235	European Working Conditions Survey (EWCS)	Montenegro	2010	1037	18–74	Repeat cross-section	P, Ha, N/S, S/A, B	Income
236	European Working Conditions Survey (EWCS)	Montenegro	2015	1005	16–83	Repeat cross-section	P, Ha, N/S, B	Income
237	European Working Conditions Survey (EWCS)	Montenegro	2021	573	17–70	Repeat cross-section	P, Ha, N/S, B	Income
238	European Working Conditions Survey (EWCS)	Netherlands	1991	367	17–60	Repeat cross-section	P, N/S, B	
239	European Working Conditions Survey (EWCS)	Netherlands	1995	478	17–63	Repeat cross-section	P, Ha, N/S, S/A, B	
240	European Working Conditions Survey (EWCS)	Netherlands	2000	896	15–64	Repeat cross-section	P, Ha, N/S, S/A, B	
241	European Working Conditions Survey (EWCS)	Netherlands	2005	275	17–64	Repeat cross-section	P, Ha, N/S, S/A, B	
242	European Working Conditions Survey (EWCS)	Netherlands	2010	1017	15–78	Repeat cross-section	P, Ha, N/S, S/A, B	Income
243	European Working Conditions Survey (EWCS)	Netherlands	2015	1023	15–81	Repeat cross-section	P, Ha, N/S, B	Income
244	European Working Conditions Survey (EWCS)	Netherlands	2021	898	16–75	Repeat cross-section	P, Ha, N/S, B	Income
245	European Working Conditions Survey (EWCS)	North Macedonia	2010	1099	17–82	Repeat cross-section	P, Ha, N/S, S/A, B	Income
246	European Working Conditions Survey (EWCS)	North Macedonia	2015	1005	15–82	Repeat cross-section	P, Ha, N/S, B	Income
247	European Working Conditions Survey (EWCS)	North Macedonia	2021	551	17–71	Repeat cross-section	P, Ha, N/S, B	Income
248	European Working Conditions Survey (EWCS)	Norway	2005	453	16–72	Repeat cross-section	P, Ha, N/S, S/A, B	
249	European Working Conditions Survey (EWCS)	Norway	2010	1085	16–89	Repeat cross-section	P, Ha, N/S, S/A, B	Income
250	European Working Conditions Survey (EWCS)	Norway	2015	1028	15–73	Repeat cross-section	P, Ha, N/S, B	Income
251	European Working Conditions Survey (EWCS)	Norway	2021	1644	16–82	Repeat cross-section	P, Ha, N/S, B	Income

252	European Working Conditions Survey (EWCS)	Poland	2001	724	17–72	Repeat cross-section	P, Ha, N/S, S/A, B	
253	European Working Conditions Survey (EWCS)	Poland	2005	607	16–74	Repeat cross-section	P, Ha, N/S, S/A, B	
254	European Working Conditions Survey (EWCS)	Poland	2010	1471	16–80	Repeat cross-section	P, Ha, N/S, S/A, B	Income
255	European Working Conditions Survey (EWCS)	Poland	2015	1142	16–85	Repeat cross-section	P, Ha, N/S, B	Income
256	European Working Conditions Survey (EWCS)	Poland	2021	1439	16–88	Repeat cross-section	P, Ha, N/S, B	Income
257	European Working Conditions Survey (EWCS)	Portugal	1991	320	15–78	Repeat cross-section	P, N/S, B	
258	European Working Conditions Survey (EWCS)	Portugal	1995	696	15–76	Repeat cross-section	P, Ha, N/S, S/A, B	
259	European Working Conditions Survey (EWCS)	Portugal	2000	880	16–76	Repeat cross-section	P, Ha, N/S, S/A, B	
260	European Working Conditions Survey (EWCS)	Portugal	2005	380	18–75	Repeat cross-section	P, Ha, N/S, S/A, B	
261	European Working Conditions Survey (EWCS)	Portugal	2010	999	18–87	Repeat cross-section	P, Ha, N/S, S/A, B	Income
262	European Working Conditions Survey (EWCS)	Portugal	2015	1034	18–87	Repeat cross-section	P, Ha, N/S, B	Income
263	European Working Conditions Survey (EWCS)	Portugal	2021	943	18–75	Repeat cross-section	P, Ha, N/S, B	Income
264	European Working Conditions Survey (EWCS)	Romania	2001	619	17–81	Repeat cross-section	P, Ha, N/S, S/A, B	
265	European Working Conditions Survey (EWCS)	Romania	2005	499	16–89	Repeat cross-section	P, Ha, N/S, S/A, B	
266	European Working Conditions Survey (EWCS)	Romania	2010	1010	17–84	Repeat cross-section	P, Ha, N/S, S/A, B	Income
267	European Working Conditions Survey (EWCS)	Romania	2015	1061	18–83	Repeat cross-section	P, Ha, N/S, B	Income
268	European Working Conditions Survey (EWCS)	Romania	2021	908	18–74	Repeat cross-section	P, Ha, N/S, B	Income
269	European Working Conditions Survey (EWCS)	Serbia	2015	1027	15–85	Repeat cross-section	P, Ha, N/S, B	Income
270	European Working Conditions Survey (EWCS)	Serbia	2021	571	17–68	Repeat cross-section	P, Ha, N/S, B	Income
271	European Working Conditions Survey (EWCS)	Slovakia	2001	727	16–73	Repeat cross-section	P, Ha, N/S, S/A, B	
272	European Working Conditions Survey (EWCS)	Slovakia	2005	522	20–65	Repeat cross-section	P, Ha, N/S, S/A, B	
273	European Working Conditions Survey (EWCS)	Slovakia	2010	1001	15–82	Repeat cross-section	P, Ha, N/S, S/A, B	Income
274	European Working Conditions Survey (EWCS)	Slovakia	2015	989	16–81	Repeat cross-section	P, Ha, N/S, B	Income
275	European Working Conditions Survey (EWCS)	Slovakia	2021	894	18–80	Repeat cross-section	P, Ha, N/S, B	Income
276	European Working Conditions Survey (EWCS)	Slovenia	2001	635	18–76	Repeat cross-section	P, Ha, N/S, S/A, B	
277	European Working Conditions Survey (EWCS)	Slovenia	2005	361	17–76	Repeat cross-section	P, Ha, N/S, S/A, B	
278	European Working Conditions Survey (EWCS)	Slovenia	2010	1397	16–81	Repeat cross-section	P, Ha, N/S, S/A, B	Income
279	European Working Conditions Survey (EWCS)	Slovenia	2015	1599	17–86	Repeat cross-section	P, Ha, N/S, B	Income
280	European Working Conditions Survey (EWCS)	Slovenia	2021	1308	16–70	Repeat cross-section	P, Ha, N/S, B	Income
281	European Working Conditions Survey (EWCS)	Spain	1991	347	17–89	Repeat cross-section	P, N/S, B	
282	European Working Conditions Survey (EWCS)	Spain	1995	567	16–67	Repeat cross-section	P, Ha, N/S, S/A, B	
283	European Working Conditions Survey (EWCS)	Spain	2000	987	17–75	Repeat cross-section	P, Ha, N/S, S/A, B	
284	European Working Conditions Survey (EWCS)	Spain	2005	328	17–70	Repeat cross-section	P, Ha, N/S, S/A, B	

285	European Working Conditions Survey (EWCS)	Spain	2010	1007	17–70	Repeat cross-section	P, Ha, N/S, S/A, B	Income
286	European Working Conditions Survey (EWCS)	Spain	2015	3357	15–87	Repeat cross-section	P, Ha, N/S, B	Income
287	European Working Conditions Survey (EWCS)	Spain	2021	1445	18–88	Repeat cross-section	P, Ha, N/S, B	Income
288	European Working Conditions Survey (EWCS)	Sweden	1995	741	18–70	Repeat cross-section	P, Ha, N/S, S/A, B	
289	European Working Conditions Survey (EWCS)	Sweden	2000	1094	16–79	Repeat cross-section	P, Ha, N/S, S/A, B	
290	European Working Conditions Survey (EWCS)	Sweden	2005	567	16–66	Repeat cross-section	P, Ha, N/S, S/A, B	
291	European Working Conditions Survey (EWCS)	Sweden	2010	972	17–77	Repeat cross-section	P, Ha, N/S, S/A, B	Income
292	European Working Conditions Survey (EWCS)	Sweden	2015	1002	17–75	Repeat cross-section	P, Ha, N/S, B	Income
293	European Working Conditions Survey (EWCS)	Sweden	2021	909	16–84	Repeat cross-section	P, Ha, N/S, B	Income
294	European Working Conditions Survey (EWCS)	Switzerland	2005	329	19–86	Repeat cross-section	P, Ha, N/S, S/A, B	
295	European Working Conditions Survey (EWCS)	Switzerland	2015	1003	16–88	Repeat cross-section	P, Ha, N/S, B	Income
296	European Working Conditions Survey (EWCS)	Switzerland	2021	619	16–79	Repeat cross-section	P, Ha, N/S, B	Income
297	European Working Conditions Survey (EWCS)	Turkiye	2005	464	16–75	Repeat cross-section	P, Ha, N/S, S/A, B	
298	European Working Conditions Survey (EWCS)	Turkiye	2010	2099	16–80	Repeat cross-section	P, Ha, N/S, S/A, B	Income
299	European Working Conditions Survey (EWCS)	Turkiye	2015	1994	15–84	Repeat cross-section	P, Ha, N/S, B	Income
300	European Working Conditions Survey (EWCS)	United Kingdom	1991	526	17–72	Repeat cross-section	P, N/S, B	
301	European Working Conditions Survey (EWCS)	United Kingdom	1995	524	15–65	Repeat cross-section	P, Ha, N/S, S/A, B	
302	European Working Conditions Survey (EWCS)	United Kingdom	2000	711	16–72	Repeat cross-section	P, Ha, N/S, S/A, B	
303	European Working Conditions Survey (EWCS)	United Kingdom	2005	212	17–70	Repeat cross-section	P, Ha, N/S, S/A, B	
304	European Working Conditions Survey (EWCS)	United Kingdom	2010	1564	16–91	Repeat cross-section	P, Ha, N/S, S/A, B	Income
305	European Working Conditions Survey (EWCS)	United Kingdom	2015	1618	16–84	Repeat cross-section	P, Ha, N/S, B	Income
306	European Working Conditions Survey (EWCS)	United Kingdom	2021	1047	18–81	Repeat cross-section	P, Ha, N/S, B	Income
307	Gender and Adolescence: Global Evidence	Bangladesh	2017	780	10–17	Household survey	Ha, S/A	
308	Gender and Adolescence: Global Evidence	Ethiopia	2017	6586	10–17	Household survey	Ha, S/A	
309	Gender and Adolescence: Global Evidence	Jordan	2018	4101	10–18	Household survey	Ha, S/A	
310	Generation 21 (G21)[a,f]	Portugal	2012	5814	7–7	Cohort	P, Ha, C, S/A, B	
311	German Health Interview and Examination Survey for Children and Adolescents (KiGGS)[b]	Germany	2003	14560	5–16	Panel	P, Ha, C, S/A, B, F	BMI; Income
312	German National Health Interview and Examination Survey 1998 (GNHIES98)	Germany	1998	6968	20–77	Cross-section	PI, P, Ha, N/S, C, S/A, B, K, H, F, Hnd, Ft	BMI; Smoking; Income
313	Health Behavior in School-aged Children (HBSC)	Albania	2014	4845	10–16	School-based survey	Ha, S/A, B	BMI
314	Health Behavior in School-aged Children (HBSC)	Albania	2018	1647	11–16	School-based survey	Ha, S/A, B	BMI
315	Health Behavior in School-aged Children (HBSC)	Armenia	2010	2394	10–16	School-based survey	Ha, S/A, B	BMI
316	Health Behavior in School-aged Children (HBSC)	Armenia	2014	3019	10–16	School-based survey	Ha, S/A, B	BMI
317	Health Behavior in School-aged Children (HBSC)	Armenia	2018	4123	11–16	School-based survey	Ha, S/A, B	BMI

318	Health Behavior in School-aged Children (HBSC)	Austria	2001	4294	10–16	School-based survey	Ha, S/A, B	BMI
319	Health Behavior in School-aged Children (HBSC)	Austria	2006	4718	10–16	School-based survey	Ha, S/A, B	BMI
320	Health Behavior in School-aged Children (HBSC)	Austria	2010	4929	10–16	School-based survey	Ha, S/A, B	BMI
321	Health Behavior in School-aged Children (HBSC)	Austria	2014	3358	11–16	School-based survey	Ha, S/A, B	BMI
322	Health Behavior in School-aged Children (HBSC)	Austria	2018	4044	10–16	School-based survey	Ha, S/A, B	BMI
323	Health Behavior in School-aged Children (HBSC)	Azerbaijan	2018	4397	10–16	School-based survey	Ha, S/A, B	BMI
324	Health Behavior in School-aged Children (HBSC)	Belgium	2001	10438	11–16	School-based survey	Ha, S/A, B	BMI
325	Health Behavior in School-aged Children (HBSC)	Belgium	2006	8417	11–16	School-based survey	Ha, S/A, B	BMI
326	Health Behavior in School-aged Children (HBSC)	Belgium	2010	7943	10–16	School-based survey	Ha, S/A, B	BMI
327	Health Behavior in School-aged Children (HBSC)	Belgium	2014	10050	10–16	School-based survey	Ha, S/A, B	BMI
328	Health Behavior in School-aged Children (HBSC)	Belgium	2018	9702	10–16	School-based survey	Ha, S/A, B	BMI
329	Health Behavior in School-aged Children (HBSC)	Bulgaria	2006	4799	11–16	School-based survey	Ha, S/A, B	BMI
330	Health Behavior in School-aged Children (HBSC)	Bulgaria	2014	4705	11–16	School-based survey	Ha, S/A, B	BMI
331	Health Behavior in School-aged Children (HBSC)	Bulgaria	2018	4548	11–16	School-based survey	Ha, S/A, B	BMI
332	Health Behavior in School-aged Children (HBSC)	Canada	2001	4321	11–16	School-based survey	Ha, S/A, B	BMI
333	Health Behavior in School-aged Children (HBSC)	Canada	2006	5720	11–16	School-based survey	Ha, S/A, B	BMI
334	Health Behavior in School-aged Children (HBSC)	Canada	2010	15295	11–16	School-based survey	Ha, S/A, B	BMI
335	Health Behavior in School-aged Children (HBSC)	Canada	2014	12549	11–16	School-based survey	Ha, S/A, B	BMI
336	Health Behavior in School-aged Children (HBSC)	Canada	2018	12243	11–16	School-based survey	Ha, S/A, B	BMI
337	Health Behavior in School-aged Children (HBSC)	Croatia	2001	4317	10–16	School-based survey	Ha, S/A, B	BMI
338	Health Behavior in School-aged Children (HBSC)	Croatia	2006	4854	11–16	School-based survey	Ha, S/A, B	BMI
339	Health Behavior in School-aged Children (HBSC)	Croatia	2010	6203	10–16	School-based survey	Ha, S/A, B	BMI
340	Health Behavior in School-aged Children (HBSC)	Croatia	2014	5335	11–16	School-based survey	Ha, S/A, B	BMI
341	Health Behavior in School-aged Children (HBSC)	Croatia	2018	4901	11–16	School-based survey	Ha, S/A, B	BMI
342	Health Behavior in School-aged Children (HBSC)	Czech Republic	2001	4954	11–16	School-based survey	Ha, S/A, B	BMI
343	Health Behavior in School-aged Children (HBSC)	Czech Republic	2006	4698	11–16	School-based survey	Ha, S/A, B	BMI
344	Health Behavior in School-aged Children (HBSC)	Czech Republic	2010	4305	11–16	School-based survey	Ha, S/A, B	BMI
345	Health Behavior in School-aged Children (HBSC)	Czech Republic	2014	5018	10–16	School-based survey	Ha, S/A, B	BMI
346	Health Behavior in School-aged Children (HBSC)	Czech Republic	2018	11010	10–16	School-based survey	Ha, S/A, B	BMI
347	Health Behavior in School-aged Children (HBSC)	Denmark	2001	4538	11–17	School-based survey	Ha, S/A, B	BMI
348	Health Behavior in School-aged Children (HBSC)	Denmark	2006	5586	11–17	School-based survey	Ha, S/A, B	BMI
349	Health Behavior in School-aged Children (HBSC)	Denmark	2010	3987	11–16	School-based survey	Ha, S/A, B	BMI
350	Health Behavior in School-aged Children (HBSC)	Denmark	2014	3660	11–16	School-based survey	Ha, S/A, B	BMI

351	Health Behavior in School-aged Children (HBSC)	Denmark	2018	3118	11–16	School-based survey	Ha, S/A, B	BMI
352	Health Behavior in School-aged Children (HBSC)	Estonia	2001	3971	10–16	School-based survey	Ha, S/A, B	BMI
353	Health Behavior in School-aged Children (HBSC)	Estonia	2006	4407	11–16	School-based survey	Ha, S/A, B	BMI
354	Health Behavior in School-aged Children (HBSC)	Estonia	2010	4193	11–16	School-based survey	Ha, S/A, B	BMI
355	Health Behavior in School-aged Children (HBSC)	Estonia	2014	4004	11–16	School-based survey	Ha, S/A, B	BMI
356	Health Behavior in School-aged Children (HBSC)	Estonia	2018	4677	11–16	School-based survey	Ha, S/A, B	BMI
357	Health Behavior in School-aged Children (HBSC)	Finland	2001	5298	11–17	School-based survey	Ha, S/A, B	BMI
358	Health Behavior in School-aged Children (HBSC)	Finland	2006	5158	11–16	School-based survey	Ha, S/A, B	BMI
359	Health Behavior in School-aged Children (HBSC)	Finland	2010	6570	11–16	School-based survey	Ha, S/A, B	BMI
360	Health Behavior in School-aged Children (HBSC)	Finland	2014	5798	11–16	School-based survey	Ha, S/A, B	BMI
361	Health Behavior in School-aged Children (HBSC)	Finland	2018	3117	11–16	School-based survey	Ha, S/A, B	BMI
362	Health Behavior in School-aged Children (HBSC)	France	2001	7869	10–16	School-based survey	Ha, S/A, B	BMI
363	Health Behavior in School-aged Children (HBSC)	France	2006	7052	11–16	School-based survey	Ha, S/A, B	BMI
364	Health Behavior in School-aged Children (HBSC)	France	2010	6018	10–16	School-based survey	Ha, S/A, B	BMI
365	Health Behavior in School-aged Children (HBSC)	France	2014	5554	10–16	School-based survey	Ha, S/A, B	BMI
366	Health Behavior in School-aged Children (HBSC)	France	2018	8762	11–16	School-based survey	Ha, S/A, B	BMI
367	Health Behavior in School-aged Children (HBSC)	Georgia	2018	3864	11–16	School-based survey	Ha, S/A, B	BMI
368	Health Behavior in School-aged Children (HBSC)	Germany	2001	5485	11–17	School-based survey	Ha, S/A, B	BMI
369	Health Behavior in School-aged Children (HBSC)	Germany	2006	7133	10–16	School-based survey	Ha, S/A, B	BMI
370	Health Behavior in School-aged Children (HBSC)	Germany	2010	4915	10–16	School-based survey	Ha, S/A, B	BMI
371	Health Behavior in School-aged Children (HBSC)	Germany	2014	5846	10–16	School-based survey	Ha, S/A, B	BMI
372	Health Behavior in School-aged Children (HBSC)	Germany	2018	4286	10–16	School-based survey	Ha, S/A, B	BMI
373	Health Behavior in School-aged Children (HBSC)	Greece	2001	3718	11–16	School-based survey	Ha, S/A, B	BMI
374	Health Behavior in School-aged Children (HBSC)	Greece	2006	3655	11–16	School-based survey	Ha, S/A, B	BMI
375	Health Behavior in School-aged Children (HBSC)	Greece	2010	4835	11–16	School-based survey	Ha, S/A, B	BMI
376	Health Behavior in School-aged Children (HBSC)	Greece	2014	4074	11–16	School-based survey	Ha, S/A, B	BMI
377	Health Behavior in School-aged Children (HBSC)	Greece	2018	3799	11–16	School-based survey	Ha, S/A, B	BMI
378	Health Behavior in School-aged Children (HBSC)	Greenland	2001	817	11–17	School-based survey	Ha, S/A, B	BMI
379	Health Behavior in School-aged Children (HBSC)	Greenland	2006	1204	11–16	School-based survey	Ha, S/A, B	BMI
380	Health Behavior in School-aged Children (HBSC)	Greenland	2010	992	10–16	School-based survey	Ha, S/A, B	BMI
381	Health Behavior in School-aged Children (HBSC)	Greenland	2014	882	10–16	School-based survey	Ha, S/A, B	BMI
382	Health Behavior in School-aged Children (HBSC)	Greenland	2018	1018	10–16	School-based survey	Ha, S/A, B	BMI
383	Health Behavior in School-aged Children (HBSC)	Hungary	2001	4036	11–16	School-based survey	Ha, S/A, B	BMI

384	Health Behavior in School-aged Children (HBSC)	Hungary	2006	3424	11–16	School-based survey	Ha, S/A, B	BMI
385	Health Behavior in School-aged Children (HBSC)	Hungary	2010	4742	11–16	School-based survey	Ha, S/A, B	BMI
386	Health Behavior in School-aged Children (HBSC)	Hungary	2014	3765	11–16	School-based survey	Ha, S/A, B	BMI
387	Health Behavior in School-aged Children (HBSC)	Hungary	2018	3682	11–16	School-based survey	Ha, S/A, B	BMI
388	Health Behavior in School-aged Children (HBSC)	Iceland	2006	9323	11–16	School-based survey	Ha, S/A, B	BMI
389	Health Behavior in School-aged Children (HBSC)	Iceland	2010	10877	10–16	School-based survey	Ha, S/A, B	BMI
390	Health Behavior in School-aged Children (HBSC)	Iceland	2014	10339	11–16	School-based survey	Ha, S/A, B	BMI
391	Health Behavior in School-aged Children (HBSC)	Iceland	2018	6842	11–16	School-based survey	Ha, S/A, B	BMI
392	Health Behavior in School-aged Children (HBSC)	Ireland	2001	2819	11–16	School-based survey	Ha, S/A, B	BMI
393	Health Behavior in School-aged Children (HBSC)	Ireland	2006	4746	11–16	School-based survey	Ha, S/A, B	BMI
394	Health Behavior in School-aged Children (HBSC)	Ireland	2010	4526	11–16	School-based survey	Ha, S/A, B	BMI
395	Health Behavior in School-aged Children (HBSC)	Ireland	2014	4002	11–16	School-based survey	Ha, S/A, B	BMI
396	Health Behavior in School-aged Children (HBSC)	Ireland	2018	3740	11–16	School-based survey	Ha, S/A, B	BMI
397	Health Behavior in School-aged Children (HBSC)	Israel	2001	5195	11–17	School-based survey	Ha, S/A, B	BMI
398	Health Behavior in School-aged Children (HBSC)	Israel	2006	4819	11–16	School-based survey	Ha, S/A, B	BMI
399	Health Behavior in School-aged Children (HBSC)	Israel	2010	3806	11–16	School-based survey	Ha, S/A, B	BMI
400	Health Behavior in School-aged Children (HBSC)	Israel	2014	5225	11–16	School-based survey	Ha, S/A, B	BMI
401	Health Behavior in School-aged Children (HBSC)	Israel	2018	7712	11–16	School-based survey	Ha, S/A, B	BMI
402	Health Behavior in School-aged Children (HBSC)	Italy	2001	4329	11–17	School-based survey	Ha, S/A, B	BMI
403	Health Behavior in School-aged Children (HBSC)	Italy	2006	3892	11–16	School-based survey	Ha, S/A, B	BMI
404	Health Behavior in School-aged Children (HBSC)	Italy	2010	4753	10–16	School-based survey	Ha, S/A, B	BMI
405	Health Behavior in School-aged Children (HBSC)	Italy	2014	3965	11–16	School-based survey	Ha, S/A, B	BMI
406	Health Behavior in School-aged Children (HBSC)	Italy	2018	4092	11–16	School-based survey	Ha, S/A, B	BMI
407	Health Behavior in School-aged Children (HBSC)	Kazakhstan	2018	4499	10–16	School-based survey	Ha, S/A, B	BMI
408	Health Behavior in School-aged Children (HBSC)	Latvia	2001	3305	11–16	School-based survey	Ha, S/A, B	BMI
409	Health Behavior in School-aged Children (HBSC)	Latvia	2006	4173	11–16	School-based survey	Ha, S/A, B	BMI
410	Health Behavior in School-aged Children (HBSC)	Latvia	2010	4110	11–16	School-based survey	Ha, S/A, B	BMI
411	Health Behavior in School-aged Children (HBSC)	Latvia	2014	5441	11–16	School-based survey	Ha, S/A, B	BMI
412	Health Behavior in School-aged Children (HBSC)	Latvia	2018	4348	11–16	School-based survey	Ha, S/A, B	BMI
413	Health Behavior in School-aged Children (HBSC)	Lithuania	2001	5549	11–17	School-based survey	Ha, S/A, B	BMI
414	Health Behavior in School-aged Children (HBSC)	Lithuania	2006	5505	11–16	School-based survey	Ha, S/A, B	BMI
415	Health Behavior in School-aged Children (HBSC)	Lithuania	2010	5251	11–16	School-based survey	Ha, S/A, B	BMI
416	Health Behavior in School-aged Children (HBSC)	Lithuania	2018	3729	10–16	School-based survey	Ha, S/A, B	BMI

417	Health Behavior in School-aged Children (HBSC)	Luxembourg	2006	4213	11–16	School-based survey	Ha, S/A, B	BMI
418	Health Behavior in School-aged Children (HBSC)	Luxembourg	2010	3961	10–16	School-based survey	Ha, S/A, B	BMI
419	Health Behavior in School-aged Children (HBSC)	Luxembourg	2014	3062	11–16	School-based survey	Ha, S/A, B	BMI
420	Health Behavior in School-aged Children (HBSC)	Luxembourg	2018	3803	10–16	School-based survey	Ha, S/A, B	BMI
421	Health Behavior in School-aged Children (HBSC)	Malta	2001	1863	11–17	School-based survey	Ha, S/A, B	BMI
422	Health Behavior in School-aged Children (HBSC)	Malta	2006	1333	11–16	School-based survey	Ha, S/A, B	BMI
423	Health Behavior in School-aged Children (HBSC)	Malta	2014	2167	11–16	School-based survey	Ha, S/A, B	BMI
424	Health Behavior in School-aged Children (HBSC)	Malta	2018	2499	11–16	School-based survey	Ha, S/A, B	BMI
425	Health Behavior in School-aged Children (HBSC)	Moldova	2014	4642	11–16	School-based survey	Ha, S/A, B	BMI
426	Health Behavior in School-aged Children (HBSC)	Moldova	2018	4536	11–16	School-based survey	Ha, S/A, B	BMI
427	Health Behavior in School-aged Children (HBSC)	Netherlands	2001	4206	11–16	School-based survey	Ha, S/A, B	BMI
428	Health Behavior in School-aged Children (HBSC)	Netherlands	2006	4131	11–16	School-based survey	Ha, S/A, B	BMI
429	Health Behavior in School-aged Children (HBSC)	Netherlands	2010	4449	11–16	School-based survey	Ha, S/A, B	BMI
430	Health Behavior in School-aged Children (HBSC)	Netherlands	2014	4167	11–16	School-based survey	Ha, S/A, B	BMI
431	Health Behavior in School-aged Children (HBSC)	Netherlands	2018	4669	11–16	School-based survey	Ha, S/A, B	BMI
432	Health Behavior in School-aged Children (HBSC)	North Macedonia	2001	3933	10–16	School-based survey	Ha, S/A, B	BMI
433	Health Behavior in School-aged Children (HBSC)	North Macedonia	2006	5202	11–16	School-based survey	Ha, S/A, B	BMI
434	Health Behavior in School-aged Children (HBSC)	North Macedonia	2010	3702	10–16	School-based survey	Ha, S/A, B	BMI
435	Health Behavior in School-aged Children (HBSC)	North Macedonia	2014	3914	11–16	School-based survey	Ha, S/A, B	BMI
436	Health Behavior in School-aged Children (HBSC)	North Macedonia	2018	4208	11–16	School-based survey	Ha, S/A, B	BMI
437	Health Behavior in School-aged Children (HBSC)	Norway	2001	4886	10–16	School-based survey	Ha, S/A, B	BMI
438	Health Behavior in School-aged Children (HBSC)	Norway	2006	4549	11–16	School-based survey	Ha, S/A, B	BMI
439	Health Behavior in School-aged Children (HBSC)	Norway	2010	4254	11–16	School-based survey	Ha, S/A, B	BMI
440	Health Behavior in School-aged Children (HBSC)	Norway	2014	3208	11–16	School-based survey	Ha, S/A, B	BMI
441	Health Behavior in School-aged Children (HBSC)	Norway	2018	3044	11–16	School-based survey	Ha, S/A, B	BMI
442	Health Behavior in School-aged Children (HBSC)	Poland	2001	6245	11–17	School-based survey	Ha, S/A, B	BMI
443	Health Behavior in School-aged Children (HBSC)	Poland	2006	5466	11–16	School-based survey	Ha, S/A, B	BMI
444	Health Behavior in School-aged Children (HBSC)	Poland	2010	4203	11–16	School-based survey	Ha, S/A, B	BMI
445	Health Behavior in School-aged Children (HBSC)	Poland	2014	4427	11–16	School-based survey	Ha, S/A, B	BMI
446	Health Behavior in School-aged Children (HBSC)	Poland	2018	5097	11–16	School-based survey	Ha, S/A, B	BMI
447	Health Behavior in School-aged Children (HBSC)	Portugal	2001	2835	11–17	School-based survey	Ha, S/A, B	BMI
448	Health Behavior in School-aged Children (HBSC)	Portugal	2006	3880	11–16	School-based survey	Ha, S/A, B	BMI
449	Health Behavior in School-aged Children (HBSC)	Portugal	2010	4002	10–16	School-based survey	Ha, S/A, B	BMI

450	Health Behavior in School-aged Children (HBSC)	Portugal	2014	4771	11–16	School-based survey	Ha, S/A, B	BMI
451	Health Behavior in School-aged Children (HBSC)	Portugal	2018	5756	11–16	School-based survey	Ha, S/A, B	BMI
452	Health Behavior in School-aged Children (HBSC)	Romania	2006	4538	11–16	School-based survey	Ha, S/A, B	BMI
453	Health Behavior in School-aged Children (HBSC)	Romania	2010	5119	11–16	School-based survey	Ha, S/A, B	BMI
454	Health Behavior in School-aged Children (HBSC)	Romania	2014	3785	10–16	School-based survey	Ha, S/A, B	BMI
455	Health Behavior in School-aged Children (HBSC)	Romania	2018	4371	10–16	School-based survey	Ha, S/A, B	BMI
456	Health Behavior in School-aged Children (HBSC)	Russian Federation	2001	7965	11–17	School-based survey	Ha, S/A, B	BMI
457	Health Behavior in School-aged Children (HBSC)	Russian Federation	2006	7911	11–16	School-based survey	Ha, S/A, B	BMI
458	Health Behavior in School-aged Children (HBSC)	Russian Federation	2010	4999	11–16	School-based survey	Ha, S/A, B	BMI
459	Health Behavior in School-aged Children (HBSC)	Russian Federation	2014	4394	11–16	School-based survey	Ha, S/A, B	BMI
460	Health Behavior in School-aged Children (HBSC)	Russian Federation	2018	4215	10–16	School-based survey	Ha, S/A, B	BMI
461	Health Behavior in School-aged Children (HBSC)	Serbia	2018	3789	11–16	School-based survey	Ha, S/A, B	BMI
462	Health Behavior in School-aged Children (HBSC)	Slovakia	2006	3661	10–16	School-based survey	Ha, S/A, B	BMI
463	Health Behavior in School-aged Children (HBSC)	Slovakia	2010	5195	11–16	School-based survey	Ha, S/A, B	BMI
464	Health Behavior in School-aged Children (HBSC)	Slovakia	2014	5933	11–16	School-based survey	Ha, S/A, B	BMI
465	Health Behavior in School-aged Children (HBSC)	Slovakia	2018	4512	11–16	School-based survey	Ha, S/A, B	BMI
466	Health Behavior in School-aged Children (HBSC)	Slovenia	2001	3887	11–17	School-based survey	Ha, S/A, B	BMI
467	Health Behavior in School-aged Children (HBSC)	Slovenia	2006	5088	11–16	School-based survey	Ha, S/A, B	BMI
468	Health Behavior in School-aged Children (HBSC)	Slovenia	2010	5395	11–16	School-based survey	Ha, S/A, B	BMI
469	Health Behavior in School-aged Children (HBSC)	Slovenia	2014	4936	11–16	School-based survey	Ha, S/A, B	BMI
470	Health Behavior in School-aged Children (HBSC)	Slovenia	2018	5603	11–16	School-based survey	Ha, S/A, B	BMI
471	Health Behavior in School-aged Children (HBSC)	Spain	2001	5748	10–16	School-based survey	Ha, S/A, B	BMI
472	Health Behavior in School-aged Children (HBSC)	Spain	2006	8735	11–16	School-based survey	Ha, S/A, B	BMI
473	Health Behavior in School-aged Children (HBSC)	Spain	2010	4969	10–16	School-based survey	Ha, S/A, B	BMI
474	Health Behavior in School-aged Children (HBSC)	Spain	2014	9966	10–16	School-based survey	Ha, S/A, B	BMI
475	Health Behavior in School-aged Children (HBSC)	Spain	2018	4286	10–16	School-based survey	Ha, S/A, B	BMI
476	Health Behavior in School-aged Children (HBSC)	Sweden	2001	3748	10–16	School-based survey	Ha, S/A, B	BMI
477	Health Behavior in School-aged Children (HBSC)	Sweden	2006	4282	10–16	School-based survey	Ha, S/A, B	BMI
478	Health Behavior in School-aged Children (HBSC)	Sweden	2010	6451	10–16	School-based survey	Ha, S/A, B	BMI
479	Health Behavior in School-aged Children (HBSC)	Sweden	2014	7497	11–16	School-based survey	Ha, S/A, B	BMI
480	Health Behavior in School-aged Children (HBSC)	Sweden	2018	4031	11–16	School-based survey	Ha, S/A, B	BMI
481	Health Behavior in School-aged Children (HBSC)	Switzerland	2001	4505	11–17	School-based survey	Ha, S/A, B	BMI
482	Health Behavior in School-aged Children (HBSC)	Switzerland	2006	4462	10–16	School-based survey	Ha, S/A, B	BMI

483	Health Behavior in School-aged Children (HBSC)	Switzerland	2010	6470	10–16	School-based survey	Ha, S/A, B	BMI
484	Health Behavior in School-aged Children (HBSC)	Switzerland	2014	6399	10–16	School-based survey	Ha, S/A, B	BMI
485	Health Behavior in School-aged Children (HBSC)	Switzerland	2018	7409	10–16	School-based survey	Ha, S/A, B	BMI
486	Health Behavior in School-aged Children (HBSC)	Turkiye	2006	5268	11–16	School-based survey	Ha, S/A, B	BMI
487	Health Behavior in School-aged Children (HBSC)	Turkiye	2010	5456	11–16	School-based survey	Ha, S/A, B	BMI
488	Health Behavior in School-aged Children (HBSC)	Turkiye	2018	5654	11–16	School-based survey	Ha, S/A, B	BMI
489	Health Behavior in School-aged Children (HBSC)	Ukraine	2001	3990	11–17	School-based survey	Ha, S/A, B	BMI
490	Health Behavior in School-aged Children (HBSC)	Ukraine	2006	4837	11–16	School-based survey	Ha, S/A, B	BMI
491	Health Behavior in School-aged Children (HBSC)	Ukraine	2010	5726	11–16	School-based survey	Ha, S/A, B	BMI
492	Health Behavior in School-aged Children (HBSC)	Ukraine	2014	4312	11–16	School-based survey	Ha, S/A, B	BMI
493	Health Behavior in School-aged Children (HBSC)	Ukraine	2018	6380	10–16	School-based survey	Ha, S/A, B	BMI
494	Health Behavior in School-aged Children (HBSC)	United Kingdom	2001	13907	11–17	School-based survey	Ha, S/A, B	BMI
495	Health Behavior in School-aged Children (HBSC)	United Kingdom	2006	15021	11–17	School-based survey	Ha, S/A, B	BMI
496	Health Behavior in School-aged Children (HBSC)	United Kingdom	2010	15202	10–16	School-based survey	Ha, S/A, B	BMI
497	Health Behavior in School-aged Children (HBSC)	United Kingdom	2014	15828	11–16	School-based survey	Ha, S/A, B	BMI
498	Health Behavior in School-aged Children (HBSC)	United Kingdom	2018	23511	11–16	School-based survey	Ha, S/A, B	BMI
499	Health Behavior in School-aged Children (HBSC)	United States	1995	9611	9–18	School-based survey	Ha, S/A, B	
500	Health Behavior in School-aged Children (HBSC)	United States	1997	15558	11–17	School-based survey	Ha, S/A, B	BMI
501	Health Behavior in School-aged Children (HBSC)	United States	2001	4939	11–16	School-based survey	Ha, S/A, B	BMI
502	Health Behavior in School-aged Children (HBSC)	United States	2006	3863	11–16	School-based survey	Ha, S/A, B	BMI
503	Health Behavior in School-aged Children (HBSC)	United States	2010	6051	11–16	School-based survey	Ha, S/A, B	BMI
504	Health and Retirement Study (HRS)	United States	2004	19926	24–100	Panel	P, Ha, B	BMI
505	Health and Retirement Study (HRS)	United States	2008	830	25–100	Panel	P, Ha, B	BMI; Income
506	Health and Retirement Study (HRS)	United States	2010	6599	18–96	Panel	P, Ha, B	BMI; Income
507	Health and Retirement Study (HRS)	United States	2012	384	12–91	Panel	P, Ha, B	BMI; Income
508	Health and Retirement Study (HRS)	United States	2016	4714	21–90	Panel	P, Ha, B	BMI
509	Health and Retirement Study (HRS)	United States	2020	332	28–92	Panel	P, Ha, B	BMI
510	Health and Retirement Study (HRS)	United States	2022	2840	29–100	Panel	P, Ha, B	BMI
511	Health and Retirement Study in Thailand (HART)	Thailand	2014	7987	44–100	Panel	PI, P, Ha, N/S, C, S/A, B, K, H, Hnd, Ft	BMI; Smoking; Income
512	Healthy Aging in Africa (HAALSI)	South Africa	2014	3914	43–100	Panel	PI, P, Ha, N/S, C, S/A, B, K, H, F, Hnd, Ft, E	BMI
513	Household Income Expenditure Survey	Kiribati	2019	12481	5–80	Household survey	Ha, S/A	
514	Household Income Expenditure Survey	Tonga	2021	11061	5–80	Household survey	Ha, S/A	
515	INDEPTH	Ghana	2006	593	50–100	Surveillance/HDSS	P, B	BMI

516	INDEPTH	India	2006	373	50–90	Surveillance/HDSS	P, B	BMI
517	INDEPTH	South Africa	2006	424	50–98	Surveillance/HDSS	P, B	BMI
518	Ibadan Study on Aging	Nigeria	2003	2014	65–100	Household survey	PI, P, Ha, C, S/A, B, F	BMI
519	Imaging Genetics Study of Adolescents (IMAGEN)	Germany	2011	1207	14–18	Cohort	P, Ha, C, S/A, B	BMI
520	Indonesian Family Life Survey (IFLS)	Indonesia	2008	5269	15–97	Household panel	PI, P, Ha, N/S, B, K, H, Hnd, Ft	BMI; Smoking; Income
521	Indonesian Family Life Survey (IFLS)	Indonesia	2015	34457	14–100	Household panel	P, Ha, N/S, B, K, H, Hnd, Ft	BMI; Smoking; Income
522	Iranian Back Pain Study	Iran	2014	3761	16–90	Cross-section	PI, B	BMI
523	Japan COVID-19 and Society Internet Survey (JACSIS)	Japan	2020	8209	18–49	Cross-section	PI, P, Ha, N/S, S/A, H	BMI; Smoking; Income
524	Japan Gerontological Evaluation Study (JAGES)	Japan	2019	19181	65–95	Panel	PI, P, N/S, B, K, H, Hnd, Ft, E	BMI; Smoking; Income
525	Japan Gerontological Evaluation Study (JAGES)	Japan	2022	20712	24–100	Panel	PI, P, N/S, B, K, H, Hnd, Ft, E	Smoking; Income
526	Japan Neuropathic Pain Survey (JNeP)	Japan	2014	5553	18–89	Cross-section	PI, P, Ha, N/S, S/A, B, K, H, F, Hnd, Ft, E	Smoking
527	Japanese Chronic Pain Survey (JCP)	Japan	2014	2682	20–99	Cross-section	PI, P, Ha, N/S, S/A, B, K, H, F, Hnd, Ft, E	
528	Khayelitsha	South Africa	2004	3300	16–92	Household survey	P, Ha, C, S/A, B	BMI; Smoking; Income
529	Korean General Social Survey (KGSS)	South Korea	2010	1570	18–92	Cross-section	P, N/S, B, K, Hnd	BMI; Income
530	Korean Longitudinal Study on Aging (KLoSA)	South Korea	2005	11174	45–98	Panel	PI, P, Ha, N/S, C, S/A, B, K, H, Hnd, Ft	BMI; Smoking; Income
531	Korean National Health and Nutrition Examination Survey	South Korea	2010	6252	19–80	Household survey	P, B, K, H	BMI; Smoking; Income
532	Korean National Health and Nutrition Examination Survey	South Korea	2012	5608	19–80	Household survey	P, B, K, H	BMI; Smoking; Income
533	Korean National Health and Nutrition Examination Survey	South Korea	2013	5365	19–80	Household survey	P, B, K, H	BMI; Smoking; Income
534	Korean National Health and Nutrition Examination Survey	South Korea	2014	5033	19–80	Household survey	P, B, K, H	BMI; Smoking; Income
535	Korean National Health and Nutrition Examination Survey	South Korea	2015	5111	19–80	Household survey	P, B, K, H	BMI; Smoking; Income
536	Libyan Telephone Pain Survey	Libya	2011	1206	20–75	Household survey	P, Ha, N/S, C, S/A, B, K, H, Hnd, Ft	
537	LifeLines	Netherlands	2006	92438	5–96	Cohort	PI, P, Ha, N/S, C, S/A, B, H, F	BMI; Smoking; Income
538	Living Standards Measurement Survey (LSMS)[e]	Kazakhstan	1996	2038	5–17	Household survey	Ha	
539	Longitudinal Ageing Study in India (LASI)	India	2017	73157	18–100	Panel	P, Ha, C, B	BMI; Smoking
540	Longitudinal Study of Australian Children (LSAC)[2]	Australia	2004	3244	15–20	Cohort	P, Ha, N/S, C, S/A, B, K, H, Hnd, Ft, E	BMI; Income
541	MAmMOTH (EpiGroup)	United Kingdom	2015	17888	17–100	Cohort	P, Ha, N/S, C, S/A, B, K, H, F, Hnd, Ft, E	
542	MOBILIZE Boston Study	United States	2008	807	64–97	Cohort	P, Ha, N/S, C, S/A, B, K, H, F, Hnd, Ft, E	BMI; Smoking
543	MUSICIAN (EpiGroup)	United Kingdom	2010	15302	18–100	Cohort	PI, P, Ha, N/S, C, S/A, B, K, H, Hnd, Ft, E	BMI
544	Malaysian Aging and Retirement Study (MARS)	Malaysia	2018	5613	40–95	Cross-section	P, Ha, N/S, C, S/A, B, K, H, Hnd, Ft	BMI; Smoking; Income

545	Manicaland General Population Cohort	Zimbabwe	2014	2838	5–17	Household survey	Ha	
546	Manicaland General Population Cohort	Zimbabwe	2022	9610	15–100	Household survey	Ha, C	
547	MeMaps	Germany	2019	1562	8–17	Cohort	PI, P, Ha, C, S/A, B	BMI
548	Mexican Family Life Survey (ENNVIH)	Mexico	2002	19779	15–98	Household panel	P, Ha, C, S/A	BMI; Smoking
549	Mexican Family Life Survey (ENNVIH)	Mexico	2005	20319	14–99	Household panel	P, Ha, C, S/A	BMI; Smoking
550	Mexican Family Life Survey (ENNVIH)	Mexico	2010	23208	11–99	Household panel	P, Ha, C, S/A	BMI; Smoking
551	Mexican Health and Aging Study (MHAS)	Mexico	2001	14981	18–100	Panel	P, S/A	BMI; Income
552	Mexican Health and Aging Study (MHAS)	Mexico	2003	162	33–99	Panel	P, S/A	BMI; Income
553	Mexican Health and Aging Study (MHAS)	Mexico	2012	5716	21–100	Panel	P, S/A	BMI; Income
554	Mexican Health and Aging Study (MHAS)	Mexico	2015	499	30–87	Panel	P, S/A	BMI; Income
555	Mexican Health and Aging Study (MHAS)	Mexico	2018	4762	16–86	Panel	P, S/A	BMI; Income
556	Mexican Health and Aging Study (MHAS)	Mexico	2021	156	39–80	Panel	P, S/A	BMI; Income
557	Midlife in Japan Study (MIDJA)	Japan	2008	1025	30–79	Cohort	P, Ha, B	Smoking
558	Midlife in the United States (MIDUS)	United States	2009	6523	23–84	Panel	P, Ha, N/S, C, B, K, H	BMI; Smoking; Income
559	Murakami Cohort Study[f]	Japan	2012	14205	42–72	Cohort	P, Ha, N/S, B, K, Hnd, E	
560	National Childhood Development Study (NCDS)	United Kingdom	2002	8499	44–46	Cohort	P, Ha, N/S, C, S/A, B, K, H, Hnd, Ft	BMI; Smoking
561	National Health Interview Survey (NHIS)	United States	1997	36051	18–85	Repeat cross-section	P, Ha, N/S, B, K, H, F, Hnd, Ft, E	BMI; Smoking; Income
562	National Health Interview Survey (NHIS)	United States	1998	32407	18–85	Repeat cross-section	P, Ha, N/S, B, K, H, F, Hnd, Ft, E	BMI; Smoking; Income
563	National Health Interview Survey (NHIS)	United States	1999	30791	18–85	Repeat cross-section	P, Ha, N/S, B, K, H, F, Hnd, Ft, E	BMI; Smoking; Income
564	National Health Interview Survey (NHIS)	United States	2000	32347	18–85	Repeat cross-section	P, Ha, N/S, B, K, H, F, Hnd, Ft, E	BMI; Smoking; Income
565	National Health Interview Survey (NHIS)	United States	2001	33310	18–85	Repeat cross-section	P, Ha, N/S, B, K, H, F, Hnd, Ft, E	BMI; Smoking; Income
566	National Health Interview Survey (NHIS)	United States	2002	31008	18–85	Repeat cross-section	PI, P, Ha, N/S, B, K, H, F, Hnd, Ft, E	BMI; Smoking; Income
567	National Health Interview Survey (NHIS)	United States	2003	30824	18–85	Repeat cross-section	PI, P, Ha, N/S, B, K, H, F, Hnd, Ft, E	BMI; Smoking; Income
568	National Health Interview Survey (NHIS)	United States	2004	31282	18–85	Repeat cross-section	P, Ha, N/S, B, K, H, F, Hnd, Ft, E	BMI; Smoking; Income
569	National Health Interview Survey (NHIS)	United States	2005	31409	18–85	Repeat cross-section	P, Ha, N/S, B, K, H, F, Hnd, Ft, E	BMI; Smoking; Income
570	National Health Interview Survey (NHIS)	United States	2006	24244	18–85	Repeat cross-section	PI, P, Ha, N/S, B, K, H, F, Hnd, Ft, E	BMI; Smoking; Income
571	National Health Interview Survey (NHIS)	United States	2007	23381	18–85	Repeat cross-section	P, Ha, N/S, B, K, H, F, Hnd, Ft, E	BMI; Smoking; Income
572	National Health Interview Survey (NHIS)	United States	2008	21777	18–85	Repeat cross-section	P, Ha, N/S, B, K, H, F, Hnd, Ft, E	BMI; Smoking; Income
573	National Health Interview Survey (NHIS)	United States	2009	27721	18–85	Repeat cross-section	PI, P, Ha, N/S, B, K, H, F, Hnd, Ft, E	BMI; Smoking; Income
574	National Health Interview Survey (NHIS)	United States	2010	27152	18–85	Repeat cross-section	PI, P, Ha, N/S, B, K, H, F, Hnd, Ft, E	BMI; Smoking; Income

575	National Health Interview Survey (NHIS)	United States	2011	33002	18–85	Repeat cross-section	P, Ha, N/S, B, K, H, F, Hnd, Ft, E	BMI; Smoking; Income
576	National Health Interview Survey (NHIS)	United States	2012	34514	18–85	Repeat cross-section	P, Ha, N/S, B, K, H, F, Hnd, Ft, E	BMI; Smoking; Income
577	National Health Interview Survey (NHIS)	United States	2013	34542	18–85	Repeat cross-section	P, Ha, N/S, B, K, H, F, Hnd, Ft, E	BMI; Smoking; Income
578	National Health Interview Survey (NHIS)	United States	2014	36683	18–85	Repeat cross-section	P, Ha, N/S, B, K, H, F, Hnd, Ft, E	BMI; Smoking; Income
579	National Health Interview Survey (NHIS)	United States	2015	33655	18–85	Repeat cross-section	P, Ha, N/S, B, K, H, F, Hnd, Ft, E	BMI; Smoking; Income
580	National Health Interview Survey (NHIS)	United States	2016	33018	18–85	Repeat cross-section	P, Ha, N/S, B, K, H, F, Hnd, Ft, E	BMI; Smoking; Income
581	National Health Interview Survey (NHIS)	United States	2017	26735	18–85	Repeat cross-section	P, Ha, N/S, B, K, H, F, Hnd, Ft, E	BMI; Smoking; Income
582	National Health Interview Survey (NHIS)	United States	2018	25407	18–85	Repeat cross-section	P, Ha, N/S, B, K, H, F, Hnd, Ft, E	BMI; Smoking; Income
583	National Health Interview Survey (NHIS)	United States	2019	31200	18–85	Repeat cross-section	PI, P, Ha, S/A, B, F	Smoking
584	National Health Interview Survey (NHIS)	United States	2021	28671	18–85	Repeat cross-section	P, Ha, S/A, B, F	Smoking
585	National Health Interview Survey (NHIS)	United States	2023	28512	18–85	Repeat cross-section	P, Ha, S/A, B, F	Smoking
586	National Health Measurement Survey 2010 (NHMS)	United States	2008	3844	35–89	Cross-section	P, Ha, C, S/A, B, H, F	BMI; Smoking; Income
587	National Health and Aging Trends Study (NHATS)	United States	2011	17285	67–92	Panel	P, Ha, N/S, S/A, B, K, H, Hnd, Ft	BMI; Smoking; Income
588	National Health and Nutrition Examination Survey (NHANES)	United States	1999	4878	20–85	Repeated cross-section	P, Ha, N/S, S/A, B, K, H, F, Hnd, Ft	BMI; Smoking; Income
589	National Health and Nutrition Examination Survey (NHANES)	United States	2001	5410	20–85	Repeated cross-section	P, Ha, N/S, C, S/A, B, K, H, F, Hnd, Ft	BMI; Smoking; Income
590	National Health and Nutrition Examination Survey (NHANES)	United States	2003	5040	20–85	Repeated cross-section	P, Ha, N/S, C, S/A, B, K, H, F, Hnd, Ft	BMI; Smoking; Income
591	National Health and Nutrition Examination Survey (NHANES)	United States	2009	5106	20–69	Repeated cross-section	P, N/S, C, B, H	BMI; Smoking; Income
592	National Income Dynamics Study (NIDS)	South Africa	2008	676	15–100	Panel	P, Ha, C, S/A, B	BMI; Smoking; Income
593	National Income Dynamics Study (NIDS)	South Africa	2010	1752	15–100	Panel	P, Ha, C, S/A, B	BMI; Smoking; Income
594	National Income Dynamics Study (NIDS)	South Africa	2012	2552	15–100	Panel	P, Ha, C, S/A, B	BMI; Smoking; Income
595	National Income Dynamics Study (NIDS)	South Africa	2014	4111	15–100	Panel	P, Ha, C, B	BMI; Smoking; Income
596	National Income Dynamics Study (NIDS)	South Africa	2017	23869	15–100	Panel	P, Ha, C, B	BMI; Smoking; Income
597	National Social Life, Health, and Aging Project (NSHAP)	United States	2010	2600	62–91	Panel	P, Ha, N/S, C, S/A, B, Hnd, Ft	BMI; Smoking; Income
598	National Survey of Health and Nutrition in Mexico 2000 (ENSANUT)	Mexico	2000	44095	20–100	Household survey	P, Ha, N/S, K, H, Hnd, Ft, E	BMI; Smoking
599	National Survey of Self-Care and Aging (NSSA)	United States	1994	3482	68–100	Household survey	P, N/S, S/A, B, K, H, Hnd, Ft, E	Smoking; Income
600	New Zealand Health Survey (NZHS)[f]	New Zealand	2017	13598	20–85	Household survey	P, Ha, C, S/A, B	
601	Ontario Health Study (OHS)	Canada	2015	20051	18–100	Cohort	P, N/S, B, K, H, F, Hnd, Ft, E	BMI; Smoking; Income
602	Orofacial Pain: Prospective Evaluation and Risk Assessment (OPPERA)	United States	2015	655	22–53	Cohort	P, N/S, C, B, K, F, Hnd, Ft, E	

603	PREVENT-AD	Canada	2021	339	56–88	Cohort	PI, P, Ha, N/S, C, S/A, B, K, H, Hnd, Ft, E	Smoking
604	Pain in Schoolchildren Study (PSC)	United Kingdom	2003	1442	11–16	School-based Survey	P, Ha, N/S, C, S/A, B, K, H, Hnd, Ft, E	BMI
605	Pain-Associated Cross-Sectional Epidemiological (PACE) Study[3]	Japan	2009	5000	20–79	Cross-section	PI, P, Ha, N/S, S/A, B, K, H, F, Hnd, Ft, E	
606	Puerto Rican Elderly: Health Conditions Project (PREHCO)	Puerto Rico	2002	3713	60–100	Household survey	P, C, B	BMI; Smoking; Income
607	SHAMA (EpiGroup)	United Kingdom	2015	1604	30–90	Cross-section	PI, P, Ha, N/S, C, S/A, B, K, H, F, Hnd, Ft, E	
608	Sacramento Area Latino Study on Aging (SALSA)	United States	1996	1716	59–93	Cohort	P, C, B, K, H, Hnd, Ft	BMI; Smoking; Income
609	Saudi Online Pain Survey	Saudi Arabia	2018	26372	24–65	Household survey	PI, P, Ha, N/S, C, S/A, B	Income
610	Scottish Family Health Study (SFHS)	United Kingdom	2008	24073	18–100	Cohort	PI, P, Ha, N/S, C, S/A, B	BMI; Smoking; Income
611	Scottish Health Study (SHS)	United Kingdom	2010	4656	16–95	Household survey	P, C	BMI; Smoking; Income
612	Scottish Health Study (SHS)	United Kingdom	2022	4732	12–97	Household survey	P, Ha, N/S, C, S/A, B	BMI; Smoking; Income
613	Shandong Adolescent Behavior & Health Cohort (SABHC)	China	2015	6571	10–20	School-based survey	P, Ha, S/A	BMI; Income
614	South African National Demographic Survey 2016 (SADS)	South Africa	2016	10336	15–95	Household survey	P, Ha, N/S, C, S/A, B, F	Smoking
615	Spanish Chronic Pain Survey	Spain	2013	1957	18–92	Household survey	P, Ha, N/S, B	
616	The Spanish Longitudinal Study on Ageing and Health (SLAS)	Spain	2011	2378	21–100	Cohort	P, B	BMI; Smoking; Income
617	Study of Health and Aging in Europe (SHARE)	Austria	2019	1014	45–100	Panel	P, B, K, H, F	BMI; Smoking; Income
618	Study of Health and Aging in Europe (SHARE)	Austria	2020	938	44–98	Panel	P, B, K, H, F	BMI; Smoking; Income
619	Study of Health and Aging in Europe (SHARE)	Austria	2021	27	56–92	Panel	P, B, K, H, F	BMI; Smoking; Income
620	Study of Health and Aging in Europe (SHARE)	Austria	2022	1959	40–99	Panel	P, B, K, H, F	BMI; Smoking; Income
621	Study of Health and Aging in Europe (SHARE)	Belgium	2019	480	46–97	Panel	P, B, K, H, F	BMI; Smoking; Income
622	Study of Health and Aging in Europe (SHARE)	Belgium	2020	1790	36–99	Panel	P, B, K, H, F	BMI; Smoking; Income
623	Study of Health and Aging in Europe (SHARE)	Belgium	2021	222	47–91	Panel	P, B, K, H, F	BMI; Smoking; Income
624	Study of Health and Aging in Europe (SHARE)	Belgium	2022	2439	31–100	Panel	P, B, K, H, F	BMI; Smoking; Income
625	Study of Health and Aging in Europe (SHARE)	Bulgaria	2019	311	42–92	Panel	P, B, K, H, F	BMI; Smoking; Income
626	Study of Health and Aging in Europe (SHARE)	Bulgaria	2020	593	40–93	Panel	P, B, K, H, F	BMI; Smoking; Income
627	Study of Health and Aging in Europe (SHARE)	Bulgaria	2021	10	52–88	Panel	P, B, K, H, F	BMI; Smoking; Income
628	Study of Health and Aging in Europe (SHARE)	Bulgaria	2022	159	49–100	Panel	P, B, K, H, F	BMI; Smoking; Income
629	Study of Health and Aging in Europe (SHARE)	Croatia	2019	319	34–95	Panel	P, B, K, H, F	BMI; Smoking; Income
630	Study of Health and Aging in Europe (SHARE)	Croatia	2020	1703	32–98	Panel	P, B, K, H, F	BMI; Smoking; Income

631	Study of Health and Aging in Europe (SHARE)	Croatia	2021	195	53–94	Panel	P, B, K, H, F	BMI; Smoking; Income
632	Study of Health and Aging in Europe (SHARE)	Croatia	2022	2749	36–100	Panel	P, B, K, H, F	BMI; Smoking; Income
633	Study of Health and Aging in Europe (SHARE)	Cyprus	2019	162	50–95	Panel	P, B, K, H, F	BMI; Smoking; Income
634	Study of Health and Aging in Europe (SHARE)	Cyprus	2020	372	51–99	Panel	P, B, K, H, F	BMI; Smoking; Income
635	Study of Health and Aging in Europe (SHARE)	Cyprus	2021	97	27–90	Panel	P, B, K, H, F	BMI; Smoking; Income
636	Study of Health and Aging in Europe (SHARE)	Cyprus	2022	295	46–98	Panel	P, B, K, H, F	BMI; Smoking; Income
637	Study of Health and Aging in Europe (SHARE)	Czech Republic	2019	1785	30–97	Panel	P, B, K, H, F	BMI; Smoking; Income
638	Study of Health and Aging in Europe (SHARE)	Czech Republic	2020	1246	38–96	Panel	P, B, K, H, F	BMI; Smoking; Income
639	Study of Health and Aging in Europe (SHARE)	Czech Republic	2021	41	54–93	Panel	P, B, K, H, F	BMI; Smoking; Income
640	Study of Health and Aging in Europe (SHARE)	Czech Republic	2022	1224	30–96	Panel	P, B, K, H, F	BMI; Smoking; Income
641	Study of Health and Aging in Europe (SHARE)	Denmark	2019	581	37–100	Panel	P, B, K, H, F	BMI; Smoking; Income
642	Study of Health and Aging in Europe (SHARE)	Denmark	2020	1783	40–98	Panel	P, B, K, H, F	BMI; Smoking; Income
643	Study of Health and Aging in Europe (SHARE)	Denmark	2021	40	54–88	Panel	P, B, K, H, F	BMI; Smoking; Income
644	Study of Health and Aging in Europe (SHARE)	Denmark	2022	625	43–97	Panel	P, B, K, H, F	BMI; Smoking; Income
645	Study of Health and Aging in Europe (SHARE)	Estonia	2019	1396	35–95	Panel	P, B, K, H, F	BMI; Smoking; Income
646	Study of Health and Aging in Europe (SHARE)	Estonia	2020	1998	40–100	Panel	P, B, K, H, F	BMI; Smoking; Income
647	Study of Health and Aging in Europe (SHARE)	Estonia	2021	370	43–95	Panel	P, B, K, H, F	BMI; Smoking; Income
648	Study of Health and Aging in Europe (SHARE)	Estonia	2022	1372	40–97	Panel	P, B, K, H, F	BMI; Smoking; Income
649	Study of Health and Aging in Europe (SHARE)	Finland	2019	790	37–96	Panel	P, B, K, H, F	BMI; Smoking; Income
650	Study of Health and Aging in Europe (SHARE)	Finland	2020	370	42–92	Panel	P, B, K, H, F	BMI; Smoking; Income
651	Study of Health and Aging in Europe (SHARE)	Finland	2022	942	31–99	Panel	P, B, K, H, F	BMI; Smoking; Income
652	Study of Health and Aging in Europe (SHARE)	France	2019	1616	39–99	Panel	P, B, K, H, F	BMI; Smoking; Income
653	Study of Health and Aging in Europe (SHARE)	France	2020	1392	39–100	Panel	P, B, K, H, F	BMI; Smoking; Income
654	Study of Health and Aging in Europe (SHARE)	France	2021	65	52–96	Panel	P, B, K, H, F	BMI; Smoking; Income
655	Study of Health and Aging in Europe (SHARE)	France	2022	715	40–99	Panel	P, B, K, H, F	BMI; Smoking; Income
656	Study of Health and Aging in Europe (SHARE)	Germany	2019	1858	47–97	Panel	P, B, K, H, F	BMI; Smoking; Income
657	Study of Health and Aging in Europe (SHARE)	Germany	2020	2004	28–95	Panel	P, B, K, H, F	BMI; Smoking; Income

658	Study of Health and Aging in Europe (SHARE)	Germany	2021	84	46–93	Panel	P, B, K, H, F	BMI; Smoking; Income
659	Study of Health and Aging in Europe (SHARE)	Germany	2022	1547	38–97	Panel	P, B, K, H, F	BMI; Smoking; Income
660	Study of Health and Aging in Europe (SHARE)	Greece	2019	1016	46–96	Panel	P, B, K, H, F	BMI; Smoking; Income
661	Study of Health and Aging in Europe (SHARE)	Greece	2020	1979	42–98	Panel	P, B, K, H, F	BMI; Smoking; Income
662	Study of Health and Aging in Europe (SHARE)	Greece	2021	93	57–97	Panel	P, B, K, H, F	BMI; Smoking; Income
663	Study of Health and Aging in Europe (SHARE)	Greece	2022	600	45–95	Panel	P, B, K, H, F	BMI; Smoking; Income
664	Study of Health and Aging in Europe (SHARE)	Hungary	2019	107	50–94	Panel	P, B, K, H, F	BMI; Smoking; Income
665	Study of Health and Aging in Europe (SHARE)	Hungary	2020	1102	39–94	Panel	P, B, K, H, F	BMI; Smoking; Income
666	Study of Health and Aging in Europe (SHARE)	Hungary	2021	112	50–95	Panel	P, B, K, H, F	BMI; Smoking; Income
667	Study of Health and Aging in Europe (SHARE)	Hungary	2022	828	21–98	Panel	P, B, K, H, F	BMI; Smoking; Income
668	Study of Health and Aging in Europe (SHARE)	Israel	2019	637	44–100	Panel	P, B, K, H, F	BMI; Smoking; Income
669	Study of Health and Aging in Europe (SHARE)	Israel	2020	763	46–100	Panel	P, B, K, H, F	BMI; Smoking; Income
670	Study of Health and Aging in Europe (SHARE)	Israel	2022	160	46–95	Panel	P, B, K, H, F	BMI; Smoking; Income
671	Study of Health and Aging in Europe (SHARE)	Italy	2019	798	40–100	Panel	P, B, K, H, F	BMI; Smoking; Income
672	Study of Health and Aging in Europe (SHARE)	Italy	2020	1366	46–100	Panel	P, B, K, H, F	BMI; Smoking; Income
673	Study of Health and Aging in Europe (SHARE)	Italy	2021	114	49–92	Panel	P, B, K, H, F	BMI; Smoking; Income
674	Study of Health and Aging in Europe (SHARE)	Italy	2022	1671	43–98	Panel	P, B, K, H, F	BMI; Smoking; Income
675	Study of Health and Aging in Europe (SHARE)	Latvia	2019	433	39–100	Panel	P, B, K, H, F	BMI; Smoking; Income
676	Study of Health and Aging in Europe (SHARE)	Latvia	2020	785	34–99	Panel	P, B, K, H, F	BMI; Smoking; Income
677	Study of Health and Aging in Europe (SHARE)	Latvia	2022	718	33–92	Panel	P, B, K, H, F	BMI; Smoking; Income
678	Study of Health and Aging in Europe (SHARE)	Lithuania	2019	812	45–94	Panel	P, B, K, H, F	BMI; Smoking; Income
679	Study of Health and Aging in Europe (SHARE)	Lithuania	2020	623	48–96	Panel	P, B, K, H, F	BMI; Smoking; Income
680	Study of Health and Aging in Europe (SHARE)	Lithuania	2021	111	45–93	Panel	P, B, K, H, F	BMI; Smoking; Income
681	Study of Health and Aging in Europe (SHARE)	Lithuania	2022	109	39–93	Panel	P, B, K, H, F	BMI; Smoking; Income
682	Study of Health and Aging in Europe (SHARE)	Luxembourg	2019	474	47–100	Panel	P, B, K, H, F	BMI; Smoking; Income
683	Study of Health and Aging in Europe (SHARE)	Luxembourg	2020	474	38–97	Panel	P, B, K, H, F	BMI; Smoking; Income
684	Study of Health and Aging in Europe (SHARE)	Luxembourg	2021	7	61–79	Panel	P, B, K, H, F	BMI; Smoking; Income

685	Study of Health and Aging in Europe (SHARE)	Luxembourg	2022	147	58–93	Panel	P, B, K, H, F	BMI; Smoking; Income
686	Study of Health and Aging in Europe (SHARE)	Malta	2019	408	48–95	Panel	P, B, K, H, F	BMI; Smoking; Income
687	Study of Health and Aging in Europe (SHARE)	Malta	2020	396	48–97	Panel	P, B, K, H, F	BMI; Smoking; Income
688	Study of Health and Aging in Europe (SHARE)	Malta	2021	22	56–90	Panel	P, B, K, H, F	BMI; Smoking; Income
689	Study of Health and Aging in Europe (SHARE)	Malta	2022	217	52–95	Panel	P, B, K, H, F	BMI; Smoking; Income
690	Study of Health and Aging in Europe (SHARE)	Netherlands	2019	1035	50–96	Panel	P, B, K, H, F	BMI; Smoking; Income
691	Study of Health and Aging in Europe (SHARE)	Netherlands	2020	898	40–97	Panel	P, B, K, H, F	BMI; Smoking; Income
692	Study of Health and Aging in Europe (SHARE)	Netherlands	2021	189	55–91	Panel	P, B, K, H, F	BMI; Smoking; Income
693	Study of Health and Aging in Europe (SHARE)	Netherlands	2022	480	51–99	Panel	P, B, K, H, F	BMI; Smoking; Income
694	Study of Health and Aging in Europe (SHARE)	Poland	2019	783	45–98	Panel	P, B, K, H, F	BMI; Smoking; Income
695	Study of Health and Aging in Europe (SHARE)	Poland	2020	2064	34–100	Panel	P, B, K, H, F	BMI; Smoking; Income
696	Study of Health and Aging in Europe (SHARE)	Poland	2021	11	57–80	Panel	P, B, K, H, F	BMI; Smoking; Income
697	Study of Health and Aging in Europe (SHARE)	Poland	2022	2558	35–98	Panel	P, B, K, H, F	BMI; Smoking; Income
698	Study of Health and Aging in Europe (SHARE)	Portugal	2022	1632	40–99	Panel	P, B, K, H, F	BMI; Smoking; Income
699	Study of Health and Aging in Europe (SHARE)	Romania	2019	210	42–86	Panel	P, B, K, H, F	BMI; Smoking; Income
700	Study of Health and Aging in Europe (SHARE)	Romania	2020	1069	40–94	Panel	P, B, K, H, F	BMI; Smoking; Income
701	Study of Health and Aging in Europe (SHARE)	Romania	2021	42	52–93	Panel	P, B, K, H, F	BMI; Smoking; Income
702	Study of Health and Aging in Europe (SHARE)	Romania	2022	361	50–93	Panel	P, B, K, H, F	BMI; Smoking; Income
703	Study of Health and Aging in Europe (SHARE)	Slovakia	2019	425	32–100	Panel	P, B, K, H, F	BMI; Smoking; Income
704	Study of Health and Aging in Europe (SHARE)	Slovakia	2020	572	44–92	Panel	P, B, K, H, F	BMI; Smoking; Income
705	Study of Health and Aging in Europe (SHARE)	Slovakia	2021	76	49–86	Panel	P, B, K, H, F	BMI; Smoking; Income
706	Study of Health and Aging in Europe (SHARE)	Slovakia	2022	79	53–80	Panel	P, B, K, H, F	BMI; Smoking; Income
707	Study of Health and Aging in Europe (SHARE)	Slovenia	2019	886	44–100	Panel	P, B, K, H, F	BMI; Smoking; Income
708	Study of Health and Aging in Europe (SHARE)	Slovenia	2020	2150	40–100	Panel	P, B, K, H, F	BMI; Smoking; Income
709	Study of Health and Aging in Europe (SHARE)	Slovenia	2021	243	54–99	Panel	P, B, K, H, F	BMI; Smoking; Income
710	Study of Health and Aging in Europe (SHARE)	Slovenia	2022	1667	38–100	Panel	P, B, K, H, F	BMI; Smoking; Income
711	Study of Health and Aging in Europe (SHARE)	Spain	2019	1094	51–99	Panel	P, B, K, H, F	BMI; Smoking; Income

712	Study of Health and Aging in Europe (SHARE)	Spain	2020	1017	37–100	Panel	P, B, K, H, F	BMI; Smoking; Income
713	Study of Health and Aging in Europe (SHARE)	Spain	2021	318	30–97	Panel	P, B, K, H, F	BMI; Smoking; Income
714	Study of Health and Aging in Europe (SHARE)	Spain	2022	814	40–97	Panel	P, B, K, H, F	BMI; Smoking; Income
715	Study of Health and Aging in Europe (SHARE)	Sweden	2019	1754	37–99	Panel	P, B, K, H, F	BMI; Smoking; Income
716	Study of Health and Aging in Europe (SHARE)	Sweden	2020	778	38–99	Panel	P, B, K, H, F	BMI; Smoking; Income
717	Study of Health and Aging in Europe (SHARE)	Sweden	2021	95	55–93	Panel	P, B, K, H, F	BMI; Smoking; Income
718	Study of Health and Aging in Europe (SHARE)	Sweden	2022	383	47–100	Panel	P, B, K, H, F	BMI; Smoking; Income
719	Study of Health and Aging in Europe (SHARE)	Switzerland	2019	1353	44–98	Panel	P, B, K, H, F	BMI; Smoking; Income
720	Study of Health and Aging in Europe (SHARE)	Switzerland	2020	735	35–100	Panel	P, B, K, H, F	BMI; Smoking; Income
721	Study of Health and Aging in Europe (SHARE)	Switzerland	2021	40	60–90	Panel	P, B, K, H, F	BMI; Smoking; Income
722	Study of Health and Aging in Europe (SHARE)	Switzerland	2022	216	51–94	Panel	P, B, K, H, F	BMI; Smoking; Income
723	Study on Nutrition and Cardiovascular Risk in Spain (ENRICA)	Spain	2010	2451	62–97	Household survey	PI, P, B	
724	Survey on Aging in Latin America and Caribbean (SABE)	Argentina	1999	1041	60–94	Household survey	P, Ha, C, B	Smoking
725	Survey on Aging in Latin America and Caribbean (SABE)	Barbados	1999	1508	60–97	Household survey	P, Ha, C, B	BMI; Smoking
726	Survey on Aging in Latin America and Caribbean (SABE)	Brazil	1999	2143	60–100	Household survey	P, Ha, C, B	BMI; Smoking
727	Survey on Aging in Latin America and Caribbean (SABE)	Chile	1999	1300	60–99	Household survey	P, Ha, C, B	BMI; Smoking
728	Survey on Aging in Latin America and Caribbean (SABE)	Cuba	1999	1905	60–100	Household survey	P, Ha, C, B	Smoking
729	Survey on Aging in Latin America and Caribbean (SABE)	Mexico	1999	1870	50–98	Household survey	P, Ha, C, B	BMI; Smoking
730	Survey on Aging in Latin America and Caribbean (SABE)	Uruguay	1999	1449	60–97	Household survey	P, Ha, C, B	BMI; Smoking
731	Survey on Social, Health and Overall Wellbeing of Older People (SSHW)	Kenya	2008	2585	44–100	Household survey	P, B	
732	Swedish Health Assets Project 2008 (SHA)	Sweden	2008	7835	19–64	Household survey	P, Ha, N/S, C, S/A, B	BMI; Income
733	Swiss Health Panel (SHP)	Switzerland	2000	7074	13–92	Panel	Ha, B	Income
734	Swiss Health Panel (SHP)	Switzerland	2001	659	13–86	Panel	Ha, B	Income
735	Swiss Health Panel (SHP)	Switzerland	2002	252	13–85	Panel	Ha, B	Income
736	Swiss Health Panel (SHP)	Switzerland	2003	211	13–79	Panel	Ha, B	Income
737	Swiss Health Panel (SHP)	Switzerland	2004	3879	13–94	Panel	Ha, B	BMI; Income
738	Swiss Health Panel (SHP)	Switzerland	2005	381	13–85	Panel	Ha, B	BMI; Income
739	Swiss Health Panel (SHP)	Switzerland	2006	413	14–84	Panel	Ha, B	BMI; Income
740	Swiss Health Panel (SHP)	Switzerland	2007	526	14–86	Panel	Ha, B	BMI; Income

741	Swiss Health Panel (SHP)	Switzerland	2008	398	14–84	Panel	Ha, B	BMI; Income
742	Swiss Health Panel (SHP)	Switzerland	2009	365	14–86	Panel	Ha, B	BMI; Income
743	Swiss Health Panel (SHP)	Switzerland	2010	377	14–92	Panel	Ha, B	BMI; Smoking; Income
744	Swiss Health Panel (SHP)	Switzerland	2011	284	14–82	Panel	Ha, B	BMI; Smoking; Income
745	Swiss Health Panel (SHP)	Switzerland	2012	274	14–82	Panel	Ha, B	BMI; Income
746	Swiss Health Panel (SHP)	Switzerland	2013	242	14–78	Panel	Ha, B	BMI; Income
747	Swiss Health Panel (SHP)	Switzerland	2014	5451	14–96	Panel	Ha, B	BMI; Smoking; Income
748	Swiss Health Panel (SHP)	Switzerland	2015	479	14–95	Panel	Ha, B	BMI; Smoking; Income
749	Swiss Health Panel (SHP)	Switzerland	2016	415	13–94	Panel	Ha, B	BMI; Smoking; Income
750	Swiss Health Panel (SHP)	Switzerland	2017	333	14–88	Panel	Ha, B	BMI; Smoking; Income
751	Swiss Health Panel (SHP)	Switzerland	2018	326	14–87	Panel	Ha, B	BMI; Smoking; Income
752	Swiss Health Panel (SHP)	Switzerland	2019	289	14–88	Panel	Ha, B	BMI; Smoking; Income
753	Swiss Health Panel (SHP)	Switzerland	2020	7712	14–100	Panel	Ha, B	BMI; Smoking; Income
754	Swiss Health Panel (SHP)	Switzerland	2021	586	14–92	Panel	Ha, B	BMI; Smoking; Income
755	Swiss Health Panel (SHP)	Switzerland	2022	302	14–92	Panel	Ha, B	BMI; Smoking; Income
756	Swiss Health Panel (SHP)	Switzerland	2023	327	14–81	Panel	Ha, B	BMI; Smoking; Income
757	Taiwan MJ Biobank (MJH)	Taiwan	1996	58985	5–96	Biobank/Repeat cross-section	S/A, B	Smoking
758	Taiwan MJ Biobank (MJH)	Taiwan	1997	74029	5–97	Biobank/Repeat cross-section	S/A, B	Smoking
759	Taiwan MJ Biobank (MJH)	Taiwan	1998	73397	5–98	Biobank/Repeat cross-section	S/A, B	Smoking
760	Taiwan MJ Biobank (MJH)	Taiwan	1999	68439	5–99	Biobank/Repeat cross-section	B	Smoking
761	Taiwan MJ Biobank (MJH)	Taiwan	2000	73149	5–100	Biobank/Repeat cross-section	B	Smoking
762	Taiwan MJ Biobank (MJH)	Taiwan	2001	66810	5–98	Biobank/Repeat cross-section	B	Smoking
763	Taiwan MJ Biobank (MJH)	Taiwan	2002	68353	5–100	Biobank/Repeat cross-section	B	Smoking
764	Taiwan MJ Biobank (MJH)	Taiwan	2003	231	6–86	Biobank/Repeat cross-section	B	Smoking
765	Taiwan MJ Biobank (MJH)	Taiwan	2014	28731	7–95	Biobank/Repeat cross-section	P	Smoking
766	Taiwan MJ Biobank (MJH)	Taiwan	2015	31167	6–96	Biobank/Repeat cross-section	P	Smoking
767	Taiwan MJ Biobank (MJH)	Taiwan	2016	25817	8–95	Biobank/Repeat cross-section	P	Smoking
768	Taiwan MJ Biobank (MJH)	Taiwan	2017	26952	7–92	Biobank/Repeat cross-section	P, N/S, B, K, H, E	Smoking

769	Taiwan MJ Biobank (MJH)	Taiwan	2018	23009	8–93	Biobank/Repeat cross-section	P, N/S, B, K, H, E	Smoking
770	Taiwan MJ Biobank (MJH)	Taiwan	2019	22646	9–98	Biobank/Repeat cross-section	P, N/S, B, K, H, E	Smoking
771	Taiwan MJ Biobank (MJH)	Taiwan	2020	18553	6–95	Biobank/Repeat cross-section	P, N/S, B, K, H, E	Smoking
772	Taiwan MJ Biobank (MJH)	Taiwan	2021	13414	7–95	Biobank/Repeat cross-section	P, N/S, B, K, H, E	Smoking
773	Taiwan MJ Biobank (MJH)	Taiwan	2022	13316	9–92	Biobank/Repeat cross-section	P, N/S, B, K, H, E	Smoking
774	Thai Students Survey	Thailand	2009	2750	9–19	School-based survey	PI, P, Ha, N/S, B, K, H, Hnd, Ft, E	BMI
775	The Filipino American Community Epidemiological Study (FACES)	United States	1996	2297	7–65	Cross-section	Ha, C, B	BMI
776	The Japan Society and New Tobacco Internet Survey (JASTIS)	Japan	2025	28000	15–84	Panel	PI, P, Ha, N/S, C, S/A, B, H	BMI; Smoking; Income
777	The Longitudinal Study of Ageing and Health in the Philippines (LSAHP)	Philippines	2018	4411	65–85	Household survey	P, Ha, N/S, B, K, H, Ft	Smoking; Income
778	The National Longitudinal Study of Adolescent to Adult Health (ADD Health)	United States	1995	6496	12–21	Panel	P, Ha, C, S/A	BMI; Smoking; Income
779	The Netherlands Study of Depression and Anxiety (NESDA)[c]	Netherlands	2004	652	18–65	Cohort	PI, P, Ha, C, S/A, B, F	BMI; Smoking
780	The Trøndelag Health Study (HUNT Study)	Norway	1996	28357	19–100	Cohort	P, N/S, C, B, K, H, Hnd, Ft, E	BMI; Smoking
781	The Trøndelag Health Study (HUNT Study)	Norway	2007	25275	13–97	Cohort	P, Ha, N/S, C, S/A, B, K, H, Hnd, Ft, E	BMI; Smoking
782	The Trøndelag Health Study (HUNT Study)	Norway	2018	48965	13–100	Cohort	P, Ha, N/S, C, S/A, B, K, H, F, Hnd, Ft, E	BMI; Smoking; Income
783	UK Biobank (UKB)	United Kingdom	2008	453392	37–74	Cohort	P, Ha, N/S, C, S/A, B, K, H, F	BMI; Smoking; Income
784	UK Biobank (UKB)	United Kingdom	2016	48990	45–83	Cohort	P, Ha, N/S, C, S/A, B, K, H, F	BMI; Smoking; Income
785	UK Biobank (UKB)[d]	United Kingdom	2020	167225	49–84	Cohort	P, Hnd, Ft	
786	Understanding America Survey (UAS)	United States	2015	231	18–88	Panel	P, Ha, B	BMI; Income
787	Understanding America Survey (UAS)	United States	2016	560	18–91	Panel	P, Ha, B	BMI; Income
788	Understanding America Survey (UAS)	United States	2017	303	18–95	Panel	P, Ha, B	BMI; Income
789	Understanding America Survey (UAS)	United States	2018	504	18–89	Panel	P, Ha, B	BMI; Income
790	Understanding America Survey (UAS)	United States	2019	628	17–100	Panel	P, Ha, B	BMI; Income
791	Understanding America Survey (UAS)	United States	2020	851	18–91	Panel	P, Ha, B	BMI; Income
792	Understanding America Survey (UAS)	United States	2021	694	18–100	Panel	P, Ha, B	BMI; Income
793	Understanding America Survey (UAS)	United States	2022	882	18–100	Panel	P, Ha, B	BMI; Income
794	Understanding America Survey (UAS)	United States	2023	4126	18–100	Panel	P, Ha, B	BMI; Income
795	Understanding America Survey (UAS)	United States	2024	5760	18–97	Panel	P, Ha, B	BMI; Income
796	Understanding America Survey (UAS)	United States	2025	3303	18–90	Panel	P, Ha, B	BMI; Income
797	WHEST (EpiGroup)	United Kingdom	2015	2088	25–94	Cohort	PI, P, Ha, N/S, C, S/A, B, K, H, Hnd, Ft, E	Income
798	WHO Multi-Country Survey (MCSS)	China	2000	9434	18–92	Household survey	P, C, B	BMI; Income

799	WHO Multi-Country Survey (MCSS)	Colombia	2000	6015	18–99	Household survey	P, C, B	BMI; Income
800	WHO Multi-Country Survey (MCSS)	Egypt	2000	4471	18–95	Household survey	P, C, B	BMI; Income
801	WHO Multi-Country Survey (MCSS)	Georgia	2000	9837	18–100	Household survey	P, C, B	BMI; Income
802	WHO Multi-Country Survey (MCSS)	India	2000	5178	18–100	Household survey	P, C, B	BMI; Income
803	WHO Multi-Country Survey (MCSS)	Indonesia	2000	9927	17–100	Household survey	P, C, B	BMI; Income
804	WHO Multi-Country Survey (MCSS)	Iran	2000	9555	18–100	Household survey	P, C, B	BMI; Income
805	WHO Multi-Country Survey (MCSS)	Lebanon	2000	3233	18–100	Household survey	P, C, B	BMI; Income
806	WHO Multi-Country Survey (MCSS)	Mexico	2000	4800	18–96	Household survey	P, C, B	BMI; Income
807	WHO Multi-Country Survey (MCSS)	Nigeria	2000	5037	18–100	Household survey	P, C, B	BMI; Income
808	WHO Multi-Country Survey (MCSS)	Singapore	2000	6216	24–85	Household survey	P, C, B	BMI
809	WHO Multi-Country Survey (MCSS)	Slovakia	2000	1174	18–87	Household survey	P, C, B	BMI; Income
810	WHO Multi-Country Survey (MCSS)	Syria	2000	8603	18–100	Household survey	P, C, B	BMI; Income
811	WHO Multi-Country Survey (MCSS)	Turkiye	2000	5135	18–85	Household survey	P, C, B	BMI; Income
812	WHO STEPS Nepal	Nepal	2019	5593	15–69	Household survey	P, Ha, B	BMI; Smoking
813	WHO Study on Global Aging and Adult Health (SAGE)	Bangladesh	2007	4004	50–100	Household survey	P	
814	WHO Study on Global Aging and Adult Health (SAGE)	China	2007	14607	18–99	Household survey	P, Ha, S/A, B	BMI; Smoking; Income
815	WHO Study on Global Aging and Adult Health (SAGE)	Ghana	2007	9398	18–100	Household survey	P, Ha, S/A, B	BMI; Smoking; Income
816	WHO Study on Global Aging and Adult Health (SAGE)	India	2007	11227	18–100	Household survey	P, Ha, S/A, B	BMI; Smoking; Income
817	WHO Study on Global Aging and Adult Health (SAGE)	Indonesia	2007	11733	50–100	Household survey	P	
818	WHO Study on Global Aging and Adult Health (SAGE)	Kenya	2007	1991	50–100	Household survey	P	
819	WHO Study on Global Aging and Adult Health (SAGE)	Mexico	2007	2622	22–100	Household survey	P, Ha, S/A, B	BMI; Smoking; Income
820	WHO Study on Global Aging and Adult Health (SAGE)	Russian Federation	2007	4338	18–100	Household survey	P, Ha, S/A, B	BMI; Smoking; Income
821	WHO Study on Global Aging and Adult Health (SAGE)	South Africa	2007	4145	18–100	Household survey	P, Ha, S/A, B	BMI; Smoking; Income
822	WHO Study on Global Aging and Adult Health (SAGE)	Tanzania	2007	5010	50–97	Household survey	P	
823	WHO Study on Global Aging and Adult Health (SAGE)	Vietnam	2007	8515	50–99	Household survey	P	
824	WHO World Health Survey (WHS)	Austria	2003	1051	18–92	Cross-section	P, B	BMI
825	WHO World Health Survey (WHS)	Bangladesh	2003	5544	18–100	Cross-section	P, B	BMI
826	WHO World Health Survey (WHS)	Belgium	2003	1004	17–98	Cross-section	P, B	BMI
827	WHO World Health Survey (WHS)	Bosnia and Herzegovina	2003	1028	18–84	Cross-section	P, B	BMI
828	WHO World Health Survey (WHS)	Brazil	2003	5000	18–95	Cross-section	P, B	BMI
829	WHO World Health Survey (WHS)	Burkina Faso	2003	4821	18–100	Cross-section	P, B	BMI
830	WHO World Health Survey (WHS)	Chad	2003	4626	17–100	Cross-section	P, B	BMI

831	WHO World Health Survey (WHS)	China	2003	3993	18–99	Cross-section	P, B	BMI
832	WHO World Health Survey (WHS)	Comoros	2003	1759	18–90	Cross-section	P, B	BMI
833	WHO World Health Survey (WHS)	Congo	2003	2246	18–85	Cross-section	P, B	BMI
834	WHO World Health Survey (WHS)	Croatia	2003	989	18–93	Cross-section	P, B	BMI
835	WHO World Health Survey (WHS)	Czech Republic	2003	929	18–93	Cross-section	P, B	BMI
836	WHO World Health Survey (WHS)	Côte d'Ivoire	2003	3147	18–95	Cross-section	P, B	BMI
837	WHO World Health Survey (WHS)	Denmark	2003	1003	17–92	Cross-section	P, B	BMI
838	WHO World Health Survey (WHS)	Dominican Republic	2003	4531	18–100	Cross-section	P, B	
839	WHO World Health Survey (WHS)	Ecuador	2003	4622	17–100	Cross-section	P, B	BMI
840	WHO World Health Survey (WHS)	Estonia	2003	1011	17–92	Cross-section	P, B	BMI
841	WHO World Health Survey (WHS)	Eswatini	2003	2059	18–98	Cross-section	P, B	BMI
842	WHO World Health Survey (WHS)	Ethiopia	2003	4929	18–100	Cross-section	P, B	BMI
843	WHO World Health Survey (WHS)	Finland	2003	1013	18–93	Cross-section	P, B	BMI
844	WHO World Health Survey (WHS)	France	2003	1007	18–92	Cross-section	P, B	BMI
845	WHO World Health Survey (WHS)	Georgia	2003	2755	18–100	Cross-section	P, B	BMI
846	WHO World Health Survey (WHS)	Germany	2003	1258	18–92	Cross-section	P, B	BMI
847	WHO World Health Survey (WHS)	Ghana	2003	3923	18–100	Cross-section	P, B	BMI
848	WHO World Health Survey (WHS)	Greece	2003	1000	18–95	Cross-section	P, B	BMI
849	WHO World Health Survey (WHS)	Guatemala	2003	4747	18–99	Cross-section	P, B	BMI
850	WHO World Health Survey (WHS)	Hungary	2003	1419	18–95	Cross-section	P, B	BMI
851	WHO World Health Survey (WHS)	India	2003	9870	17–100	Cross-section	P, B	BMI
852	WHO World Health Survey (WHS)	Ireland	2003	1011	18–92	Cross-section	P, B	BMI
853	WHO World Health Survey (WHS)	Israel	2003	1208	18–91	Cross-section	P, B	BMI
854	WHO World Health Survey (WHS)	Italy	2003	999	18–96	Cross-section	P, B	BMI
855	WHO World Health Survey (WHS)	Kazakhstan	2003	4495	18–89	Cross-section	P, B	BMI
856	WHO World Health Survey (WHS)	Kenya	2003	4406	18–100	Cross-section	P, B	BMI
857	WHO World Health Survey (WHS)	Laos	2003	4887	17–98	Cross-section	P, B	BMI
858	WHO World Health Survey (WHS)	Latvia	2003	855	18–89	Cross-section	P, B	BMI
859	WHO World Health Survey (WHS)	Luxembourg	2003	700	18–90	Cross-section	P, B	BMI
860	WHO World Health Survey (WHS)	Malawi	2003	5227	18–96	Cross-section	P, B	BMI
861	WHO World Health Survey (WHS)	Malaysia	2003	6016	18–97	Cross-section	P, B	BMI
862	WHO World Health Survey (WHS)	Mali	2003	3649	17–98	Cross-section	P, B	BMI
863	WHO World Health Survey (WHS)	Mauritania	2003	3763	17–95	Cross-section	P, B	BMI

864	WHO World Health Survey (WHS)	Mauritius	2003	3888	18–93	Cross-section	P, B	BMI
865	WHO World Health Survey (WHS)	Mexico	2003	38745	18–100	Cross-section	P, B	BMI
866	WHO World Health Survey (WHS)	Morocco	2003	5000	18–98	Cross-section	P, B	BMI
867	WHO World Health Survey (WHS)	Myanmar	2003	5886	18–99	Cross-section	P, B	
868	WHO World Health Survey (WHS)	Namibia	2003	3988	18–100	Cross-section	P, B	BMI
869	WHO World Health Survey (WHS)	Nepal	2003	8686	18–100	Cross-section	P, B	BMI
870	WHO World Health Survey (WHS)	Netherlands	2003	1091	18–84	Cross-section	P, B	BMI
871	WHO World Health Survey (WHS)	Norway	2003	969	18–93	Cross-section	P, B	BMI
872	WHO World Health Survey (WHS)	Pakistan	2003	6370	18–100	Cross-section	P, B	BMI
873	WHO World Health Survey (WHS)	Paraguay	2003	5139	18–95	Cross-section	P, B	BMI
874	WHO World Health Survey (WHS)	Philippines	2003	10075	18–99	Cross-section	P, B	BMI
875	WHO World Health Survey (WHS)	Portugal	2003	1030	18–94	Cross-section	P, B	BMI
876	WHO World Health Survey (WHS)	Russian Federation	2003	4419	18–94	Cross-section	P, B	BMI
877	WHO World Health Survey (WHS)	Senegal	2003	2959	17–99	Cross-section	P, B	BMI
878	WHO World Health Survey (WHS)	Slovakia	2003	1788	18–92	Cross-section	P, B	BMI
879	WHO World Health Survey (WHS)	Slovenia	2003	584	18–91	Cross-section	P, B	BMI
880	WHO World Health Survey (WHS)	South Africa	2003	2342	18–100	Cross-section	P, B	BMI
881	WHO World Health Survey (WHS)	Spain	2003	6363	18–100	Cross-section	P, B	BMI
882	WHO World Health Survey (WHS)	Sri Lanka	2003	6728	18–100	Cross-section	P, B	BMI
883	WHO World Health Survey (WHS)	Sweden	2003	1000	18–92	Cross-section	P, B	BMI
884	WHO World Health Survey (WHS)	Tunisia	2003	5059	18–100	Cross-section	P, B	BMI
885	WHO World Health Survey (WHS)	Turkiye	2003	11204	18–99	Cross-section	P	BMI
886	WHO World Health Survey (WHS)	Ukraine	2003	2844	18–95	Cross-section	P, B	BMI
887	WHO World Health Survey (WHS)	United Arab Emirates	2003	1180	18–90	Cross-section	P, B	BMI
888	WHO World Health Survey (WHS)	United Kingdom	2003	1197	18–99	Cross-section	P, B	BMI
889	WHO World Health Survey (WHS)	Uruguay	2003	2979	18–93	Cross-section	P, B	BMI
890	WHO World Health Survey (WHS)	Vietnam	2003	3490	18–95	Cross-section	P, B	BMI
891	WHO World Health Survey (WHS)	Zambia	2003	3802	18–99	Cross-section	P, B	BMI
892	WHO World Health Survey (WHS)	Zimbabwe	2003	4051	17–100	Cross-section	P, B	BMI
893	WHO World Health Survey Plus (WHS+)	Cambodia	2023	5320	18–88	Cross-section	P, B	BMI
894	Wisconsin Longitudinal Study (WLS)	United States	2010	4989	70–74	Cohort	P, Ha, N/S, C, S/A, B, K, H, Hnd, Ft	BMI; Smoking; Income
895	Young Lives: Caregiver Survey	Ethiopia	2002	1979	10–58	Household survey	Ha	
896	Young Lives: Caregiver Survey	India	2002	2000	12–54	Household survey	Ha	

897	Young Lives: Caregiver Survey	Peru	2002	2046	14–58	Household survey	Ha	
898	Young Lives: Caregiver Survey	Vietnam	2002	1906	15–59	Household survey	Ha	
899	Young Lives: Child Survey	Ethiopia	2016	11761	11–29	School-based survey	Ha, S/A	
900	Young Lives: Child Survey	India	2016	9720	12–18	School-based survey	Ha, S/A	
901	Young Lives: Child Survey	Vietnam	2016	8378	14–24	School-based survey	Ha, S/A	
902	Yucatan Aging Study (YAS)	Mexico	2015	6675	68–100	Household survey	P	

Abbreviations: P, any pain; Ha, headache; N/S, neck or shoulder pain; C, chest pain; S/A, stomach or abdominal pain; B, back pain; K, knee pain; H, hip pain; Hnd, hand pain; Ft, foot pain; F, facial pain; E, elbow pain, PI, pain intensity.
a. Generation 21: At the Year 7 wave, pain items were parent-reported (mother or father responded on behalf of the child).
b. KiGGS: Pain items were parent-reported for children aged 3–10 years; self-reported for participants aged 11–17 years.
c. NESDA: Only the control sample (participants not selected on the basis of a depressive or anxiety disorder history) was included.
d. UK Biobank 2020: Refers to the UK Biobank "Experience of Pain" follow-up questionnaire; this instrument was used only to extract Hand, Foot, and Elbow pain.
e. LSMS: Headache item was parent-reported for adolescents aged 3–17 years.
f. Generation 21, New Zealand Health Survey, and Murakami Cohort contributed summary statistics rather than individual-participant data.

Data were identified and accessed through a multi-stage process that combined literature searches, repository searches, and direct contact with investigators. We searched PubMed, Google Scholar, and Global Health Data Exchange (GHDx) using terms related to overall and site-specific pain, including "pain", "bodily pain", "back pain", "knee pain", "headache", "neck pain", "pain prevalence", and others, to identify population-based studies that met our inclusion criteria. In parallel, we reviewed major public microdata repositories, including Inter-university Consortium for Political and Social Research (ICPSR), the World Health Organization (WHO) Microdata Library, the World Bank Microdata Catalog, Harvard Dataverse, the UK Data Service, and national statistics portals. Publicly available datasets were downloaded directly from their respective repositories.

For studies that were not publicly available or for which pain variables were not included in public releases, we contacted study investigators to request access to individual participant data. We also submitted data-access applications to large population cohorts and biobanks such as Lifelines, UK Biobank, and CONSTANCES, which met inclusion criteria. Custodians of COPCORD and related rheumatology programs were contacted separately to request participation and data sharing.

All but three studies shared individual participant data. Generation 21, the New Zealand Health Survey, and the Murakami Cohort were governed by data-protection protocols that did not permit transfer of individual-level data outside the host institution. For these studies, local analysts generated age and sex stratified summary statistics for each pain site using a standardized protocol. All other studies provided full individual participant level data suitable for harmonization of pain variables and relevant covariates.

2.2 Data inclusion criteria

We included population-based surveys and cohort studies that recruited participants from the general population or from broadly defined community samples without restricting eligibility based on health status or specific clinical diagnoses. Studies were eligible if they included at least one self-report item assessing pain at one or more of the target bodily sites (head, face, neck or shoulder, chest, back, stomach or abdomen, hip, knee, hand or wrist, foot or ankle, or elbow), or a self-report measure of overall or average pain intensity recorded on a 0–10 numerical rating scale or visual analogue scale with 0 indicating no pain. Pain had to be assessed directly through participant self-report. Studies were excluded if they selectively sampled individuals on the basis of particular clinical conditions, did not assess any of the target pain sites or pain intensity, or relied on clinician ratings, proxy reports, or observational assessments rather than self-report. For a small number of paediatric cohorts, parent-reported pain items were accepted for young children below the age of reliable self-report (supplementary table 2, footnotes 1, 2, 5). For inclusion in the pooled analyses, individual-level data had to be available for at least age, sex, and one harmonizable pain variable.

Section 3. Variable definitions, harmonization, and derived outcomes

3.1 Pain outcome harmonization

Across contributing studies, bodily pain was assessed using a range of instruments and question formats. Question wording varied substantially and included both time bound items, such as "*Over the past month, have you had any aches or pains in your body*?" or "*In the last 12 months, have you had pain that interfered with your life?*", and non-time specific items, such as "*Are you*

currently suffering from bodily pain?" or "*Do you have pain in your [body site]?*". Recall periods ranged from very short windows, such as current pain or past day, through short to intermediate windows, such as past week or past month, to extended windows, such as past 3 months, 6 months, up to past 12 months. We did not restrict inclusion based on the exact question syntax or recall window. Instead, pain variables were harmonized by anatomical site and recall period was treated as a methodological covariate in the statistical models. For each pain item, we recorded the stated recall period and created a harmonized recall variable with three levels: current or short-term recall (current to past week), intermediate recall (past month), and extended recall (3 months or longer, including past 3, 6, or 12 months). This recall variable was included as a covariate in all models, and model-based prevalence estimates were standardized to a past month recall by setting the recall category to the intermediate (past month) level when generating predictions.

Past-month recall was selected as the standardisation reference based on measurement evidence and practical considerations. Validation studies comparing retrospective pain ratings against prospective momentary assessments have shown that recall accuracy degrades as the reporting period extends, but that correspondence between recalled and daily ratings remains acceptable at 28 days ($r \geq 0.80$)[4,5]. Direct validation of the 1-month reference frame for the Graded Chronic Pain Scale demonstrated good psychometric properties and higher reporting stability (ICC = 0.89) compared with the 6-month version (ICC = 0.66), indicating a distinct recency advantage for the 30-day period[6]. Shorter recall periods (past week or less) risk underestimating the burden of symptoms that fluctuate day-to-day, while longer periods (3–12 months) introduce greater recall inflation and may conflate current with historical pain experience[7,8]. Past-month recall therefore provides a favourable balance between accuracy and representativeness. Additionally, past-month was the most common recall period across contributing data sources (used in 48% of studies compared to 25% for past week and 27% for past 3 months or longer), maximising the proportion of observations requiring no model-based adjustment.

We additionally included a study level indicator for pain case definition because 20 of 138 cohorts assessed chronic pain explicitly, typically requiring pain duration of at least 3 months, whereas other cohorts assessed pain without a chronicity requirement. Chronic explicit case definitions are expected to yield systematically lower prevalence than general pain questions[9] so this indicator was included as a fixed effect alongside recall window to adjust for systematic differences in outcome definition across cohorts. Prevalence estimates were then standardized to the general pain definition by fixing this indicator to the general pain level when generating predictions.

For interpretability, the standardized outcome can be understood as pain, aching, or discomfort in a given anatomical region reported as experienced within the past month.

We identified a core set of eleven anatomical sites that were sufficiently common across datasets and affected by pain across life to support pooled modelling: head, face, neck or shoulder, chest, back, stomach or abdomen, hip, knee, hand or wrist, foot or ankle, and elbow. Study specific pain items were mapped to these harmonized categories using study provided codebooks, original questionnaires where available, and, when necessary, consultation with study investigators. As an example, terms such as "oral", "jaw", and "orofacial" were grouped under facial pain, and items referring to "spine" or "lower back" were grouped as back pain. Where studies assessed paired or adjacent regions separately, these were combined into the corresponding composite category,

including neck or shoulder, hand or wrist, and foot or ankle, to maximise comparability with surveys that used joint items. A complete mapping from study specific item labels, question texts, and body map codes to the harmonized anatomical categories is provided in supplementary table 2.

Supplementary Table 3: Harmonization definitions and inclusion criteria for anatomical pain sites

Site	Operational anatomical definition	Included source phrasings	Notes/edge rules
Head	Scalp/cranial regions: forehead, temples, crown, occiput	"headache", "head pain/discomfort", "pain in the head"	Exclude face or jaw items; exclude ear pain
Face	Orofacial regions: jaw, cheeks, mouth, peri-oral	"facial pain/discomfort", "jaw pain", "mouth pain", "orofacial pain"	
Neck/ Shoulder	Cervical region and shoulder girdle: anterior/posterior neck, trapezius, acromion/clavicle, periscapular	"neck pain/discomfort", "shoulder pain/discomfort", "stiff neck", "shoulder blade pain"	Map "upper back" here only when trapezius or shoulder blade is specified; otherwise count as back
Chest	Anterior thorax: sternum, ribs, upper front torso	"chest pain/discomfort", "rib pain", "thoracic pain/discomfort"	Exclude diagnostic specifications such as "angina"; include symptom wording only. Exclude breast pain and heartburn
Back	Posterior thorax and lumbar regions: upper, mid, lower back; spine; waist	"back pain/discomfort", "back ache", "lower back", "lumbar", "mid-back", "upper back", "spine", "waist pain"	If "upper back" lacks trapezius/shoulder cues, treat as back (not neck/shoulder)
Stomach/ Abdomen	Anterior abdomen/stomach: upper or lower stomach or abdomen, belly	"abdominal pain/discomfort", "stomach pain/discomfort", "stomach ache", "belly ache"	
Hip	Hip joint and peri-pelvic/inguinal: groin, lateral hip, buttocks/gluteal	"hip pain/discomfort", "groin pain", "buttocks pain", "outer pelvis", "hip stiffness", "hip swelling"	
Knee	Knee joint: anterior/posterior, patella	"knee pain/discomfort", "pain in the knee", "knee joint", "knee stiffness", "knee swelling"	
Hand/Wrist	Distal upper limb: wrist, hand, palm, fingers, thumb	"hand pain/discomfort", "wrist pain/discomfort", "finger pain", "thumb pain"	
Elbow	Elbow region	"elbow pain/discomfort", "pain in elbow"	
Foot/Ankle	Distal lower limb: ankle, heel, foot, toes	"foot pain/discomfort", "ankle pain/discomfort", "heel pain", "toe pain"	
Any Bodily Pain	Composite outcome representing pain present at any assessed location or globally	"general pain", "bodily pain/discomfort", "pain intensity >	Derived variable. Study inclusion restricted to those assessing ≥5 specific sites OR a general bodily pain item OR a general

		0”, or endorsement of ≥1 specific anatomical site	pain/intensity item. Participants coded as present if reporting pain on any eligible item.
Joint Pain	Composite phenotype representing pain in appendicular joints (shoulder, elbow, wrist/hand, hip, knee, ankle/foot)	“joint pain”, “aching joints”, “painful joints”, “stiff joints”	Derived variable: coded present if ≥1 joint site reported or if a study assessed general joint pain without anatomical specification.

We derived a composite "any bodily pain" outcome for studies that assessed at least five anatomical sites, included a general pain item (defined as a single question assessing overall bodily pain without site specification; for example, "During the past month, have you had any bodily pain?"), or collected pain intensity ratings on a numerical scale. Participants were classified as having "any bodily pain" if they reported pain at one or more specific anatomical sites, endorsed the general pain item, or reported pain intensity greater than 0. Studies that assessed fewer than five anatomical sites and did not include a general pain item were excluded from this composite outcome.

The primary outcomes for site specific analyses were binary indicators of pain presence at each anatomical site. Because contributing cohorts used heterogeneous instruments, question wording, and response options, we followed established principles for retrospective harmonization and integrative data analysis by prespecifying a target construct and applying transparent, rule-based recoding to derive comparable variables across studies[10,11]. Most cohorts provided site specific pain outcomes that were already dichotomous (120 of 138), whereas a smaller subset used ordinal response options (18 of 138), requiring prespecified thresholding to derive binary indicators. For ordinal frequency items, categories reflecting pain occurring at least weekly (e.g., 'once a week', 'often', 'daily') were coded as present. For ordinal severity items, responses indicating more than minimal discomfort were coded as present; responses such as 'no pain', 'a little', or 'slight' were coded as absent. Items whose wording or response structure could not be mapped transparently to this binary definition were excluded from pooled analyses for the relevant site.

3.2 Secondary pain outcomes

Secondary outcomes captured both pain severity and the spatial extent of pain. Where available, average or general pain intensity rated on a 0–10 numerical rating scale or visual analogue scale with 0 indicating no pain was retained as a continuous variable. We restricted these analyses to items that explicitly asked about "average", "usual", or "overall" pain intensity, and excluded items that referred only to "worst pain", "least pain", or "pain at its most intense". Intensity items coding only coarse ordinal response categories such as mild, moderate, or severe were not included in the intensity analyses, as our focus was on numerical and analogue scales that are widely used and validated as core outcome measures in clinical pain research[12,13].

As with pain presence, pain intensity items differed in recall period. We recorded the stated recall window for each intensity question and applied the same harmonized recall structure used for site-specific prevalence. Recall was grouped into short-term (current or past week), intermediate (past month), and extended (past 3 months). This recall variable was included as a covariate in all intensity models so that differences in reported intensity attributable to recall length were partially adjusted for. When generating model-based intensity trajectories, we standardized predictions to a past week recall, aligning with recommended guidelines in clinical pain assessment[5,14].

Spatial extent of pain was quantified using generalized widespread pain criteria defined by the 2016 fibromyalgia diagnostic criteria and subsequent updates by Wolfe and colleagues[15,16]. The aim was not to diagnose fibromyalgia, but rather to characterize the point prevalence of highly burdensome, anatomically widespread pain in the general population using contemporary consensus criteria. For each participant, we assigned the collected pain sites to five body regions (left upper, right upper, left lower, right lower, and axial). Regions were coded as positive if any

mapped site within that region was reported as painful; for example, a positive response for any of left hand, left elbow, left shoulder, or left arm would count as positive for the left upper region. Generalized widespread pain was then defined as pain present in at least four of the five bodily regions[15]. Only studies that collected sufficient laterality and axial site detail to construct all five regions were eligible for these analyses. In total, 28 of 138 studies met these criteria and were included. For models of generalized widespread pain, recall period was again included as a covariate, and predicted trajectories were standardized to a past week recall to match the recall horizon used in the underlying criteria[15]. Because many contributing studies did not collect the full symptom severity components (WPI/SSS), and chronicity (≥3 months) was not consistently assessed even among those with adequate site detail, we report generalised pain distribution rather than the full ACR fibromyalgia case definition.

3.3 Socioeconomic and geographic variables

Total household income

Total household income was used as a pragmatic indicator of socioeconomic position, capturing material resources and purchasing power at the household level, which are widely used dimensions of socioeconomic circumstances in population health research[17,18]. For children and adolescents, this variable reflects parent or guardian reported total household income.

Because income was collected using different currencies, scales, and reporting conventions across cohorts, we operationalized income as a within study relative rank rather than as an absolute monetary value. Where self-reported total household income was available, we grouped participants into within study income quintiles (lowest 20% through highest 20% among respondents with non-missing income) and used these ordered categories in modelling. Income quintiles should be interpreted as relative socioeconomic position within each cohort, not as an absolute measure of poverty across countries or survey years.

World region classification

Countries were assigned to world regions using the United Nations SDG Indicators regional groupings, which are based on the United Nations Statistics Division M49 geographic regions[19]. We used a slightly modified version of these SDG regional groupings which disaggregated the combined “Europe and Northern America” region into three groups: Northern America, Western Europe, and Eastern Europe, to improve interpretability and geographic resolution in high income settings. Each study observation was assigned a world region based on the country or territory in which the survey was conducted. The mapping of every country and territory in the pooled dataset to its assigned world region is provided in Supplementary Table 4.

Supplementary Table 4: Classification of countries and territories by world region

Country	Global Region
Bangladesh	Central Asia
India	Central Asia
Pakistan	Central Asia
Kazakhstan	Central Asia
Sri Lanka	Central Asia
Nepal	Central Asia
Vietnam	East and SE Asia
Thailand	East and SE Asia
Taiwan	East and SE Asia
South Korea	East and SE Asia
Singapore	East and SE Asia
Philippines	East and SE Asia
Malaysia	East and SE Asia
Laos	East and SE Asia
Japan	East and SE Asia
Indonesia	East and SE Asia
China	East and SE Asia
Cambodia	East and SE Asia
Myanmar	East and SE Asia
Montenegro	Eastern Europe
North Macedonia	Eastern Europe
Poland	Eastern Europe
Romania	Eastern Europe
Slovenia	Eastern Europe
Serbia	Eastern Europe
Slovakia	Eastern Europe
Ukraine	Eastern Europe
Moldova	Eastern Europe
Russian Federation	Eastern Europe
Latvia	Eastern Europe
Lithuania	Eastern Europe
Kosovo	Eastern Europe
Albania	Eastern Europe
Armenia	Eastern Europe
Bosnia and Herzegovina	Eastern Europe
Bulgaria	Eastern Europe
Azerbaijan	Eastern Europe

Czech Republic	Eastern Europe
Estonia	Eastern Europe
Georgia	Eastern Europe
Hungary	Eastern Europe
Croatia	Eastern Europe
Mexico	Latin America
Uruguay	Latin America
Puerto Rico	Latin America
Peru	Latin America
Paraguay	Latin America
Venezuela	Latin America
Guatemala	Latin America
Colombia	Latin America
Dominican Republic	Latin America
Cuba	Latin America
Chile	Latin America
Brazil	Latin America
Barbados	Latin America
Argentina	Latin America
Ecuador	Latin America
Saudi Arabia	North Africa and West Asia
United Arab Emirates	North Africa and West Asia
Turkiye	North Africa and West Asia
Tunisia	North Africa and West Asia
Syria	North Africa and West Asia
Qatar	North Africa and West Asia
Iran	North Africa and West Asia
Libya	North Africa and West Asia
Lebanon	North Africa and West Asia
Jordan	North Africa and West Asia
Israel	North Africa and West Asia
Morocco	North Africa and West Asia
Egypt	North Africa and West Asia
Canada	Northern America
Greenland	Northern America
United States	Northern America
Tonga	Oceania
New Zealand	Oceania
Australia	Oceania

Kiribati	Oceania
Mali	Sub-saharan Africa
Zambia	Sub-saharan Africa
Tanzania	Sub-saharan Africa
South Africa	Sub-saharan Africa
Senegal	Sub-saharan Africa
Nigeria	Sub-saharan Africa
Namibia	Sub-saharan Africa
Zimbabwe	Sub-saharan Africa
Mauritania	Sub-saharan Africa
Malawi	Sub-saharan Africa
Mauritius	Sub-saharan Africa
Ghana	Sub-saharan Africa
Ethiopia	Sub-saharan Africa
Eswatini	Sub-saharan Africa
Democratic Republic of the Congo	Sub-saharan Africa
Cote d'Ivoire	Sub-saharan Africa
Congo	Sub-saharan Africa
Comoros	Sub-saharan Africa
Chad	Sub-saharan Africa
Burkina Faso	Sub-saharan Africa
Kenya	Sub-saharan Africa
Luxembourg	Western Europe
Malta	Western Europe
Netherlands	Western Europe
Sweden	Western Europe
Portugal	Western Europe
Spain	Western Europe
Italy	Western Europe
Norway	Western Europe
Ireland	Western Europe
Finland	Western Europe
Greece	Western Europe
Germany	Western Europe
France	Western Europe
Denmark	Western Europe
Cyprus	Western Europe
Belgium	Western Europe
Austria	Western Europe

Switzerland	Western Europe
Iceland	Western Europe
United Kingdom	Western Europe

Human Development Index (HDI)
The Human Development Index (HDI) is a composite measure of human well-being developed by the United Nations Development Programme (UNDP). It combines three normalised dimensions: (1) health, measured by life expectancy at birth; (2) education, measured by mean years and expected years of schooling; and (3) standard of living, measured by gross national income (GNI) per capita. Each dimension is expressed on a scale from 0 to 1, and the HDI is calculated as the geometric mean of the three indices. Country level Human Development Index (HDI) values were obtained from the United Nations Development Programme (UNDP) Human Development Reports database (https://hdr.undp.org/data-center/human-development-index#/indicies/HDI), which provides annual HDI estimates by country. HDI values were linked to individual participants based on the country in which the study was conducted and the calendar year of data collection. When a study spanned multiple years, the HDI value corresponding to the midpoint year of data collection was used. If HDI values were not available for a specific country year, values were interpolated using adjacent years where possible. HDI was analyzed as a contextual, country level indicator of socioeconomic development and was not interpreted as an individual level socioeconomic measure.

Section 4. Data processing and quality control

We applied a common data processing pipeline to all studies. Across all contributing studies, the raw pooled dataset contained reported ages ranging from approximately 3 to 117 years. A small number of observations fell outside biologically plausible limits (e.g., ages >110) and were judged to reflect data-entry or coding errors; these records were removed prior to harmonization. To ensure consistent representation of early-life ages across studies, ages <5 years were winsorized to 5 years. To stabilize estimates at the upper end of the distribution, ages >100 years were winsorized to 100 years. After these procedures, the analytic dataset comprised participants aged 5–100+ years.

Implausible values for BMI and underlying anthropometrics were identified using global thresholds and set to missing. For adults, BMI values below 10 kg/m² or above 80 kg/m² were treated as implausible. For pediatric participants, BMI was handled using age and sex specific BMI for age reference categories based on established WHO growth charts[20]. BMI was analyzed as categories (normal weight, overweight, obese), using standard adult cut points and pediatric percentile-based definitions[21].

The final pooled sample included 138 study programmes (defined as overarching survey or cohort frameworks with a consistent questionnaire structure and design) spanning 118 countries and territories. Within these study programmes, 902 country- or wave-specific datasets were identified (for example, individual survey years or country implementations), contributing approximately 6 million cross-sectional individual-level observations with information on pain at one or more body sites or a pain intensity rating. Data collection spanned calendar years 1990 to 2025.

Section 5. Statistical methods

5.1 Primary prevalence models

We estimated age-specific prevalence trajectories using hierarchical generalized linear mixed-effects models (GLMM) with a binomial distribution and logit link function. This framework

accounted for the hierarchical and partially crossed structure of the data, adjusting for methodological heterogeneity across sources. All analyses were performed in R (version 4.4.1). For an individual i within study programme p and world region r, the probability of reporting pain (p_{ipr}) was modeled as:

$$\text{logit}(p_{ipr}) = \beta_0 + f(\text{Age}_i, \text{Sex}_i) + \beta_{TW}\text{TimeWindow}_i + \beta_{CD}\text{CaseDefinition}_i + \beta_{Year}\text{Year}_c + u_r + v_p$$

Non-linear age trajectories were modeled using natural cubic splines (f) with 3 degrees of freedom. An interaction term between the age spline and sex allowed the shape of the lifespan trajectory to differ freely between females and males. To harmonize diverse survey instruments, the model included fixed effects for the recall Time Window (TW; e.g., past week, past month, past 3-12 months) and Case Definition (CD; general pain vs. explicit chronic definition), and survey year (Year_c; centred on the sample mean). To account for the complex clustering of the data, we specified a crossed random effects structure. This included random intercepts for world region (u_r) and study programme (v_p).

We derived globally comparable reference curves using estimated marginal means. Predicted probabilities were generated on a grid of ages from 5 to 100 years and standardized to a harmonized reference profile defined as "past month" recall and "general" (i.e., as opposed to chronic) pain definition, and the mean survey year. Random effects for world region and study were included during model fitting to account for clustering and heterogeneity. For reporting reference curves and contrasts, predictions were computed from fixed effects only, with random effects set to zero, corresponding to a reference region and study in the model. Disparities were summarized as age specific risk differences and risk ratios on the probability scale. Uncertainty for predicted probabilities and age-specific contrasts was quantified using Wald-based standard errors derived from the fixed-effects variance–covariance matrix of the fitted mixed-effects model, and we report pointwise 95% confidence intervals.

5.2 Disparity metrics and dynamics

We quantified group disparities at each age using the Risk Difference (RD) ($p_{\text{Group A}} - p_{\text{Group B}}$) and Risk Ratio (RR) ($p_{\text{Group A}}/p_{\text{Group B}}$). When reporting summary contrasts within pre-specified age bands (5–17, 18–35, 35–50, 50–65, 65–80, and $\geq$ 80 years), predicted probabilities were averaged across ages within the band before computing the RD and RR.

To characterize changes in prevalence across the life course, we computed the rate of change of the predicted prevalence curve using finite differences on a one-year grid ($[p_{a+1} - p_a]/1$ year). These rates are reported as percentage points per year to highlight phases of acceleration and deceleration.

5.3 Exposure analyses (BMI, smoking, income), HDI, region

To assess how pain trajectories are modified by sociodemographic and lifestyle factors, we extended the primary framework by interacting age with the exposure of interest. To ensure stable estimation of age-varying effects within smaller stratified subgroups, the age spline complexity was reduced to 2 degrees of freedom. For categorical risk factors (BMI category, smoking status,

household income quintile), we estimated age-specific prevalence trajectories and age-adjusted prevalence estimates for each subgroup. For national development, we modeled the Human Development Index (HDI) as a continuous variable interacting with the age spline. For presentation, predicted trajectories were generated at four representative HDI values (0.55, 0.70, 0.80, 0.90) aligned with United Nations thresholds for low, medium, high, and very high human development. The HDI models were the single exception to the survey-year adjustment described above: because country-level HDI is assigned at the survey year and is itself time-varying, HDI and survey year are only partially observed independently, and jointly adjusting for both would extrapolate beyond the region of overlapping data; the HDI trajectories were therefore estimated without a survey-year term. We derived adjusted prevalence estimates, risk differences, and risk ratios using estimated marginal means. All estimates were standardized to a harmonized reference profile ("past month" recall, "general" pain definition, and the mean survey year for models that included it) and computed from fixed effects as the reference trajectories.

5.4 Population attributable fraction (PAF) analysis

We estimated population attributable fractions (PAFs) to quantify the proportion of the pain burden associated with obesity, smoking, and low household income rank. We combined age- and sex-specific risk ratios (RRs) derived from the pooled cohort (Section 5.3) with country-, age-, and sex-specific exposure prevalence and population denominators from the Global Burden of Disease (GBD) study. Obesity prevalence was obtained from GBD 2021 estimates, merging child and adolescent (ages 5–24 years)[22] and adult (ages ≥ 25 years)[23] datasets to ensure continuity across the life course. Smoking prevalence was derived from the most recent GBD release providing granular age-sex resolution (GBD 2015)[24]. All external inputs were harmonised to 5-year age-by-sex strata and aligned to the analytic age range (ages ≥ 5 years, with the oldest group capped at ≥ 80 years). For obesity and smoking, PAFs were computed using the standard binary formulation:

$$\mathrm{PAF} = \frac{\mathrm{P}(\mathrm{RR} - 1)}{1 + \mathrm{P}(\mathrm{RR} - 1)}$$

where P denotes the country-, age-, and sex-specific exposure prevalence, and RR denotes the corresponding age- and sex-specific risk ratio.

For income, socioeconomic position was operationalised as within-study household income quintiles (relative rank), with the quintile prevalence fixed at $\mathrm{P} = 0.20$ under a relative-income framework. We estimated a partial polytomous PAF to quantify the pain burden specifically attributable to the lowest income quintile. This metric represents the model-based proportion of pain cases attributable to being in the bottom 20% of the income distribution relative to the top 20% (the reference group), while correctly accounting for the population-average risk implied by the full socioeconomic gradient. This was calculated as:

$$\mathrm{PAF}_{\mathrm{lowest}} = \frac{\mathrm{P}_{\mathrm{lowest}}(\mathrm{RR}_{\mathrm{lowest}} - 1)}{\sum_{\mathrm{i}=1}^{5} \mathrm{P}_{\mathrm{i}}\, \mathrm{RR}_{\mathrm{i}}}$$

where i indexes the income quintiles, $\mathrm{P}_{\mathrm{i}} = 0.20$ for all quintiles, and RR_{i} is the risk ratio for the i-th quintile relative to the highest income quintile. Because income was operationalised as within-study relative rank, the prevalence of each quintile is fixed at 0.20 across all countries by definition;

cross-national variation in income PAF therefore reflects differences in demographic structure interacting with age- and sex-specific risk ratios rather than differences in exposure prevalence.

To estimate the joint burden attributable to all three exposures simultaneously, we computed a combined PAF using the multiplicative independence formula:

$$PAF_{combined} = 1 - (1 - PAF_{obesity})(1 - PAF_{smoking})(1 - PAF_{income})$$

which combines the exposure-specific PAFs under an assumption of multiplicative independence across exposures. Country- and regional-level summary PAFs were obtained by summing exposure-attributable cases and total pain cases across age-sex strata and across the 11 anatomical pain sites, then computing the ratio. Regional and global estimates therefore represent case-weighted averages across the 11 site-specific models. Results are reported in supplementary p.81 (Section 8).

5.5 Secondary outcome models (average pain intensity, high-intensity pain, generalized widespread pain)

We analyzed average pain intensity (1–10 NRS; Extended Data Figure 2) and high-intensity pain (NRS $\geq$ 7) using the hierarchical framework described above, adjusted for recall window and clustering within study and region. Pain intensity was modeled using a generalized linear mixed model with a Gamma distribution and log link function to account for the non-negative, right-skewed distribution of symptom ratings. High-intensity pain and generalized widespread pain were modeled using a binomial distribution with a logit link.

Section 6. Sensitivity analyses

We conducted a series of sensitivity analyses to assess the robustness of our findings. These included: (1) a leave-one-study-out (LOSO) analysis to quantify single-study influence; (2) covariate adjustments for survey methodology (e.g., mode, setting); (3) data-exclusion scenarios based on recall period stringency; (4) alternative random-effects specifications (random slopes); (5) model selection for functional form (spline complexity); (6) an imputation analysis to evaluate the impact of missing exposure variables (e.g., BMI, smoking status, and household income) on exposure–pain associations and (7) a sampling-design analysis restricting to probability-based general-population samples and excluding volunteer/biobank-style cohorts to assess whether the reference trajectories were influenced by sampling.

The estimated reference curves demonstrated high stability across these sensitivity checks. The exclusion of individual studies and adjustment for minor methodological covariates yielded negligible shifts in prevalence estimates (typically < 1.0 percentage points). While recall period was identified as a primary source of heterogeneity, our fixed-effect adjustment strategy produced trajectories highly concordant with stricter data-inclusion subsets. Although random-slope specifications suggested slightly altered trajectory shapes for headache and back pain specifically (shifting the peak age or flattening the late-life decline), the fundamental conclusions regarding the magnitude of sex disparities, the relative ranking of pain sites, and the ubiquitous burden of pain across the lifespan remained consistent across all valid sensitivity scenarios.

Section 6.1 Leave-One-Study-Out Sensitivity Analysis

To evaluate the robustness of the estimated reference curves, we performed a leave-one-study-out (LOSO) sensitivity analysis. We iteratively removed one study at a time from the dataset, refit the generalized linear mixed models, and recalculated age-specific predicted prevalence using the same protocol applied in the main analysis. The influence of each study was quantified as the Mean Absolute Difference (MAD) which is defined as the mean absolute difference in predicted prevalence between the LOSO model and the full model, averaged across the modeled age range and sex.

The analysis demonstrated high consistency across all pain sites (Supplementary Figure 1). The trajectories were robust to the exclusion of individual datasets: all studies produced an MAE of less than 2.5 percentage points, with a median deviation of approximately 0.06 percentage points.

As shown in the study influence matrix (Supplementary Figure 2), the datasets with the greatest influence were primarily those providing unique coverage of specific age groups or world regions. These included the WHO Study on global Ageing and adult health (SAGE), the WHO Multi-Country Survey Study (MCSS), and the Libyan Pain Survey, which provide critical data from non-Western populations. Additionally, cohorts focused on childhood and adolescence such as CATS (Childhood to Adult Transition Study), PSC (Pain in School Children), HBSC (Health Behavior in School Age Children), and ABCD (Adolescent Brain Cognitive Development Study), were among the most influential. This is expected, as these studies anchor the early-life portion of the lifespan trajectory where data is sparser compared to adulthood. However, even when excluding these key datasets, the magnitude of change remained small (mean MAE ≈ 1-2.5%) and

did not alter the fundamental shape or relative ordering of the trajectories. This supports that the reported reference curves are not driven by the idiosyncratic features of any single study.

Supplementary Figure 1: Stability of global trajectories in leave-one-study-out sensitivity analysis

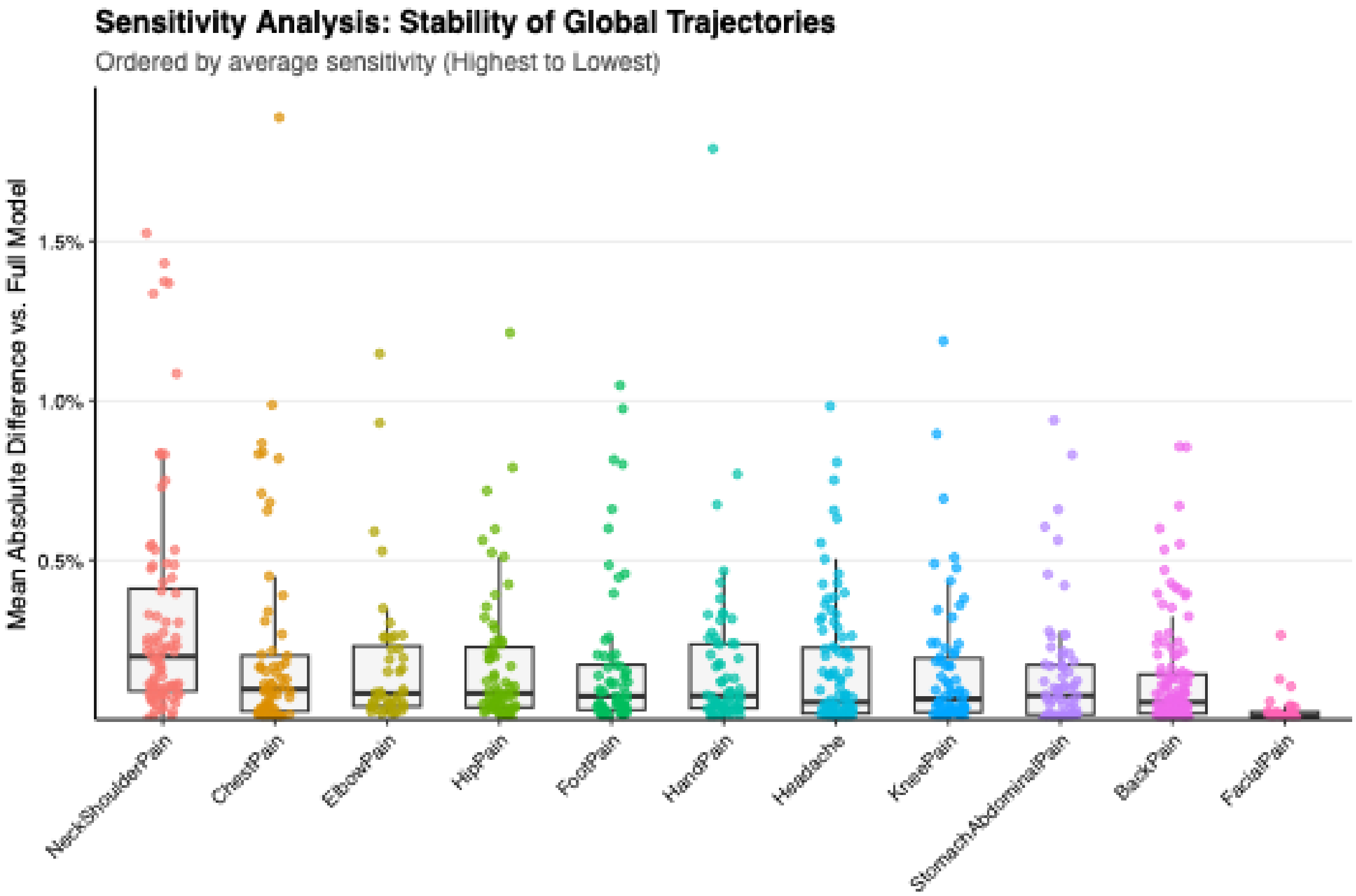

Distribution of the Mean Absolute Difference (MAD) in predicted prevalence between the full model and leave-one-study-out models. Each point represents a single excluded study. Boxplots indicate the median and interquartile range, whiskers extend to the most extreme values within 1.5 × the interquartile range.

Supplementary Figure 2: Matrix of individual study influence on prevalence estimates

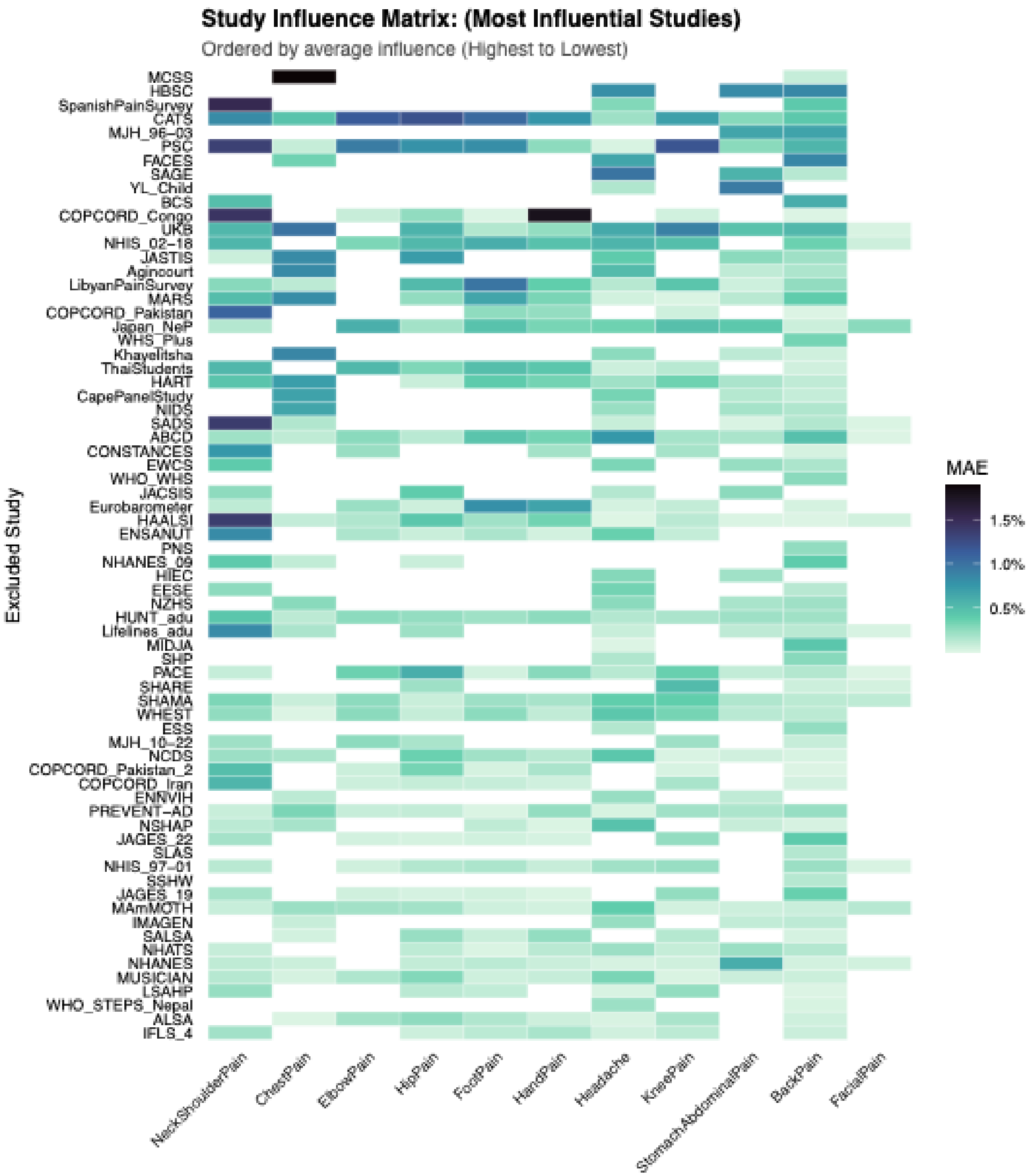

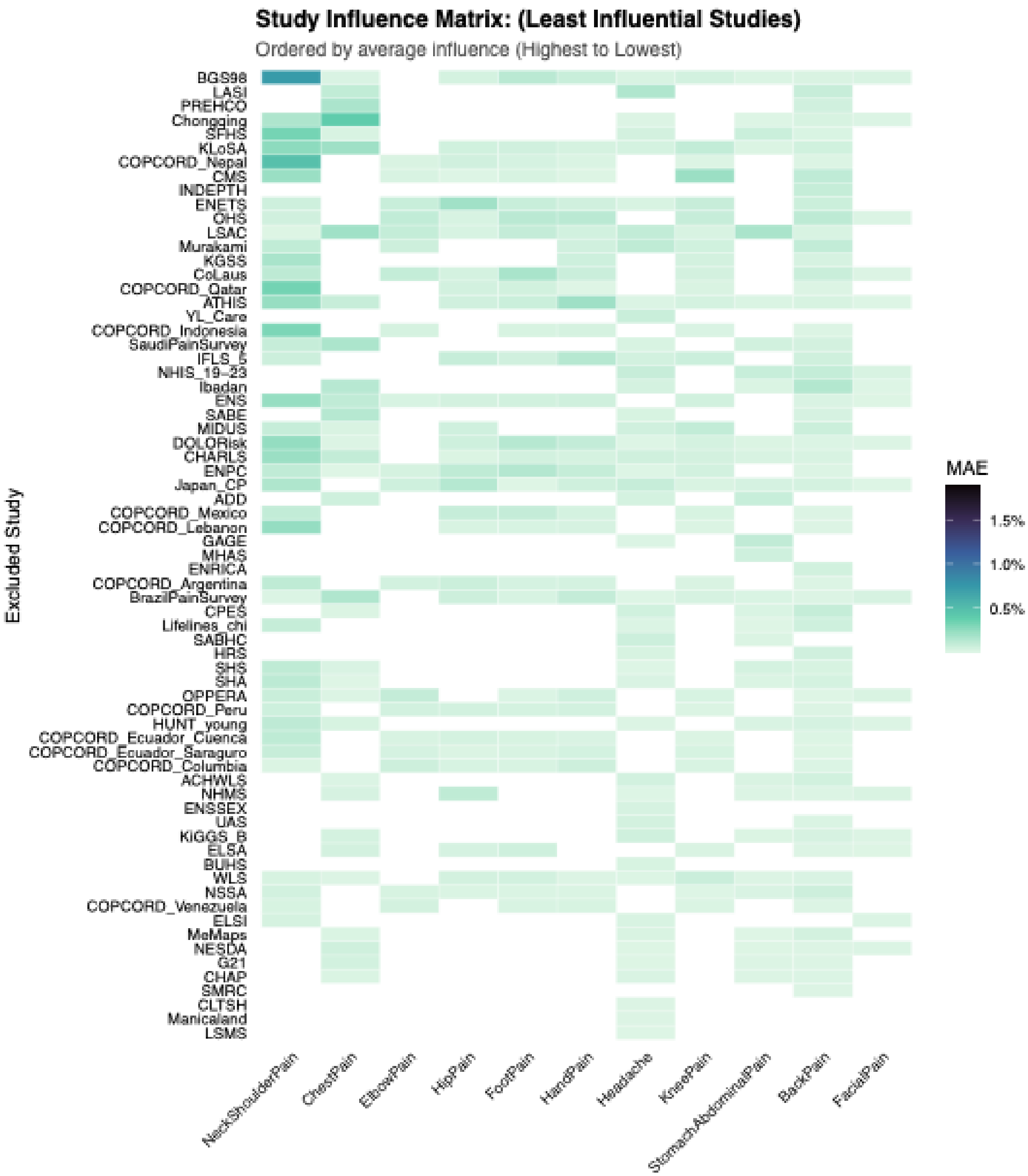

Mean Absolute Difference (MAD) in predicted prevalence when specific studies (y-axis) are excluded from the model. Studies are ordered by average influence across all pain sites.

Section 6.2. Sensitivity Analysis of Methodological Covariates

To assess the robustness of the estimated age–sex trajectories to study-level heterogeneity, we performed a sensitivity analysis ranking the influence of seven methodological covariates. We defined these covariates as follows:

- **Recall Period:** The reference time frame for pain reporting (e.g., "past week", "past month" or “past 3-12 months“).
- **Case Definition:** The stringency of the criteria used to classify pain (e.g., "chronic/frequent" vs. "general").
- **Survey Format:** The mode of administration (e.g., oral interview vs. written/online questionnaire).
- **Survey Setting:** The physical environment of data collection (e.g., in-person vs. remote).
- **Question Valence:** The phrasing of the pain item (True if pain questionnaire qualified by terms like “Bothered”, “Troubled”, “Suffered”, or “Interfered”, otherwise False).
- **Question Format:** The structure of response options (e.g., binary Yes/No vs. ordinal scales).
- **Sampling Strategy:** The method of participant recruitment (e.g., random probability sampling, convenience/web-based, or volunteer/biobank).

We specified a "naive" baseline mixed-effects model (natural spline for age [df=3], age–sex interaction, and random intercepts for study programme and world region) without methodological adjustments. We compared this baseline against separate models, each adding a single covariate as a fixed main effect. To quantify the population-level impact, we calculated weighted marginal predictions for all models. Deviations from the baseline were summarized as the Mean Absolute Difference (MAD) in percentage points (pp).

The analysis demonstrated high stability of the reference curves (Supplementary Figure 3). Across all pain sites and methodological factors, the mean absolute difference between the naive and adjusted models remained below 2.4 percentage points. This indicates that the specified random effects structure absorbed the majority of measurable methodological heterogeneity. As shown in Supplementary Figure 4, even under the adjustment of the maximally influencing covariate for each site, the magnitude of this shift was minimal.

The choice of recall period was the primary consistent driver of variation. Adjusting for recall period resulted in a mean trajectory shift of 1.2–2.4 pp for high-prevalence sites. Analysis of effect directionality (Supplementary Figure 5) confirmed that broader recall periods consistently yielded higher prevalence estimates than point prevalence. Consequently, recall period was retained as a fixed effect to ensure comparable reference standards. Case definition was also retained due to its clinical relevance, despite a moderate statistical impact (mean shift $\approx$ 0.5 pp).
In contrast, covariates like survey format, setting, sampling strategy, and valence had limited impacts (average mean shift $\approx$ 0.5 pp). Furthermore, these factors displayed inconsistent directionality across pain sites (e.g., "oral" surveys raised prevalence for some sites but lowered it for others), suggesting they may not introduce a systematic bias requiring global correction. Therefore, these factors were excluded from the final models.

Supplementary Figure 3: Sensitivity of prevalence estimates to methodological covariates

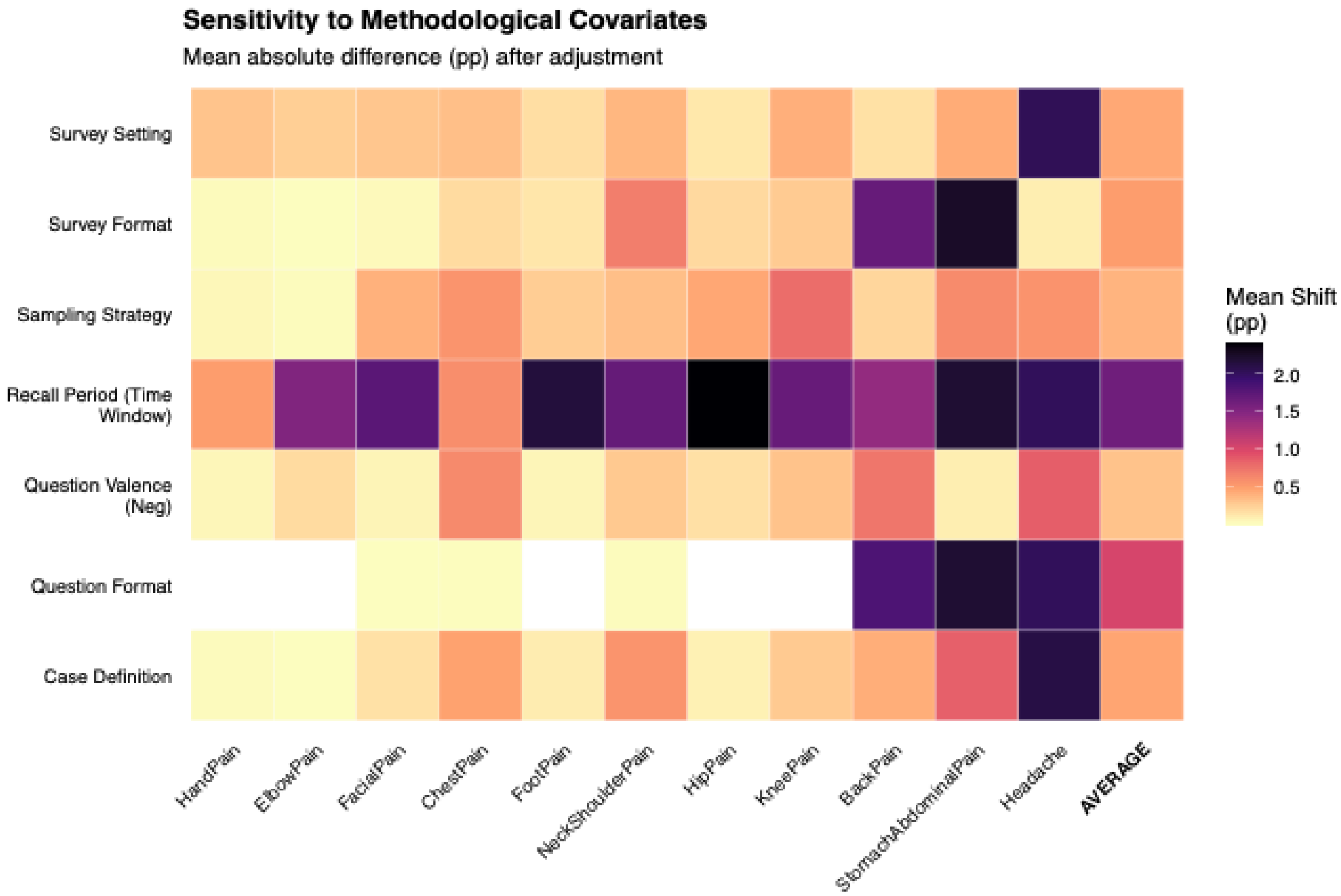

Mean absolute shift in predicted prevalence (percentage points) when adjusting for specific study-level factors compared to the naive baseline model.

Supplementary Figure 4: Trajectory robustness under maximum methodological adjustment

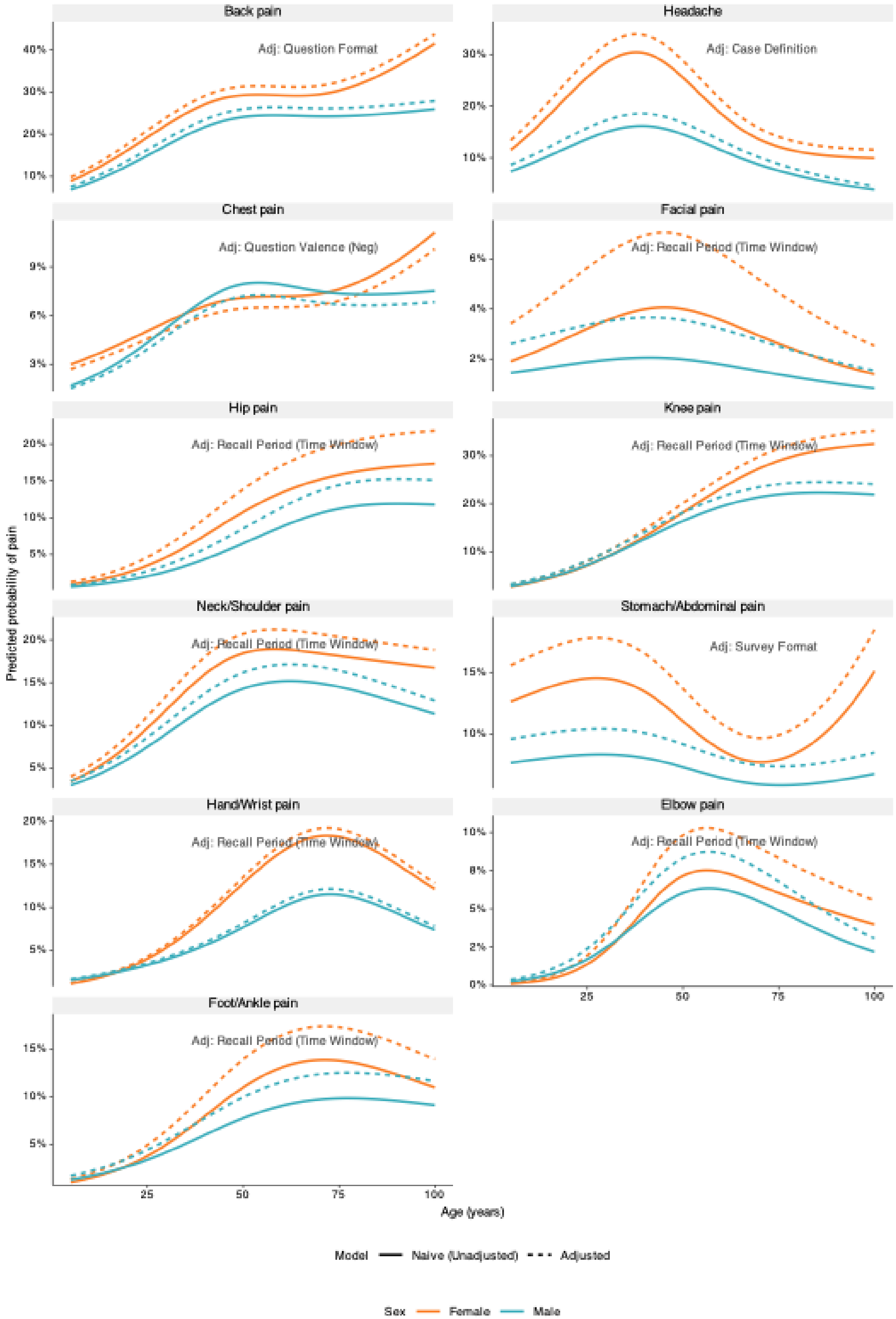

Comparison of the naive model (solid lines) against the model adjusted for the single most influential covariate for each pain site (dashed lines).

Supplementary Figure 5: Directionality of methodological effects on reported prevalence

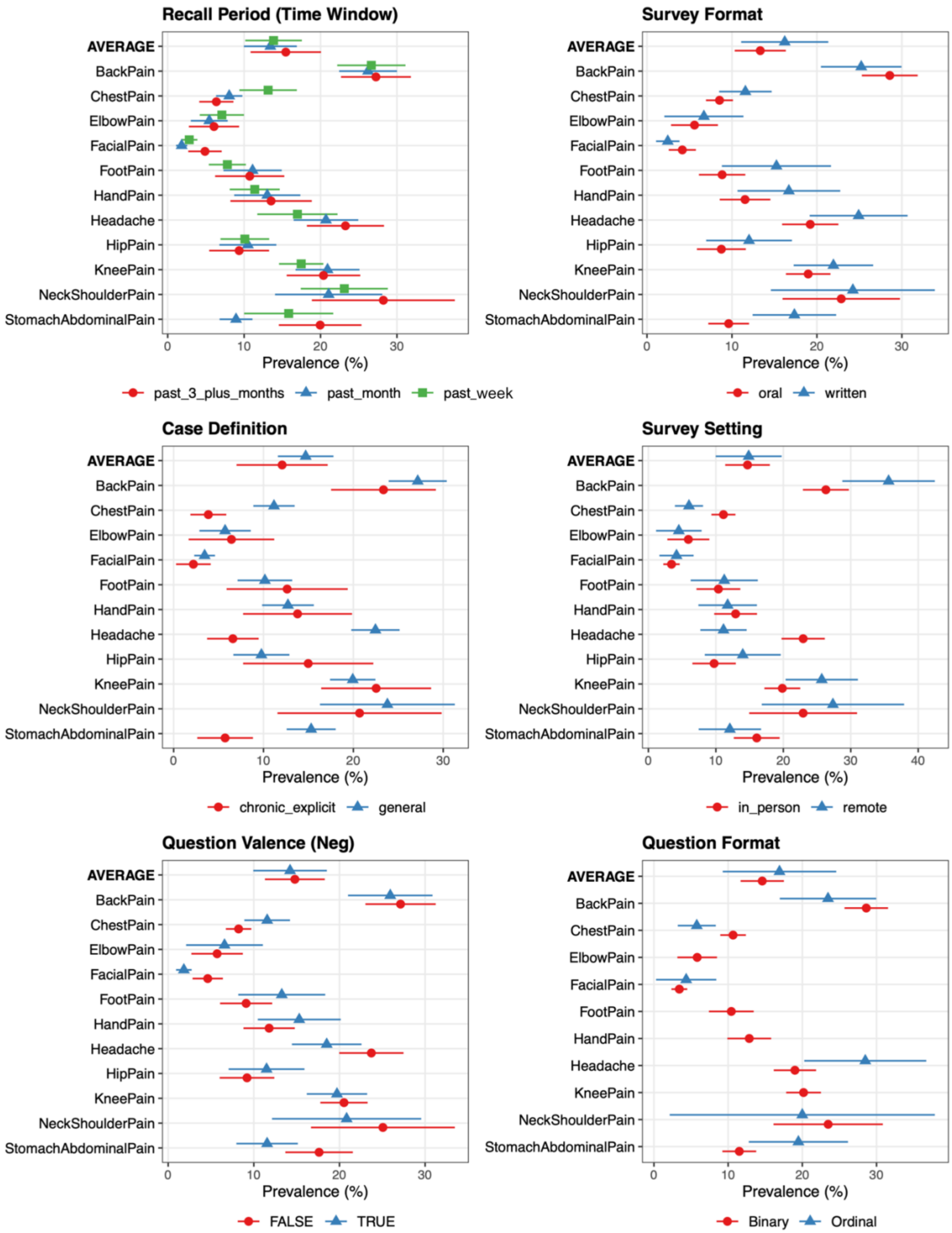

Marginal predicted prevalence by covariate category, standardized to the global age-sex distribution. Points indicate the mean estimate; error bars indicate 95% confidence intervals.

Section 6.3. Sensitivity Analysis of Data Exclusion based on Recall Period

To evaluate whether statistical adjustment for recall period materially influenced estimates, we conducted a data-exclusion sensitivity analysis comparing three analytical tiers. Tier Main (primary approach) included all recall periods (past week, past month, past ≥ 3 months) and incorporated a fixed-effect adjustment standardized to a past-month reference. This was compared with two restricted subsets. Tier A (Acute-Focused) excluded studies using past ≥ 3 month recall windows or explicit chronic pain definitions, retaining only past-week or past-month recall without chronic specification. Tier B (Strict) retained only studies explicitly using a past-month recall period. For Tier A, models were refit and standardized to past month; for Tier B, no recall adjustment was required. Absolute deviations between the primary model and the restricted subsets were calculated across the lifespan.

Trajectories from both restricted subsets showed high concordance with the primary model (Supplementary Figure 6). Tier A produced nearly identical estimates, with mean absolute deviations of 0.6 percentage points (Supplementary Figure 7), indicating strong alignment between adjusted past-week and past-month data.

The Strict subset (Tier B) generally tracked the primary model within 2.0 pp; however, greater variability was observed due to reduced sample size and geographic coverage. Deviations remained stable across the lifespan for most pain sites, though larger divergences (up to ~3.5 pp) were observed in the oldest age groups (≥ 80 years) for chest pain likely due to data sparsity in the strict subset. Overall, restricting the analysis to strictly past-month studies yielded trajectories substantively similar to the full adjusted model but with reduced precision, supporting the validity of using the full global dataset to maximize representativeness.

Supplementary Figure 6: Deviation of restricted data subsets from the primary model

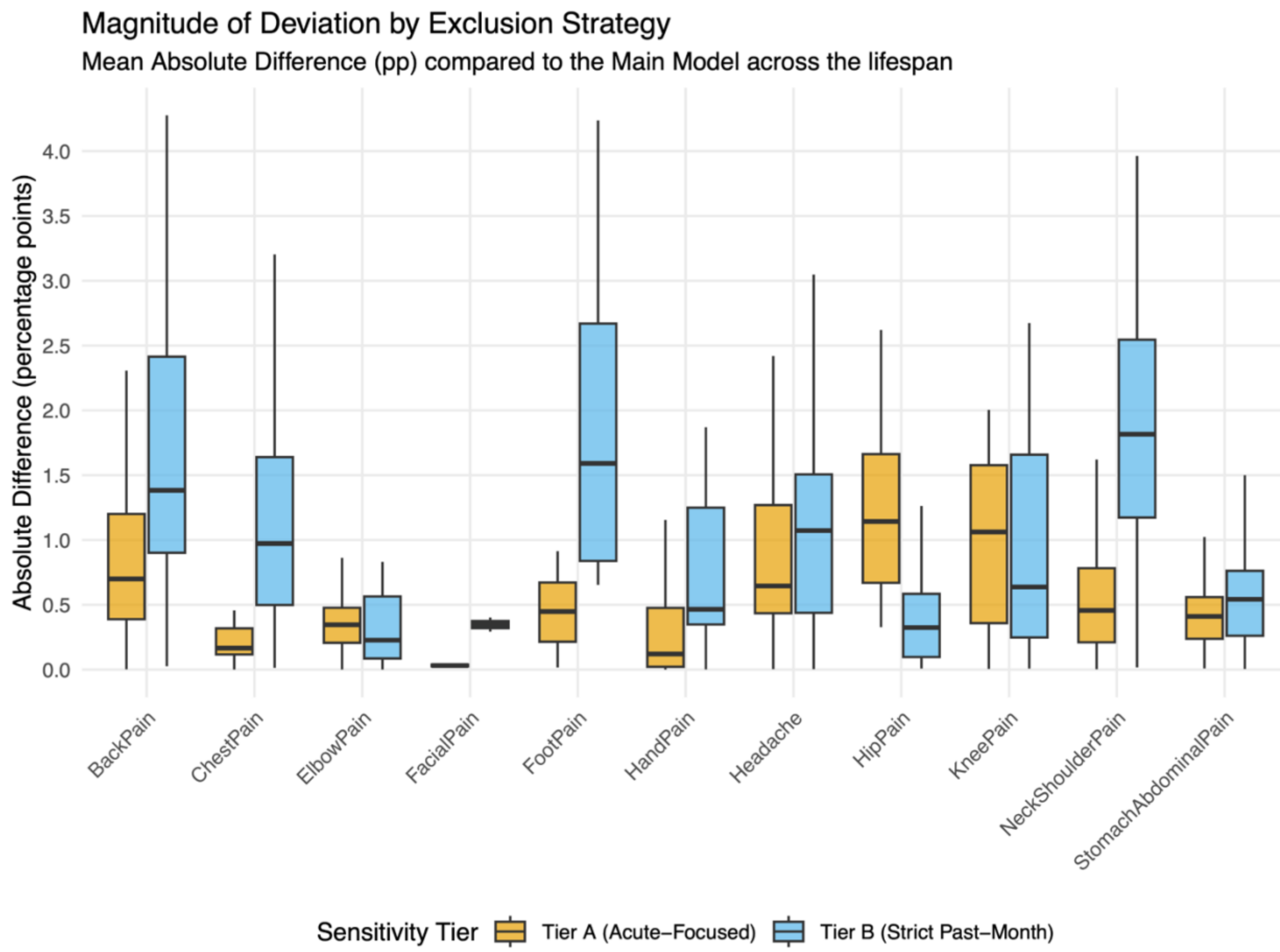

Distribution of absolute differences in prevalence estimates for the Acute-Focused (Tier A) and Strict (Tier B) subsets relative to the primary model. Boxplots indicate the median and interquartile range, whiskers extend to the most extreme values within 1.5 × the interquartile range.

Supplementary Figure 7: Concordance between primary model and restricted data subsets

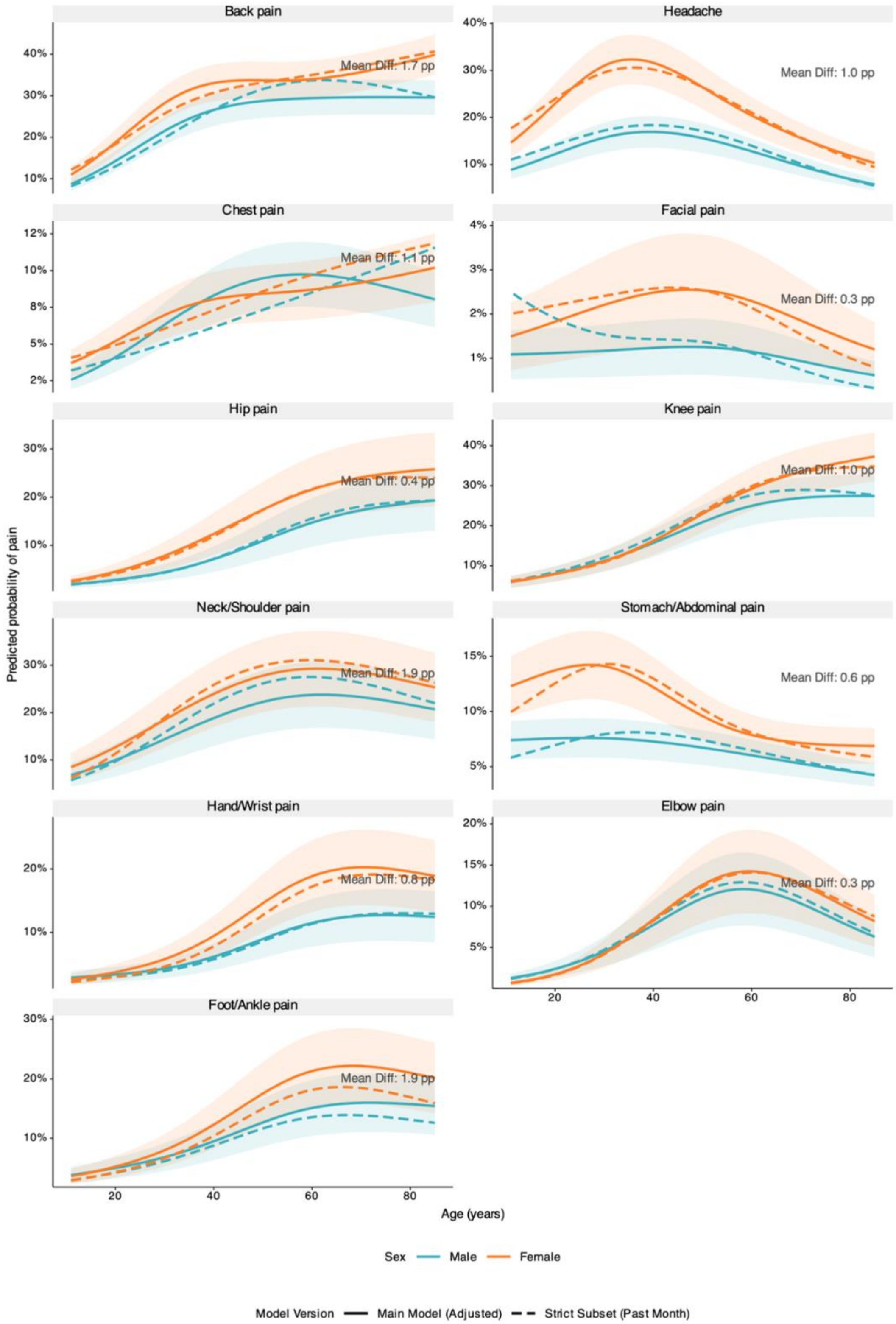

The primary model is compared against a restricted subset containing only studies explicitly using a "past month" recall period (dashed line). Shaded regions indicate 95% confidence intervals.

Section 6.4. Sensitivity Analysis of Random-Effects Structure

To evaluate the robustness of the estimated trajectories to model specification, we compared the primary model against an alternative random-effects structure allowing for study-specific random slopes for age. The primary model utilized crossed random intercepts (clustering by study and global region), assuming a common age trajectory shape while allowing prevalence levels to vary. The alternative model included random slopes to test whether the rate of aging varies systematically between study populations. Note that random slopes were only estimated for studies with sufficient within-study age variation to ensure statistical convergence.

For the majority of pain sites (9 of 11), the estimated age–sex trajectories were highly robust to changes in model structure (Supplementary Figure 8). The mean absolute difference between the primary and random-slope models for these sites was minimal (average MAD ≈ 0.9 pp; Supplementary Figure 9). However, the random-slope model produced notable deviations for two sites: headache (MAD = 3.9 pp), which exhibited a later age of decline, and back pain (MAD = 5.1 pp), which showed a steeper increase in late life compared to the primary model.

Despite these specific deviations, the primary random-intercept model was retained to ensure the most globally representative estimates. Estimating study-specific age slopes requires data spanning a wide age range; however, a substantial proportion of the included studies focus on specific life stages (e.g., exclusively adolescents or older adults) and lack the age span coverage to estimate a reliable age-slope. As such, relying on a random-slope specification would disproportionately weight results toward general population surveys with broad age spans, which predominantly come from high-income regions, while effectively discounting data from age-specific cohorts. The primary model integrates evidence from all study designs regardless of their internal age range, aiming to represent a more inclusive estimation of the global burden.

Supplementary Figure 8: Magnitude of deviation between random-intercept and random-slope models

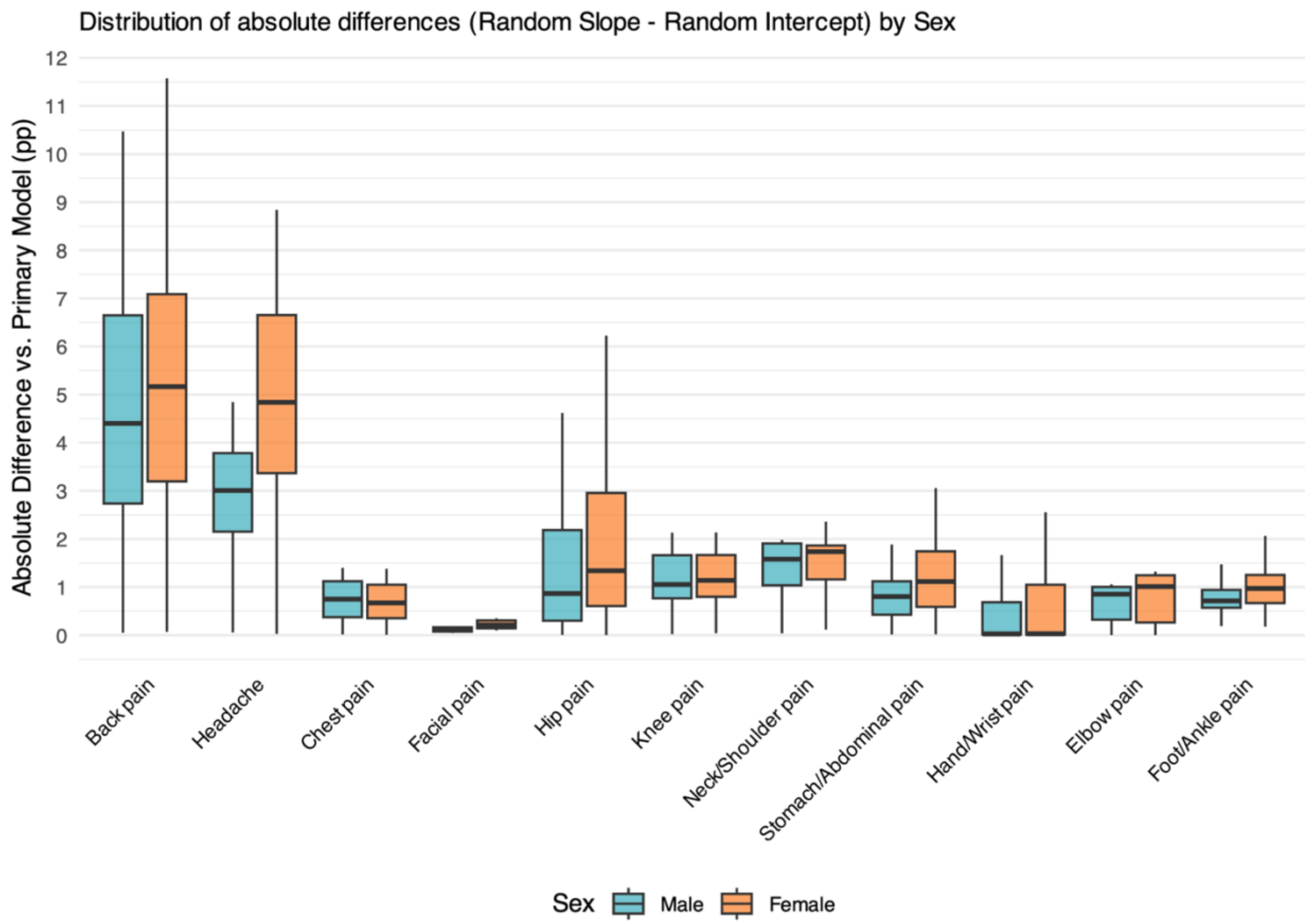

Distribution of absolute differences in predicted prevalence across the lifespan when including study-specific random slopes. Boxplots indicate the median and interquartile range, whiskers extend to the most extreme values within 1.5 × the interquartile range.

Supplementary Figure 9: Sensitivity of trajectories to random-effects structure

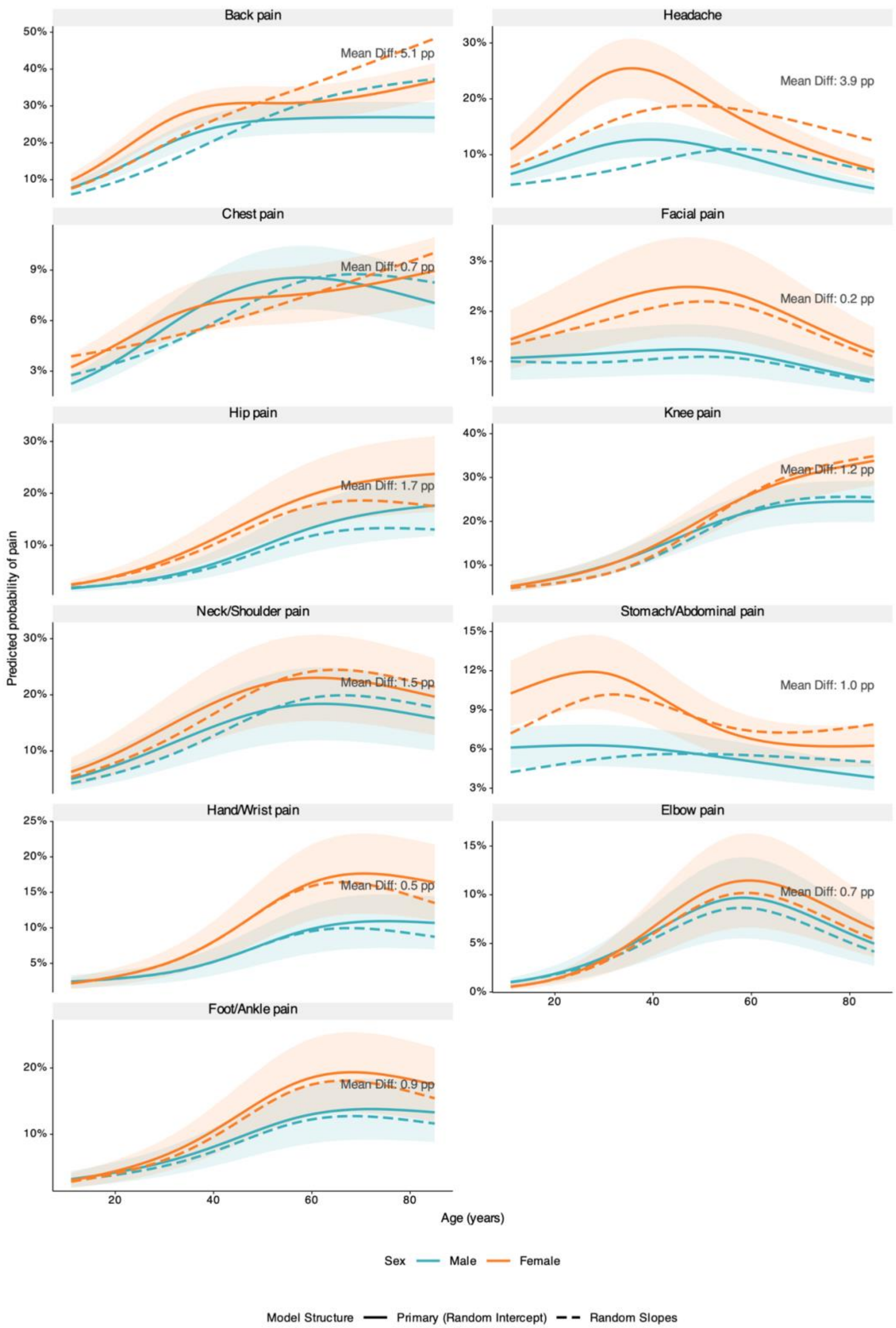

Comparison of the primary random-intercept model (solid lines) against a model allowing for study-specific random slopes for age (dashed lines).

Section 6.5. Sensitivity Analysis of Functional Form (Age Spline Complexity)

To determine the optimal functional form for the age trajectory, we performed a model selection sensitivity analysis comparing natural cubic splines with varying levels of complexity (degrees of freedom [df] = 1 to 4). We utilized a 5-fold cross-validation approach grouped by study to assess out-of-sample predictive performance, alongside Akaike Information Criterion (AIC) and Bayesian Information Criterion (BIC) on the full dataset to assess goodness-of-fit.

Performance metrics (Log Loss, AUC, and Brier score) were broadly similar across all degrees of freedom (Supplementary Table 5). For example, the AUC and Brier score for back pain remained stable at approximately 0.57 regardless of spline complexity, and Log Loss and Brier score differences were negligible (<0.01). This suggests that while age is a predictor of pain, increasing the complexity of the age curve does not significantly change the ranking of high-risk vs. low-risk individuals.

However, goodness-of-fit statistics calculated on the full dataset strongly favored non-linear specifications (Supplementary Figure 10). For all pain sites, AIC and BIC values decreased substantially moving from a linear specification (df=1) to a spline (df=3), with reductions often exceeding 10,000 points. This indicates that while a linear model can roughly rank risk, it significantly underfits the data and fails to accurately estimate the probability of pain at specific ages.

We selected df=3 (a natural spline) as the primary functional form for all sites. While df=4 provided marginal further improvements in BIC for some sites, the trajectory shapes for df=3 and df=4 were visually indistinguishable (Supplementary Figure 11), indicating that the additional complexity yielded little benefit in the epidemiological interpretation. Conversely, the linear model (df=1), despite having similar predictive power, lacks epidemiological plausibility; it fails to capture well-established non-monotonic life-course patterns, such as the early life peak followed by decline through late-life in headache. The df=3 specification provided the optimal balance, capturing the requisite biological curvature while maximizing parsimony and minimizing the risk of boundary artifacts (overfitting) in the oldest age groups where data density is lower.

Supplementary Figure 10: Model selection statistics for functional form

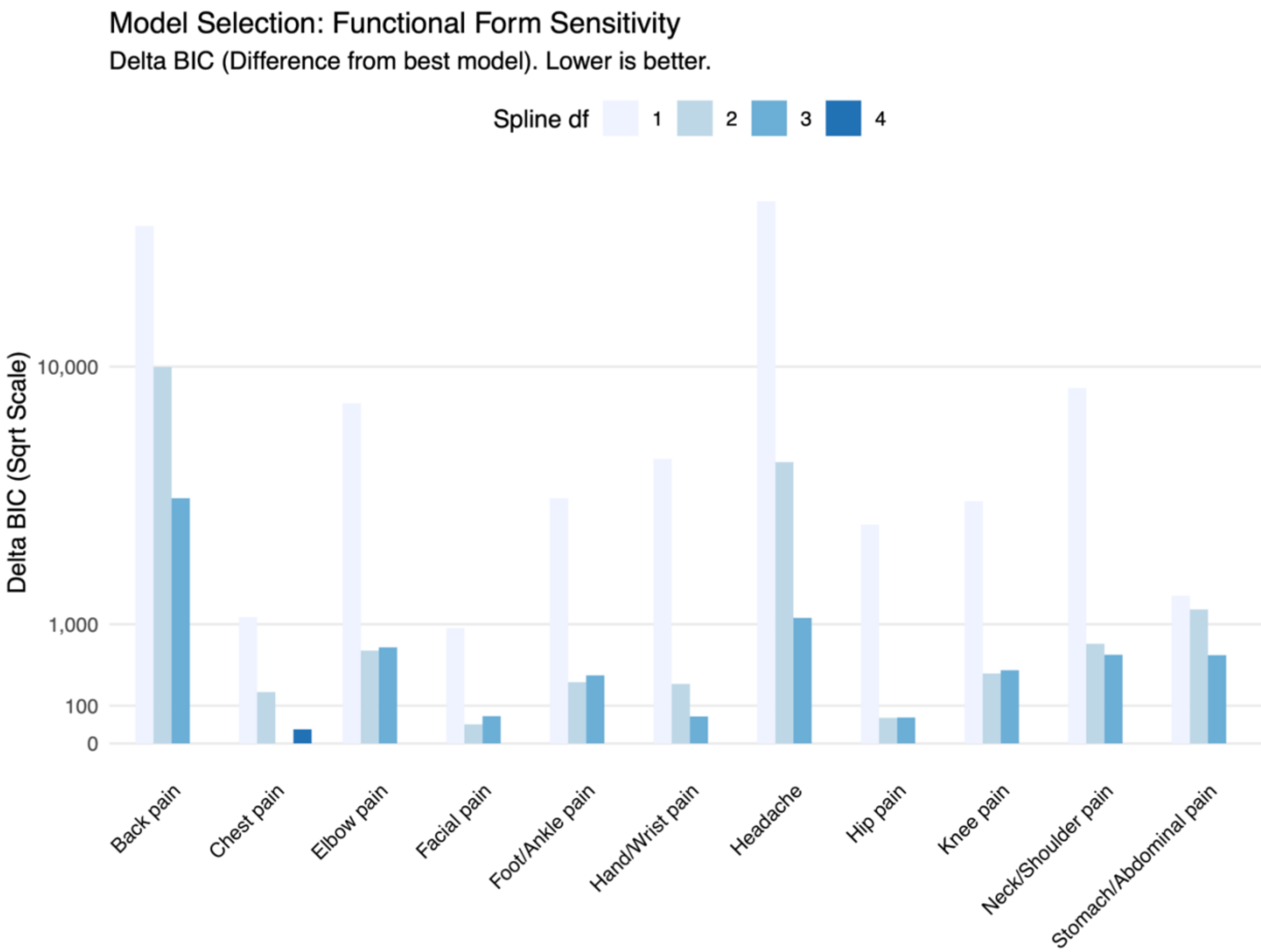

Change in Bayesian Information Criterion (BIC) relative to the best-fitting model. Lower values indicate better fit. Note the square-root scale on the y-axis.

Supplementary Figure 11: Sensitivity of age trajectories to spline complexity

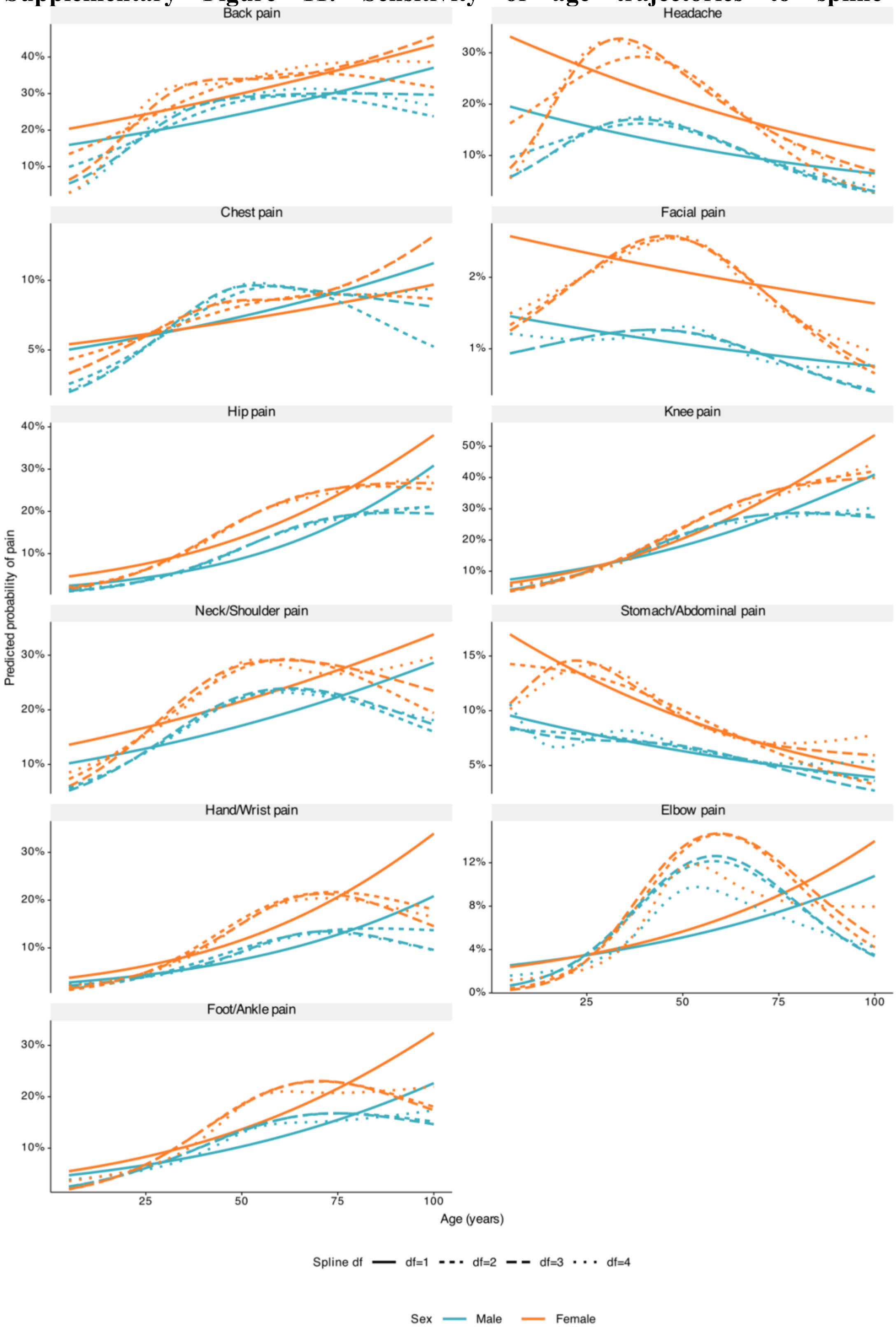

Overlaid trajectories derived from natural cubic splines with varying degrees of freedom (df=1 to df=4).

Supplementary Table 5: Model selection metrics for age-trajectory functional form

Pain site	Spline complexity	AUC	Log loss	Brier	AIC	BIC
Back pain	1	0.571	0.585	0.197	5488518.8	5488639.5
Back pain	2	0.573	0.584	0.197	5479608.5	5479755.9
Back pain	3	0.574	0.584	0.197	5473836.2	5474010.5
Back pain	4	0.574	0.585	0.197	5469576.6	5469777.7
Chest pain	1	0.562	0.309	0.082	853833.3	853942.3
Chest pain	2	0.568	0.307	0.082	852873.1	853006.3
Chest pain	3	0.569	0.307	0.082	852663.4	852820.8
Chest pain	4	0.569	0.307	0.082	852653.1	852834.7
Elbow pain	1	0.571	0.233	0.057	507045.7	507153.5
Elbow pain	2	0.618	0.233	0.058	499486.5	499618.2
Elbow pain	3	0.617	0.234	0.058	499505.2	499660.9
Elbow pain	4	0.626	0.229	0.057	498831.4	499011.1
Facial pain	1	0.616	0.149	0.034	458326.2	458436.8
Facial pain	2	0.617	0.15	0.034	457392.7	457527.9
Facial pain	3	0.617	0.15	0.034	457394.7	457554.5
Facial pain	4	0.618	0.15	0.034	457318	457502.4
Foot/Ankle pain	1	0.609	0.341	0.097	915301.6	915411.2
Foot/Ankle pain	2	0.613	0.342	0.097	911311.6	911445.6
Foot/Ankle pain	3	0.613	0.342	0.097	911349.6	911508
Foot/Ankle pain	4	0.612	0.341	0.097	910999.3	911182
Hand/Wrist pain	1	0.631	0.381	0.112	1056832.7	1056942.5
Hand/Wrist pain	2	0.634	0.379	0.112	1051358.3	1051492.5
Hand/Wrist pain	3	0.634	0.379	0.112	1051136.7	1051295.3
Hand/Wrist pain	4	0.635	0.379	0.112	1051060.9	1051244
Headache	1	0.609	0.499	0.161	3514562.9	3514680.8
Headache	2	0.618	0.505	0.163	3499415.9	3499560
Headache	3	0.616	0.511	0.166	3494926.7	3495097
Headache	4	0.616	0.509	0.165	3493791.1	3493987.6
Hip pain	1	0.63	0.327	0.092	1265875.7	1265988.6
Hip pain	2	0.633	0.331	0.093	1262526.6	1262664.6
Hip pain	3	0.633	0.331	0.093	1262502.8	1262665.9
Hip pain	4	0.634	0.33	0.093	1262430.4	1262618.6
Knee pain	1	0.615	0.489	0.156	1917582	1917694.6
Knee pain	2	0.614	0.491	0.157	1913775.1	1913912.7
Knee pain	3	0.614	0.491	0.157	1913783.3	1913945.9
Knee pain	4	0.615	0.49	0.157	1913380.9	1913568.5

Neck/Shoulder pain	1	0.565	0.566	0.186	2375098.6	2375212.5
Neck/Shoulder pain	2	0.577	0.555	0.182	2366876.2	2367015.5
Neck/Shoulder pain	3	0.578	0.554	0.182	2366706.5	2366871.1
Neck/Shoulder pain	4	0.579	0.555	0.182	2366127.7	2366317.6
Stomach/Abdominal pain	1	0.584	0.385	0.114	2042452.7	2042567.4
Stomach/Abdominal pain	2	0.582	0.386	0.114	2042155.6	2042295.8
Stomach/Abdominal pain	3	0.579	0.387	0.115	2041413.6	2041579.3
Stomach/Abdominal pain	4	0.577	0.388	0.115	2040840.5	2041031.7

Comparison of predictive performance and goodness-of-fit across natural cubic splines with varying degrees of freedom (df=1–4). AUC, Log Loss, and Brier Score represent out-of-sample performance averaged across 5 cross-validation folds grouped by study. AIC and BIC represent goodness-of-fit calculated on the full dataset. AIC=Akaike Information Criterion; AUC=Area Under the Curve; BIC=Bayesian Information Criterion; df=degrees of freedom.

Section 6.6. Sensitivity Analysis of Missing Risk Factor Data

To evaluate the potential influence of selection bias arising from missing covariate data, we performed a sensitivity analysis comparing the primary complete-case approach against an imputed dataset. The primary models excluded participants with missing data on BMI, smoking status, or income. Given the scale of the pooled dataset, conventional multiple imputation (e.g., MICE) was computationally prohibitive; we therefore employed a single stochastic imputation based on a Stochastic Gradient Descent (SGD) classifier, which scales efficiently to large samples. Because single imputation does not propagate imputation uncertainty into variance estimates, this analysis was designed to assess the direction and magnitude of potential bias rather than to replace the primary complete-case framework.

The imputation model was fitted within each study separately using predictors including education level, employment status, marital status, age, sex, country, and pain site; imputed datasets were then pooled across studies before refitting the exposure models. To avoid unreliable extrapolation, studies with excessive missingness (>50%) for a given variable were excluded from the imputation process. We compared the age-adjusted Relative Risks (RR) derived from the complete-case data against those derived from the imputed data.

Results demonstrated high robustness across all exposure–outcome associations (Supplementary Figure 12). For BMI and smoking status, point estimates and 95% confidence intervals (CIs) from the imputed models substantially overlapped with those from the complete-case analysis across all pain sites. For income (Lower vs. Upper quintile), the direction and magnitude of associations were largely consistent between methods. However, for headache, hip, knee, and neck-shoulder pain the imputed models yielded slightly higher relative risk estimates than the complete-case analysis. These findings suggest that the primary complete-case results are conservative and not substantively biased by the exclusion of participants with missing covariates. Consequently, the primary complete-case analysis was retained to maintain transparency and avoid over-reliance on modeled assumptions.

Supplementary Figure 12: Sensitivity of relative risk estimates to missing data imputation

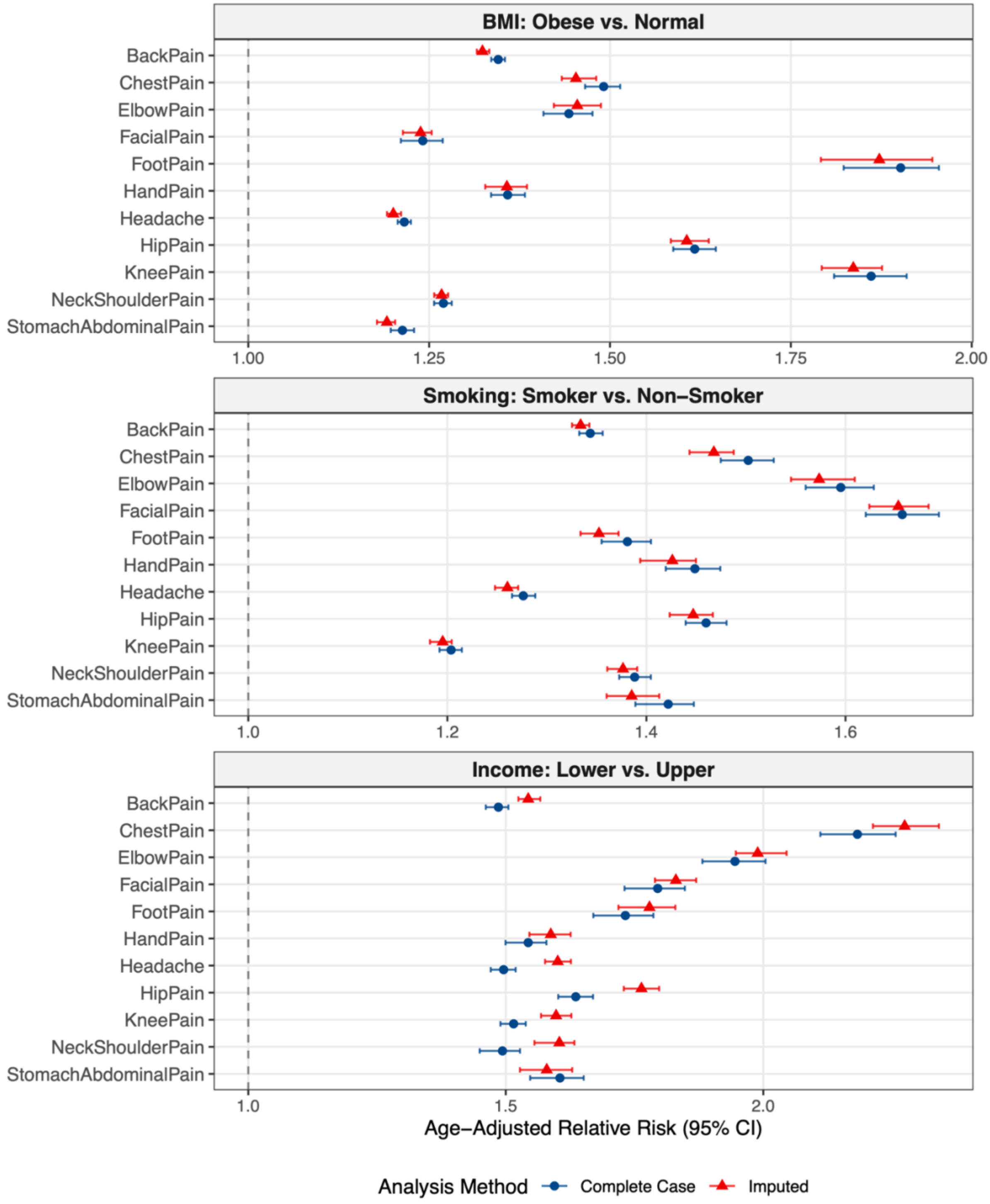

Comparison of age-adjusted Relative Risks (RR) derived from the primary Complete Case analysis (Blue circles) versus the Imputed sensitivity analysis (Red triangles). Error bars represent 95% confidence intervals.

Section 6.7. Sensitivity Analysis of Sampling Design

To evaluate whether the inclusion of volunteer and biobank-style cohorts influenced the estimated reference trajectories, we repeated the primary age-by-sex models under two restricted sampling scenarios. The first excluded sources classified as volunteer or biobank-style cohorts. The second retained probability-sampled general-population sources where sufficient data were available. Models used the same age spline, covariate adjustment, random-effects structure, and prediction procedure as the primary analyses.

Restricted-sample trajectories were compared with the primary full-data trajectory for each pain outcome. Across outcomes, the restricted trajectories closely reproduced the primary age-by-site patterns. Stomach or abdominal pain showed the largest deviation under restricted sampling, but the restricted trajectory coincided with a substantial reduction in contributing sample size from 1,489,306 participants to 676,570 participants over the age of 18. These findings indicate that the principal lifespan patterns were not driven by the inclusion of volunteer or biobank-style cohorts.

Supplementary Figure 13: Sensitivity of age-by-site trajectories to sampling design

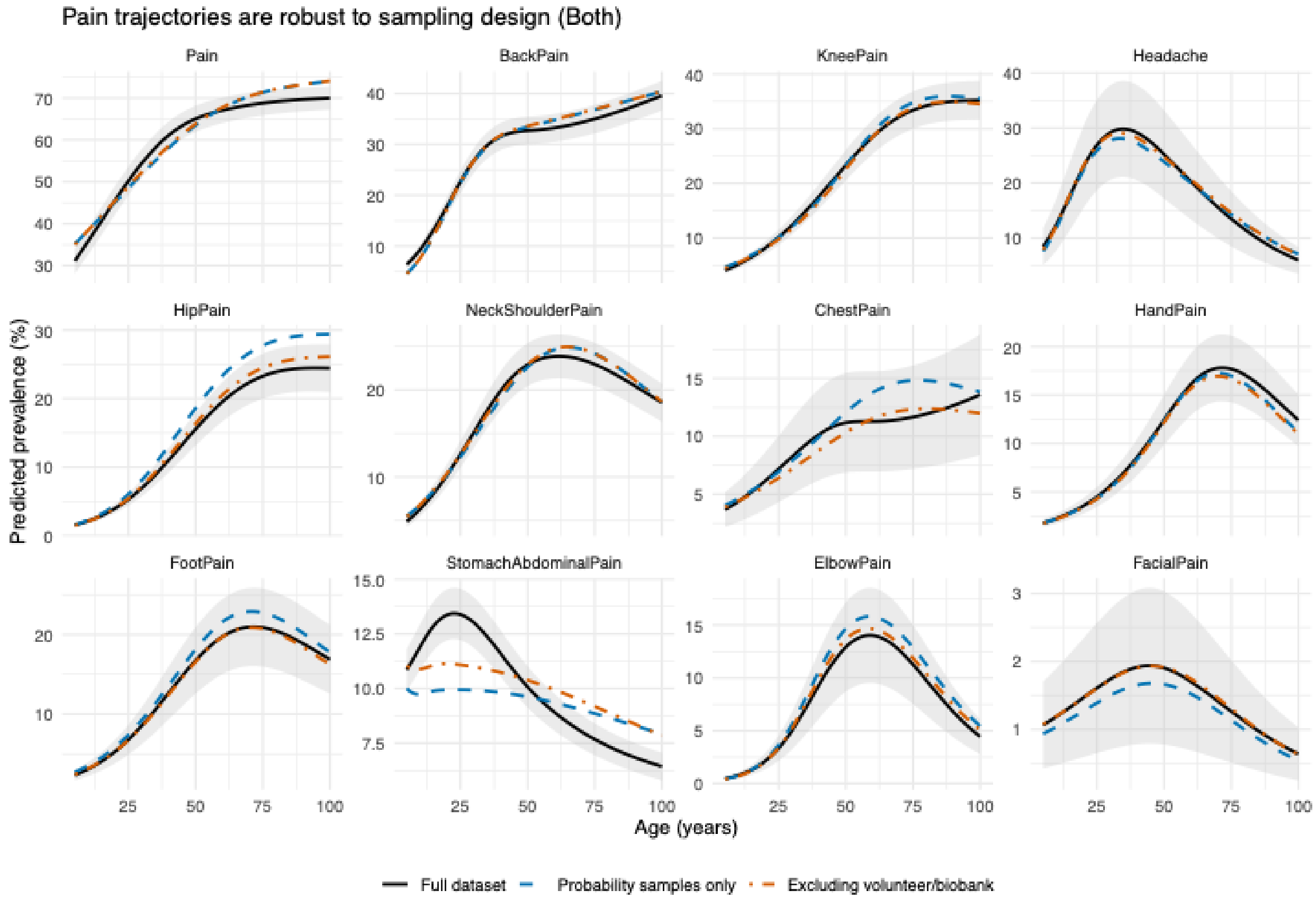

Predicted age-specific prevalence trajectories from the primary full-data model compared with restricted-sample models excluding volunteer and biobank-style cohorts, and restricting to probability-sampled general-population sources. Shaded bands indicate the 95% confidence intervals for the primary full-data trajectory.

Section 6.8. Sensitivity analysis: HDI–survey-year common support

Country-level HDI was assigned by survey year. Because lower-HDI settings were disproportionately represented in earlier survey years, HDI and survey year had limited independent overlap in the pooled dataset. For this reason, the primary HDI models did not include survey year as a covariate (Section 5.3).

To evaluate whether the HDI findings were sensitive to this choice, we performed a common-support sensitivity analysis. We restricted the data to the region in which HDI and survey year were jointly observed with sufficient overlap and refit the HDI models with survey year included. Within this common-support sample, the lower-HDI excess was preserved (Supplementary Fig. 14), indicating that the attenuation or reversal observed at younger ages in the full-data survey-year-adjusted model reflected extrapolation beyond the region of HDI–year overlap rather than a different underlying association. We therefore retained the no-survey-year HDI specification as the primary analysis.

Supplementary Figure 14: HDI sensitivity analysis under common support for survey year.

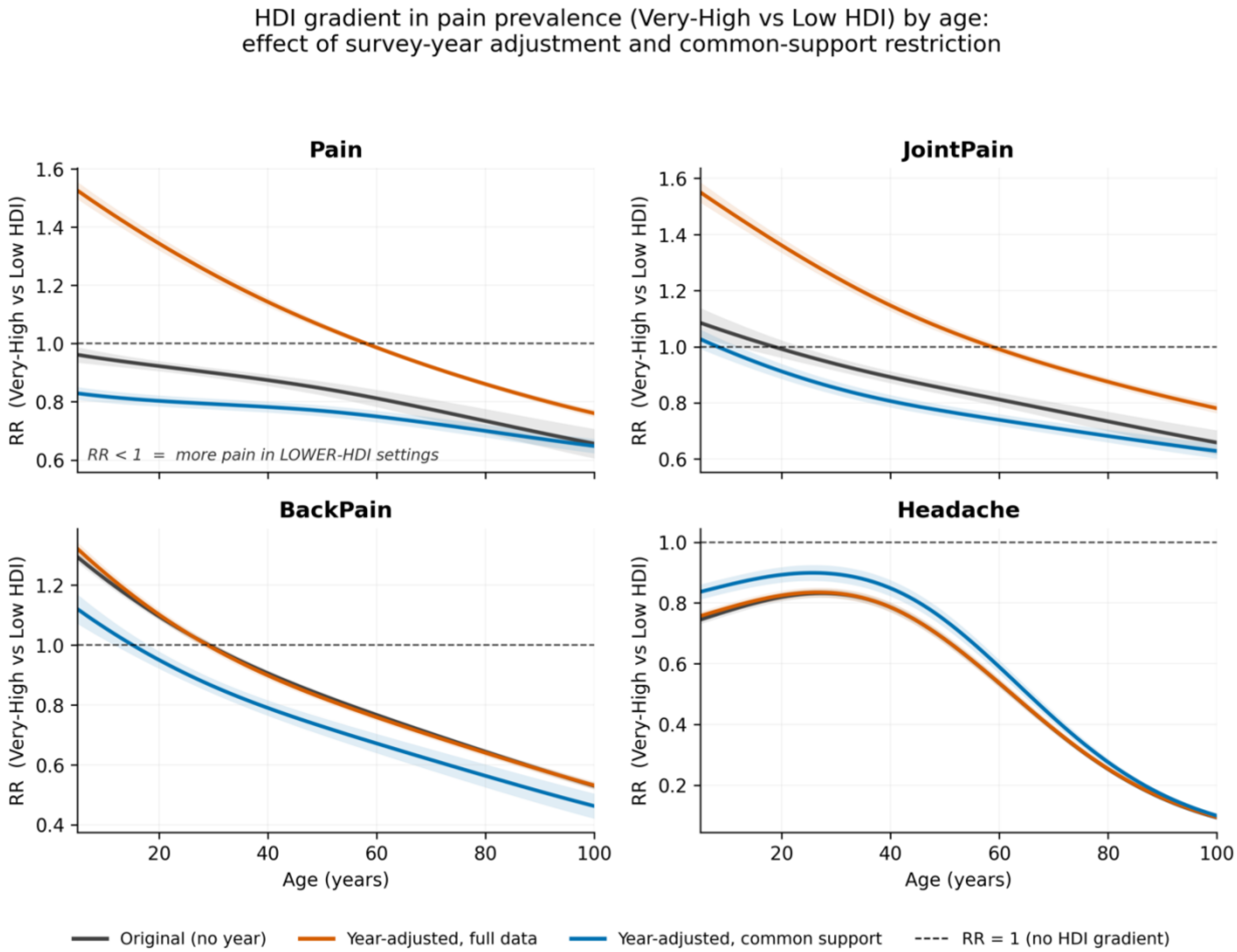

Very-high-versus-low-HDI risk ratios by age for the primary HDI model without survey-year adjustment, the full-data survey-year-adjusted model, and the survey-year-adjusted model restricted to common HDI–year support. Values below 1 indicate higher pain prevalence in lower-HDI settings. Shaded bands indicate 95% confidence intervals.

Section 7. Individual-level risk factor gradients of pain across the lifespan

Age-specific gradients in pain prevalence were estimated for sex, smoking status, body mass index category, and income quintile. For each pain site, model-predicted trajectories across age are displayed by exposure category. For smoking analyses, the analytic sample was restricted to participants aged 13 years and older, as self-reported smoking was not available in younger children. Across exposures, the strongest gradients were generally observed between early and mid-adulthood (ages 18–65), with attenuation in older age groups, particularly for smoking and income. This late-life attenuation may partly reflect selective survival, as individuals with the highest exposure burden are less likely to survive into older age, potentially underestimating the true exposure–pain association in these groups. All three exposures were associated with elevated prevalence across all pain sites. Income gradients were generally the largest in magnitude, likely reflecting its role as a proxy for multiple structural determinants of health, followed by obesity and then smoking. Obesity showed disproportionately strong associations at lower-limb sites (knee, foot, hip), consistent with cumulative mechanical load. Additionally, age-binned and age-adjusted (overall) risk ratios are provided for smoking, body mass index, and income across all pain sites, as well as sex-based risk ratios for all pain sites plus three secondary phenotypes: any bodily pain, high-intensity pain (NRS $\geq$ 7), and generalized pain.

Supplementary Table 6. Risk ratios for sex and health exposures across pain sites.

Pain site	Overall	5–17 y	18–35 y	35–50 y	50–65 y	65–80 y	80–100 y
**Sex (Female vs. Male)**							
Back	1.27 (1.26–1.28)	1.27 (1.26–1.27)	1.28 (1.27–1.29)	1.22 (1.21–1.22)	1.17 (1.17–1.18)	1.23 (1.22–1.24)	1.41 (1.39–1.43)
Knee	1.22 (1.20–1.23)	0.97 (0.93–1.01) §	1.00 (0.99–1.02) §	1.07 (1.06–1.08)	1.16 (1.16–1.17)	1.27 (1.25–1.28)	1.39 (1.35–1.43)
Headache	1.74 (1.73–1.75)	1.62 (1.61–1.64)	1.85 (1.83–1.86)	1.74 (1.73–1.76)	1.61 (1.59–1.62)	1.64 (1.62–1.66)	1.91 (1.86–1.95)
Hip	1.45 (1.42–1.49)	1.56 (1.50–1.62)	1.70 (1.66–1.74)	1.64 (1.61–1.68)	1.49 (1.46–1.52)	1.38 (1.35–1.42)	1.37 (1.32–1.42)
Neck/shoulder	1.26 (1.25–1.28)	1.21 (1.19–1.23)	1.28 (1.27–1.29)	1.28 (1.27–1.29)	1.24 (1.23–1.25)	1.24 (1.23–1.25)	1.30 (1.27–1.34)
Hand	1.52 (1.50–1.55)	0.90 (0.86–0.94)	1.31 (1.28–1.34)	1.57 (1.55–1.60)	1.62 (1.59–1.64)	1.58 (1.56–1.61)	1.54 (1.49–1.60)
Foot	1.29 (1.26–1.33)	0.93 (0.88–0.99)	1.17 (1.14–1.20)	1.32 (1.29–1.36)	1.39 (1.35–1.43)	1.36 (1.32–1.40)	1.26 (1.20–1.33)
Stomach/abdominal	1.60 (1.59–1.62)	1.52 (1.51–1.54)	1.81 (1.79–1.83)	1.59 (1.58–1.61)	1.38 (1.36–1.39)	1.42 (1.39–1.44)	1.75 (1.70–1.81)
Elbow	1.16 (1.14–1.19)	0.59 (0.54–0.64)	0.95 (0.93–0.98)	1.11 (1.09–1.13)	1.17 (1.15–1.19)	1.23 (1.20–1.26)	1.36 (1.28–1.44)
Chest	1.08 (1.06–1.10)	1.49 (1.44–1.55)	1.12 (1.09–1.14)	0.95 (0.93–0.96)	0.92 (0.91–0.93)	1.01 (0.99–1.03) §	1.27 (1.20–1.35)
Facial	1.83 (1.79–1.88)	1.44 (1.37–1.51)	1.75 (1.71–1.80)	1.97 (1.93–2.02)	2.00 (1.96–2.05)	1.93 (1.86–2.00)	1.83 (1.63–2.07)
Any bodily pain	1.16 (1.16–1.17)	1.25 (1.24–1.26)	1.20 (1.20–1.21)	1.15 (1.14–1.15)	1.11 (1.11–1.12)	1.12 (1.12–1.13)	1.18 (1.17–1.19)
High-intensity pain	1.52 (1.49–1.55)	1.57 (1.45–1.68)	1.43 (1.38–1.46)	1.31 (1.28–1.34)	1.28 (1.25–1.30)	1.50 (1.45–1.53)	2.39 (2.15–2.52)
Generalized pain	1.50 (1.46–1.54)	1.04 (0.95–1.14) §	1.23 (1.19–1.26)	1.39 (1.36–1.42)	1.51 (1.48–1.55)	1.58 (1.54–1.63)	1.61 (1.51–1.72)
**Income quintile (Lower vs. Upper)**							
Back	1.50 (1.47–1.52)	0.96 (0.94–0.99)	1.30 (1.28–1.33)	1.54 (1.50–1.59)	1.57 (1.53–1.61)	1.43 (1.39–1.46)	1.19 (1.16–1.22)
Knee	1.52 (1.49–1.55)	1.01 (0.96–1.05) §	1.35 (1.32–1.39)	1.60 (1.56–1.63)	1.59 (1.55–1.62)	1.44 (1.40–1.48)	1.22 (1.18–1.25)
Headache	1.48 (1.46–1.50)	1.07 (1.04–1.10)	1.28 (1.26–1.30)	1.48 (1.45–1.51)	1.61 (1.58–1.65)	1.66 (1.61–1.71)	1.58 (1.47–1.68)
Hip	1.63 (1.59–1.66)	1.20 (1.12–1.28)	1.57 (1.52–1.63)	1.79 (1.75–1.83)	1.71 (1.66–1.75)	1.47 (1.43–1.51)	1.15 (1.11–1.20)
Neck/shoulder	1.52 (1.48–1.54)	0.86 (0.83–0.88)	1.22 (1.21–1.24)	1.53 (1.50–1.55)	1.62 (1.59–1.65)	1.52 (1.49–1.56)	1.31 (1.27–1.36)
Hand	1.55 (1.50–1.60)	1.15 (1.06–1.24)	1.59 (1.52–1.65)	1.80 (1.73–1.87)	1.65 (1.58–1.72)	1.29 (1.25–1.33)	0.97 (0.92–1.01) §
Foot	1.74 (1.69–1.79)	0.94 (0.87–1.02) §	1.49 (1.43–1.56)	1.90 (1.81–2.00)	1.89 (1.78–2.00)	1.57 (1.49–1.64)	1.20 (1.13–1.27)
Stomach/abdominal	1.53 (1.48–1.58)	1.20 (1.14–1.26)	1.45 (1.40–1.50)	1.65 (1.59–1.71)	1.65 (1.59–1.70)	1.48 (1.41–1.54)	1.14 (1.03–1.25)
Elbow	1.95 (1.88–2.01)	1.25 (1.07–1.43)	1.65 (1.54–1.75)	1.90 (1.82–1.98)	1.98 (1.88–2.08)	1.95 (1.83–2.07)	1.83 (1.63–2.04)
Chest	2.09 (2.01–2.17)	1.39 (1.19–1.58)	1.95 (1.80–2.10)	2.39 (2.26–2.51)	2.19 (2.09–2.29)	1.69 (1.58–1.80)	0.99 (0.84–1.14) §
Facial	1.79 (1.74–1.85)	0.87 (0.77–0.96)	1.41 (1.34–1.47)	1.88 (1.81–1.94)	2.03 (1.95–2.12)	1.79 (1.70–1.88)	1.37 (1.25–1.49)

Pain site	Overall	5–17 y	18–35 y	35–50 y	50–65 y	65–80 y	80–100 y
**Smoking (Smoker vs. Never-Smoker)***							
Back	1.34 (1.29–1.37)	1.58 (1.55–1.61)	1.48 (1.46–1.50)	1.36 (1.34–1.38)	1.25 (1.24–1.27)	1.16 (1.15–1.17)	1.07 (1.05–1.09)
Knee	1.20 (1.19–1.21)	1.69 (1.65–1.74)	1.55 (1.53–1.58)	1.36 (1.34–1.38)	1.16 (1.14–1.17)	0.98 (0.96–0.99)	0.79 (0.77–0.82)
Headache	1.27 (1.25–1.28)	1.51 (1.48–1.54)	1.39 (1.37–1.41)	1.25 (1.24–1.27)	1.17 (1.15–1.18)	1.11 (1.09–1.13)	1.04 (1.00–1.09)
Hip	1.46 (1.43–1.48)	1.63 (1.52–1.73)	1.68 (1.62–1.73)	1.63 (1.60–1.66)	1.45 (1.43–1.48)	1.25 (1.22–1.27)	1.01 (0.97–1.05) §
Neck/shoulder	1.40 (1.38–1.42)	1.56 (1.53–1.60)	1.56 (1.54–1.58)	1.48 (1.46–1.50)	1.33 (1.32–1.35)	1.15 (1.13–1.17)	0.93 (0.90–0.96)
Hand	1.45 (1.42–1.48)	2.23 (2.12–2.33)	1.99 (1.94–2.04)	1.69 (1.65–1.72)	1.37 (1.35–1.40)	1.09 (1.06–1.11)	0.87 (0.83–0.91)
Foot	1.38 (1.35–1.40)	1.67 (1.58–1.77)	1.65 (1.60–1.70)	1.54 (1.50–1.58)	1.34 (1.31–1.38)	1.11 (1.08–1.14)	0.90 (0.85–0.95)
Stomach/abdominal	1.40 (1.36–1.44)	1.56 (1.49–1.62)	1.52 (1.48–1.56)	1.43 (1.40–1.46)	1.32 (1.30–1.35)	1.22 (1.18–1.26)	1.09 (1.02–1.17)
Elbow	1.59 (1.55–1.63)	1.65 (1.51–1.80)	1.83 (1.75–1.90)	1.80 (1.76–1.85)	1.52 (1.48–1.56)	1.12 (1.07–1.17)	0.77 (0.71–0.84)
Chest	1.47 (1.44–1.50)	1.31 (1.23–1.38)	1.48 (1.43–1.54)	1.61 (1.57–1.64)	1.46 (1.43–1.49)	1.19 (1.15–1.22)	0.80 (0.75–0.86)
Facial	1.67 (1.63–1.70)	2.07 (1.96–2.19)	1.94 (1.87–2.01)	1.74 (1.70–1.79)	1.48 (1.44–1.52)	1.19 (1.13–1.24)	0.90 (0.83–0.98)
**Obesity (Obese vs. Normal)**							
Back	1.35 (1.34–1.36)	1.32 (1.30–1.34)	1.32 (1.31–1.34)	1.32 (1.31–1.34)	1.34 (1.32–1.35)	1.36 (1.34–1.37)	1.39 (1.37–1.41)
Knee	1.86 (1.82–1.90)	1.02 (0.99–1.05) §	1.53 (1.50–1.55)	1.97 (1.92–2.02)	2.04 (1.97–2.11)	1.88 (1.83–1.94)	1.62 (1.57–1.66)
Headache	1.21 (1.20–1.22)	1.30 (1.28–1.32)	1.25 (1.23–1.27)	1.20 (1.18–1.22)	1.14 (1.13–1.15)	1.07 (1.05–1.08)	0.96 (0.93–0.98)
Hip	1.61 (1.57–1.66)	0.89 (0.85–0.94)	1.28 (1.25–1.31)	1.63 (1.60–1.66)	1.72 (1.67–1.76)	1.63 (1.58–1.68)	1.44 (1.40–1.49)
Neck/shoulder	1.27 (1.26–1.28)	1.01 (0.97–1.04) §	1.14 (1.12–1.16)	1.25 (1.23–1.27)	1.30 (1.28–1.32)	1.31 (1.29–1.33)	1.29 (1.26–1.32)
Hand	1.36 (1.33–1.39)	1.13 (1.07–1.20)	1.32 (1.29–1.36)	1.43 (1.39–1.46)	1.42 (1.38–1.45)	1.32 (1.28–1.36)	1.22 (1.18–1.26)
Foot	1.91 (1.82–1.97)	1.30 (1.22–1.38)	1.78 (1.71–1.84)	2.09 (2.00–2.17)	2.05 (1.95–2.15)	1.79 (1.71–1.87)	1.49 (1.43–1.56)
Stomach/abdominal	1.21 (1.19–1.23)	1.18 (1.17–1.20)	1.24 (1.22–1.26)	1.24 (1.22–1.26)	1.20 (1.18–1.22)	1.15 (1.13–1.18)	1.08 (1.03–1.12)
Elbow	1.45 (1.40–1.48)	1.18 (1.07–1.30)	1.38 (1.32–1.44)	1.47 (1.43–1.52)	1.47 (1.42–1.52)	1.40 (1.35–1.45)	1.30 (1.22–1.38)
Chest	1.48 (1.46–1.51)	0.89 (0.82–0.95)	1.21 (1.17–1.26)	1.53 (1.50–1.57)	1.56 (1.53–1.59)	1.39 (1.36–1.43)	1.08 (1.02–1.15)
Facial	1.24 (1.22–1.27)	1.03 (0.98–1.08) §	1.16 (1.13–1.19)	1.25 (1.22–1.28)	1.29 (1.26–1.32)	1.26 (1.22–1.30)	1.19 (1.12–1.26)

** For smoking, the adolescent age band corresponds to ages 13–17 years and is shown under the 5–17 column*

Age stratified risk ratios (RR) for prevalence of self-reported pain outcomes comparing female versus male, lower versus upper income quintile, smoker versus never-smoker, and obese versus normal weight. Values are RR (95% CI). The Overall column reports the age-adjusted pooled RR estimated across all ages; the age-stratified columns report age-specific RRs. § denotes comparisons not significant after Benjamini–Hochberg false-discovery-rate correction (5%)

Section 8. Population attributable fraction by region and country

Population attributable fractions (PAFs) were estimated for obesity, smoking, and low household income across 11 anatomical pain sites and stratified by world region and country. For each exposure, age- and sex-specific PAFs were computed at the country level by combining study-derived risk ratios with country-specific exposure prevalence from the Global Burden of Disease study and population denominators (Section 5.4). The combined PAF was calculated using the multiplicative independence formula. Regional and global estimates were obtained as case-weighted averages across the 11 anatomical sites.

Globally, an estimated 18.3% of the site-specific pain burden was jointly attributable to the three modifiable exposures (Supplementary Table 7). Obesity contributed the largest share in eastern Europe (13.0%), northern America (14.6%), and north Africa and west Asia (13.3%), whereas its contribution was below 5% in sub-Saharan Africa and central and southern Asia. Smoking-attributable fractions were highest in eastern Europe (8.5%) and western Europe (7.2%) and lowest in sub-Saharan Africa (2.5%). Income-attributable fractions were more uniform across regions (6.6%–8.6%), consistent with the use of within-study relative income ranking. The combined PAF ranged from 12.6% in sub-Saharan Africa to 27.1% in eastern Europe, indicating that the composition and magnitude of modifiable pain burden differ substantially across global contexts.

Supplementary Table 7: Population attributable fractions for pain by exposure and world region

Region	Obesity PAF (%)	Smoking PAF (%)	Income PAF (%)	Combined PAF (%)
**Eastern Europe**	13.0	8.5	8.6	27.1
**Northern America**	14.6	5.1	8.4	25.5
**North Africa & West Asia**	13.3	5.4	7.8	24.1
**Oceania**	11.2	6.6	8.0	23.6
**Western Europe**	10.1	7.2	8.4	23.4
**Latin America**	11.3	4.6	8.0	21.8
**East & Southeast Asia**	3.8	7.0	8.5	18.0
**Central & Southern Asia**	2.7	4.4	7.6	13.9
**Sub-Saharan Africa**	4.2	2.5	6.6	12.6
**Global**	6.3	5.5	8.0	18.3

Case-weighted population attributable fractions (PAF, %) for obesity, smoking, and low household income, averaged across 11 anatomical pain sites. The combined PAF was calculated as $1 - (1 - PAF_{obesity})(1 - PAF_{smoking})(1 - PAF_{income})$*, combined under an assumption of multiplicative independence across exposures; individual exposure-specific PAFs may therefore sum to more than the combined value. Because individuals may report pain at multiple anatomical sites, site-specific pain cases are not mutually exclusive; estimates represent the proportion of the total site-specific pain burden attributable to each exposure rather than the proportion of individuals with any pain. Obesity and smoking prevalence were obtained from the Global Burden of Disease study (obesity: GBD 2021; smoking: GBD 2015). Income was operationalised as within-study relative rank (quintiles), with a fixed prevalence of 20% per quintile across all countries.*

Section 9. Average pain intensity across the lifespan

Model-estimated average pain intensity among individuals reporting pain (NRS 1–10) followed a non-linear trajectory across the lifespan (Extended Data Figure 2). Intensity rose steeply from childhood, with the most rapid gains occurring around age 20 in both sexes, before peaking at approximately 5.0 in women and 4.6 in men around age 48. After midlife, intensity declined gradually, falling to approximately 3.5 in men and 4.0 in women by age 100. Women reported consistently higher intensity than men across the full age range, with a mean difference of approximately 0.4–0.5 NRS points that widened from early adulthood through midlife. These reference trajectories provide the distributional basis for individual-level percentile scoring in the Global Lifespan Pain Benchmarking Tool (Section 10).

Section 10. Global benchmarking framework and interactive application

To facilitate the application of the reference curves for exploratory public health surveillance, we developed the Global Lifespan Pain Benchmarking Tool — an open-source interactive web application enabling external users to benchmark independent cohort datasets against the global reference standards derived in this study, without requiring access to the individual participant data (IPD) or advanced programming expertise.

The framework addresses two fundamental questions for any external population: (1) Is the observed pain burden higher or lower than demographically expected? and (2) Is this deviation statistically meaningful given the known heterogeneity of global pain reporting? Given the substantial cultural and methodological variability inherent in self-reported pain, this tool is intended for exploratory insight and hypothesis generation rather than definitive clinical inference.

Statistical Methodology

The benchmarking tool employs a Conditional Maximum Likelihood Estimation (MLE) strategy for out-of-sample prevalence estimation. The global fixed-effects trajectories derived in the primary analysis are treated as fixed reference values. For each participant in the external dataset, an expected probability (P_{exp}) is assigned based on age, sex, recall window (short [past week], medium [past month], or long [past ≥3 months]), and case definition (chronic vs. general). A study-specific log-odds deviation (u_{new}) is then estimated via an intercept-only logistic GLM with the logit-transformed expected probabilities entered as an offset term. The resulting intercept represents the log-odds shift required to maximise the likelihood of the external data conditional on the global reference.

To determine whether an observed shift constitutes a statistical anomaly, it must be standardised against the expected between-study variance. Global pain reporting exhibits substantial heterogeneity attributable to cultural, linguistic, and methodological factors, with heavy-tailed distributions of study-level effects. To prevent extreme outliers in the reference pool from artificially widening the tolerance band for normality, between-study heterogeneity (τ) was estimated using the Median Absolute Deviation (MAD) of the study-level random effects from the primary models, scaled by the constant factor 1.4826 to provide a consistent estimator of the standard deviation under normality. The resulting standardised Z-score quantifies the deviation of the external cohort (u_{new}) relative to this heterogeneity estimate (τ) and the within-study estimation uncertainty (SE_u):

$$Z = \frac{u_{new}}{\sqrt{\tau^2 + SE_u^2}}$$

where SE_u is the standard error of the estimated offset intercept, which decreases as a function of external cohort sample size. This formulation ensures that benchmarking thresholds are defined by the central consensus of global studies, while guarding against over-interpretation of random fluctuations in small external datasets.

The tool additionally allows benchmarking of pain intensity when numeric rating scale (NRS) data are available. For cohorts reporting NRS pain scores, each participant receives a population-referenced percentile relative to the global age- and sex-specific reference distribution derived from the intensity models. Because NRS scores are bounded (1–10), right-skewed, and heteroscedastic, percentiles are estimated from a Gamma distribution parameterised using the model-derived mean intensity and the within-study coefficient of variation from the fitted reference model. The resulting percentile reflects where an individual's reported pain intensity lies relative to the expected distribution for their demographic profile.

Application Architecture and Deployment

The framework is deployed via a cloud-hosted web application [https://evppainlab.shinyapps.io/global-pain-benchmark/] which allows users to upload cohort data (CSV format) directly within their browser for instantaneous analysis; no uploaded data are stored or transmitted beyond the active session.

Interactive Visualisations and Output

The application generates a suite of automated visualisations to contextualise population-level pain burden (Extended Data Figure 1). Users may toggle between sex-stratified and combined-cohort views, and define custom demographic stratification variables.

Global Pain Scorecard: A lollipop chart reporting the magnitude of deviation (Observed-to-Expected [O/E] Ratio) and statistical significance (Z-Score) for each of the 11 pain sites simultaneously. Each site is assigned to a colour-coded deviation zone: Within Norm ($|Z| < 1.28$) indicates prevalence consistent with standard global variation; Above/Below Norm ($1.28 \leq |Z| < 1.96$) indicates deviation exceeding the central range of global heterogeneity; Far Above/Far Below Norm ($|Z| \geq 1.96$) flags a statistical outlier falling outside the 95% global prediction interval.

Anatomical Pain Profile: A polar radar chart mapping the O/E ratio across all 11 bodily sites in anatomical order (head to foot), which can be used to identify the anatomical pattern of a cohort's pain burden relative to a globally aligned expectation (O/E = 1.0).

Lifespan Trajectory Overlay: An age-prevalence fan chart superimposing the cohort's observed LOESS trend against the global reference trajectory and its 50%, 75%, and 95% prediction intervals. This visualisation allows users to identify at which life stages their cohort diverges most substantially from global norms, and whether that divergence is consistent or age-specific.

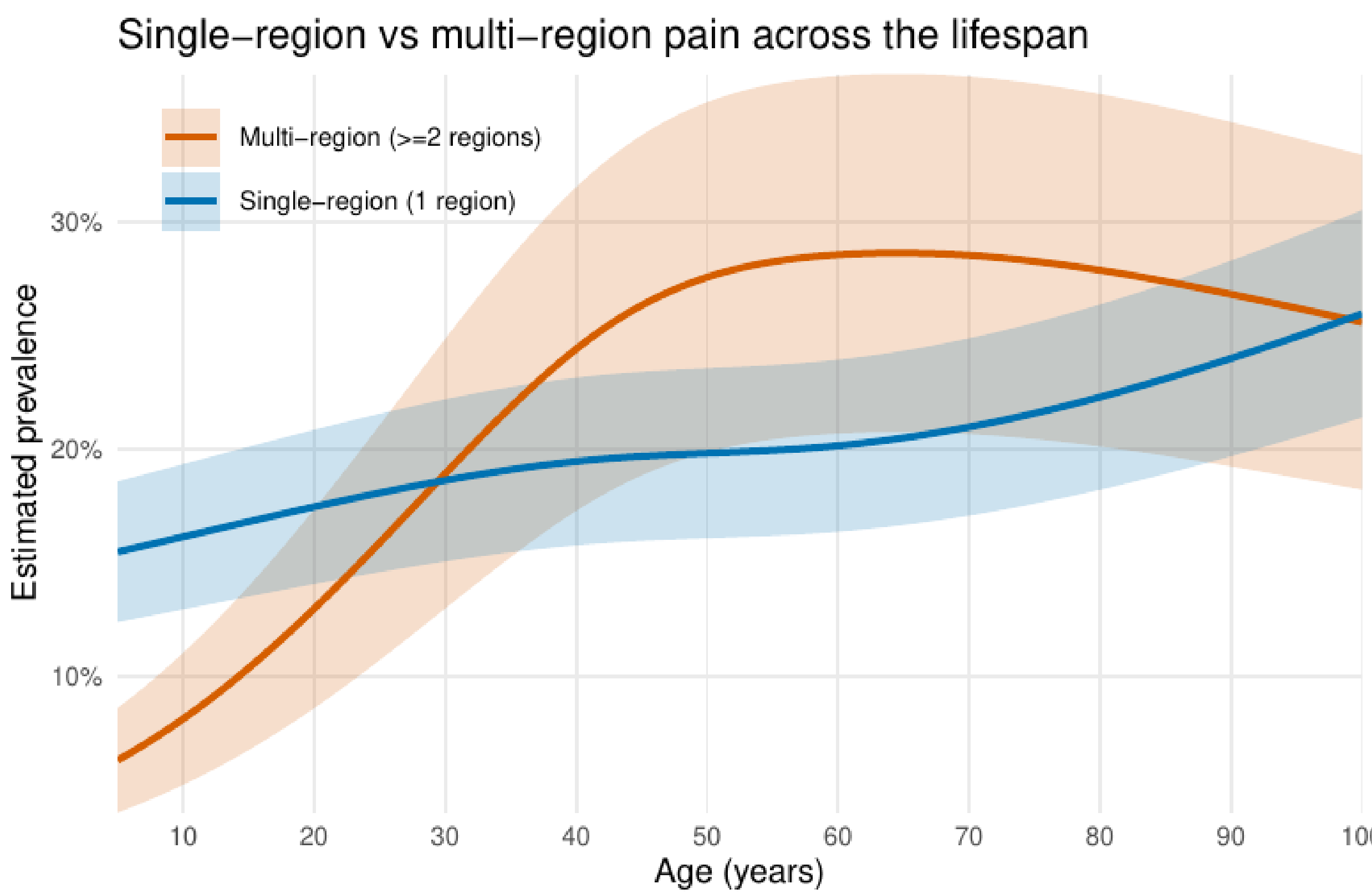

Supplementary Figure 15: Single-region versus multi-region pain across the lifespan. Model-estimated population prevalence (%) of single-region pain (pain in exactly one of seven non-overlapping anatomical regions: head/face, neck/shoulder, back, chest, abdomen, upper limb, and lower limb) and multi-region pain (pain in two or more of these regions), restricted to studies that assessed at least four of the seven regions (101 studies, n = 2,439,544). Shaded bands are 95% confidence intervals.

References

1. Zins, M., Goldberg, M., & CONSTANCES team. The French CONSTANCES population-based cohort: design, inclusion and follow-up. *Eur J Epidemiol* **30**, 1317–1328 (2015).
2. Cooklin, A. R. *et al.* Cohort Profile: Growing up in Australia: the Longitudinal Study of Australian Children (LSAC). *International Journal of Epidemiology* **54**, dyaf168 (2025).
3. Yamada, K., Matsudaira, K., Imano, H., Kitamura, A. & Iso, H. Influence of work-related psychosocial factors on the prevalence of chronic pain and quality of life in patients with chronic pain. *BMJ Open* **6**, e010356 (2016).
4. Broderick, J. E., Schneider, S., Schwartz, J. E. & Stone, A. A. Interference with activities due to pain and fatigue: accuracy of ratings across different reporting periods. *Qual Life Res* **19**, 1163–1170 (2010).
5. Broderick, J. E. *et al.* The accuracy of pain and fatigue items across different reporting periods. *Pain* **139**, 146–157 (2008).
6. Sharma, S., Kallen, M. A. & Ohrbach, R. Graded Chronic Pain Scale: Validation of 1-Month Reference Frame. *Clin J Pain* **38**, 119–131 (2021).
7. Norquist, J. M., Girman, C., Fehnel, S., DeMuro-Mercon, C. & Santanello, N. Choice of recall period for patient-reported outcome (PRO) measures: criteria for consideration. *Qual Life Res* **21**, 1013–1020 (2012).
8. Stull, D. E., Leidy, N. K., Parasuraman, B. & Chassany, O. Optimal recall periods for patient-reported outcomes: challenges and potential solutions. *Curr Med Res Opin* **25**, 929–942 (2009).
9. Nahin, R. L. Estimates of Pain Prevalence and Severity in Adults: United States, 2012. *The Journal of Pain* **16**, 769–780 (2015).
10. Fortier, I. *et al.* Maelstrom Research guidelines for rigorous retrospective data harmonization. *Int J Epidemiol* **46**, 103–105 (2017).
11. Curran, P. J. & Hussong, A. M. Integrative data analysis: the simultaneous analysis of multiple data sets. *Psychol Methods* **14**, 81–100 (2009).
12. Dworkin, R. H. *et al.* Core outcome measures for chronic pain clinical trials: IMMPACT recommendations. *Pain* **113**, 9–19 (2005).
13. Dworkin, R. H. *et al.* Interpreting the clinical importance of group differences in chronic pain clinical trials: IMMPACT recommendations. *Pain* **146**, 238–244 (2009).

14. Shi, Q., Wang, X. S., Mendoza, T. R., Pandya, K. J. & Cleeland, C. S. Assessing persistent cancer pain: a comparison of current pain ratings and pain recalled from the past week. *J Pain Symptom Manage* **37**, 168–174 (2009).

15. Wolfe, F. *et al.* 2016 Revisions to the 2010/2011 fibromyalgia diagnostic criteria. *Seminars in Arthritis and Rheumatism* **46**, 319–329 (2016).

16. Wolfe, F. *et al.* Revised chronic widespread pain criteria: development from and integration with fibromyalgia criteria. *Scandinavian Journal of Pain* **20**, 77–86 (2019).

17. Galobardes, B., Shaw, M., Lawlor, D. A., Lynch, J. W. & Davey Smith, G. Indicators of socioeconomic position (part 1). *J Epidemiol Community Health* **60**, 7–12 (2006).

18. Adler, N. E. & Newman, K. Socioeconomic disparities in health: pathways and policies. *Health Aff (Millwood)* **21**, 60–76 (2002).

19. SDG Indicators — SDG Indicators. https://unstats.un.org/sdgs/indicators/regional-groups/?utm_source=chatgpt.com.

20. BMI-for-age (5-19 years). https://www.who.int/tools/growth-reference-data-for-5to19-years/indicators/bmi-for-age.

21. Adult BMI Categories | BMI | CDC. https://www.cdc.gov/bmi/adult-calculator/bmi-categories.html?utm_source=chatgpt.com.

22. Kerr, J. A. *et al.* Global, regional, and national prevalence of child and adolescent overweight and obesity, 1990–2021, with forecasts to 2050: a forecasting study for the Global Burden of Disease Study 2021. *The Lancet* **405**, 785–812 (2025).

23. Ng, M. *et al.* Global, regional, and national prevalence of adult overweight and obesity, 1990–2021, with forecasts to 2050: a forecasting study for the Global Burden of Disease Study 2021. *The Lancet* **405**, 813–838 (2025).

24. Reitsma, M. B. *et al.* Smoking prevalence and attributable disease burden in 195 countries and territories, 1990–2015: a systematic analysis from the Global Burden of Disease Study 2015. *The Lancet* **389**, 1885–1906 (2017).

Acknowledgements and funding for contributing studies

Adolescent Brain Cognitive Development (ABCD) Study (RRID:SCR_015769). Data used in this report were obtained from the Adolescent Brain Cognitive Development Study, a multisite longitudinal study supported by the US National Institutes of Health and additional federal partners. ABCD consortium investigators designed and implemented the study and/or provided data but did not necessarily participate in the analysis or writing of this report. This manuscript reflects the views of the authors and does not necessarily reflect the views of the NIH or ABCD consortium investigators.

National Longitudinal Study of Adolescent to Adult Health (Add Health). Add Health is directed by Robert A. Hummer and funded by the National Institute on Aging cooperative agreements U01 AG071448 and U01 AG071450 at the University of North Carolina at Chapel Hill. Waves I to V data are from the Add Health Program Project, grant P01 HD31921 from the Eunice Kennedy Shriver National Institute of Child Health and Human Development, with cooperative funding from 23 other federal agencies and foundations. Add Health was designed by J. Richard Udry, Peter S. Bearman, and Kathleen Mullan Harris at the University of North Carolina at Chapel Hill.

African Population and Health Research Centre and WHO/INDEPTH Survey on Social, Health and Overall Wellbeing of Older People.

Agincourt Integrated Family Survey 2004.

Agincourt HAALSI (Health and Aging in Africa: A Longitudinal Study of an INDEPTH Community), conducted by the Harvard Center for Population and Development Studies in collaboration with the MRC/Wits Agincourt Unit, University of the Witwatersrand, South Africa.

Australian Longitudinal Study on Ageing (ALSA), coordinated by Flinders University and supported by the Australian National Health & Medical Research Council.

Austrian Health Interview Survey (ATHIS), provided by Statistics Austria.

Bangladesh Urban Health Survey (UHS), implemented by the National Institute of Population Research and Training (NIPORT), Measure Evaluation, and icddr,b.

Brazilian Longitudinal Study of Aging (ELSI-Brazil). ELSI-Brazil was approved by the Fundação Oswaldo Cruz (FIOCRUZ) ethics committee (protocol number 34649814.3.0000.5091) and supported by the Brazilian Ministry of Health: DECIT/SCTIE (Grants 404965/2012-1 and TED 28/2017) and COPID/DECIV/SAPS (Grants 20836, 22566, 23700, 25560, 25552, and 27510)

Brazil National Survey of Health (Pesquisa Nacional de Saúde, PNS), conducted by the Brazilian Institute of Geography and Statistics (IBGE) in partnership with the Ministry of Health.

Brazilian population-based chronic pain survey. de Souza JB, Grossmann E, Perissinotti DMN, de Oliveira Junior JO, da Fonseca PRB, Posso IP. Prevalence of Chronic Pain, Treatments, Perception,

and Interference on Life Activities: Brazilian Population-Based Survey. Pain Res Manag. 2017;2017:4643830. doi: 10.1155/2017/4643830. Epub 2017 Sep 26. PMID: 29081680; PMCID: PMC5634600.

The BRINCA (*estudo* ***BR****asileiro de dor* ***IN****capacitante em* ***C****rianças e* ***A****dolescentes)* project was founded by the Sao Paulo Research Foundation (FAPESP 2017/17484-1).

Cape Area Panel Study (CAPS), a longitudinal survey of adolescents and young adults in metropolitan Cape Town, conducted by the University of Cape Town and the University of Michigan, and distributed by ICPSR.

Central Statistics Division of Tuvalu, Household Income and Expenditure Survey 2022 (HIES 2022), Version 1 of the licensed dataset (May 2025), provided by the Pacific Data Hub-Microdata Library.

Child to Adult Transition Study, Murdoch Children's Research Institute, Melbourne, Victoria, Australia.

Chile Ministry of Health health surveys. We acknowledge the Department of Epidemiology, Ministry of Health of Chile, and the Undersecretariat of Public Health for providing access to the databases. All results obtained from these data are the sole responsibility of the authors and do not in any way represent the views of the Ministry of Health.

The CoLaus|PsyCoLaus study a population-based cohort study led by Lausanne University Hospital, Switzerland, was supported by unrestricted research grants from GlaxoSmithKline, the Faculty of Biology and Medicine of Lausanne University, the Swiss National Science Foundation (grants 3200B0–105993, 3200B0-118308, 33CSCO-122661, 33CS30-139468, 33CS30-148401, 33CS30_177535, 324730_204523 and 320030_220190) and the Swiss Personalized Health Network (grant 2018DRI01).

Community-Led Total Sanitation and Hygiene (CLTSH) program in Ethiopia, an initiative of the Government of Ethiopia's Federal Ministry of Health.

Collaborative Psychiatric Epidemiology Surveys (CPES), including NCS-R, NSAL, and NLAAS, collected in the United States and distributed by ICPSR.

CONSTANCES cohort, coordinated by Inserm, Université Paris Cité, Université Paris Saclay, and UVSQ. Funding reference number ANR-11-INBS-0002.

COPCORD-LATAM Group (Community Oriented Programme for Control of Rheumatic Diseases-Latin America Group) and GLADERPO (Latin American Group for Studying Rheumatic Diseases in Indigenous Peoples)

Demographic and Health Surveys (DHS) Program, implemented by ICF International and national statistical offices and funded by USAID.

Epidemiology of Neuropathic Pain in Canada study was conducted through Queen's University School of Nursing and the Department of Anesthesiology and Perioperative Medicine in Kingston, Canada. The survey was piloted in September 2011, and data collection for the main study occurred between November 2011 and September 2012. It was funded by Pfizer Canada.

English Longitudinal Study of Ageing (ELSA), coordinated by University College London, the Institute for Fiscal Studies, the University of Manchester, and NatCen Social Research, with funding from UK government departments and the National Institute on Aging (USA).

Eurobarometer Surveys, conducted on behalf of the European Commission and coordinated by Kantar Public and other research agencies, and distributed by GESIS.

European Social Survey (ESS), funded by the European Commission, the European Science Foundation, and national funding bodies, with data distribution by NSD (Norwegian Centre for Research Data).

Filipino American Community Epidemiological Study (FACES), conducted in the United States.

Generation Scotland. Generation Scotland received core support from the Chief Scientist Office of the Scottish Government Health Directorates (CZD/16/6) and the Scottish Funding Council (HR03006) and is currently supported by the Wellcome Trust (216767/Z/19/Z). Genotyping of the GS:SFHS samples was carried out by the Genetics Core Laboratory at the Edinburgh Clinical Research Facility, University of Edinburgh, and funded by the Medical Research Council UK and the Wellcome Trust (Strategic Award "STRatifying Resilience and Depression Longitudinally" (STRADL), 104036/Z/14/Z).

Generation XXI birth cohort (Geração 21), Porto, Portugal, coordinated by the Institute of Public Health, University of Porto.

German National Health Interview and Examination Survey 1998 (GNHIES98), conducted by the Robert Koch Institute, Germany.

Growing Up in Australia: the Longitudinal Study of Australian Children (LSAC), conducted in partnership by the Australian Government Department of Social Services and the Australian Institute of Family Studies.

HBSC (Health Behaviour in School-aged Children), a WHO collaborative cross-national study.

Health and Retirement Study (HRS), sponsored by the National Institute on Aging (U01AG009740) and conducted by the University of Michigan.

Health, Aging, and Retirement in Thailand (HART), sponsored by the Fundamental Fund, Thailand Science Research and Innovation (TSRI) and conducted by the National Institute of Development Administration (NIDA).

Household, Income and Labour Dynamics in Australia (HILDA) Survey, initiated and funded by the Australian Government Department of Social Services (DSS) and managed by the Melbourne Institute of Applied Economic and Social Research, University of Melbourne.

Ibadan Study of Ageing, conducted by the WHO Collaborating Centre for Research and Training in Mental Health, Neurosciences and Substance Abuse, Department of Psychiatry, University of Ibadan, Nigeria.

IMAGEN study, a European consortium investigating imaging genetics in adolescence, which received support from the following sources: the European Union-funded FP6 Integrated Project IMAGEN (Reinforcement-related behaviour in normal brain function and psychopathology) (LSHM-CT- 2007-037286), the Horizon 2020 funded ERC Advanced Grant 'STRATIFY' (Brain network based stratification of reinforcement-related disorders) (695313), Horizon Europe 'environMENTAL', grant no: 101057429, UK Research and Innovation (UKRI) Horizon Europe funding guarantee (10041392 and 10038599), Human Brain Project (HBP SGA 2, 785907, and HBP SGA 3, 945539), the Chinese government via the Ministry of Science and Technology (MOST). The German Center for Mental Health (DZPG), the Bundesministerium für Bildung und Forschung (BMBF grants 01GS08152; 01EV0711; Forschungsnetz AERIAL 01EE1406A, 01EE1406B; Forschungsnetz IMAC-Mind 01GL1745B), the Deutsche Forschungsgemeinschaft (DFG project numbers 458317126 [COPE], 186318919 [FOR 1617], 178833530 [SFB 940], 386691645 [NE 1383/14-1], 402170461 [TRR 265], 454245598 [IRTG 2773]), the Medical Research Foundation and Medical Research Council (grants MR/R00465X/1 and MR/S020306/1), the National Institutes of Health (NIH) funded ENIGMA-grants 5U54EB020403-05, 1R56AG058854-01 and U54 EB020403 as well as NIH R01DA049238, the National Institutes of Health, Science Foundation Ireland (16/ERCD/3797). NSFC grant 82150710554. Further support was provided by grants from: the Eranet Neuron (Grant ANR-18-NEUR00002-01– ADORe); Agence Nationale de la Recherche (Grant ANR-12-SAMA-0004 -GeBra); Assistance-Publique Hôpitaux-de-Paris and INSERM (interface grant); Paris Descartes University (Grant collaborative-project-2010); Paris Sud University (Grant IDEX-2012); Fondation de l'Avenir (Grant AP-RM-17-013); Fondation de France (Grant 00081242); Fédération pour la Recherche sur le Cerveau, and Fondation pour la Recherche Médicale (Grants DPA20140629802 and ADOLIMIS DPP20151033945); the Ile-de-France Region (Action 16700103 -grant to QIM–VEAVE, n°23002745–23002747).

Indonesian Family Life Survey (IFLS), collected by RAND Corporation in collaboration with Indonesian institutions.

Japan COVID-19 and Society Internet Survey (JACSIS). The JACSIS/JASTIS studies were supported by the Japan Society for the Promotion of Science (JSPS) KAKENHI Grants (grant numbers 16KK0059, 17H03589, 18H03062, 18H03107, 19H01073, 19H01074, 19H03860, 19K10446, 19K10671, 19K13704, 19K19439, 20H00040, 20K10467, 21H03203, 21H04856, 22H03225, 23H03160, 23K07492, 23K16245, 23K18370, and 23K09693), Health Labour

Sciences Research Grants, the Japan Agency for Medical Research and Development (AMED), and the Japan Science and Technology Agency (JST).

Japan Society and New Tobacco Internet Survey (JASTIS). The JASTIS study was supported by Health Labour Sciences Research Grants (H26-junkankitou-ippan-023, H28-junkankitou-ippan-002, H28-junkankitou-ippan-008, and H29-tokubetsu-shitei-006) and the Japan Society for the Promotion of Science (JSPS) KAKENHI Grants (15K19256 and 18H03062).

Japan Gerontological Evaluation Study (JAGES), Japan. JAGES was supported by the MEXT-Supported Program for the Strategic Research Foundation at Private Universities (2009–2013); the Japan Society for the Promotion of Science (JSPS) KAKENHI Grants (including JP15H01972, 22330172, 22390400, 23243070, 23590786, 23790710, 24390469, 24530698, 24683018, 25253052, and 26285138); Research and Development Grants for Longevity Science from the Japan Agency for Medical Research and Development (AMED); and Health Labour Sciences Research Grants.

Khayelitsha Health and Socioeconomic Study, conducted in Khayelitsha township, South Africa, by researchers at the University of Cape Town in collaboration with international partners.

KiGGS, conducted by the Robert Koch Institute, Germany.

Kiribati National Statistics Office, Household Income and Expenditure Survey 2019 (HIES 2019), Version 01 of the licensed dataset (December 2020), provided by the Pacific Data Hub - Microdata Library.

Korean General Social Survey (KGSS), conducted by Sungkyunkwan University's Survey Research Center and distributed via ICPSR.

Korean Longitudinal Study of Aging (KLoSA), conducted by the Korea Employment Information Service (KEIS) and funded by the Korean Ministry of Employment and Labor.

Longitudinal Ageing Study in India (LASI), coordinated by the International Institute for Population Sciences (IIPS), Mumbai, India.

Lifelines cohort study and biobank (North of the Netherlands). The Lifelines initiative has been made possible by subsidy from the Dutch Ministry of Health, Welfare and Sport, the Dutch Ministry of Economic Affairs, the University Medical Center Groningen, the University of Groningen, and the Provinces of Drenthe, Friesland, and Groningen.

Living Standards Measurement Study (LSMS), implemented by national statistical offices with technical support from the World Bank.

The Longitudinal Study of Ageing and Health in the Philippines (LSAHP). LSAHP was commissioned by the Economic Research Institute for ASEAN and East Asia (ERIA) in collaboration with Demographic Research and Development Foundation, Inc. (DRDF).

Manicaland General Population Cohort. Core funding for the Manicaland Cohort has been provided by the Wellcome Trust (084401/Z/07/B and 069516/Z/02/Z). UNAIDS, WHO, the European Union, the World Bank, and the Bill and Melinda Gates Foundation have provided funds for aspects of the work.

Malaysia Ageing and Retirement Survey (MARS) 2018. We thank the Social Wellbeing Research Centre (SWRC), Universiti Malaya, for permission to use the data and to publish this work.

Mexican Health and Aging Study (MHAS), conducted by the University of Texas Medical Branch (UTMB) in collaboration with the Instituto Nacional de Estadística y Geografía (INEGI) and the Instituto Nacional de Geriatría (INGER), Mexico.

Midlife in the United States (MIDUS), managed by the University of Wisconsin–Madison with funding from the National Institute on Aging.

Murakami Cohort Study, Japan. The Murakami Cohort Study was supported in part by JSPS KAKENHI Grants (Nos. JP23249035, JP40339958, and JP16K09078) and the National Cancer Center Research and Development Fund (23-A31[toku] and 26-A-2).

National Child Development Study (NCDS), a UK birth cohort study managed by the Centre for Longitudinal Studies, UCL Social Research Institute.

National Health and Aging Trends Study (NHATS), supported by the National Institute on Aging (U01AG032947) and conducted by the Johns Hopkins University Bloomberg School of Public Health.

National Health and Nutrition Examination Survey (NHANES), conducted by the U.S. Centers for Disease Control and Prevention, National Center for Health Statistics.

National Health Interview Survey (NHIS), conducted by the U.S. National Center for Health Statistics, Centers for Disease Control and Prevention.

National Health Measurement Study (NHMS), a national telephone survey of older adults (2005–2006) distributed by ICPSR.

National Income Dynamics Study (NIDS), implemented by the Southern Africa Labour and Development Research Unit (SALDRU) at the University of Cape Town and funded by the South African Presidency.

National Survey of Health and Nutrition (ENSANUT) 2000, conducted by the National Institute of Public Health (INSP) of Mexico.

National Survey of Self-Care and Aging (NSSA), United States, distributed by ICPSR.

Netherlands Study of Depression and Anxiety (NESDA), coordinated by Amsterdam UMC, The Netherlands.

New Zealand Health Survey (NZHS), conducted by the New Zealand Ministry of Health.

Ontario Health Study (OHS). We thank the participants in the Ontario Health Study. The data and biosamples used for this research were made available by the OHS with financial support from the Canadian Partnership Against Cancer, Health Canada, the Ontario Institute for Cancer Research, and the Government of Ontario. The views expressed herein are those of the authors and do not necessarily represent the views of these organizations.

PREVENT-AD open science dataset. Data were obtained from the Pre-symptomatic Evaluation of Experimental or Novel Treatments for Alzheimer's Disease (PREVENT-AD) program (StoP-AD Centre, Douglas Mental Health University Institute, McGill University). PREVENT-AD was launched in 2011 as a $13.5 million, 7-year public–private partnership using funds provided by McGill University, the Fonds de Recherche du Québec – Santé (FRQ-S), an unrestricted research grant from Pfizer Canada, the Levesque Foundation, the Douglas Hospital Research Centre and Foundation, the Government of Canada, and the Canada Foundation for Innovation. A complete listing of the PREVENT-AD Research Group can be found in the PREVENT-AD database (https://preventad.loris.ca/acknowledgements/).

Puerto Rican Elderly: Health Conditions (PREHCO) Project, funded by Grant R01 AG16209-01A2 from the National Institute on Aging (NIA).

Sacramento Area Latino Study on Aging (SALSA), conducted by the University of California, Davis.

South African National Demographic Survey 2016, conducted by Statistics South Africa.

Survey of Health, Ageing and Retirement in Europe (SHARE), coordinated by the Munich Center for the Economics of Aging, Max Planck Institute for Social Law and Social Policy. The SHARE data collection has been funded by the European Commission through Horizon 2020 (SHARE-DEV3: GA N°676536, SHARE-COHESION: GA N°870628, SERISS: GA N°654221, SSHOC: GA N°823782, SHARE-COVID19: GA N°101015924) and earlier EU Framework Programmes (FP5, FP6, and FP7); by DG Employment, Social Affairs & Inclusion through VS 2015/0195, VS 2016/0135, VS 2018/0285, VS 2019/0332, VS 2020/0313, SHARE-EUCOV: GA N°101052589, and EUCOVII: GA N°101102412; and by the U.S. National Institute on Aging (U01_AG09740-13S2, P01_AG005842, P01_AG08291, P30_AG12815, R21_AG025169, Y1-AG-4553-01, IAG_BSR06-11, OGHA_04-064, HHSN271201300071C, and RAG052527A), the German Federal Ministry of Education and Research, the Max Planck Society, and various national sources. The complete funding statement is available at www.share-project.org.

Survey on Aging in Latin America and the Caribbean (SABE), coordinated by the Pan American Health Organization (PAHO) and national statistical agencies.

Swedish Health Assets Project (SHA) 2008, coordinated by Swedish research institutions.

Taiwan MJ Health Resource / MJ Biobank, collected through MJ Health Management Institution, Taipei, Taiwan.

Population-based survey of musculoskeletal pain among adolescents in Thailand conducted by collaborating investigators.

Understanding America Study (UAS), maintained by the Center for Economic and Social Research (CESR) at the University of Southern California. The content of this paper is solely the responsibility of the authors and does not necessarily represent the official views of USC or UAS.

United Kingdom Biobank resource under Application Number 45551.

WHO World Health Survey (WHS), coordinated by the World Health Organization.

Encuesta Europea de Salud en España (EESE, Spanish Health Survey), conducted by the Spanish National Statistics Institute (Instituto Nacional de Estadística, INE) in collaboration with the Ministry of Health.

Mexico National Survey on Household Living Standards (ENNVIH), implemented in Mexico and distributed for research use.

ENRICA study (nutrition and cardiovascular risk in Spain, Estudio de Nutrición y Riesgo Cardiovascular en España).

Wisconsin Longitudinal Study (WLS), a long-term study of Wisconsin high school graduates and their siblings, coordinated by the University of Wisconsin–Madison.

The Trøndelag Health Study (HUNT), Norway, coordinated by HUNT Research Centre (NTNU) in collaboration with local and national health authorities.

Alameda County Health and Ways of Living Study (1994 and 1995), United States.

National Social Life, Health, and Aging Project (NSHAP) Wave 2 (2010), coordinated by the University of Chicago and NORC at the University of Chicago.

OPPERA (Orofacial Pain: Prospective Evaluation and Risk Assessment) study.

The Spanish Longitudinal Study on Ageing and Health (Ageing and Health). The Spanish Longitudinal Study on Ageing and Health (Ageing and Health) was supported by the European Community's Seventh Framework Programme (FP7/2007–2013) under Grant Agreement No. 223071 (COURAGE in Europe); the Spanish Ministry of Science and Innovation ACI-Promociona (Grant No. ACI2009–1010); the Institute of Health Carlos III-FIS (Grant Nos. PS09/00295, PS09/01845, PI12/01490, PI13/00059, PI16/00218, PI16/00173, PI19/00150, PI19/00103, PI19/00088, PI19/00235, PI22/00309, PI22/00340, PI22/00374, and PI22/00375); and CIBER – Consorcio Centro de Investigación Biomédica en Red (CIBER), Mental Health Network, Institute of Health Carlos III, Ministry of Science and Innovation. Projects PI12/01490, PI13/00059, PI16/00218, PI16/00173, PI19/00150, PI19/00103, PI19/00088, PI19/00235, PI22/00309,

PI22/00340, PI22/00374, and PI22/00375 were co-funded by the European Union through the European Regional Development Fund (ERDF) under the programme “A Way to Build Europe”.

Tonga Statistics Department, Household Income and Expenditure Survey 2021 (HIES 2021) version 01 of the licensed dataset (October 2022), provided by the Microdata Library.

Living in Switzerland panel (Swiss Household Panel, SHP Group, Living in Switzerland Waves 1–23 plus COVID-19 data), distributed by FORS (Swiss Centre of Expertise in the Social Sciences).

Sixth European Working Conditions Survey, coordinated by Eurofound.

The Yucatan Aging Study was supported by funding from the State of Yucatan and by Grants R01AG035008, P01AG022481, and R21AG033312 from the National Institute on Aging and various units at the RAND Corporation.

All of Us Research Program (AoU). Data were provided by the All of Us Research Program, supported by the US National Institutes of Health. We gratefully acknowledge All of Us participants for their contributions, without whom this research would not have been possible.

Brazilian Longitudinal Study of Adult Health (ELSA-Brasil), supported by the Brazilian Ministry of Health (Department of Science and Technology) and the Ministry of Science, Technology and Innovation (FINEP and CNPq).

Chilean Gender and Sexuality Survey (ENSSEX), conducted by the Government of Chile.

Chilean Workers Survey (ENETS), conducted by the Government of Chile.

China Health and Retirement Longitudinal Study (CHARLS), conducted by the National School of Development and China Center for Economic Research, Peking University. CHARLS was supported by the U.S. National Institute on Aging (grant numbers R01AG037031 and R01AG053228), the National Natural Science Foundation of China (grant numbers 71450001, 71603013, and 71873010), the World Bank (contract number 7172961), the China Medical Board (grant number 16-249), and Peking University.

Gender and Adolescence: Global Evidence (GAGE), a longitudinal research programme led by the Overseas Development Institute (ODI), with data collected in Bangladesh, Ethiopia, and Jordan, funded by UK aid from the Foreign, Commonwealth & Development Office (FCDO).

Korean National Health and Nutrition Examination Survey (KNHANES), conducted by the Korea Disease Control and Prevention Agency (KDCA).

WHO Multi-Country Survey Study (MCSS), coordinated by the World Health Organization.

WHO STEPwise approach to noncommunicable disease risk factor surveillance (STEPS), Nepal, coordinated by the World Health Organization.

WHO Study on Global Ageing and Adult Health (SAGE), coordinated by the World Health Organization.

WHO World Health Survey Plus (WHS+), Cambodia, coordinated by the World Health Organization.

Young Lives, an international study of childhood poverty coordinated by the University of Oxford, following cohorts in Ethiopia, India, Peru, and Vietnam, funded by UK aid from the Foreign, Commonwealth & Development Office (FCDO).
