# Supplementary material for: Global and regional reference curves for pain across the lifespan in 6.1 million individuals in 118 countries
Source: Nat Med. 2026 Oct 5;32(11):4298–306. doi: 10.1038/s41591-026-04696-w (PMC13768165; doi:10.1038/s41591-026-04696-w)
Supplement: Supplementary file 2 [file 41591_2026_4696_MOESM2_ESM.pdf]

nature portfolio

Corresponding author(s): Matt Fillingim, Etienne Vachon-Presseau

Last updated by author(s): Aug 30, 2026

Reporting Summary

Nature Portfolio wishes to improve the reproducibility of the work that we publish. This form provides structure for consistency and transparency in reporting. For further information on Nature Portfolio policies, see our Editorial Policies and the Editorial Policy Checklist.

Statistics

For all statistical analyses, confirm that the following items are present in the figure legend, table legend, main text, or Methods section.

n/a	Confirmed	
☐	☒	The exact sample size (*n*) for each experimental group/condition, given as a discrete number and unit of measurement
☐	☒	A statement on whether measurements were taken from distinct samples or whether the same sample was measured repeatedly
☐	☒	The statistical test(s) used AND whether they are one- or two-sided<br>*Only common tests should be described solely by name; describe more complex techniques in the Methods section.*
☐	☒	A description of all covariates tested
☐	☒	A description of any assumptions or corrections, such as tests of normality and adjustment for multiple comparisons
☐	☒	A full description of the statistical parameters including central tendency (e.g. means) or other basic estimates (e.g. regression coefficient) AND variation (e.g. standard deviation) or associated estimates of uncertainty (e.g. confidence intervals)
☐	☒	For null hypothesis testing, the test statistic (e.g. *F*, *t*, *r*) with confidence intervals, effect sizes, degrees of freedom and *P* value noted<br>*Give P values as exact values whenever suitable.*
☒	☐	For Bayesian analysis, information on the choice of priors and Markov chain Monte Carlo settings
☐	☒	For hierarchical and complex designs, identification of the appropriate level for tests and full reporting of outcomes
☐	☒	Estimates of effect sizes (e.g. Cohen's *d*, Pearson's *r*), indicating how they were calculated

Our web collection on statistics for biologists contains articles on many of the points above.

Software and code

Policy information about availability of computer code

Data collection	No custom software was used for primary data collection; datasets were obtained from existing population-based surveys, cohorts, and public microdata repositories. Data were assembled and managed in Python and R.
Data analysis	Statistical analyses were performed in R version 4.4.1 using lme4 1.1-35.5, glmmTMB 1.1.11, marginaleffects 0.28.0, emmeans 1.10.4, clubSandwich 0.6.1, data.table 1.17.8, dplyr 1.1.4, tidyr 1.3.1, readr 2.1.5, forcats 1.0.0, MASS 7.3-60.2, splines 4.4.1, foreach 1.5.2, doParallel 1.0.17, parallel 4.4.1, ggplot2 3.5.2, cowplot 1.2.0, patchwork 1.3.0, gridExtra 2.3, ggthemes 5.1.0, RColorBrewer 1.1-3, scales 1.4.0 and merTools 0.6.2. Country-level population attributable fraction calculations and geographical visualizations were performed in Python 3.8.13 using pandas 2.0.3, NumPy 1.21.6, GeoPandas 0.13.2 and Matplotlib 3.7.1.

For manuscripts utilizing custom algorithms or software that are central to the research but not yet described in published literature, software must be made available to editors and reviewers. We strongly encourage code deposition in a community repository (e.g. GitHub). See the Nature Portfolio guidelines for submitting code & software for further information.

Data

Policy information about availability of data

All manuscripts must include a data availability statement. This statement should provide the following information, where applicable:

- Accession codes, unique identifiers, or web links for publicly available datasets
- A description of any restrictions on data availability
- For clinical datasets or third party data, please ensure that the statement adheres to our policy

Annotated analysis code is publicly available via GitHub (https://github.com/EVPlab/lifespan-pain-trajectories). An interactive benchmarking tool and downloadable summary statistics are available at https://evppainlab.shinyapps.io/global-pain-benchmark/. The original individual-level data cannot be shared by the authors; researchers should apply directly to the respective data custodians according to their access policies. A full list of the 138 study programmes / 902 data sources is given in Supplementary Table 2.

Research involving human participants, their data, or biological material

Policy information about studies with human participants or human data. See also policy information about sex, gender (identity/presentation), and sexual orientation and race, ethnicity and racism.

Reporting on sex and gender	Sex was self-reported within each contributing study; "sex" denotes the male/female classification recorded by each source study. Gender was not separately assessed and is not claimed. Sex was considered throughout the study design: it was included as a covariate in all models and analysed as the primary exposure in the sex-comparison analyses (Figs 2–3; relative risks by sex in Supplementary Table 6). Results are reported disaggregated by sex.
Reporting on race, ethnicity, or other socially relevant groupings	individual race/ethnicity wasn't used as an analytic variable (not consistently collected or harmonisable across the source studies); populations are instead grouped by country/world region, HDI, and within-study income quintiles, all defined a priori.
Population characteristics	6,125,459 community-dwelling individuals spanning ages 5 to 100+ (ages <5 and >100 bottom/top-coded), both sexes, from 118 countries and territories, drawn from population-based national, regional, and international surveys and cohorts conducted 1990–2025. Covariates include age, sex, BMI category, smoking status, household-income quintile, Human Development Index, and world region. Detailed characteristics are in Supplementary Table 1.
Recruitment	Participants were not recruited by the study team; this is a secondary pooled analysis of existing population-based studies, each of which recruited community-dwelling participants under its own sampling design (Supplementary Table 2). Studies were eligible if they sampled community-dwelling participants without selection on health status and assessed pain by self-report. Self-selection and non-response biases inherent to the source surveys are mitigated by the use of many population-based probability samples and by study/region random effects; potential residual bias is addressed in sensitivity analyses (Supplementary Section 6).
Ethics oversight	The pooled analysis was approved by the McGill University Faculty of Medicine and Health Sciences Research Ethics Board (IRB Study Number A12-M69-23B; 23-10-068). The work is a secondary analysis of de-identified individual-level data obtained from public repositories and through data transfer/data-use agreements; each contributing study obtained its own ethical approvals and participant consent.

Note that full information on the approval of the study protocol must also be provided in the manuscript.

Field-specific reporting

Please select the one below that is the best fit for your research. If you are not sure, read the appropriate sections before making your selection.

☒ Life sciences ☐ Behavioural & social sciences ☐ Ecological, evolutionary & environmental sciences

For a reference copy of the document with all sections, see nature.com/documents/nr-reporting-summary-flat.pdf

Life sciences study design

All studies must disclose on these points even when the disclosure is negative.

Sample size	No statistical method was used to predetermine sample size. We compiled all eligible population-based datasets that could be identified and harmonised.
Data exclusions	Exclusion criteria were pre-established. Studies were excluded if they relied on clinician, observational, or other proxy reports (parent-report was accepted only for young children below the age of reliable self-report) or did not measure a target pain outcome. At the participant level, observations lacking a valid pain outcome were excluded and ages <5 and >100 were bottom/top-coded. No data were excluded post hoc to influence results.
Replication	Robustness was established through sensitivity analyses including trajectory stability across studies, methodological covariates, recall periods, random-effects structures, functional form, data missingness, and sampling design (Supplementary Section 6), plus leave-one-study-out

analyses. Findings were consistent across these checks. Experimental replication is not applicable to this observational design.

Randomization	Not applicable. This was an observational secondary analysis of existing datasets; participants were not assigned to experimental groups. Measured covariates and clustering by study and world region were addressed statistically as described in the Methods.
Blinding	Not applicable. There was no intervention or experimental group allocation, and the investigators analysed existing de-identified observational data; blinding to group allocation was therefore not relevant.

Reporting for specific materials, systems and methods

We require information from authors about some types of materials, experimental systems and methods used in many studies. Here, indicate whether each material, system or method listed is relevant to your study. If you are not sure if a list item applies to your research, read the appropriate section before selecting a response.

Materials & experimental systems

n/a	Involved in the study	
☒	☐	Antibodies
☒	☐	Eukaryotic cell lines
☒	☐	Palaeontology and archaeology
☒	☐	Animals and other organisms
☒	☐	Clinical data
☒	☐	Dual use research of concern
☒	☐	Plants

Methods

n/a	Involved in the study	
☒	☐	ChIP-seq
☒	☐	Flow cytometry
☒	☐	MRI-based neuroimaging

Plants

Seed stocks	*Report on the source of all seed stocks or other plant material used. If applicable, state the seed stock centre and catalogue number. If plant specimens were collected from the field, describe the collection location, date and sampling procedures.*
Novel plant genotypes	*Describe the methods by which all novel plant genotypes were produced. This includes those generated by transgenic approaches, gene editing, chemical/radiation-based mutagenesis and hybridization. For transgenic lines, describe the transformation method, the number of independent lines analyzed and the generation upon which experiments were performed. For gene-edited lines, describe the editor used, the endogenous sequence targeted for editing, the targeting guide RNA sequence (if applicable) and how the editor was applied.*
Authentication	*Describe any authentication procedures for each seed stock used or novel genotype generated. Describe any experiments used to assess the effect of a mutation and, where applicable, how potential secondary effects (e.g. second site T-DNA insertions, mosiacism, off-target gene editing) were examined.*
