# Supplementary material for: Global and regional reference curves for pain across the lifespan in 6.1 million individuals in 118 countries
Source: Nat Med. 2026 Oct 5;32(11):4298–306. doi: 10.1038/s41591-026-04696-w (PMC13768165; doi:10.1038/s41591-026-04696-w)
Supplement: Supplementary file 3 [file 41591_2026_4696_MOESM3_ESM.pdf]

nature portfolio

Peer Review File

Global and regional reference curves for pain across the lifespan in 6.1 million individuals in 118 countries

Corresponding Author: Dr Etienne Vachon-Presseau

This file contains all reviewer reports in order by version, followed by all author rebuttals in order by version.

Version 0:

Reviewer comments:

Reviewer #1

(Remarks to the Author)
There are many things to admire about the submitted manuscript. The writing itself is excellent. The authors made herculean efforts to identify, download and merge the data from hundreds of studies. The goal of harmonizing data across these hundreds of different studies is applauded. Nevertheless, this Reviewer has several concerns as stated below with the most critical issues first.

1. The authors seem unaware of the considerable efforts made by the WHO to develop pain questions (known as the Washington Group questions) that are culturally neutral and extensively validated in a variety of countries using their native languages: Home - The Washington Group on Disability Statistics. If available, these pain questions should be the ONLY questions for analyses in the current study, since it is known that data from these questions are comparable across languages and countries: Results of the Testing of the ESCAP/WG Extended Question Set on Disability.

2. If the Washington Group questions are not available and other pain measures (i.e., the current measures) are being incorporated into these analyses, then documentation on the validity of the pain questions for each country/language for both adults and children (if applicable) MUST be identified (even if years old) and then assessed by an INDEPENDENT panel of survey methodologists including experts in psychometrics and cognitive analyses and ideally including individuals from non-English speaking countries.. Only those surveys approved by this independent panel MAY be included in a revised analysis. To be clear, this means that pain questions without any documented validation cannot be included.

3. The inclusion of cohort studies is a major concern; no matter the effort made during recruitment and retention, except perhaps at baseline (if this baseline cohort was a TRUE random sample), these are not nationally representative samples no matter their size (e.g., UN Biobank, NIH All of Us). Regardless of the sensitivity analysis in Supplementary Figure 3, the cohort data MUST NOT be included in this analysis. I will go even farther and state that ONLY surveys based on random probability sampling should be included.

4. The authors' state: "majority of sites (7 of 11) exhibited an inverted U trajectory." There are analyses that will let you test this assumption of being inverted U-shaped such as change point detection. Without these tests you cannot make blanket statements about the shape of the trajectories.

5. I find most of the figures unreadable given the small size of individuals panels (even on my large screen monitor). Particularly problematic is the use of shading to indicate 95% CI. In many cases, it is impossible to tell if the confidence intervals overlap.

6. Relative risk values are presented to indicate differences across groups. However, 95% CIs are only presented for selected variables for selected painful conditions. I would like to see tables containing the complete relative risk data for every model employed, whether the relative risk 95% CI crosses 1. I would like to see the output from regression models for all covariates in the given model. Survey year MUST be included as a covariate in all regression models.

7. The number of comparisons must be near or over a hundred. Given this, some correction to alpha must be made.

8. There is no real table 1 showing the overall demographic characteristics of the harmonized combined data set. What are

the proportions by sex, age, region, etc. Survey year should be included.

9. I do not like stacked graphs e.g. extended data figure 1. They are impossible to read and interpret. Confidence intervals must always be presented.

10. While it is clear there is a truly international group of investigators involved with this project, the roles of these individuals are not specific. Are they all truly deserving of authorship? Do they make SUBSTANTIAL contributions? Nature Medicine lists these requirements for authorship: “each author is expected to have made substantial contributions to the conception or design of the work; or the acquisition, analysis, or interpretation of data; or the creation of new software used in the work; or have drafted the work or substantively revised it.” “Contributors who do not meet all criteria for authorship should be listed in the Acknowledgements section.”

Reviewer #2

(Remarks to the Author)
This manuscript provides population-based estimates of pain prevalence across 118 countries and over 6 million people, including data from all habituated continents. In developing their estimates, the investigators harmonized individual-level self-reported pain data across 894 population-based data sources to establish global reference trajectories of pain. The investigators report pain prevalence estimates ranging from 2.5% for facial pain to 45.0% for back pain. Estimates across all sites of pain were consistently higher among women, with prevalence estimates generally peeking before age 55. Investigators addressed hypothesis-driven key knowledge gaps and reported that pain prevalence was generally higher in lower Human Development Index (HDI) regions, and that pain burden was largely accounted for by three modifiable risk factors, namely smoking, obesity, and low income. One particularly important recommendation is to continue to systematically examine key contributors to pain in lower-HDI settings that remain poorly characterized.

This manuscript is highly significant and innovative. It has the potential to stand as a key resource that can inform global health policy, practice, and scientific inquiry related to pain and pain management by providing rigorously developed and reliable pain prevalence and burden estimates, along with important information about sex differences, age trajectories, regional variation, associations with health, education, and income, and modifiable risk factors.
This manuscript has numerous strengths and few relative weaknesses. It is well-written, concise, and clear. The focus on a limited number of significant gaps and hypotheses related to sex, age, income, and modifiable risk factors is an important asset. The background provided supports these focuses and provides a compelling rationale and context for the significance of the report. Key ethical and regulatory issues are appropriately addressed related to data sharing and data use. The authors provide a compelling rationale for the use of self-report data as opposed to data from health care records since limitations of use of health care resources in some countries and regions may seriously compromise the reliability and validity of these data. The analytic approaches are well-informed and carefully described, leading to confidence in the data and the analyses. Key limitations are noted. The conclusions are appropriate. References are appropriate.
The long-time frame (1990-2025) for the study is a strength (i.e., more data from more countries) and also a serious limitation since pain prevalence, surveillance and reporting methods, and care and care access is dynamic. This observation could be specifically noted.
More details about how data used in the study were identified and incorporated could be useful. Any risk of selection bias should be acknowledged.
The rationale for focusing on pain sites as opposed to clinical diagnostic information is appropriate and an asset. At the same time, additional details to support this decision and the sites selected could be helpful. It could have been useful to consider pain at multiple sites were managed and “whole body pain.” The concept of chronic overlapping pain conditions might be acknowledged.

The investigators note an “inverted U” for the age and pain prevalence association is an important observation, although this is not novel or unique to this study. A more detailed discussion of the varying hypotheses that could account for this observation could be included. For example, some have hypothesized a cohort effect, that is: that older persons may report more widespread pain leading to an underreporting of site-specific pain; and/or pain interference among younger persons may be more salient than pain experienced in older persons (context matters).

The rationale and empirical and conceptual support for focusing on smoking, obesity, and income relative to other variables could be provided. What about employment status or type of work? Housing security? Food insecurity?

The paragraph on limitations is important; could this be expanded with some examples of variance in the way presence of pain was measured and the approach for operationalization.

In conclusion, I consider this manuscript to be particularly strong and likely to have a major impact on the field, both in terms of the methodology and findings. Limitations are all minor and are overshadowed by many methodological and other strengths and the significance of the report.

[redacted]

Version 1:

Reviewer comments:

Reviewer #1

(Remarks to the Author)
For the most part the authors have adequately addressed many of the specific concerns expressed by this reviewer. However, I still strongly question the validity of combining data from surveys using very different questions and approaches to collect data. The authors cite they are replicating the approach taken by Global Burden of Disease Study (GBDS). Perhaps I am one of the few population-based epidemiologists who does not think the comparisons made in the GBDS are valid. I certainly never cite it. At this point it is unlikely the journal will reject this manuscript. However, I still think a large supplement should list EVERY question from EVERY country that was combined in this study, so that the reader can judge themselves as to the comparability of the survey questions.

(Remarks on code availability)
I could not find the study's information using the provided URL

Reviewer #2

(Remarks to the Author)
Thanks to the authors for a particularly comprehensive, thoughtful, and scholarly response to reviewers' concerns and recommendations on the original manuscript. I am pleased that responses reflect a high level of respect for each of the prior reviewers' comments. It is clear that in most cases, the authors have made substantial efforts to address reviewer recommendations, have accepted the reviewer concerns, and have taken steps to address them. In the few cases that the authors have decided not to pursue reviewer recommendations, I found their arguments for not pursuing recommended changes to be strongly supported by a well-articulated conceptual or empirical basis, and I cannot disagree with their decisions. In the end, I am entirely satisfied with the authors' responses to prior critiques, with the additional analyses they conducted, and with revisions to the manuscript. I have no additional questions or comments that require further response from the authors. I am very enthusiastic about the work of these authors and the revised manuscript.

(Remarks on code availability)

Open Access This Peer Review File is licensed under a Creative Commons Attribution 4.0 International License, which permits use, sharing, adaptation, distribution and reproduction in any medium or format, as long as you give appropriate credit to the original author(s) and the source, provide a link to the Creative Commons license, and indicate if changes were made.
In cases where reviewers are anonymous, credit should be given to 'Anonymous Referee' and the source.
The images or other third party material in this Peer Review File are included in the article’s Creative Commons license, unless indicated otherwise in a credit line to the material. If material is not included in the article’s Creative Commons

license and your intended use is not permitted by statutory regulation or exceeds the permitted use, you will need to obtain permission directly from the copyright holder.
To view a copy of this license, visit https://creativecommons.org/licenses/by/4.0/

Response to the Editor and Reviewers

Manuscript: *Pain across the lifespan: global and regional reference curves from 6.1 million individuals in 118 countries*

Formatting key:

Bold black = reviewer comment (verbatim).

Blue = author response.

Green box = change made to the manuscript (with location).

Response to the Editor

We thank the Editor and reviewers for their careful reading, constructive feedback, and positive assessment of the manuscript. We have revised the manuscript substantially in response to the comments and believe the revisions have strengthened the transparency, reproducibility, and interpretability of the study. We summarise the principal changes below before responding point by point.

First, we expanded and updated the harmonised dataset. Since the original submission, eight additional eligible population-based data sources became available including ELSA-Brasil, the Manicaland Cohort round 9, and KNHANES, improving coverage in regions that remain underrepresented in global pain surveillance. ELSA-Brasil and the Manicaland Cohort were already part of the planned data-sharing pipeline at submission but received approval afterwards. The revised analytic sample now includes 6,125,459 participants from 902 data sources, representing 138 study programmes across 118 countries and territories. We updated all demographic summaries, figures, tables, and in-text statistics accordingly, added a new demographic Supplementary Table 1, and now define the analytic sample explicitly as participants with at least one harmonisable pain measure together with valid age and sex. We also corrected author-list omissions and naming issues and updated the Acknowledgements and author contributions.

Second, we strengthened the primary statistical specification and reporting. In response to Reviewer 1, we added survey year as a covariate in all models, centred on the sample mean, and now report survey-year-adjusted estimates as the primary specification. This adjustment had minimal impact on estimated trajectories and did not alter the substantive conclusions. We also applied a Benjamini–Hochberg false-discovery-rate correction across the full family of reported relative-risk comparisons. To improve transparency, we now provide complete relative-risk results, fixed-effect model outputs, site-level exposure prevalence estimates, and primary age-specific prediction outputs as supplementary or machine-readable files.

Third, we added and clarified sensitivity and secondary analyses. The revised Supplement now includes sensitivity analyses covering leave-one-study-out influence, methodological covariates, recall-period restrictions, alternative random-effects structures, spline complexity, missing exposure data, and sampling design. The new sampling-design analysis compares the primary trajectories with restricted-sample models excluding volunteer and biobank-style cohorts and restricting to probability-sampled general-population sources where supported by the data. These analyses support the robustness of the principal age-by-site trajectories. We also revised Extended Data Fig. 1 to include the expected number of co-occurring pain sites with 95%

confidence intervals, added an analysis of single-region versus multi-region pain to complement the generalised-pain outcome, and clarified how inverted-U trajectory shapes were classified using age-specific rates of change.

Fourth, we revised the manuscript text to improve clarity and balance. We sharpened the rationale for using site-specific self-reported pain as a complementary framework to diagnosis-based surveillance, particularly in settings where clinical diagnostic data are sparse or health-system access is limited. We expanded the Methods to summarise the data-identification and acquisition process, clarify the harmonisation of anatomical pain categories, describe the limited use of parent-reported pain items in young children, and explain the prespecified rationale for the smoking, BMI, and income analyses. We also revised the Discussion and Limitations to address measurement heterogeneity, cross-country and cross-language variation, sampling design and representativeness, the broad 1990–2025 observation window, data-availability selection bias, and the interpretation of cross-sectional age patterns.

We are grateful to the Editor and reviewers for feedback that materially improved the manuscript. We hope the revised version addresses the concerns raised and presents a clearer, more transparent, and more useful global reference framework for self-reported pain across the lifespan.

Reviewer 1

There are many things to admire about the submitted manuscript. The writing itself is excellent. The authors made herculean efforts to identify, download and merge the data from hundreds of studies. The goal of harmonizing data across these hundreds of different studies is applauded. Nevertheless, this Reviewer has several concerns as stated below with the most critical issues first.

Response: We thank the reviewer for the generous assessment of the work and for the constructive concerns raised below, which we address point by point.

1.1

The authors seem unaware of the considerable efforts made by the WHO to develop pain questions (known as the Washington Group questions) that are culturally neutral and extensively validated in a variety of countries using their native languages: Home - The Washington Group on Disability Statistics. If available, these pain questions should be the ONLY questions for analyses in the current study, since it is known that data from these questions are comparable across languages and countries: Results of the Testing of the ESCAP/WG Extended Question Set on Disability.

Response: We thank the reviewer for highlighting the Washington Group (WG) pain items and agree that they represent an important contribution to internationally comparable disability surveillance [1]. The WG extended set assesses pain globally through two items addressing pain frequency over the previous three months and the intensity of the most recent pain episode. These items do not distinguish anatomical location or clinical phenotype. They are therefore not suitable as the sole basis for the present study, whose central aim is to estimate distinct lifespan trajectories of self-reported pain prevalence across distinct anatomical sites rather than to summarise pain using a global disability-surveillance measure.

This distinction is fundamental to the study. Headache, abdominal pain, upper-body musculoskeletal pain, and lower-body musculoskeletal pain follow clearly different age trajectories, with distinct timing of peak prevalence and peak rates of age-related change. A global pain-frequency or pain-intensity measure cannot recover these site-specific patterns. Restricting the analysis to WG items would therefore not provide a more standardized version of the present analysis, it would instead make the primary research question unanswerable and result in a substantively different study.

In addition, the WG pain items were not available across the contributing data sources in a way that could support the present analyses. The dataset was assembled from 902 population-based data sources spanning 118 countries and territories, most of which used locally or study-specific self-reported pain questions rather than WG pain items. Requiring WG items as the only admissible measure would therefore exclude nearly all of the assembled evidence base and would substantially reduce geographic, temporal, and age coverage, particularly in settings where pain surveillance is already sparse.

Our approach instead follows the logic of large-scale global harmonisation efforts [2,3], which do not generally require a single instrument across all contributing sources but instead harmonise the measures that exist. This is the approach normally used by global surveillance initiatives, such as the global burden of disease (see below). In this study, we mapped study-specific wording and body-map coding schemes to pre-specified anatomical pain categories (Supplementary Section 3; Supplementary Table 3), coded major methodological features of each source, and tested whether these features altered the estimated trajectories (Supplementary Section 6.2; Supplementary Figs. 3-5). These analyses showed that

methodological variation, including question format, valence, survey format, survey setting, and sampling strategy, had small and site-specific effects and did not alter the central age-by-site patterns; recall period and chronic-explicit case definition were retained in the primary models because they had the clearest interpretive and empirical influence. We further evaluated recall-period restrictions directly in Supplementary Section 6.3 and Supplementary Figs. 6-7.

We therefore retained the full harmonised dataset rather than restricting to WG items, which would not address the manuscript's aims. We have clarified in the limitations that cross-country and cross-language variation in self-reported pain measurement may influence absolute prevalence estimates, although the central lifespan and anatomical patterns were robust across the methodological sensitivity analyses.

Changes to the manuscript: We expanded the Discussion/Limitations to acknowledge cross-country and cross-language variation in self-reported pain measurement and the potential influence of residual differences in translation, symptom interpretation, and response behaviour on absolute prevalence estimates. We clarified that the principal age-by-site patterns were robust across the measured methodological features and recall-period sensitivity analyses, while recognising that harmonisation cannot eliminate all measurement heterogeneity. We also added clearer Methods cross-references to the anatomical harmonisation definitions and methodological sensitivity analyses in Supplementary Section 3, Supplementary Table 3, and Supplementary Sections 6.2–6.3.

Revised text (Discussion/Limitations): *Pain measures differed across studies, countries, and languages, including current, past-week, past-month, and 3- to 12-month recall periods; general versus chronic-explicit case definitions; direct site-specific questions versus body-map coding; and different anatomical resolution, such as separate sites versus grouped regions including neck/shoulder, hand/wrist, foot/ankle, or stomach/abdominal pain. We mapped these measures to pre-specified anatomical categories and evaluated methodological covariates and recall-period restrictions in sensitivity analyses (Supplementary Section 3; Supplementary Table 3; Supplementary Sections 6.2–6.3), though residual differences in translation, symptom interpretation, and response behaviour may influence absolute prevalence estimates.*

1.2

If the Washington Group questions are not available and other pain measures (i.e., the current measures) are being incorporated into these analyses, then documentation on the validity of the pain questions for each country/language for both adults and children (if applicable) MUST be identified (even if years old) and then assessed by an INDEPENDENT panel of survey methodologists including experts in psychometrics and cognitive analyses and ideally including individuals from non-English speaking countries.. Only those surveys approved by this independent panel MAY be included in a revised analysis. To be clear, this means that pain questions without any documented validation cannot be included.

Response: We appreciate the reviewer's concern that the validity and comparability of contributing pain measures should be transparent. We agree that measurement heterogeneity is an important limitation in any global harmonisation study and have expanded the manuscript accordingly.

However, we do not believe that convening an independent panel to approve or reject each item would provide a more valid or reproducible solution. There is no established protocol or accepted set of criteria for such a panel in global pain-prevalence harmonisation, and a panel could not generate validation evidence where none exists. Instead, it would introduce a subjective filtering step whose decisions may be difficult to reproduce. Moreover, expert panels

are inherently subject to disagreement, as demonstrated by longstanding debates in pain medicine where panels with similar expertise have reached conflicting recommendations on the same evidence[4,5]. A data-driven demonstration of variance across data sources, as presented in this paper, provides a more transparent and reproducible basis for evaluating measurement comparability than a subjective filtering step whose decisions would be more difficult to replicate.

We also do not believe that excluding every pain question without published country-, language-, age-, and item-specific validation would improve the validity of this analysis. Across 902 historical and contemporary data sources, many pain items were embedded in national health surveys, cohort questionnaires, community studies, or regional surveillance programmes for which formal validation evidence is unevenly available. Applying this proposed requirement would remove much of the global evidence base and would disproportionately exclude lower-resource and underrepresented settings, where validation studies and surveillance infrastructure are least available. This would likely make the dataset less globally representative, not more.

Moreover, validation is context-specific. An instrument validated in the United States or United Kingdom cannot automatically be assumed to perform equivalently in East Asia, sub-Saharan Africa, or other settings where language, literacy, cultural frameworks for reporting symptoms, and health-care context differ. Our approach therefore respects the locally selected measures used by investigators and surveillance programmes within their populations, while applying a common, transparent harmonisation framework and empirically evaluating the influence of methodological variation. This approach is consistent with large-scale global surveillance efforts, including the Global Burden of Disease study, which synthesise heterogeneous data collected using different instruments, languages, and health-system contexts rather than requiring identical measurement instruments or independent validation of every contributing item.

Our approach was therefore to use a transparent and reproducible harmonisation framework. We mapped study-specific pain questions and body-map coding schemes to pre-specified anatomical categories, excluded items that could not be mapped transparently, coded key methodological features of each source, and quantified their influence in sensitivity analyses. Anatomical mappings and inclusion criteria are documented in Supplementary Section 3 and Supplementary Table 3. Methodological sensitivity analyses are reported in Supplementary Section 6.2 and Supplementary Figs. 3-5. These included recall period, case definition, survey format, survey setting, question valence, question format, and sampling strategy. Recall period and chronic-explicit case definition were retained as model covariates, while the remaining features showed small or inconsistent effects and did not alter the age-by-site patterns. Recall-period exclusions were further evaluated in Supplementary Section 6.3 and Supplementary Figs. 6-7.

We have expanded the limitations to state more explicitly that formal validation evidence is uneven across countries, languages, and age groups, and that residual measurement heterogeneity may affect absolute prevalence estimates despite harmonisation. We also now point readers more clearly to the supplementary harmonisation definitions and methodological-covariate sensitivity analyses, which document how pain items were operationalised and how sensitive the estimated trajectories were to measurement and survey features.

Finally, we would emphasise that this project was developed and interpreted by an international consortium with expertise spanning pain epidemiology, paediatric pain, public health, cross-cultural measurement, and regional population health across the settings represented. Notably, Professors Fiona Blyth and Gary Macfarlane, both senior authors on this manuscript, currently lead an international working group of experts endorsed by the International Association for the

Study of Pain to develop a small, globally applicable core set of questions suitable for inclusion in general population surveys. The establishment of this task force reflects the recognised need for improved and internationally applicable pain-measurement strategies, a gap that the present study directly addresses. Their expertise, together with that of collaborators from the represented regions, informed the study's conception and design, identification and interpretation of relevant data sources, measurement and harmonisation decisions, and contextual interpretation of the findings.

Changes to the manuscript: We expanded the Discussion/Limitations to state that formal country-, language-, age-, and item-specific validation evidence was not uniformly available across contributing sources and that harmonisation should not be interpreted as establishing full measurement equivalence across all populations. We clarified that residual differences in translation, symptom interpretation, and response behaviour may affect absolute prevalence estimates despite transparent anatomical mapping and methodological sensitivity analyses. We also clarified in the main and Supplementary Methods that pain was assessed through direct participant self-report, with a limited exception for parent-reported pain items among young children below the age of reliable self-report.

Revised text (Methods, Data sources): *Pain was assessed by direct participant self-report, except that parent-reported pain items were accepted for young children below the age of reliable self-report in a small number of paediatric cohorts (Supplementary Section 2.2; Supplementary Table 2). Studies were excluded if they relied on clinician or observational reports, other proxy reports, or did not measure any target pain outcome.*

Revised text (Discussion/Limitations): *(see also the measurement-variation passage quoted under 1.1, noting that residual differences in translation, symptom interpretation, and response behaviour may influence absolute prevalence estimates.)*

1.3

The inclusion of cohort studies is a major concern; no matter the effort made during recruitment and retention, except perhaps at baseline (if this baseline cohort was a TRUE random sample), these are not nationally representative samples no matter their size (e.g., UN Biobank, NIH All of Us). Regardless of the sensitivity analysis in Supplementary Figure 3, the cohort data MUST NOT be included in this analysis. I will go even farther and state that ONLY surveys based on random probability sampling should be included.

Response: We agree with the reviewer that cohort studies, volunteer cohorts, and biobank samples should not be assumed to be nationally representative. We have clarified this point in the manuscript and statistically evaluated the influence of sampling design.

Importantly, the aim of this study is not to produce official nationally representative prevalence estimates for each country from a single sampling frame. Rather, the aim is to construct harmonised reference trajectories of self-reported pain across age, sex, anatomical site, and population context using the available individual-level population-based evidence. For a global lifespan analysis spanning childhood through very old age and including regions where repeated probability surveys are limited, restricting inclusion to random-probability surveys alone would substantially reduce age, geographic, and phenotypic coverage.

We therefore examined whether the main findings were sensitive to sampling design rather than assuming that all sources were equivalent. In the existing methodological-covariate sensitivity analysis, sampling strategy was explicitly coded and evaluated alongside other study-level features (Supplementary Section 6.2; Supplementary Fig. 3). Its influence on predicted prevalence was small and inconsistent across sites, and it did not alter the age-by-site trajectory patterns (Supplementary Figs. 3-5). We also note that the primary models include study-level

random intercepts and estimate marginal reference trajectories rather than simple sample-size-weighted pooled prevalences, reducing the extent to which any single large cohort or biobank can dominate the global curves. Consistent with this, the leave-one-study-out analysis showed that the global trajectories were not driven by any single study programme (Supplementary Section 6.1; Supplementary Figs. 1-2).

To further address the reviewer's concern, we have added sensitivity analyses comparing the full-data trajectories with models excluding volunteer/biobank-style cohorts and models restricted to probability or general-population samples (new Supplementary Section 6.7; Supplementary Fig. 13). These analyses showed that the core findings were materially unchanged for 11 of 12 outcomes: the shape of the age trajectories, the timing of peak prevalence and peak rate of increase, and the main sex differences were preserved. Stomach/abdominal pain was the only outcome for which the restricted trajectories differed appreciably; however, this restriction substantially reduced the contributing sample from 1,489,306 participants to 676,570 participants over the age of 18. We therefore retained the full harmonised dataset, while making the sampling-design limitations explicit.

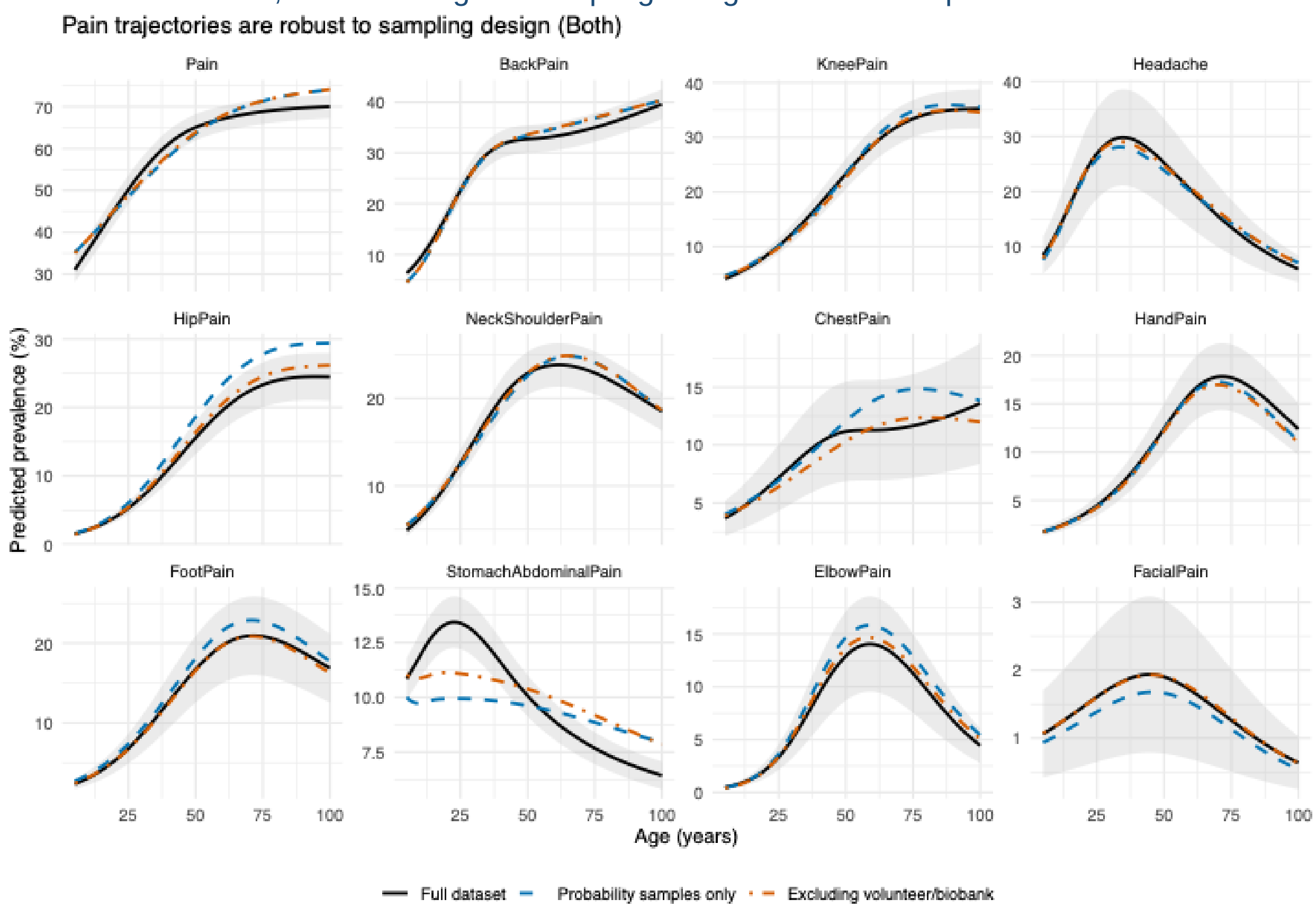

Sampling-design sensitivity (back pain, illustrative). Predicted age trajectory for the full harmonised dataset versus a model excluding volunteer/biobank-style cohorts; shaded bands are 95% CIs. The trajectory shape, peak timing, and prevalence are materially unchanged.

Changes to the manuscript: We clarified in the Methods and Discussion that contributing sources varied in sampling design and were not uniformly nationally representative. We added Supplementary Section 6.7 and Supplementary Fig. 13 comparing the primary trajectories with restricted-sample analyses excluding volunteer and biobank-style cohorts and restricting to probability-sampled general-population sources. These analyses showed that the principal age-

by-site trajectories were materially unchanged across outcomes, with the largest deviation observed for stomach or abdominal pain under reduced sample coverage.

Revised text (Discussion/Limitations): *Sampling designs also varied and were not uniformly nationally representative, although trajectories were materially unchanged in restricted-sample analyses except for stomach or abdominal pain, where the observed deviation coincided with a substantial reduction in sample size (Supplementary Section 6.7).*

Revised text (Supplement, Section 6.7): *To evaluate whether the inclusion of volunteer and biobank-style cohorts influenced the estimated reference trajectories, we repeated the primary age-by-sex models under two restricted sampling scenarios. The first excluded sources classified as volunteer or biobank-style cohorts. The second retained probability-sampled general-population sources where sufficient data were available. Across outcomes, the restricted trajectories closely reproduced the primary age-by-site patterns. Stomach or abdominal pain showed the largest deviation under restricted sampling, but the restricted trajectory coincided with a substantial reduction in contributing sample size from 1,489,306 to 676,570 participants over the age of 18. These findings indicate that the principal lifespan patterns were not driven by the inclusion of volunteer or biobank-style cohorts (Supplementary Fig. 13).*

1.4

The authors' state: "majority of sites (7 of 11) exhibited an inverted U trajectory." There are analyses that will let you test this assumption of being inverted U-shaped such as change point detection. Without these tests you cannot make blanket statements about the shape of the trajectories.

Response: This is a great point and we agree that the shape of each age trajectory should be evaluated formally rather than asserted qualitatively. We therefore added a formal trajectory-shape assessment based on the age-specific slope of the fitted prevalence curve. Specifically, for each pain site, we examined the first derivative of the model-estimated prevalence trajectory across age, together with its 95% confidence interval. A site was classified as showing an inverted-U pattern only when the slope was positive earlier in life and negative later in life, with the corresponding 95% confidence intervals excluding zero in both portions of the trajectory. This criterion identifies sites with evidence of an interior peak followed by a decline, rather than simply a visual plateau.

This formal assessment supports the original description but allows us to report it more precisely. Seven of the eleven anatomical sites met the inverted-U criterion: headache, stomach/abdominal, facial, elbow, neck/shoulder, hand, and foot pain. Their estimated interior peak ages ranged from approximately 23 years for stomach/abdominal pain to approximately 72 years for hand and foot pain. The remaining four sites, back, knee, hip, and chest pain, did not show evidence of a later-life decline and instead increased across the observed age range, peaking at or near the oldest ages.

We have revised the Methods to reflect the formal test and Results to replace the broad qualitative statement with a per-site description of trajectory shape, including estimated peak age and evidence for pre-peak rise and post-peak decline.

Changes to the manuscript: Revised the Methods and Results to report the formal trajectory-shape assessment rather than relying on the qualitative phrase "inverted-U."

Revised text (Results): *Using the age-specific rates of change from the fitted trajectories, the majority of sites (7 of 11) exhibited an inverted-U trajectory, with prevalence peaking in mid-to-late adulthood and declining thereafter. This pattern was most pronounced for headache (peak*

age 34 years) and abdominal pain (23 years), and across upper-body sites including neck or shoulder (62 years), elbow (59 years), and facial pain (45 years).

Revised text (Methods, Statistical analysis): *We used these age-specific rates of change to classify trajectory shape. A site was described as showing an inverted-U pattern only when the fitted trajectory showed both an early-life increase and a later-life decline, defined by 95% confidence intervals excluding zero for the rate of change in both phases.*

1.5

I find most of the figures unreadable given the small size of individuals panels (even on my large screen monitor). Particularly problematic is the use of shading to indicate 95% CI. In many cases, it is impossible to tell if the confidence intervals overlap.

Response: We thank the reviewer for raising this concern. We agree that the original confidence-interval shading was too faint in several panels, particularly where the curves closely overlap. We have therefore increased the opacity of the 95% CI bands in the trajectory figures to make the uncertainty intervals more visible and easier to distinguish.

We note that the figures contain many panels because the study reports lifespan trajectories across eleven anatomical sites and composite pain outcomes, stratified by sex. This small-multiple structure is necessary to present the full anatomical pattern of results in a comparable format. However, we agree that the uncertainty display should be clearer, and the revised rendering improves the visibility of the shaded intervals without changing the underlying estimates or figure structure.

We also note that visual overlap of confidence bands is not the most reliable way to determine whether groups differ at a given age. The formal age-specific contrasts are now provided in the complete relative-risk and risk-difference tables, with 95% confidence intervals, including comparisons whose intervals cross the null.

Changes to the manuscript: Increased the opacity of the 95% confidence-interval shading across the model-estimated trajectory figures. Specifically, the site-specific lifespan trajectories (Figure 2), the aggregate pain-measure trajectories (Figure 3), the regional and national-development (HDI) trajectories (Figure 4), and the exposure trajectories (Figure 5), together with the corresponding Extended Data trajectory figures, to improve legibility of the uncertainty bands at reduced panel sizes.

Revised text (figure rendering only): *No manuscript text was changed. The 95% confidence-interval bands in Figures 2–5 and the corresponding Extended Data trajectory figures were made more opaque to improve legibility at reduced panel sizes.*

1.6

Relative risk values are presented to indicate differences across groups. However, 95% CIs are only presented for selected variables for selected painful conditions. I would like to see tables containing the complete relative risk data for every model employed, whether the relative risk 95% CI crosses 1. I would like to see the output from regression models for all covariates in the given model. Survey year MUST be included as a covariate in all regression models.

Response: We thank the reviewer and address each of the three requests.

(a) Survey year. We have added survey year as a covariate to all regression models, centred on the sample mean so that estimates are standardised to the mean survey year. Adjusting for survey year produced negligible changes throughout: predicted prevalence shifted by a mean of <0.5 percentage points (maximum ~1.9 pp, for neck/shoulder pain), pain-intensity estimates by

≤0.1 points, and the modifiable-risk-factor relative risks by <1% on average (maximum ~2%). We now report the survey-year–adjusted models as our primary specification.

The HDI analysis presents a unique challenge because observations from lower-HDI settings are disproportionately drawn from earlier survey years, whereas higher-HDI settings are represented across a much broader temporal range. As a result, HDI and survey year are only partially observed independently, creating limited overlap in the HDI-survey-year space. To determine whether these shifts reflected confounding or lack of overlap, we performed a common-support sensitivity analysis. Specifically, we restricted the data to the range over which HDI and survey year were jointly observed and refit the models with survey year included. Under this restriction, the lower-HDI gradient was fully preserved (shown below; now provided in Supplementary Section 6.8 and Supplementary Fig. 14), indicating that the apparent reversal observed in the full-data year-adjusted model was driven by extrapolation resulting from limited overlap rather than evidence of a fundamentally different underlying association. We therefore retain the original HDI specification as our primary analysis and report both the survey-year-adjusted and common-support sensitivity analyses in the Supplement.

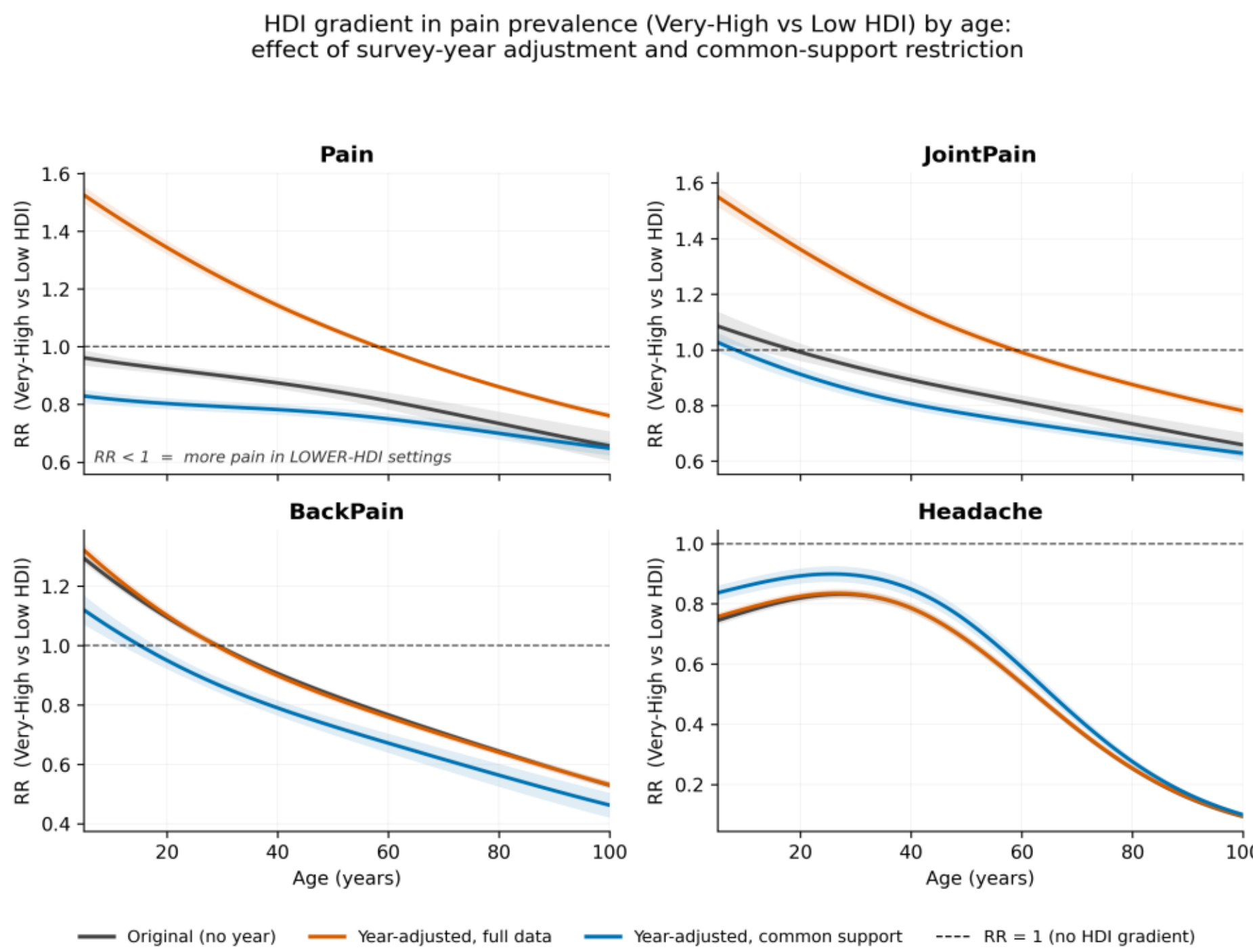

Supporting figure. Very-High-vs-Low-HDI risk ratio by age. The full-data year-adjusted curve (orange) reverses at young ages for any-pain and joint pain, but restricting to the region of common support (blue) preserves the lower-HDI gradient seen in the original model (black).

(b) Complete relative-risk tables. We now provide the complete relative-risk results for every model and comparison across all pain sites and age bands, and including comparisons whose 95% CI crosses 1 in a revised Supplementary Table 6 and as a machine-readable file.

(c) Output for all covariates. We additionally provide the full fixed-effects output (estimate, SE, 95% CI, p) for every covariate in every model as Supplementary Data.

Changes to the manuscript: (i) Methods – Statistical analysis: added sentences stating all models include survey year (centred; standardised to the mean survey year), with the HDI exception and its justification. (ii) Revised Supplementary Table 6 (year-adjusted RRs with 95% CIs, all comparisons incl. CI-crossing-1; FDR-flagged). (iii) New Supplementary Data file with full model coefficients (all covariates, 94 models). (iv) HDI common-support sensitivity analysis

now added to the Supplement (Section 6.8, Supplementary Fig. 14); the HDI-by-year collinearity diagnostic is reported in this response. (v) Updated in-text statistics to reflect the year-adjusted primary models and the expanded dataset (e.g., the site prevalence range is now reported as approximately 2% for facial pain to 40% for back pain, with peak-age, sex, and exposure estimates updated accordingly).

Revised text (Methods, Statistical analysis): *...adjusted for methodological differences using fixed effects for recall period and case definition, and, except for the HDI models, survey year (centred on the sample mean). Survey year was omitted from the HDI models because HDI is assigned by country-year and showed limited overlap with survey year across the pooled dataset, making joint adjustment prone to extrapolation.*

Revised text (Supplement, Section 5.3): *The HDI models were the single exception to the survey-year adjustment described above: because country-level HDI is assigned at the survey year and is itself time-varying, HDI and survey year are only partially observed independently, and jointly adjusting for both would extrapolate beyond the region of overlapping data; the HDI trajectories were therefore estimated without a survey-year term.*

1.7

The number of comparisons must be near or over a hundred. Given this, some correction to alpha must be made.

Response: We agree. We now applied a Benjamini-Hochberg false-discovery-rate (FDR) correction [6] across the full family of reported relative-risk comparisons (665 comparisons spanning sex, obesity, overweight, smoking, income, and HDI contrasts across all pain sites and age bands). The comparisons no longer meeting the 5% FDR threshold were concentrated at the youngest and oldest age extremes and corresponded to small effect sizes close to the null. The age-dependent trajectories, relative-risk patterns, and all substantive conclusions were unchanged. These new statistics are provided in Supplementary Table 6, with exact q-values in the accompanying machine-readable file.

Changes to the manuscript: Methods – Statistical analysis: added a sentence describing the Benjamini–Hochberg FDR correction. Supplementary Table 6 now flags FDR-adjusted significance; the machine-readable file reports exact q-values.

Revised text (Methods, Statistical analysis): *Relative risks and their 95% confidence intervals were computed for all sex, exposure, and HDI contrasts across pain sites and age bands, and a Benjamini–Hochberg false-discovery-rate correction was applied across this full family of comparisons to account for multiple testing (Supplementary Table 6).*

1.8

There is no real table 1 showing the overall demographic characteristics of the harmonized combined data set. What are the proportions by sex, age, region, etc. Survey year should be included.

Response: We have now added a demographic table (Supplementary Table 1) summarising the harmonised dataset overall and by world region, reporting the number of contributing data sources and countries, the analytic sample size, the proportion female, age (median and interquartile range), and the survey year (median and range). We note that the analytic sample is now slightly larger than at submission (6,125,459 vs 6,075,021 participants; 902 vs 894 data sources), as eight additional eligible data sources became available during revision; all key estimates and conclusions were materially unchanged.

Changes to the manuscript: New Supplementary Table 1 with demographic characteristics by world region, including data-source and country counts and survey year. Abstract, Results, and Methods sample sizes updated to the expanded dataset (902 data sources; N = 6,125,459).

Revised text (Results, Study population): *We analysed a total of 6,125,459 participants across 902 data sources from 138 population-based study programmes, spanning 118 countries and territories (figure 1A). Demographic characteristics of the analytic sample are summarised by world region in Supplementary Table 1.*

Revised text (Supplement): *New Supplementary Table 1, "Demographic characteristics of the analytic sample, by world region," reporting data-source and country counts, N (analytic sample), % female, age median (IQR), and survey-year median (range) per region.*

1.9

I do not like stacked graphs e.g. extended data figure 1. They are impossible to read and interpret. Confidence intervals must always be presented.

Response: We thank the reviewer for this point and have revised the presentation of Extended Data Fig. 1 accordingly. We agree that uncertainty should be available for all estimates shown or summarised in the figure. To address this, we have added two complementary sources of uncertainty. First, we added a supplementary table reporting the adjusted prevalence and 95% confidence interval for every pain site, exposure level, and age band underlying Extended Data Fig. 1. Second, within each exposure panel of Extended Data Fig. 1 (A, smoking; B, BMI; C, income) we added the expected number of co-occurring pain sites, calculated as the summed adjusted site-level prevalence across anatomical sites, by age, with 95% confidence bands. This overlay preserves the anatomical-composition information of the stacked figure while displaying uncertainty on the cumulative burden directly.

We have retained the stacked figure because it compactly displays the anatomical composition of pain burden across exposure groups, but the revised manuscript now provides uncertainty estimates both for the site-specific components and for the total accumulated burden.

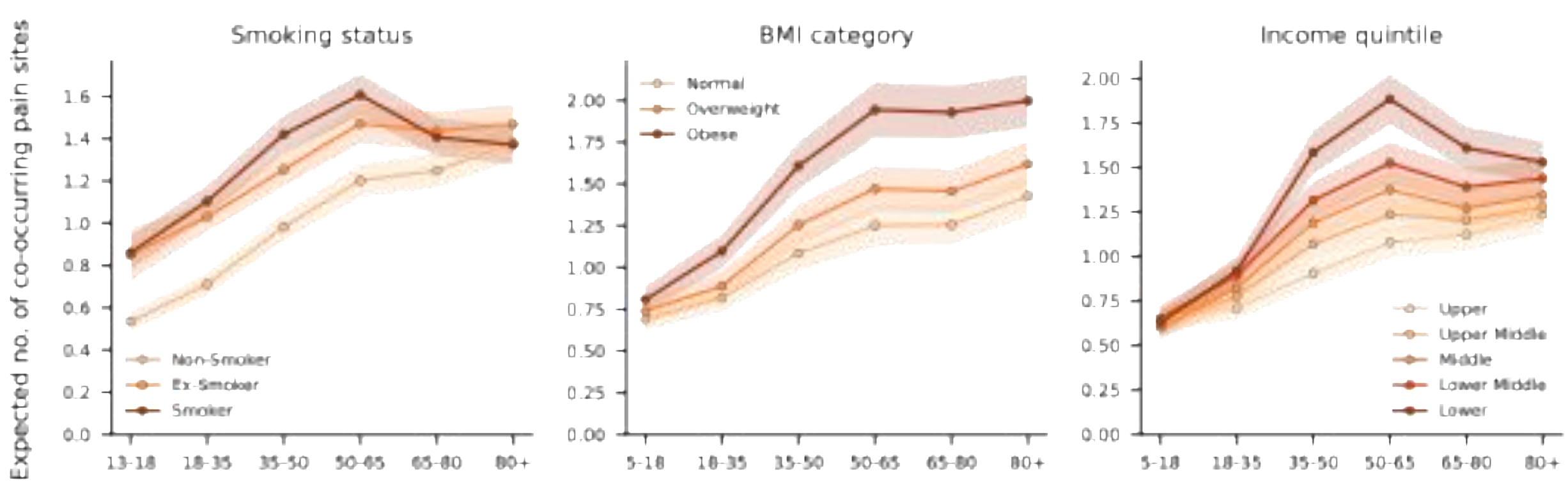

Revised Extended Data Fig. 1. Each exposure panel (A, smoking; B, BMI; C, income) shows the stacked anatomical composition of pain burden together with the expected number of co-occurring pain sites (summed adjusted site-level prevalence); shaded bands are 95% CIs on the cumulative quantity.

Changes to the manuscript: Revised Extended Data Fig. 1 so that each exposure panel (A, smoking; B, BMI; C, income) now shows both the stacked anatomical composition and the expected number of co-occurring pain sites with 95% confidence bands. Provided the adjusted prevalence and 95% confidence interval for every pain site, exposure level, and age band underlying Extended Data Fig. 1 as an ancillary Supplementary Data file. Revised the Extended

Data Fig. 1 legend accordingly and to cross-reference the ancillary prevalence file and the complete relative-risk results in Supplementary Table 6.

Revised text (Extended Data Fig. 1 legend): *Pain burden across the lifespan by smoking, BMI, and household income. Upper-row stacked bars show age-group-specific, site-level prevalence (%) for each anatomical pain site, stratified by (A) smoking status, (B) BMI category, and (C) household income quintile. Because individuals can report pain at multiple sites, site-level prevalences are not mutually exclusive and stacked totals can exceed 100%. Lower-row line plots show the expected number of co-occurring pain sites for the same age groups and exposure categories, calculated by summing site-level prevalence across anatomical sites. Shaded bands indicate 95% confidence intervals. Smoking results are shown from ages 13 years and older.*

1.10

While it is clear there is a truly international group of investigators involved with this project, the roles of these individuals are not specific. Are they all truly deserving of authorship? Do they make SUBSTANTIAL contributions? Nature Medicine lists these requirements for authorship: "each author is expected to have made substantial contributions to the conception or design of the work; or the acquisition, analysis, or interpretation of data; or the creation of new software used in the work; or have drafted the work or substantively revised it." "Contributors who do not meet all criteria for authorship should be listed in the Acknowledgements section."

Response: We thank the reviewer for raising this important point. We agree that a large international author group requires a clear and transparent account of each contributor's role.

All retained authors made substantial contributions to one or more components of the work, including study conception and design, acquisition and curation of population-based data, cohort-specific interpretation, measurement and harmonisation decisions, statistical analysis, interpretation of findings, or substantive revision of the manuscript. Many collaborators provided access to or stewardship of data sources that could not be obtained through public repositories, but their contributions extended beyond the provision of data. They advised on study design, sampling frames, variable definitions, measurement validity, population context, and the limitations of their respective data sources; contributed to decisions about whether and how variables could be harmonised; and reviewed the interpretation of findings in their regional or cohort-specific context.

These contributions were essential to the scientific validity of an individual-participant-data study spanning 118 countries and 902 data sources. The project was not conducted solely as a centralized statistical exercise. It relied on substantive input from investigators with direct knowledge of the contributing populations, study programmes, cultural contexts, and pain-measurement practices, including experts who lead pain research within the represented regions. This distributed expertise was necessary both to construct a defensible harmonised dataset and to interpret the resulting global and regional patterns appropriately.

We have also clarified that authorship was not assigned solely on the basis of general endorsement or nominal consortium membership. Contributors who provided data access, cohort expertise, methodological input, or substantive manuscript revision are retained as authors; individuals or groups whose contributions do not meet the journal's authorship criteria are listed in the Acknowledgements rather than as authors. This revision better reflects the collaborative structure of the project, which required expertise across pain epidemiology, paediatric pain, public health, survey methodology, and regional population health.

Reviewer 2

This manuscript provides population-based estimates of pain prevalence across 118 countries and over 6 million people, including data from all habituated continents. In developing their estimates, the investigators harmonized individual-level self-reported pain data across 894 population-based data sources to establish global reference trajectories of pain. The investigators report pain prevalence estimates ranging from 2.5% for facial pain to 45.0% for back pain. Estimates across all sites of pain were consistently higher among women, with prevalence estimates generally peeking before age 55. Investigators addressed hypothesis-driven key knowledge gaps and reported that pain prevalence was generally higher in lower Human Development Index (HDI) regions, and that pain burden was largely accounted for by three modifiable risk factors, namely smoking, obesity, and low income. One particularly important recommendation is to continue to systematically examine key contributors to pain in lower-HDI settings that remain poorly characterized.

This manuscript is highly significant and innovative. It has the potential to stand as a key resource that can inform global health policy, practice, and scientific inquiry related to pain and pain management by providing rigorously developed and reliable pain prevalence and burden estimates, along with important information about sex differences, age trajectories, regional variation, associations with health, education, and income, and modifiable risk factors.

This manuscript has numerous strengths and few relative weaknesses. It is well-written, concise, and clear. The focus on a limited number of significant gaps and hypotheses related to sex, age, income, and modifiable risk factors is an important asset. The background provided supports these focuses and provides a compelling rationale and context for the significance of the report. Key ethical and regulatory issues are appropriately addressed related to data sharing and data use. The authors provide a compelling rationale for the use of self-report data as opposed to data from health care records since limitations of use of health care resources in some countries and regions may seriously compromise the reliability and validity of these data. The analytic approaches are well-informed and carefully described, leading to confidence in the data and the analyses. Key limitations are noted. The conclusions are appropriate. References are appropriate.

Response: We are grateful to Reviewer 2 for this positive and thorough assessment. We address the specific suggestions below.

2.1

The long-time frame (1990-2025) for the study is a strength (i.e., more data from more countries) and also a serious limitation since pain prevalence, surveillance and reporting methods, and care and care access is dynamic. This observation could be specifically noted.

Response: We agree with the reviewer. The long temporal coverage is a strength because it increases geographic and age coverage, particularly in regions where contemporary pain surveillance remains limited, but it also introduces potential temporal heterogeneity in pain prevalence, reporting practices, surveillance methods, and access to care.

As described in response to Reviewer 1.6, we have now added survey year as a covariate to all primary regression models, centred on the sample mean, and report the survey-year-adjusted models as the primary specification. This adjustment had minimal impact on the estimated trajectories and substantive conclusions: predicted prevalence shifted by a mean of <0.5

percentage points, pain-intensity estimates by ≤0.1 points, and modifiable-risk-factor relative risks by <1% on average.

We have additionally expanded the Limitations to state explicitly that the 1990–2025 observation window should be interpreted as both a strength and a limitation. Specifically, we now note that pain prevalence, survey wording, surveillance infrastructure, health-care access, and norms around symptom reporting may have changed over this period, and that the resulting trajectories should therefore be interpreted as harmonised reference estimates across available historical and contemporary data rather than as a direct description of secular change.

> **Changes to the manuscript:** Discussion/Limitations: added text acknowledging that the 1990–2025 timeframe may introduce temporal heterogeneity in pain prevalence, reporting, surveillance practices, and care access. Methods/Statistical analysis: added survey year as a covariate to the primary models, with year-adjusted estimates reported as the primary specification.
>
> **Revised text (Discussion/Limitations):** *The broad 1990–2025 observation window increased geographic and age coverage but also combines historical and contemporary data collected under changing survey practices, care access, case definitions, and symptom-reporting norms. Although primary models adjusted for survey year and this had minimal impact on the estimated trajectories, these curves should not be interpreted as secular trends in pain prevalence.*

2.2

More details about how data used in the study were identified and incorporated could be useful. Any risk of selection bias should be acknowledged.

Response: Details of the data-identification and access process are provided in the Supplementary Methods. We have now expanded the Methods to summarize the multi-stage data-identification process. Briefly, data sources were identified through literature searches, public microdata repositories, national and international survey programmes, and direct contact with investigators and data custodians. Searches included PubMed, Google Scholar, the Global Health Data Exchange, ICPSR, the WHO Microdata Library, the World Bank Microdata Catalog, Harvard Dataverse, the UK Data Service, national statistics portals, and direct applications or requests to large cohorts, biobanks, COPCORD investigators, and related rheumatology programmes. Publicly available datasets were downloaded directly, whereas restricted-access studies were incorporated through data-use agreements, formal data-access applications, or standardized local analysis protocols where individual-level transfer was not permitted.

We have also expanded the limitations to acknowledge potential selection bias. Inclusion depended not only on whether a study measured pain, but also on the availability of individual-level data, data-access permissions, repository coverage, institutional data-protection rules, and the responsiveness of investigators or data custodians. As a result, countries and study programmes with stronger surveillance infrastructure, public data repositories, or established data-sharing mechanisms may be overrepresented, while settings where pain data were not collected, not digitized, not publicly indexed, or not shareable may be underrepresented. We therefore clarify that the study provides harmonised reference trajectories from the available individual-level evidence base rather than a complete census of all pain surveys conducted globally.

> **Changes to the manuscript:** We expanded the Methods/Data sources section to summarize the multi-stage source-identification and incorporation process already described in the Supplementary Methods, including literature searches, repository searches, public microdata downloads, direct investigator contact, data-access applications, and standardized local analysis where individual-level transfer was not permitted. We also expanded the

Discussion/Limitations to acknowledge possible selection bias due to data availability, repository coverage, data-access permissions, institutional data-protection rules, and uneven global surveillance infrastructure.

Revised text (Methods, Data sources): *Data sources were identified and acquired between April 2024 and June 2026 through structured searches of bibliographic databases and public microdata repositories, review of national and international survey programmes, and direct contact with investigators, data custodians, cohort and biobank administrators, and rheumatology networks. Publicly available datasets were downloaded directly, whereas restricted-access studies were incorporated through data-use agreements, formal data-access applications, or standardised local analysis protocols when individual-level data transfer was not permitted.*

Revised text (Discussion/Limitations): *Finally, the database may be affected by selection bias in which studies measured pain, made individual-level data available, received data-access approval, or could be incorporated through investigator collaboration.*

2.3

The rationale for focusing on pain sites as opposed to clinical diagnostic information is appropriate and an asset. At the same time, additional details to support this decision and the sites selected could be helpful. It could have been useful to consider pain at multiple sites were managed and "whole body pain." The concept of chronic overlapping pain conditions might be acknowledged.

Response: We appreciate this comment and agree that the rationale for the site-based framework should be stated more clearly. The manuscript already contrasts our self-reported, site-based framework with diagnosis-based disease surveillance initiatives such as GBD estimates for migraine, osteoarthritis, neck pain, and low back pain. We have revised the Introduction and Discussion to clarify why anatomical pain sites, rather than clinical diagnoses, were used as the primary organising framework.

This choice reflects both the aims of the study and the structure of the available data. Clinical diagnoses require diagnostic evaluation, health-care access, and condition-specific criteria that vary substantially across countries, age groups, and study designs. Diagnosis-based estimates can therefore under-ascertain pain in settings with limited access to care or clinical surveillance. By contrast, site-specific self-reported pain is more consistently available across population-based surveys and allows pain burden to be compared across the lifespan and across regions, including in settings where formal diagnostic data are sparse or unavailable. The eleven anatomical sites analysed here capture both musculoskeletal and non-musculoskeletal pain phenotypes that showed distinct epidemiological trajectories, including early peaks for headache and stomach/abdominal pain, midlife peaks for several upper-body sites, and later-life increases for lower-body musculoskeletal sites.

We agree that multi-site and whole-body pain are important related constructs. The manuscript already includes generalised pain as a secondary outcome, operationalised using the 2016 ACR fibromyalgia spatial criteria, which applies an established spatial definition of widespread pain rather than a simple count of painful sites. We have expanded the Discussion to make clearer that site-specific pain should be interpreted alongside broader measures of multi-site, generalised, and overlapping pain phenotypes.

To address the reviewer's specific suggestion, we also added a new descriptive analysis of single-region versus multi-region pain across the lifespan. We grouped anatomical pain reports into seven non-overlapping regions: head/face, neck/shoulder, back, chest, abdomen, upper limb, and lower limb. To ensure adequate body coverage, this analysis was restricted to studies

that assessed at least four of the seven regions, yielding 101 studies and approximately 2.44 million participants. Single-region pain was defined as pain in exactly one region, and multi-region pain as pain in two or more regions. Multi-region pain increased more steeply with age than single-region pain, overtook it around age 30, peaked near 29% in the mid-60s, and then declined, converging with single-region pain by the oldest ages. This analysis complements the generalised-pain results and shows how co-occurring pain burden accumulates across the lifespan within the site-based framework.

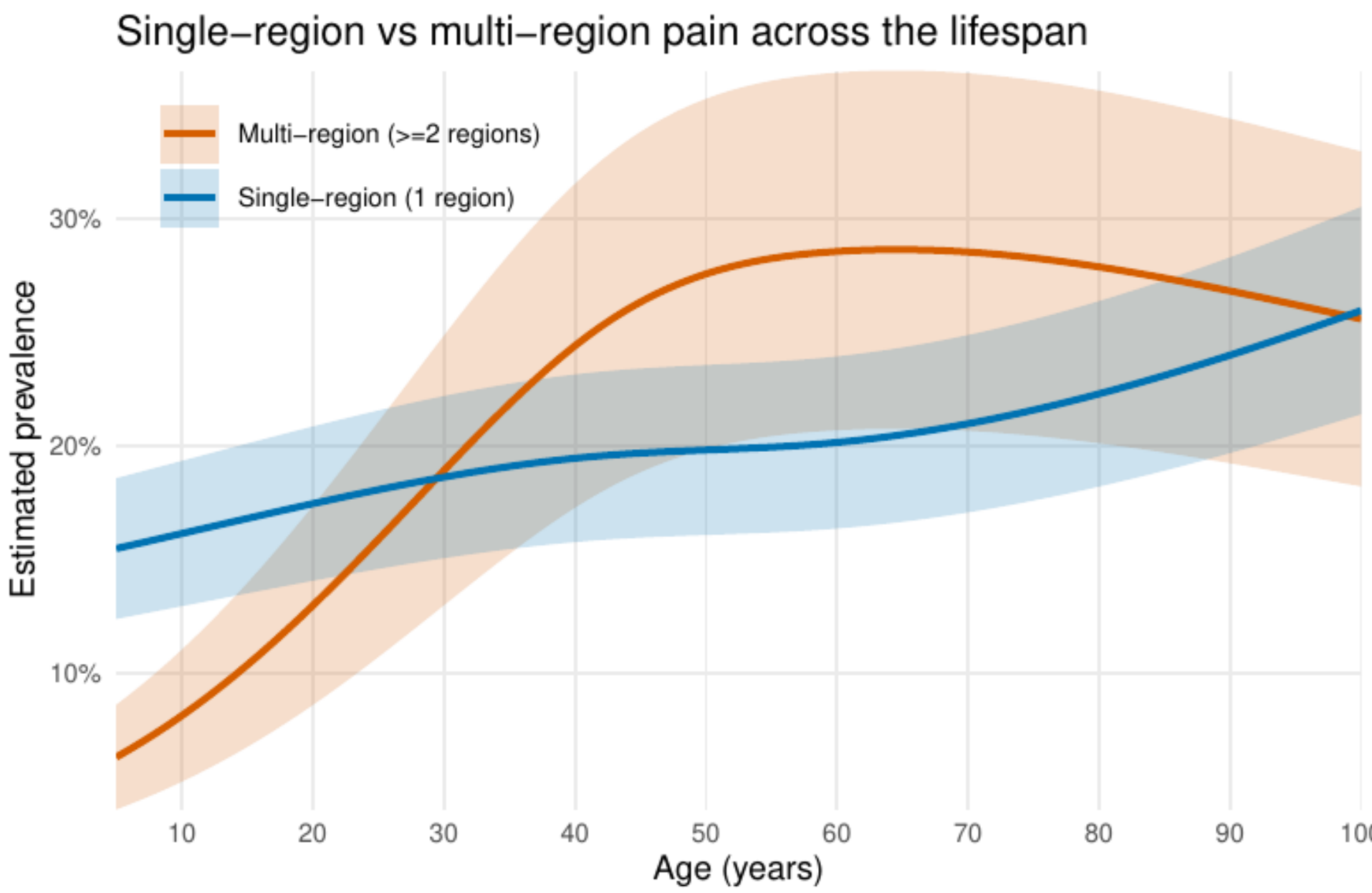

Single-region vs multi-region pain across the lifespan (population prevalence; pain reports grouped into seven body regions; studies assessing ≥4 of 7 regions; 101 studies, ~2.44 million participants). Shaded bands are 95% CIs.

Changes to the manuscript: We revised the Introduction and Discussion to better integrate the rationale for using a site-based self-report framework alongside diagnosis-based disease surveillance approaches, including GBD estimates for migraine, osteoarthritis, neck pain, and low back pain. The revised text clarifies that clinical diagnoses depend on diagnostic work-up, health-care access, and condition-specific definitions that vary across settings, whereas site-specific self-report can capture pain burden in populations where formal diagnostic data are sparse or unavailable. We also expanded the discussion of generalised and multi-site pain and added a supplementary analysis contrasting single-region and multi-region pain prevalence across the lifespan, grouping pain reports into seven body regions and including studies that assessed at least four regions (101 studies, approximately 2.44 million participants; Supplementary Fig. 14).

Revised text (Introduction): *Existing frameworks, including the Global Burden of Disease (GBD) study, rely on clinically defined conditions and modelled estimates of heterogeneous data sources. These approaches are essential for disease surveillance but may not fully capture pain that does not enter diagnostic pathways, particularly in settings with limited access to care. Site-specific self-reported pain provides a complementary framework because anatomical pain reports are more consistently available across population-based studies than diagnostic information and can be compared across settings where clinical work-up, diagnostic criteria, and health-system access vary.*

Revised text (Discussion): *Because pain often occurs across multiple locations and may reflect broader overlapping pain phenotypes, we also examined generalised pain using the 2016*

ACR fibromyalgia spatial criteria and single-region versus multi-region pain among studies with sufficient anatomical coverage (Supplementary Fig. 14). These complementary analyses showed that co-occurring pain burden increases substantially across adulthood, reinforcing that site-specific trajectories should be interpreted alongside broader measures of multi-site and generalised pain.

Revised text (Supplementary Fig. 14 legend): *Single-region versus multi-region pain across the lifespan. Model-estimated population prevalence (%) of single-region pain (pain in exactly one of seven non-overlapping anatomical regions) and multi-region pain (pain in two or more of these regions), restricted to studies that assessed at least four of the seven regions (101 studies, approximately 2.44 million participants). Shaded bands are 95% confidence intervals.*

2.4

The investigators note an "inverted U" for the age and pain prevalence association is an important observation, although this is not novel or unique to this study. A more detailed discussion of the varying hypotheses that could account for this observation could be included. For example, some have hypothesized a cohort effect, that is: that older persons may report more widespread pain leading to an underreporting of site-specific pain; and/or pain interference among younger persons may be more salient than pain experienced in older persons (context matters).

Response: We thank the reviewer for this helpful suggestion. We agree that inverted-U age patterns are not unique to this study and that the manuscript should discuss plausible explanations more explicitly. As described in response to Reviewer 1.4, we have also revised the Results to characterize trajectory shape formally using the age-specific slope of the fitted prevalence curves rather than relying on a qualitative visual description.

We have expanded the Discussion to acknowledge several non-mutually exclusive explanations for the observed inverted-U patterns. Some patterns likely reflect true site-specific population epidemiology. For example, headache, facial pain, and stomach/abdominal pain peaked relatively early in life and declined thereafter, consistent with trajectories that differ biologically and clinically from lower-body musculoskeletal pain. In contrast, back, hip, and knee pain showed continued increases into later life or peaks at the oldest ages. This site-specific heterogeneity argues against a single explanation such as survivorship, cohort effects, or generalized underreporting in older age; if those factors were the dominant driver, we would expect later-life declines to appear more uniformly across pain sites.

At the same time, we agree that cohort, reporting, and contextual effects may contribute to the apparent late-life decline in some site-specific outcomes, particularly for several musculoskeletal sites. Older adults may appraise or report pain differently [9], may experience pain across multiple regions rather than as a single discrete site, or may be less likely to identify one dominant anatomical location when pain is more widespread. We have revised the Discussion to make clear that these mechanisms cannot be fully separated in the present cross-sectional harmonised dataset, and that the trajectories should be interpreted as population age-patterns in self-reported site-specific pain rather than definitive within-person aging effects.

We also now cross-reference the generalized-pain and single-region versus multi-region analyses, which provide complementary information on the age distribution of co-occurring pain.

Changes to the manuscript: We expanded the Discussion to clarify that inverted-U trajectories may reflect true site-specific life-course processes, such as earlier peaks in headache and abdominal pain and later accumulation of musculoskeletal pain, but may also reflect cohort or reporting effects. The revised text now notes that older adults may appraise or localise pain

differently, particularly when pain is widespread, and that the cross-sectional design prevents these explanations from being fully separated from within-person ageing effects.

Revised text (Discussion): *These patterns may reflect true site-specific life-course processes, including earlier peaks in headache and abdominal pain and later accumulation of musculoskeletal pain, but cohort and reporting effects may also contribute. Older adults may appraise pain differently, experience pain across multiple regions rather than as a discrete site, or be less likely to identify a single dominant location when pain is widespread. Because most contributing studies were cross-sectional, these explanations cannot be fully separated.*

2.5

The rationale and empirical and conceptual support for focusing on smoking, obesity, and income relative to other variables could be provided. What about employment status or type of work? Housing security? Food insecurity?

Response: We thank the reviewer for this helpful suggestion and have expanded the rationale for the selected risk factors. Smoking, obesity/BMI, and household income were specified a priori rather than selected post hoc. We focused on these three exposures because they have strong prior empirical support as correlates of pain [10–12], align with major global burden-of-disease risk-factor frameworks [2], and were available in a sufficiently large and harmonisable subset of contributing datasets to support pooled estimation. Together, they span complementary behavioural, metabolic, and socioeconomic dimensions of pain risk.

We agree that employment status, occupation and type of work, housing security, and food insecurity are important and plausibly informative determinants of pain. However, these variables were not consistently available across the included studies, and when available they were often measured using study-specific definitions that could not be harmonised reliably at global scale. For example, employment and occupational measures varied in whether they captured labour-force status, job title, physical workload, informal work, or retirement; food insecurity and housing security were available in only a limited subset of studies. We therefore did not include these variables in the pooled primary exposure analyses, but now identify them explicitly as important priorities for future work.

We revised the Methods and Discussion accordingly, framing smoking, obesity/BMI, and income as a focused set of empirically supported and harmonisable exposures, while acknowledging that other social and occupational determinants are likely important but require dedicated study.

Changes to the manuscript: We revised the Methods to clarify that smoking, obesity/BMI, and household income were prespecified exposures selected based on prior empirical support, alignment with global burden-of-disease risk-factor frameworks, and sufficient cross-study harmonisability, while spanning behavioural, metabolic, and socioeconomic domains. We also revised the Discussion to identify employment status and type of work, housing security, and food insecurity as important social and occupational determinants that were not consistently available or harmonisable across contributing datasets and require dedicated future study.

Revised text (Methods, Individual risk factors): *We evaluated a prespecified set of three modifiable or policy-relevant exposures with established associations with pain outcomes across the literature: body mass index, smoking status, and household income. These exposures were selected based on prior empirical support, alignment with global burden-of-disease (GBD) risk-factor frameworks, and sufficient cross-study harmonisability to support pooled estimation. Together, they capture metabolic, behavioural, and socioeconomic dimensions of pain risk. Other social and occupational determinants, including employment*

status, type of work, housing security, and food insecurity, were not consistently available or harmonisable across the contributing datasets and require dedicated study.

2.6

The paragraph on limitations is important; could this be expanded with some examples of variance in the way presence of pain was measured and the approach for operationalization.

Response: We thank the reviewer for this suggestion. Detailed measurement and operationalisation procedures are described in the Supplementary Methods, including the anatomical harmonisation definitions and inclusion criteria (Supplementary Section 3; Supplementary Table 3) and the methodological-covariate and recall-period sensitivity analyses (Supplementary Sections 6.2–6.3). We agree that the main limitations paragraph should include more concrete examples.

We therefore revised the limitations to specify that contributing studies varied in recall period, including current, past-week, past-month, and 3- to 12-month windows; case definition, including general pain versus chronic-explicit pain lasting longer than 3 months; question format, including direct site-specific questions versus body-map coding; and anatomical resolution, including separately assessed sites versus grouped regions such as neck/shoulder, hand/wrist, foot/ankle, or stomach/abdominal pain. We also clarified that these differences were operationalised through the harmonisation framework and evaluated empirically in sensitivity analyses, while acknowledging that residual measurement heterogeneity may still influence absolute prevalence estimates.

Changes to the manuscript: We revised the Discussion/Limitations to include concrete examples of measurement variation across studies, including recall period, case definition, question format, body-map coding, and anatomical resolution. We also added clearer cross-references to the Supplementary Methods describing anatomical harmonisation and methodological sensitivity analyses (Supplementary Section 3; Supplementary Table 3; Supplementary Sections 6.2–6.3).

Revised text (Discussion/Limitations): *This point is addressed by the same Discussion/Limitations passage quoted under 1.1 (concrete examples of measurement variation: recall period, case definition, question format, body-map coding, and anatomical resolution, with cross-references to Supplementary Section 3; Supplementary Table 3; Supplementary Sections 6.2–6.3).*

In conclusion, I consider this manuscript to be particularly strong and likely to have a major impact on the field, both in terms of the methodology and findings. Limitations are all minor and are overshadowed by many methodological and other strengths and the significance of the report.

Response: We thank the reviewer again for the supportive and enthusiastic evaluation.

References

1. Madans JH, Loeb ME, Altman BM. Measuring disability and monitoring the UN Convention on the Rights of Persons with Disabilities: the work of the Washington Group on Disability Statistics. BMC Public Health. 2011;11(Suppl 4):S4.
2. GBD 2021 Risk Factors Collaborators. Global burden and strength of evidence for 88 risk factors in 204 countries and territories, 1990–2021: a systematic analysis for the Global Burden of Disease Study 2021. Lancet. 2024;403(10440):2162–2203.
3. NCD Risk Factor Collaboration (NCD-RisC). Worldwide trends in body-mass index, underweight, overweight, and obesity from 1975 to 2016: a pooled analysis of 2416 population-based measurement studies in 128.9 million children, adolescents, and adults. Lancet. 2017;390(10113):2627–2642.
4. O'Connell NE, Cook CE, Wand BM, Ward SP. Clinical guidelines for low back pain: a critical review of consensus and inconsistencies across three major guidelines. Best Pract Res Clin Rheumatol. 2016;30:968–980.
5. The IASP clinical-practice-guideline fact sheet, which explicitly explains why committees examining the same topic may reach conflicting conclusions.
6. Benjamini Y, Hochberg Y. Controlling the false discovery rate: a practical and powerful approach to multiple testing. J R Stat Soc Series B Stat Methodol. 1995;57(1):289–300.
7. Wolfe F, Clauw DJ, Fitzcharles MA, et al. 2016 Revisions to the 2010/2011 fibromyalgia diagnostic criteria. Semin Arthritis Rheum. 2016;46(3):319–329.
8. Maixner W, Fillingim RB, Williams DA, Smith SB, Slade GD. Overlapping chronic pain conditions: implications for diagnosis and classification. J Pain. 2016;17(9 Suppl):T93–T107.
9. Gagliese L. Pain and aging: the emergence of a new subfield of pain research. J Pain. 2009;10(4):343–353.
10. Shiri R, Karppinen J, Leino-Arjas P, Solovieva S, Viikari-Juntura E. The association between smoking and low back pain: a meta-analysis. Am J Med. 2010;123(1):87.e7–87.e35.
11. Okifuji A, Hare BD. The association between chronic pain and obesity. J Pain Res. 2015;8:399–408.
12. Poleshuck EL, Green CR. Socioeconomic disadvantage and pain. Pain. 2008;136(3):235–238.

Response to Reviewers

Manuscript ID: NMED-A151857A
Title: "Global and regional reference curves for pain across the lifespan in 6.1 million individuals in 118 countries"

We thank the reviewers for their careful and constructive assessment of our revised manuscript. Reviewer 2 requested no further changes. Reviewer 1 raised two remaining points, which we address below. The reviewer comments are reproduced verbatim.

Reviewer 1

Comment 1: Comparability of survey instruments

Remarks to the Author:

For the most part the authors have adequately addressed many of the specific concerns expressed by this reviewer. However, I still strongly question the validity of combining data from surveys using very different questions and approaches to collect data. The authors cite they are replicating the approach taken by Global Burden of Disease Study (GBDS). Perhaps I am one of the few population-based epidemiologists who does not think the comparisons made in the GBDS are valid. I certainly never cite it. At this point it is unlikely the journal will reject this manuscript. However, I still think a large supplement should list EVERY question from EVERY country that was combined in this study, so that the reader can judge themselves as to the comparability of the survey questions.

Response:

We thank Reviewer 1 for raising this concern. We agree that readers should be able to assess the comparability of the survey instruments directly. We have therefore added Supplementary Data 5 to the accompanying Supplementary Data workbook. It provides the verbatim primary pain questions for every contributing study, together with the countries covered and the pain-intensity questions where collected. Where a study used different wording or recall periods for specific pain sites, the additional site-specific questions are also included. For multicountry survey programmes that administered a common instrument, the entry identifies all countries covered. This information allows readers to evaluate the comparability and heterogeneity of the survey questions directly.

Comment 2: Code availability

Remarks on code availability:

I could not find the study's information using the provided URL.

Response:

We thank Reviewer 1 for identifying this problem. The previously provided URL directed readers to our laboratory's GitHub organisation rather than to the project repository. We have corrected the link in the Code Availability statement. The annotated analysis code is publicly available at:

https://github.com/EVPlab/lifespan-pain-trajectories

The public repository includes a README, an open-source licence and a synthetic-data demonstration.

Reviewer 2

Remarks to the Author:

Thanks to the authors for a particularly comprehensive, thoughtful, and scholarly response to reviewers' concerns and recommendations on the original manuscript. I am pleased that responses reflect a high level of respect for each of the prior reviewers' comments. It is clear that in most cases, the authors have made substantial efforts to address reviewer recommendations, have accepted the reviewer concerns, and have taken steps to address them. In the few cases that the authors have decided not to pursue reviewer recommendations, I found their arguments for not pursuing recommended changes to be strongly supported by a well-articulated conceptual or empirical basis, and I cannot disagree with their decisions. In the end, I am entirely satisfied with the authors' responses to prior critiques, with the additional analyses they conducted, and with revisions to the manuscript. I have no additional questions or comments that require further response from the authors. I am very enthusiastic about the work of these authors and the revised manuscript.

Response:

We thank Reviewer 2 for these generous comments and for the careful assessment of our responses, additional analyses and revised manuscript.

We again thank both reviewers for their careful and constructive evaluation of our work.

Etienne Vachon-Presseau
on behalf of all authors
